# Searching for Drug Synergy in Complex Dose–Response Landscapes Using an Interaction Potency Model

**DOI:** 10.1016/j.csbj.2015.09.001

**Published:** 2015-09-25

**Authors:** Bhagwan Yadav, Krister Wennerberg, Tero Aittokallio, Jing Tang

**Affiliations:** Institute for Molecular Medicine Finland (FIMM), FI-00014, University of Helsinki, Finland

**Keywords:** Drug combination scoring, Interaction landscape, Dose–response matrix, High-throughput screening

## Abstract

Rational design of multi-targeted drug combinations is a promising strategy to tackle the drug resistance problem for many complex disorders. A drug combination is usually classified as synergistic or antagonistic, depending on the deviation of the observed combination response from the expected effect calculated based on a reference model of non-interaction. The existing reference models were proposed originally for low-throughput drug combination experiments, which make the model assumptions often incompatible with the complex drug interaction patterns across various dose pairs that are typically observed in large-scale dose–response matrix experiments. To address these limitations, we proposed a novel reference model, named zero interaction potency (ZIP), which captures the drug interaction relationships by comparing the change in the potency of the dose–response curves between individual drugs and their combinations. We utilized a delta score to quantify the deviation from the expectation of zero interaction, and proved that a delta score value of zero implies both probabilistic independence and dose additivity. Using data from a large-scale anticancer drug combination experiment, we demonstrated empirically how the ZIP scoring approach captures the experimentally confirmed drug synergy while keeping the false positive rate at a low level. Further, rather than relying on a single parameter to assess drug interaction, we proposed the use of an interaction landscape over the full dose–response matrix to identify and quantify synergistic and antagonistic dose regions. The interaction landscape offers an increased power to differentiate between various classes of drug combinations, and may therefore provide an improved means for understanding their mechanisms of action toward clinical translation.

## 1 Introduction

Drug combinations that consist of multiple chemical agents have shown great promises to improve efficacy and overcome resistance for treating complex and refractory diseases. In cancer therapy, for example, an effective drug combination may target multiple proteins or pathways that are aberrantly activated in disease, but not in normal cells, and thus reduces the chances that a cancer will develop drug resistance by activating bypassing pathways, when compared to standard monotherapies [1], [2], [3]. To accelerate the discovery of novel drug combinations using an empirical approach, preclinical drug screening platforms have been developed that allow for assaying the phenotypic endpoint responses *in vitro* or *ex vivo*. With high-throughput techniques, it has become possible to systematically evaluate the pairwise combinations from a large collections of both approved and investigational chemical compounds [4]. Such functional screening approaches have increasingly led to advances in the characterization of drug–drug interactions for multiple diseases, and prioritization of effective and safe drug combinations for further clinical examination.

To quantify the interaction between drugs, the observed combination response is often compared to the expected effect under the assumption of non-interaction predicted by a reference model. When the combination response is greater than what is expected, then the combinations is classified as synergistic, while antagonism is concluded when the combination produces less than the expected effect. Currently, there are three popular classes of reference models: Highest single agent (HSA) model [5], Loewe additivity model [6] and Bliss independence model [7]. These reference models, together with many of their subsequent variants and extensions, have been developed based on different assumptions about the expected effect of non-interaction. The HSA model, or Gaddum's non-interaction model, assumes that the expected combination effect equals to the higher individual drug effect at the dose in the combination, representing the idea that a synergistic drug combination should produce additional benefits on top of what its components can achieve alone. In many preclinical drug combination studies, however, even a drug combined with itself can easily produce an excess over HSA. For more stringent synergy classification, the Loewe additivity and Bliss independence models are being widely used. The Loewe additivity model defines the expected effect as if a drug was combined with itself, while the Bliss independence model utilizes probabilistic theory to model the effects of individual drugs in a combination as independent yet competing events. Due to the inherent differences in the model assumptions, there is a lack of consensus on which references model one should use in an unbiased and statistically robust manner. As pointed out by many others [8], [9], [10], there is still no standardized guideline on how to choose the optimal reference model.

Apart from the disagreement among the theoretical aspects, there are considerable pragmatic challenges on how to apply the Loewe and Bliss models for analyzing drug combination experiments. First, the currently available scoring tools are rather limited and suboptimal for a high-throughput setting. For instance, CompuSyn is one of the most-cited standalone software packages used to calculate the interaction score based on the Loewe model [11]. However, it only allows for a manual input of dose–response data for one drug combination at a time, and thus becomes quickly unfeasible for analyzing large-scale drug screening data which typically consists of hundreds of combinations. For implementations of the Loewe model, there are a few packages in R, such as drc [12] and SYNERGY [13], but these packages are not flexible enough to handle the noise in a typical high-throughput drug screen, which tend to invalidate many of the widely used curve fitting functions. As a result, drugs that have negative or irregular responses may easily lead to a fitting error that breaks down the program. Secondly, many existing methods utilize a summary interaction score to characterize the overall drug interaction effect, which may be sufficient for the initial filtering of potential synergistic drug combinations. However, a follow-up confirmatory screen often utilizes a dose–response matrix for which a single interaction parameter cannot effectively capture the synergies and antagonisms that may occur within specific dose regions only. With an increasing number of drug combinations that have been tested using the dose–response matrix design, there is a critical need to develop efficient and robust computational tools to systematically evaluate and visualize the drug interaction at the level of individual dose combinations.

To address these challenges, we introduced a novel reference model, named zero interaction potency (ZIP), which overcomes many of the limitations of the existing models. By combining the advantages of both the Loewe and Bliss models, the ZIP model assumes that two non-interacting drugs are expected to incur minimal changes in their dose–response curves. We calculated a delta score to quantify the deviation from the expectation of ZIP for a given dose pair and utilized the average delta over a dose–response matrix as a summary interaction score for a drug combination. To test this model, we analyzed the data from a recent high-throughput drug combination study in cancer [14]. We showed that the ZIP-based delta scoring approach tolerates well the experimental noise in such combination screens and provides an improved solution for identifying true synergistic interactions, while keeping the false positive rate relatively low. Further, we demonstrated how an interaction landscape visualization of delta scores can facilitate the identification of clinically relevant drug synergy within specific dose regions. We believe our drug interaction scoring method has the potential to allow for a systematic evaluation of drug combinations in a high-throughput setting, and may therefore become increasingly beneficial for drug combination discovery and development in the future experiments.

## 2 Methods

### 2.1 Relationships Between the Existing Reference Models of Non-Interaction

Consider a typical drug screening experiment where the drug's effect *y* is expressed on a continuous scale between 0 and 1, i.e. 0 ≤ *y* ≤ 1. In a cell-based drug screening, *y* is usually measured as the fractional inhibition of cell growth, or percentage of cell death. Note that some studies alternatively consider the drug's effect as a percentage of survival or viability, which is opposite to our definition, but the subsequent mathematical derivation also applies by replacing *y* with 1 – *y*. Suppose that one drug produced an effect *y*_1_ at dose *x*_1_ and the other drug produced an effect *y*_2_ at dose *x*_2_ while combining them produced *y_c_*. We follow a common three-class nomenclature to distinguish the interactions between the two drugs. Namely a drug interaction can be classified as synergistic, antagonistic or non-interactive, depending on whether *y_c_* is greater or less than the expected effect under the assumption of non-interaction. To calculate the expected effect, one needs to utilize a reference model that is based on certain principles or assumptions. There are three frequently-used reference models: Highest single agent (HSA), Bliss independence and Loewe additivity.

The HSA model states that the expected combination effect equals to the higher effect of individual drugs, i.e. *y*_HSE_ = max(*y*_1_, *y*_2_). Therefore, any additional effect over the higher single drug will be considered as a HSA synergy [5].

The Bliss independence model [7] assumes a stochastic process in which two drugs elicit their effects independently, and the expected combination effect can be calculated based on the probability of independent events as

(1) $$y_{\mathrm{BLISS}}=y_{1}+y_{2}-y_{1}y_{2}\text{.}$$

**Fig. 1:**
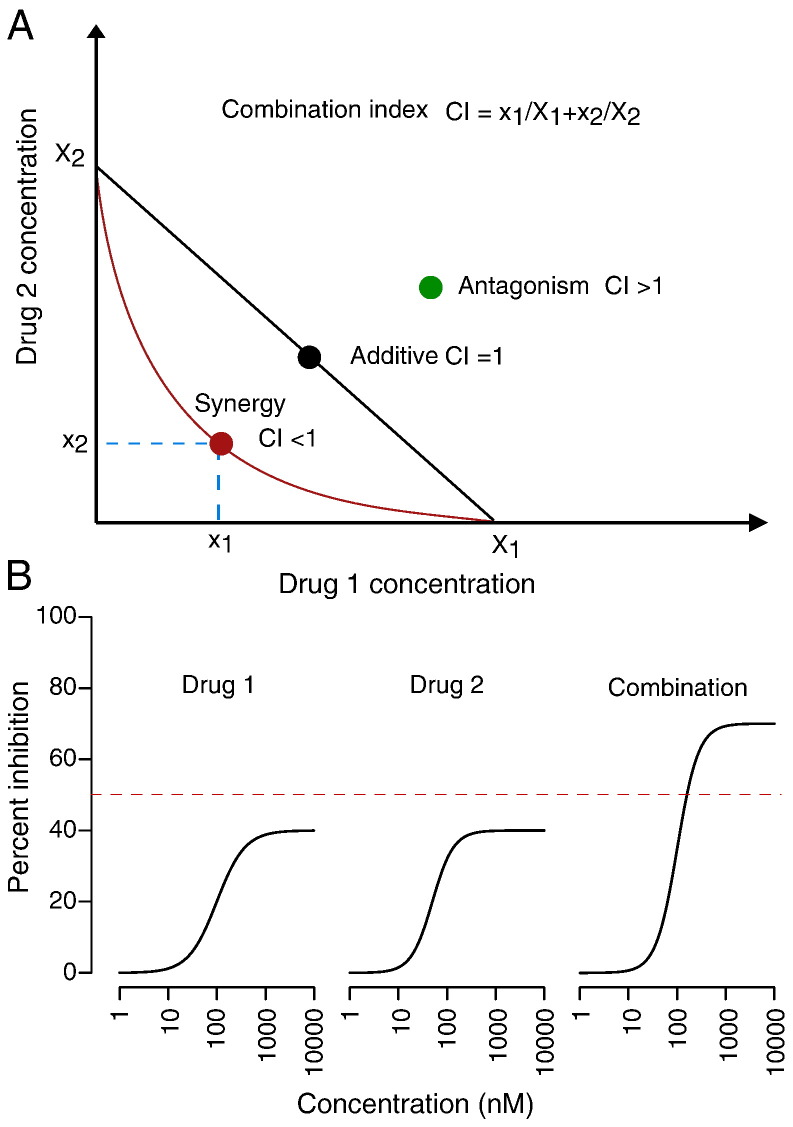
The concept of Combination Index (CI) based on the Loewe additivity model. (A) Loewe additivity with CI = 1 can be visualized as a straight line at a two-dimensional isobologram with the doses of drug 1 and drug 2 as coordinates. A synergistic (CI < 1) and antagonistic (CI > 1) drug combination will be positioned below and above the additivity line, respectively (adopted from [9]). (B) Loewe additivity model cannot directly assess such drug interaction in which a combination effect is higher than the achievable effect of the individual drugs, even though by intuition one would expect a clinically relevant synergy in such cases.

The Loewe additivity model states that the expected effect *y*_LOEWE_ must satisfy

(2) $$\frac{x_{1}}{X_{\mathrm{LOEWE}}^{1}}+\frac{x_{2}}{X_{\mathrm{LOEWE}}^{2}}=1\text{,}$$

where *X*^1^_LOEWE_ and *X*^2^_LOEWE_ are the doses of drug 1 and 2 alone that produce *y*_LOEWE_[6]. In a Cartesian coordinate system, with the axes representing the individual drug doses, Eq. (2) can be represented as a straight line of additivity connecting *X*^1^_LOEWE_ and *X*^2^_LOEWE_ (Fig. 1A). This is also called the Isobole of additivity [15]. Note that a hidden assumption has been made that the individual drug dose–responses must be monotonic, i.e., a drug alone produces *y*_LOEWE_ at a higher dose than in the combination. Suppose that the observed combination effect *y*_c_ > *y*_LOEWE_ then we have *X*_*c*_^1^ > *X*_LOEWE_^1^ and *X*_*c*_^2^ > *X*_LOEWE_^2^, where *X*_*c*_^1^ and *X*_*c*_^2^ are defined similarly as the doses of drug 1 and 2 alone that produce *y*_c_. This leads to

(3) $$\frac{x_{1}}{X_{c}^{1}}+\frac{x_{2}}{X_{c}^{2}}<1\text{.}$$

The left part of Eq. (3) is also called Combination Index (CI), with CI < 1 corresponding to *y*_c_ > *y*_LOEWE_ and thus a Loewe synergy.

The Loewe additivity model can also take a parametric form. One way to describe the dose–response curves is a commonly-used 4-parameter log-logistic (4PL) function

(4) $$y=\frac{E_{\text{min}}+E_{\text{max}}{\left(\frac{x}{m}\right)}^{\lambda }}{1+{\left(\frac{x}{m}\right)}^{\lambda }}\text{.}$$

Here, *E*_min_ and *E*_max_ are the minimal and maximal effects of the drug (0 ≤ *E*_min_ < *E*_max_ ≤ 1); *m* is the dose that produces the midpoint effect of *E*_min_ + *E*_max_, also known as relative EC_50_ or IC_50_, and *λ*(*λ* > 0) is the shape parameter indicating the sigmoidicity or slope of the curve. In addition to much mathematical and statistical convenience, the 4PL function leads to an odds ratio of the affected *f_a_* and unaffected *f_u_* fractions as a logit model

(5) $$\frac{f_{a}}{f_{u}}=\frac{y-E_{\text{min}}}{E_{\text{max}}-y}={\left(\frac{x}{m}\right)}^{\lambda }\text{.}$$

Eq. (5) corresponds to the widely used Chou and Talalay median effect equation, which fits to the expectation of the mass-action law principle that dictates many biological processes such as cell growth or ligand-binding interactions [9].

Following this line of parameterization, we can calculate the dose that produces a given effect as

(6) $$x=m{\left(\frac{y-E_{\text{min}}}{E_{\text{max}}-y}\right)}^{1/\lambda }\text{.}$$

Assuming *λ* = 1, *E*_min_ = 0 and *E*_max_ = 1, Eq. (6) leads to a constant dose ratio for the two drugs that alone produce the same effect, i.e., *x*_1_ / *x*_2_ = *m*_1_ / *m*_2_, which is called the constant relative potency model that has been explored before [15].

From Eq. (6), one can derive the analytical form of Eq. (2) for *y*_LOEWE_ as

(7) $$\frac{x_{1}}{m_{1}{\left(\frac{y_{\mathrm{LOEWE}}-E_{\text{min}}^{1}}{E_{\text{max}}^{1}-y_{\mathrm{LOEWE}}}\right)}^{1/\lambda_{1}}}+\frac{x_{2}}{m_{2}{\left(\frac{y_{\mathrm{LOEWE}}-E_{\text{min}}^{2}}{E_{\text{max}}^{2}-y_{\mathrm{LOEWE}}}\right)}^{1/\lambda_{2}}}=1\text{,}$$

for which a numerical nonlinear solver can be used to determine *y*_LOEWE_ for (*x*_1_, *x*_2_).

The Combination Index can be also derived in an analytical form as

(8) $$CI=\frac{x_{1}}{m_{1}{\left(\frac{y_{c}-E_{\text{min}}^{1}}{E_{\text{max}}^{1}-y_{\mathrm{LOEWE}}}\right)}^{1/\lambda_{1}}}+\frac{x_{2}}{m_{2}{\left(\frac{y_{c}-E_{\text{min}}^{2}}{E_{\text{max}}^{2}-y_{\mathrm{LOEWE}}}\right)}^{1/\lambda_{2}}}\text{.}$$

Further extending Eq. (8), [8] proposed an interaction index *a* defined in the following equation:

(9) $$1=\frac{x_{1}}{m_{1}{\left(\frac{y_{c}-E_{\text{min}}^{1}}{E_{\text{max}}^{1}-y_{c}}\right)}^{1/\lambda_{1}}}+\frac{x_{2}}{m_{2}{\left(\frac{y_{c}-E_{\text{min}}^{2}}{E_{\text{max}}^{2}-y_{c}}\right)}^{1/\lambda_{2}}}+\alpha \frac{x_{1}x_{2}}{m_{1}m_{2}{\left(\frac{y_{c}-E_{\text{min}}^{1}}{E_{\text{max}}^{1}-y_{c}}\right)}^{1/2\lambda_{1}}{\left(\frac{y_{c}-E_{\text{min}}^{2}}{E_{\text{max}}^{2}-y_{c}}\right)}^{1/2\lambda_{2}}}\text{,}$$

with case *a* = 0 corresponding to *CI* = 1 and thus equivalent to the Loewe additivity.

Consider a sham experiment, where two identical drugs are combined, i.e., *m*_1_ = *m*_2_ and *λ*_1_ = *λ*_2_, then Eq. (7) can be simplified as

(10) $$\frac{x_{1}+x_{2}}{m{\left(\frac{y_{\mathrm{LOEWE}}-E_{\text{min}}}{E_{\text{max}}-y_{\mathrm{LOEWE}}}\right)}^{1/\lambda }}=1\text{,}$$

from which one can derive that

(11) $$y_{\mathrm{LOEWE}}=\frac{E_{\text{min}}+E_{\text{max}}{\left(\frac{x_{1}+x_{2}}{m}\right)}^{\lambda }}{1+{\left(\frac{x_{1}+x_{2}}{m}\right)}^{\lambda }}\text{.}$$

This shows that *y*_LOEWE_ is equal to the single drug response at dose *x*_1_ + *x*_2_ in a sham experiment, which has been an important justification of the Loewe additivity model. However, for actual drug combination studies where two drugs are unlikely identical, such an additivity implication according to Eq. (11) might become less intuitive, and sometimes it can be even problematic. For example, *y*_LOEWE_ is expected to be lower than the achievable effects of both individual drugs according to Eq. (7), i.e., *y*_LOEWE_ < min(*E*_max_^(1)^, *E*_max_^(2)^). If we observe a combination effect which is not observed in one of the individual drugs, we do not have a definite answer for *y*_LOEWE_. In fact, this limitation of the Loewe additivity model is irrespective of what the parameterization might be. Consider a simple case where *y*_1_ = 0.3, *y*_2_ = 0.4 and *y*_c_ = 0.6, and we know that *E*_max_^1^ = 0.4 and *E*_max_^2^ = 0.5. We ask the following question: what is the expected effect of non-interaction and how much is the synergy for *y*_c_? Both HSA and Bliss can provide a sound answer but with the Loewe additivity model the solution is not straightforward. Following the sham experimental principle, one might conclude either that *y*_LOEWE_ = 0.4 or *y*_LOEWE_ = 0.5 depending on which individual drugs one focuses on.

The upper limit of *y*_LOEWE_ does not seem to be sufficiently discussed in the literature. One reason might be that traditionally one would start testing a drug combination only if the individual drugs were already known to be effective. This would lead to the increase of *E*_max_^1^ and *E*_max_^2^ close to 1, which allows that *y*_LOEWE_ can be calculated for the majority of the tested dose pairs. In a high-throughput setting, however, we often do not know beforehand whether the drugs in a combination are effective or not within the tested dose ranges. If we see a combination that produces a stronger effect than what an individual drug can achieve within the dose range alone, by intuition, we would consider it as a significant synergy. For this scenario, unfortunately, the Loewe additive model cannot be utilized directly due to the lower boundaries for individual drugs (Fig. 1B).

### 2.2 Zero Interaction Potency Model for Evaluating Drug Interactions

Given the effect *y*_1_ at dose *x*_1_ for drug 1 and *y*_2_ at dose *x*_2_ for drug 2, we rephrased the question: what is the expected combination effect for a dose pair (*x*_1_, *x*_2_)? Both the HSA and the Bliss independence models give a point estimate using different assumptions while the Loewe additivity model considered the dose–response curves of individual drugs. We took one step further by considering not only the dose–response curves for individual drugs but also for their combinations and derive a score to quantify their interactions. We considered a 4-parameter log-logistic function as defined in Eq. (4) to model the dose–response curves. The logistic function has been commonly utilized in the characterization of complex dose–response relationship as it offers flexibility even for fitting a flat dose–response. For the sake of simplicity in the remaining derivations, we assumed further that the individual drugs are equally effective to reach the complete inhibition of the cell growth, i.e., *E*_min_ = 0 and *E*_max_ = 1. The derivation of ZIP using the full 4-parameter logistic function is provided in Appendix A3. We took the perspective of zero potency shift for non-interaction, i.e., an expected combination effect should not change the potency of the individual drug dose–response curves. Take drug 1 as an example, the single dose–response relationships follows:

(12) $$y=\frac{{\left(\frac{x}{m_{1}}\right)}^{\lambda_{1}}}{1+{\left(\frac{x}{m_{1}}\right)}^{\lambda_{1}}}\text{.}$$

When drug 2 at dose *x*_2_ is added to drug 1, it will change the dose–response curve accordingly. If there is no interaction, then adding drug 2 should simply increase the baseline level of drug 1, while incurring no potency shift in the dose–response curve. This would imply that *m*_1_ and *λ*_1_ remain unchanged. The dose–response curve for drug 1 in the combination then becomes

(13) $$y_{1\leftarrow 2}=\frac{y_{2}+{\left(\frac{x}{m_{1}}\right)}^{\lambda_{1}}}{1+{\left(\frac{x}{m_{1}}\right)}^{\lambda_{1}}}\text{.}$$

Here, we used _1 ← 2_ to emphasize the drug combination as drug 1-centric, i.e., adding drug 2 to drug 1 to examine the change in dose–response curve of drug 1. The expected combination effect at dose pair (*x*_1_, *x*_2_) can be derived from Eq. (13) as

(14) $$y_{\mathrm{ZIP}}^{1\leftarrow 2}=\frac{y_{2}+{\left(\frac{x_{1}}{m_{1}}\right)}^{\lambda_{1}}}{1+{\left(\frac{x_{1}}{m_{1}}\right)}^{\lambda_{1}}}=\frac{\frac{1}{1+{\left(\frac{m_{2}}{x_{2}}\right)}^{\lambda_{2}}}+{\left(\frac{x_{1}}{m_{1}}\right)}^{\lambda_{1}}}{1+{\left(\frac{x_{1}}{m_{1}}\right)}^{\lambda_{1}}}\text{.}$$

Similarly, the expected combination effect from the drug-2 centric viewpoint by adding drug 1 to drug 2 becomes

(15) $$y_{\mathrm{ZIP}}^{2\leftarrow 1}=\frac{y_{1}+{\left(\frac{x_{2}}{m_{2}}\right)}^{\lambda_{2}}}{1+{\left(\frac{x_{2}}{m_{2}}\right)}^{\lambda_{2}}}=\frac{\frac{1}{1+{\left(\frac{m_{1}}{x_{1}}\right)}^{\lambda_{1}}}+{\left(\frac{x_{2}}{m_{2}}\right)}^{\lambda_{2}}}{1+{\left(\frac{x_{2}}{m_{2}}\right)}^{\lambda_{2}}}\text{.}$$

A simple algebra shows that *y*_ZIP_^1 ← 2^ = *y*_ZIP_^2 ← 1^, which reflects the intuition that the expected combination effect should be independent of the order of adding individual drugs in the combination. Appendix A1 shows how *y*_ZIP_ can be factorized as a multiplicative of single dose–response curves, which implies the probabilistic independence between the two drugs:

(16) $$y_{\mathrm{ZIP}}=\frac{{\left(\frac{x_{1}}{m_{1}}\right)}^{\lambda_{1}}}{1+{\left(\frac{x_{1}}{m_{1}}\right)}^{\lambda_{1}}}+\frac{{\left(\frac{x_{2}}{m_{2}}\right)}^{\lambda_{2}}}{1+{\left(\frac{x_{2}}{m_{2}}\right)}^{\lambda_{2}}}-\frac{{\left(\frac{x_{1}}{m_{1}}\right)}^{\lambda_{1}}}{1+{\left(\frac{x_{1}}{m_{1}}\right)}^{\lambda_{1}}}\frac{{\left(\frac{x_{2}}{m_{2}}\right)}^{\lambda_{2}}}{1+{\left(\frac{x_{2}}{m_{2}}\right)}^{\lambda_{2}}}\text{.}$$

### 2.3 Delta Score to Quantify the Deviation from the ZIP Model

**Fig. 2:**
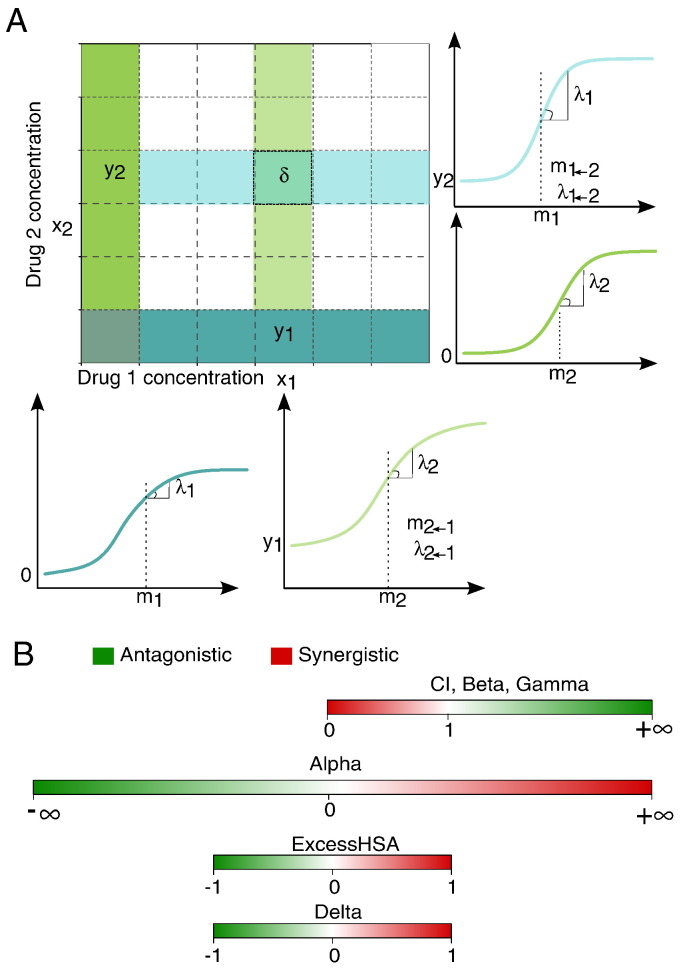
(A) Formulation of the ZIP model and the delta scoring illustrated in a dose–response matrix. To evaluate the degree of interaction at a dose combination (*x*_1_, *x*_2_), the midpoint *m* and the shape parameter *λ* from the individual drug responses (the first column and the last row) as well as their combined effects at column *x*_1_ and row *x*_2_ are compared. The delta scoring considers the changes of *m* and *λ* for the dose–response curves between drug 1 alone (the bottom row) and the combination after adding *x*_2_ (row *x*_2_), as well as between drug 2 alone (the first column) and the combination after adding *x*_1_ (column *x*_1_). (B) Scale and interpretation of the drug interaction scores. Each scoring method determines a synergistic drug combination differently. The delta scoring quantifies the synergistic effects as the percentage inhibition values and thus a non-interaction will correspond to delta value of 0. Alpha and HSA also have a score of 0 for non-interaction, whereas for CI, beta and gamma scores, the reference score for non-interaction stands at 1. CI, beta and gamma scores are left-bounded at 0. The directions of the interaction scores are also different. For CI, beta and gamma, a lower score is more synergistic while for delta, alpha and HSA it is the opposite interpretation.

The ZIP model was derived from the zero interaction potency perspective, but from Eq. (16) one can see that when modeling the dose–response with logistic functions, the zero interaction from the ZIP model corresponds to probabilistic independence. Following this line, we further reasoned that a degree of drug interaction can be modeled as the potency shifts captured by the dose–response curve parameters: If one drug alters the potency of the other drug, then comparing the dose–response curves of individual drugs and their drug combination should give a quantitative measure of the interaction effect. Namely, we fitted the observed combination effect *y_c_* in a two-way manner similarly to (14), (15):

(17) $$y_{c}^{1\leftarrow 2}=\frac{y_{2}+{\left(\frac{x_{1}}{m_{1\leftarrow 2}}\right)}^{\lambda_{1\leftarrow 2}}}{1+{\left(\frac{x_{1}}{m_{1\leftarrow 2}}\right)}^{\lambda_{1\leftarrow 2}}}=\frac{\frac{1}{1+{\left(\frac{m_{2}}{x_{2}}\right)}^{\lambda_{2}}}+{\left(\frac{x_{1}}{m_{1\leftarrow 2}}\right)}^{\lambda_{1\leftarrow 2}}}{1+{\left(\frac{x_{1}}{m_{1\leftarrow 2}}\right)}^{\lambda_{1\leftarrow 2}}}\text{,}$$

(18) $$y_{c}^{2\leftarrow 1}=\frac{y_{1}+{\left(\frac{x_{2}}{m_{2\leftarrow 1}}\right)}^{\lambda_{2\leftarrow 1}}}{1+{\left(\frac{x_{2}}{m_{2\leftarrow 1}}\right)}^{\lambda_{2\leftarrow 1}}}=\frac{\frac{1}{1+{\left(\frac{m_{1}}{x_{1}}\right)}^{\lambda_{1}}}+{\left(\frac{x_{2}}{m_{2\leftarrow 1}}\right)}^{\lambda_{2\leftarrow 1}}}{1+{\left(\frac{x_{2}}{m_{2\leftarrow 1}}\right)}^{\lambda_{2\leftarrow 1}}}\text{,}$$

where *m*_1 → 2_ and *λ*_1 → 2_ are the projected potency and shape parameters for drug 1 when adding *x*_2_; *m*_2 → 1_ and *λ*_2 → 1_ are those parameters derived for drug 2 when adding *x*_1_ (see Fig. 2A for illustrations). We defined a delta score to capture the overall interaction potency shift by taking an average deviation between *y_c_* and *y*_ZIP_ from Eqs. (14), (15), (17), (18):

(19) $$\begin{array}{l} \delta \left(\theta \right)=\frac{y_{c}^{1\to 2}-y_{\mathrm{ZIP}}^{1\to 2}}{2}+\frac{y_{c}^{2\to 1}-y_{\mathrm{ZIP}}^{2\to 1}}{2}\\ =\frac{1}{2}\left(\frac{\frac{1}{1+{\left(\frac{m_{2}}{x_{2}}\right)}^{\lambda_{2}}}+{\left(\frac{x_{1}}{m_{2\to 1}}\right)}^{\lambda_{2\to 1}}}{1+{\left(\frac{x_{1}}{m_{2\to 1}}\right)}^{\lambda_{2\to 1}}}+\frac{\frac{1}{1+{\left(\frac{m_{1}}{x_{1}}\right)}^{\lambda_{1}}}+{\left(\frac{x_{2}}{m_{1\to 2}}\right)}^{\lambda_{1\to 2}}}{1+{\left(\frac{x_{2}}{m_{1\to 2}}\right)}^{\lambda_{1\to 2}}}\right)-\left(\frac{{\left(\frac{x_{1}}{m_{1}}\right)}^{\lambda_{1}}}{1+{\left(\frac{x_{1}}{m_{1}}\right)}^{\lambda_{1}}}+\frac{{\left(\frac{x_{2}}{m_{2}}\right)}^{\lambda_{2}}}{1+{\left(\frac{x_{2}}{m_{2}}\right)}^{\lambda_{2}}}-\frac{{\left(\frac{x_{1}}{m_{1}}\right)}^{\lambda_{1}}}{1+{\left(\frac{x_{1}}{m_{1}}\right)}^{\lambda_{1}}}\frac{{\left(\frac{x_{2}}{m_{2}}\right)}^{\lambda_{2}}}{1+{\left(\frac{x_{2}}{m_{2}}\right)}^{\lambda_{2}}}\right)\text{,} \end{array}$$

where the parameter set θ = {*m*_1_, *m*_2_, *m*_1 →2_, *m*_2 →1_, *λ*_1_, *λ*_2_, *λ*_1 →2_, *λ*_2 →1_} can be estimated from the dose–response data, typically using the least-squares method which is equivalent to a maximum likelihood estimate for normally distributed errors [13]. Score of *δ* = 0, > 0 or < 0 corresponds to the zero interaction, synergy and antagonism, respectively. We further showed that *δ* = 0 also holds for a sham experiment where two identical drugs are combined, i.e., *m*_1_ = *m*_2_ and *λ*_1_ = *λ*_2_ (proof details in Appendix A2). From this perspective, we may consider the ZIP model an integration of the Bliss independence and the Loewe additivity models.

### 2.4 Interaction Landscape Surface Plot Based on the Delta Score

As seen from Eq. (19), the delta scoring requires the parameters for the dose–response curves both in monotherapy and in combination. The estimation of these parameters requires at least three dose–response data points, i.e., (*y*_1_, *y*_2_, *y*_c_) at (*x*_1_, *x*_2_, (*x*_1_, *x*_2_,)). However, for a reliable estimate, one would need more comprehensive response data where multiple doses have been tested. High-throughput screening has made it possible to efficiently probe a drug pair at multiple doses in a full matrix. Therefore, a delta score can be calculated for each dose combination in the matrix, which allows for a surface plot of delta scores. Such a surface plot enables one to characterize drug interaction effects over the full dose matrix, which is more informative than what a single summary score can provide. We utilized the surface plot of the delta scores to visualize the interaction landscape for a drug combination, aiming to identify synergistic and antagonistic dose regions for further dose optimization in a validation screen. It is worth noting that a delta score has a unit of percentage inhibition (e.g., *δ* = 0.2 corresponds to 20% of response beyond expectation). Therefore, the delta scores are directly comparable within and between drug combinations. This is also an important feature of our approach that facilitates efficient prioritization in a high-throughput setting.

### 2.5 The Mathews Griner Drug Combination Screening Dataset

To demonstrate the performance of the ZIP-based delta scoring, we considered a recent cancer drug screen study involving ibrutinib in combination with 466 compounds for the activated B-cell-like subtype (ABC) of diffuse large B-cell lymphoma (DLBCL) [14]. Ibrutinib is a small molecule targeting Bruton's tyrosine kinase (BTK) approved for the treatment of mantle cell lymphoma and chronic lymphocytic leukemia [16]. In this study, a high-throughput drug combination screening was used to identify other compounds that can synergistically interact with ibrutinib to improve its anticancer efficacy and circumvent drug resistance. For each drug pair, a 6 × 6 dose–response matrix design was utilized, where the drug effect was measured as percentage of cell viability using TMD8 cancer cell line. The raw combination data was provided by the authors via personal communication, but can now be downloaded from https://tripod.nih.gov/matrix-client/rest/matrix/export/241. We transformed the original percentage viability data into the percentage inhibition data before applying the drug combination analysis to be compatible with the mathematical formulation defined in the Methods section.

We ran the ZIP model on the drug combination data and calculated a summary delta score Δ for each drug pair by taking the average of all the delta scores over its dose combinations, i.e., $\Delta =\frac{1}{n}\sum_{i=1}^{n}\delta \text{,}$ where *n* is the number of dose combinations and *n* = 25 for a 6 × 6 dose–response matrix (monotherapy responses were removed). We compared the summary delta scores with the other scores derived from the HSA-, Bliss- and Loewe-based models. For HSA and Bliss, there were existing scores implemented in the original study [14], which were based on the following methods: 1) NumExcess is the number of wells in the dose matrix that produced higher effect than both of the individual drug effects; 2) ExcessHSA is the sum of differences between the combination effect and the expected HSA effect; 3) MedianExcess is the median of the HSA excess; 4) ExcessCRX is an extension of the HSA model that was adjusted by dilution factors; 5) LS3 × 3 is the ExcessHSA applied to a 3 × 3 block showing the best HSA synergy in the dose matrix; 6) Beta (*β*) is the interaction parameter minimizing the deviance from the Bliss independence model over all dose combinations defined as $\underset{\beta }{\arg\min}\left(\sum {\left(1-y_{c}-\beta \left(1-y_{1}\right)\left(1-y_{2}\right)\right)}^{2}\right)$; and 7) Gamma (*γ*) is a combination of HSA and Bliss models minimizing $\underset{\gamma }{\arg\min}\left(\sum {\left(1-y_{c}-\gamma \max\left(1-y_{1},1-y_{2}\right)\right)}^{2}\right)\text{.}$ For the Loewe-based models, we calculated the two common interaction indices CI (Eq. (8)) and alpha(*a*) (Eq. (9)). The CI was calculated using an R package SYNERGY [13] and the alpha score was estimated using the R package *drc* [12].

### 2.6 Statistical Significance Testing for an Observed Delta Score

For a drug combination where multiple replicates are available, we may take a two-step approach for the evaluation of statistical significance of the observed delta score at a given dose pair (*x*_1_, *x*_2_).First, we may utilize non-linear regression fitted with logistic functions to estimate the parameters in *θ*.Then, we can utilize a bootstrapping method by randomly sampling the parameter set *θ* from normal distributions with their estimated means and variances, with which we calculated delta using Eq. (19). This simulation can be repeated multiple times to get a stable distribution of delta from which an asymptotic *p*-value can be calculated as the proportion of the bootstrap samples with delta higher than 0. Further, as long as the random sampling of delta follows approximately a normal distribution, 100(1 − *a*)% confidence interval for delta can be calculated as

$$\left(\overset{\wedge }{\delta }-z_{1-\alpha /2}\sqrt{\mathrm{var}\left(\delta \right)/n},\overset{\wedge }{\delta }+z_{1-\alpha /2}\sqrt{\mathrm{var}\left(\delta \right)/n}\right)\text{,}$$

where *z*_1 − *a*/2_ is the 100(1 − *a* / 2) percentile of the standard normal distribution and *α* is the type I error that normally is set at 0.05 [17].

**Supplementary Fig. 1:**
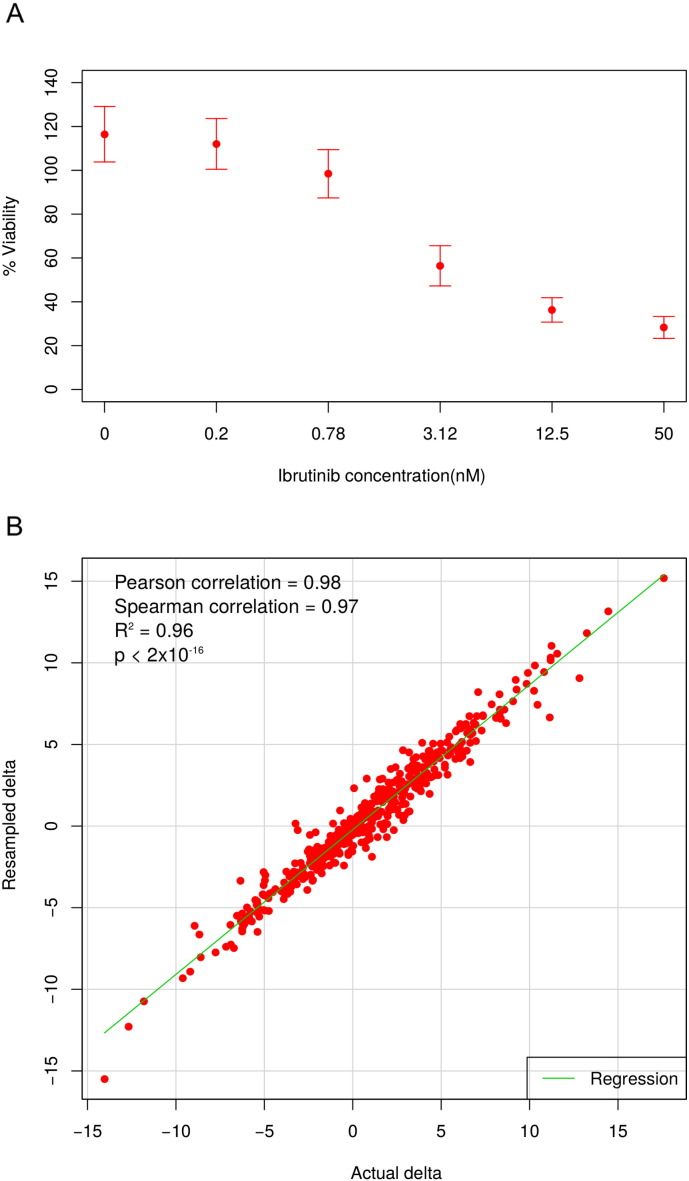
(A) The means and standard deviations of the cell viability in response to six concentrations of ibrutinib. (B) The consistency between the delta scores calculated using the actual data and the simulated data.

Since each of the 466 drug combinations in the Mathews Griner data was tested only once, the statistical procedure above cannot be implemented as such in this dataset. However, because all of the drug combinations involved ibrutinib, we can collect its replicated response data at each of the six tested concentrations. The variance of the ibrutinib monotherapy data can be further utilized to quantify the experimental noise and extrapolated for the drug combination measurements (Supplementary Fig. 1A). By assuming that the measurement uncertainty depends on a specific concentration of ibrutinib, we generated random samples for each drug combination according to normal distributions *N*(*y*_*ij*_, *σ*_*i*_^2^), where *y_ij_* is the observed drug combination response where ibrutinib *i*th concentration is combined with the other drug at the *j*th concentration; *σ*_*i*_^2^ is the variance of the ibrutinib response at the *i*th concentration. We simulated 1,000 samples for each drug combination, and calculated a resampled delta score, for which the statistical significance can be approximated.

## 3 Results

### 3.1 The Symmetry, Range and Robustness of the Delta Score

We proposed the delta score based on the ZIP model for evaluating drug interaction effect at a given dose pair. For a dose–response matrix, we utilized a summary delta score to average the overall interaction effect over all the dose pairs for a drug combination. For a comparative analysis, we explored altogether ten drug interaction scoring methods, i.e., NumExcess, ExcessHSA, MedianExcess, ExcessCRX, LS3 × 3, Beta, Gamma, CI, Alpha and Delta. These scoring methods can be represented as an axis with a reference point for non-interaction (Fig. 2B). For beta, gamma and CI, the reference score for non-interaction is 1 and a value less than 1 indicates synergy. These scoring methods are thus non-symmetric since the range of synergy is between 0 and 1, which is much smaller than the range of antagonism which is [1,∞]. On the other hand, delta, alpha and HSA-based scores have the reference point at 0 for non-interaction, which fits more to the intuition that synergy and antagonism should have opposite signs. Unlike CI and alpha which are dimensionless, the delta score tells the percentage of cell inhibition and thus can provide directly an estimate of the extra effect of a drug interaction. For example, a delta of 0.1 would indicate that the drug combination will produce on average 10% more of the cell inhibition compared to the expected effect, while a delta of − 0.1 would indicate an antagonism with the same level of magnitude. Such a symmetry cannot be assumed for Loewe additive models (e.g., *y*_*CI* = 1.1_−*y*_LOEWE_ ≠ *y*_LOEWE_−*y*_*CI* = 0.9_, where *y*_*CI* = 1.1_ and *y*_*CI* = 0.9_ are the combination effects for *CI* = 1.1 and *CI* = 0.9, respectively). When comparing delta with HSA, it is obvious that delta is more stringent to classify synergy, and therefore it fits better with the observations that true synergies are rare [18].

The ZIP model was successfully applied to the Mathews Griner data consisting of 466 drug combinations, producing a summary delta score for each dose–response matrix (Supplementary Table 1). Since these drug combinations were not replicated, we carried out an ad-hoc simulation procedure to derive asymptotic delta scores for each combination by assuming that the measurement error follows a normal distribution with zero mean and variance estimated from the ibrutinib single-compound dose–responses (Supplementary Fig. 1A). We found that the summary delta scores obtained from the real data are highly similar to those from the simulated data (rank correlation = 0.97, root mean square error = 1.02%, Supplementary Fig. 1B), indicating a high degree of reproducibility in response to measurement errors. Further, the simulated scores showed a low level of dispersion (average median absolute deviation = 3.07%), implying that a robust estimation can be made when sufficient number of replicate measurements are available.

### 3.2 Correlations With the Other Interaction Scores and Their Classification Accuracy

**Supplementary Fig. 2:**
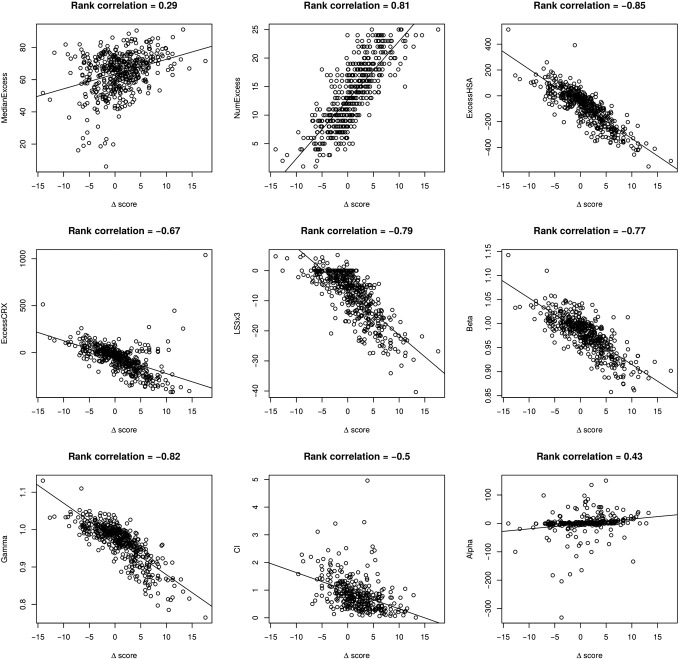
The agreement between the delta scoring and the other scoring methods. The delta scoring was highly correlated with the HSA, beta and gamma scores, but not with CI and alpha scores.

There is a general agreement between delta and most of the other scorings reported in the original publication: the highest correlations were found for ExcessHSA (rank correlation of − 0.85), beta (rank correlation of − 0.77) and gamma (rank correlation of − 0.83) (Supplementary Fig. 2). However, we found a large portion of drug combinations (*n* = 135), where the CI cannot be calculated due to the limitation of the Loewe-based models described in the Method section. After the removal of such cases, the correlation between the summary delta and CI was relatively poor (rank correlation of − 0.5). We note that the calculation of CI in the SYNERGY R package was done at a fixed-ratio level and therefore it utilized only the diagonal of a dose–response matrix. Ignoring the rest of the dose–response data makes the CI calculation more sensitive to outliers and therefore become less comparable with the other scores which utilize the whole dose–response matrix data. Similarly, the alpha score did not correlate well with delta (rank correlation of 0.43), and we also found difficulties to run the *drc* package on some of the combination data (*n* = 115 cases could not be calculated).

**Fig. 3:**
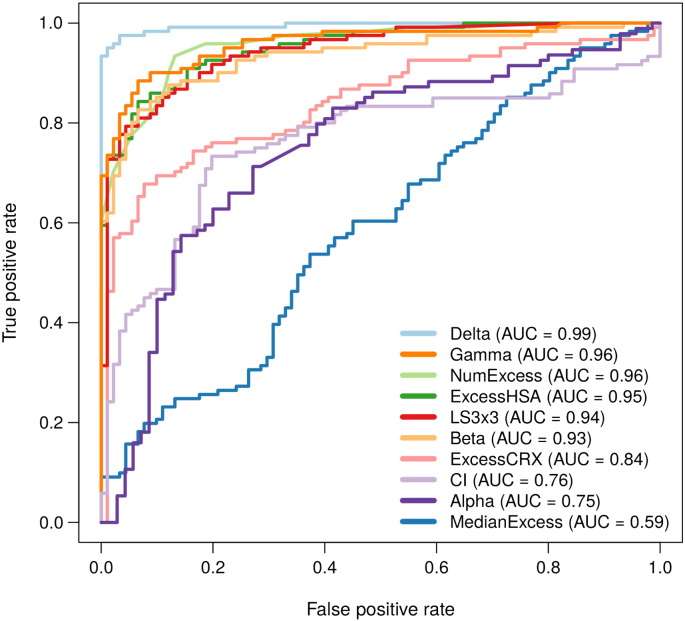
Classification accuracy of the drug interaction scoring methods. The ROC (receiver operator characteristic) curves were plotted using a visual classification of the raw drug combination data, which was blinded to the quantitative scoring methods. The area under the ROC curve (AUROC) is shown for each scoring method when classifying 112 synergistic and 91 antagonistic drug combinations. The statistical significance between the observed AUROCs can be found in Supplementary Table 2. Classification accuracy of the drug interaction scoring methods. The ROC (receiver operator characteristic) curves were plotted using a visual classification of the raw drug combination data, which was blinded to the quantitative scoring methods. The area under the ROC curve (AUROC) is shown for each scoring method when classifying 112 synergistic and 91 antagonistic drug combinations. The statistical significance between the observed AUROCs can be found in Supplementary Table 2.

Taken together, these comparative analyses suggested that delta scoring was more consistent with the HSA- and Bliss-based models than with the Loewe-based models. We also found that curve fitting pose frequent problems for the Loewe-based models, indicating that a Loewe model might not be flexible enough to cope with the high-throughput data, where a dose–response curve cannot be always fitted with logistic functions. To further evaluate the accuracy of these scoring models, we classified the 466 combinations into three interaction classes (synergistic, antagonistic and non-interaction), according to a visual inspection of the dose–response matrix raw data. Altogether, 121 combinations were labeled as synergistic, including 10 out of 11 synergistic combinations confirmed in [14], and 91 antagonistic combinations. We then performed the ROC analysis for the model scorings (Fig. 3, Supplementary Table 2), which showed that the delta scoring performed the best in this dataset, followed by the beta, gamma and ExcessHSA scorings. Consistent with the correlation results, the CI and alpha scorings produced lower classification accuracies.

**Fig. 4:**
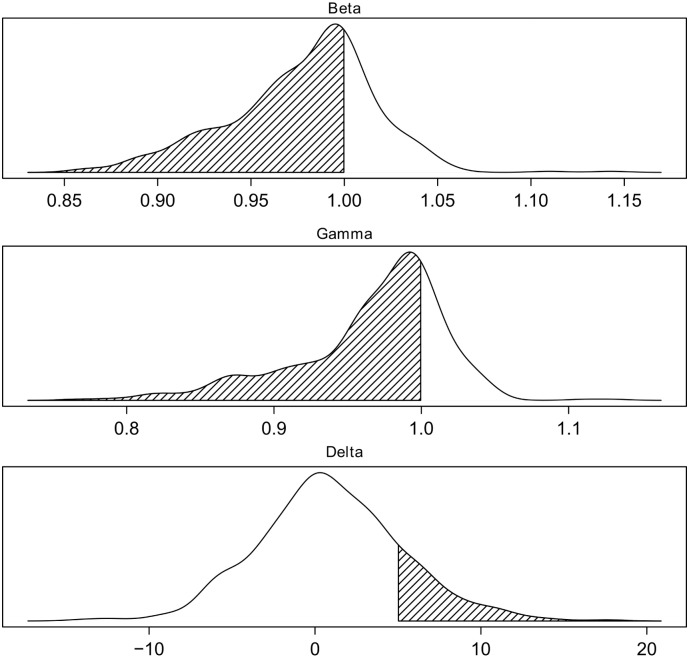
Density plots for beta, gamma and delta scores across the full set of 466 drug combinations in the Mathews Griner data. Beta and gamma scores tend to overestimate the number of synergistic combinations (shaded areas), while delta minimizes the rate of false positives by applying a threshold of 5% response, which is the typical noise level in a large-scale drug combination experiment.

Since there were significant portions of cases where we could not get valid CI and alpha scores, we decided to focus next on the comparisons between ZIP, HSA and Bliss models. The summary delta scores derived from the 466 drug combinations had a median at 0.007, corresponding to 0.7% of extra inhibition effect, which is quite close to its theoretic reference point at 0 (Table 1). The distribution also fits to our expectation that drug combinations in a high-throughput setting should be mostly non-interactive. For the other models, the interaction score was more shifted away from the theoretical reference point toward the synergistic regions. For example, the median and skewness of ExcessHSA scores are − 63.9 and − 0.27, respectively, indicating that more drug combinations were classified as synergistic in the HSA models. In particular, if we used a delta cut-off 0.05 (corresponding to an average excess of 5% inhibition in the combination) to classify the drug interaction, there were 74 synergistic combinations, which is much lower than the number of synergistic combinations determined by the other scores (Fig. 4). For example, using the beta score, one would identify 338 synergistic combinations with *β* < 1 and with *γ* < 1 this number becomes even higher (*n* = 354). As the main purpose of the high-throughput drug combination screening is to prioritize potential synergistic drug combinations for a secondary confirmation screen, these results suggest the delta scoring as a reliable approach to keep the prioritization process more cost-efficient and less error-prone.

### 3.3 Identification of Clinically Relevant Synergy and Antagonism via the Interaction Landscapes

**Fig. 5:**
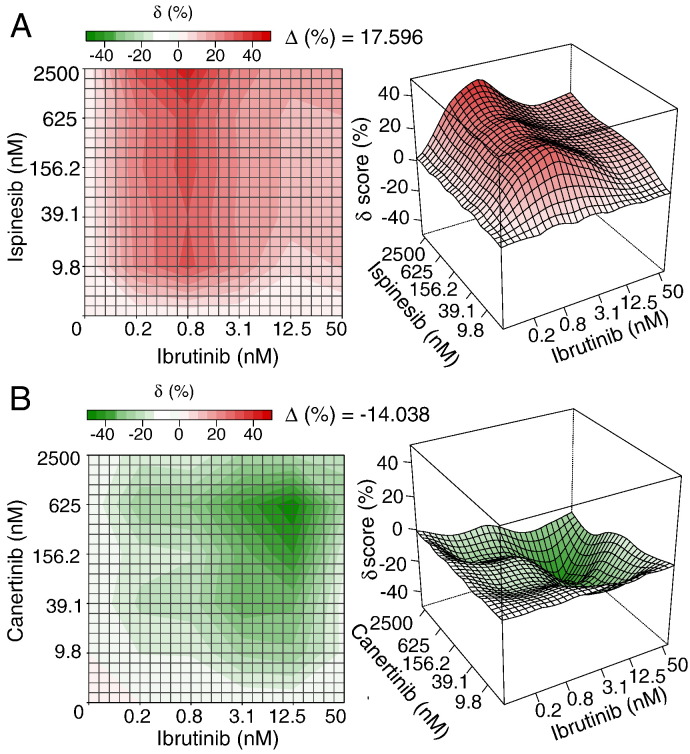
The top synergistic and antagonistic drug combinations identified from the Mathews Griner data. (A) The ispinesib and ibrutinib combination. (B) The canertinib and ibrutinib combination. For each combination, the interaction landscapes are shown in both 2D and 3D.*δ*: the excess % inhibition beyond the expectation by the ZIP model; Δ: the average *δ* scores over the dose–response matrix. The complete interaction landscapes for all the 466 drug combinations can be found in Supplementary Fig. 3. The top synergistic and antagonistic drug combinations identified from the Mathews Griner data. (A) The ispinesib and ibrutinib combination. (B) The canertinib and ibrutinib combination. For each combination, the interaction landscapes are shown in both 2D and 3D.*δ*: the excess % inhibition beyond the expectation by the ZIP model; Δ: the average *δ* scores over the dose–response matrix. The complete interaction landscapes for all the 466 drug combinations can be found in Supplementary Fig. 3.

We next utilized the delta scores to visualize in more detail the synergistic and antagonistic patterns on the dose–response matrix (Fig. 5, Supplementary Fig. 3). The top synergistic interaction was found with ispinesib, which is a selective Kinesin spindle protein (KSP) inhibitor that has entered clinical trials for many cancers [19]. The interaction between ispinesib and ibrutinib was almost universally synergistic over the whole dose–response matrix, leading to a maximal combination effect close to 100% of cell inhibition at the higher concentrations of both individual drugs. The strongest synergistic effect (40% inhibition) was found within the region of dose combinations where iburutinib's dose is fixed at 0.78 nM (Fig. 5A). Such a landscape pattern indicated that the dose of ispinesib can be lowered by 16-fold from its maximal (2500 nM) to 156 nM while keeping the combination response at the same level. Further, we found that the actual effect of combining ispinesib at 156 nM and ibrutinib at 0.78 nM is able to achieve the same effect compared to the combination of 2500 nM ispinesib and 0.78 nM ibrutinib. Taken together, the interaction landscape indicated that the combination of ispinesib and ibrutinib might be able to achieve a higher effect than individual drugs while maintaining acceptable doses and hence side effects. Such an interaction landscape might therefore provide a new way of detecting clinically relevant synergy beyond what a binary classification of drug interaction can answer. On the other hand, the interaction landscape analysis also revealed many antagonistic drug combinations. For example, the top antagonistic interaction occurred between ibrutinib and canertinib, a potent EGFR/HER family inhibitor. As can be seen from Fig. 5B, the strongest antagonism was found centered at the dose combination of 12.5 nM of ibrutinib and 625 nM of canertinib. The antagonism was also clearly visible in the dose–response matrix as the combination effect is almost universally lower than the individual drug effects. Although from the cancer treatment point of view, antagonistic drug combinations are often ignored, the mechanisms of the antagonistic interactions might be of equal importance to understand the cross-talks between the cancer signaling pathways, and these may also provide clinically important guidelines to avoid administrating a drug combination that may interfere each other by triggering such an antagonistic cross-talk in cancer.

**Fig. 6:**
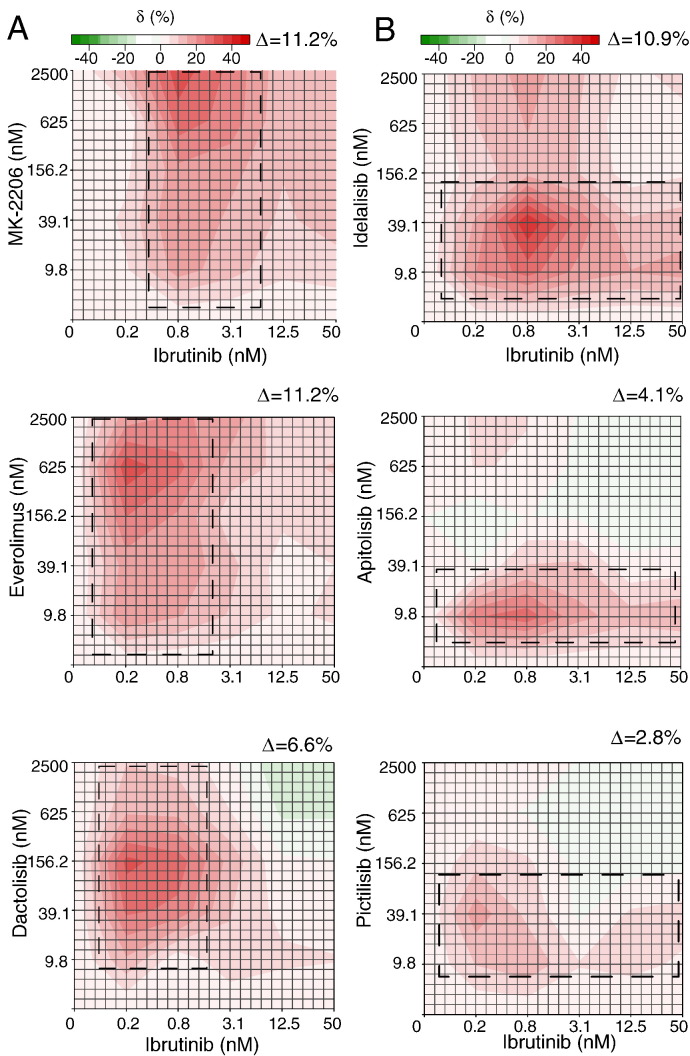
The different interaction patterns for PI3K inhibitors and ibrutinib. (A) Ibrutinib-driven synergy is triggered by a fixed dose of ibrutinib and becomes visible at the full dose ranges for a PI3K inhibitor, highlighted as a vertical box in the interaction landscape. (B) In contrast, PI3K-driven synergy is mainly constrained within a horizontal box aligned with the dose.

High delta scores (> 0.05 or 5%) were also confirmed for several drug combinations reported in the original Mathews Grinder study interacting with ibrutinib [14], such as Bcl-2 family inhibitor (navitoclax), PI3K pathway inhibitors (MK-2206, idelalisib (CAL-101), dactolisib (BEZ-235) and everolimus), as well as with common chemotherapeutic agents (doxorubicin, dexamethasone, docetaxel and plinabulin). The average delta score for these confirmed drug combinations was 0.1 (10.1%), which is significantly higher than the average (0.007 or 0.7%) of the total 466 combinations (Wilcoxon rank sum test, *p* = 2.1 × 10^− 6^). With their interaction landscapes available, it is now possible to differentiate the drugs of same mechanisms in terms of the interaction patterns. For these PI3K pathway inhibitors, we found that their interaction patterns can be classified into two categories: ibrutinib-driven (together with AKT and mTOR inhibitors MK-2206, everolimus and dactolisib), and PI3K inhibitor-driven (together with the CLL-approved PI3Kδ inhibitor idelalisib) (Fig. 6). Take MK-2206 as an example, higher delta scores were found for all the tested doses when ibrutinib was fixed at 0.78 nM. We may consider that such a synergy was most likely initiated by ibrutinib. On the contrary, the synergy region for idelalisib was mainly following the other direction in the landscape, producing a stable efficacy boost for ibrutinib. Therefore, one can attribute such a pattern as triggered by the mechanism of PI3K inhibition. We also found that two pan-PI3K inhibitors including pictilisib (GDC-0941) and apitolisib (GDC-0980) were showing the ibrutinib-driven pattern in the combination, but with delta scores lower than 0.05 (5%), and therefore were considered insignificant at the whole dose-matrix level (Fig. 6). Such a diluted synergy effect might be due to the limited specificity of such a pan-PI3K inhibition. Unfortunately, the Mathews Griner data does not contain replicates for the same drug combinations, which would have enabled evaluation of the significance of such interaction patterns using the statistical testing proposed in the Method section. Despite these limitations in the example data, our results suggest that the interaction landscape has the potential to capture patterns that are related to their underlying target interactions and thus warrants further mechanistic studies.

## 4 Discussion

Systematic evaluation of drug combination experiments to pinpoint synergistic interactions is a challenging task. In this work, we examined the limitations of the current reference models for assessing drug interactions in the high-throughput setup, and developed a novel ZIP model to capture the shift of interaction potency for a non-interactive drug combination. The ZIP model takes the advantages of both the Loewe and the Bliss models, aiming at a systematic assessment of various types of drug interactions patterns that may arise in a high-throughput drug combination screening. It utilizes the concept of zero interaction between two drugs to derive the expected effect, where the potency of the dose–response curve for one drug should remain unaltered after adding the other drug. We proposed the delta scoring to capture the deviation of the observed combination dose–response curves from the expectation, which can be calculated efficiently with fewer assumptions on the actual dose–response relations. For facilitating more systematic analysis over the whole dose–response matrix, we implemented a surface plot approach based on the delta scoring, to visualize the landscape of drug interaction over all the all tested dose pairs. The detailed drug synergy patterns should provide improved information on the dose optimization that warrants further exploration. These new features in the ZIP model allow for exploiting the rich data from a dose–response matrix experiment, where two drugs are tested at various dose pairs in a serially diluted manner.

Utilizing a public large-scale drug combination data [14], we have shown that the delta score based on the ZIP model can reproduce the reported synergistic combinations, while keeping the total number of synergistic combinations low, making it advantageous for the prioritization purposes. The delta score is centered at 0 and symmetric, which makes its interpretation more straightforward compared to the scoring based on the Loewe additive models. On the other hand, the delta score also considers the dose–response relationships of individual drugs and drug combinations, and therefore becomes more accurate at the characterization of potency changes in drug interactions, compared to the relatively simplistic HSA and Bliss independence models. Further, with the help of the interaction landscape analysis, one can always search for the particular patterns of synergy and antagonism within the dose combination matrices, and evaluate whether such a pattern is globally persistent at clinically relevant doses, or present only for a specific region in the dose matrix. We have also made available the R code and the dynamic report for running the calculation of delta score using the Mathews Griner data as an example (Supplementary File 1).

Although the current ZIP-based delta scoring was formulated based on the logistic curve fitting for dose–response relationships, the same principle about characterizing potency shift can be derived irrespective of the mathematical formulation of dose–responses. For example, with the exponential family *y* = 1 − exp(−*βx*), the change in the parameter *β* between the individual drugs and a drug combination can be used to quantify the degree of interaction potency. Since the main focus of this article was to introduce the ZIP concept, the mathematical extension of the ZIP model for other curve fitting functions is outside the scope of the present work, but worth exploring in the future. While the current ZIP model deals with the common phenotypic outcomes, such as percentage of cell inhibition, the model can be readily extended to the analysis of other functional phenotypes, such as the change of phosphorylation level of biomarkers in the downstream cancer pathways [20]. Despite several potential extensions, the present ZIP model has already shown its flexibility and interpretability when evaluating high-throughput drug combination data. Further, the interaction landscape analysis has also potential to extract detailed interaction patterns from a dose–response matrix, which may eventually provide valuable novel insights into how the mechanisms of these drugs are connected in the context of cellular pathways.

## Funding

This work was supported by the Academy of Finland (grants 272437, 269862, 279163 and 292611 for TA, 277293 for KW); and Cancer Society of Finland (JT, TA and KW). This project has received funding from the European Union's Horizon 2020 research and innovation program 2014–2020 under Grant Agreement No 634143 (MedBioinformatics).

## Author Contributions

B.Y. and J.T. implemented the approach and performed the experiments. J.T., T.A. and K.W. conceived the study and designed the experiments. J.T. developed the method and supervised the study. All authors contributed to the writing of the manuscript.

The following are the supplementary data related to this article

Supplementary Fig. 3 The interaction landscapes of the 466 drug combinations in the Mathews Griner data.

Supplementary Table 1 The interaction scores for the 466 drug combinations in the Mathews Griner data.

Supplementary Table 2 The *p*-values for the AUROC differences between the interaction scores using the DeLong's test.

Supplementary File 1 The R source code for calculating delta scores and the dynamic report including user instructions.

## Figures and Tables

**Table 1:** Summary statistics of the drug interaction scores in the Mathews Griner data.

Score	Median	Mean	Skewness	Reference point
MedianExcess	66.46	66.29	− 1.12	0
NumExcess	13.00	13.59	0.08	0
ExcessHSA	− 63.0	− 86.50	− 0.27	0
ExcessCRX	− 45.52	− 83.74	− 18.17	0
LS3 × 3	− 5.72	− 8.15	− 0.88	0
Beta	0.99	0.98	− 0.34	1
Gamma	0.98	0.97	− 0.99	1
Delta	0.007	0.009	0.16	0

## Appendix A

### Appendix A1

We prove that $y_{\mathrm{ZIP}}=\frac{{\left(\frac{x_{1}}{m_{1}}\right)}^{\lambda_{1}}}{1+{\left(\frac{x_{1}}{m_{1}}\right)}^{\lambda_{1}}}+\frac{{\left(\frac{x_{2}}{m_{2}}\right)}^{\lambda_{2}}}{1+{\left(\frac{x_{2}}{m_{2}}\right)}^{\lambda_{2}}}-\frac{{\left(\frac{x_{1}}{m_{1}}\right)}^{\lambda_{1}}}{1+{\left(\frac{x_{1}}{m_{1}}\right)}^{\lambda_{1}}}\frac{{\left(\frac{x_{2}}{m_{2}}\right)}^{\lambda_{2}}}{1+{\left(\frac{x_{2}}{m_{2}}\right)}^{\lambda_{2}}}$.

Let $A={\left(\frac{m_{1}}{x_{1}}\right)}^{\lambda_{1}}$ and $B={\left(\frac{m_{2}}{x_{2}}\right)}^{\lambda_{2}}$ then according to Eq. (14) and Eq. (15)$y_{\mathrm{ZIP}}^{1\leftarrow 2}=\frac{\frac{1}{1+B}+\frac{1}{A}}{1+\frac{1}{A}}$ and $y_{\mathrm{ZIP}}^{2\leftarrow 1}=\frac{\frac{1}{1+A}+\frac{1}{B}}{1+\frac{1}{B}}$. It can be hence derived that $y_{\mathrm{ZIP}}=y_{\mathrm{ZIP}}^{1\leftarrow 2}=y_{\mathrm{ZIP}}^{1\leftarrow 2}=\frac{1+A+B}{1+A+B+\mathrm{AB}}$. Q.E.D

### Appendix A2

We prove that for a sham experiment *δ* = 0.

In a sham experiment, both drugs have the same dose–effect curve, denoted as

(A2.1) $$y=\frac{{\left(\frac{x}{m}\right)}^{\lambda }}{1+{\left(\frac{x}{m}\right)}^{\lambda }}\text{.}$$

Since it is a Sham experiment, it holds that *m*_1_ = *m*_2_ = *m*_1 → 2_ = *m*_2 → 1_ and *λ*_1_ = *λ*_2_ = *λ*_1 → 2_ = *λ*_2 → 1_. According to Eq. (19)

(A2.2) $$\begin{array}{l} \delta =\frac{1}{2}\left(\frac{\frac{1}{1+{\left(\frac{m_{2}}{x_{2}}\right)}^{\lambda_{2}}}+{\left(\frac{x_{1}}{m_{2\to 1}}\right)}^{\lambda_{2\to 1}}}{1+{\left(\frac{x_{1}}{m_{2\to 1}}\right)}^{\lambda_{2\to 1}}}+\frac{\frac{1}{1+{\left(\frac{m_{1}}{x_{1}}\right)}^{\lambda_{1}}}+{\left(\frac{x_{2}}{m_{1\to 2}}\right)}^{\lambda_{1\to 2}}}{1+{\left(\frac{x_{2}}{m_{1\to 2}}\right)}^{\lambda_{1\to 2}}}\right)-\left(\frac{{\left(\frac{x_{1}}{m_{1}}\right)}^{\lambda_{1}}}{1+{\left(\frac{x_{1}}{m_{1}}\right)}^{\lambda_{1}}}+\frac{{\left(\frac{x_{2}}{m_{2}}\right)}^{\lambda_{2}}}{1+{\left(\frac{x_{2}}{m_{2}}\right)}^{\lambda_{2}}}-\frac{{\left(\frac{x_{1}}{m_{1}}\right)}^{\lambda_{1}}}{1+{\left(\frac{x_{1}}{m_{1}}\right)}^{\lambda_{1}}}\frac{{\left(\frac{x_{2}}{m_{2}}\right)}^{\lambda_{2}}}{1+{\left(\frac{x_{2}}{m_{2}}\right)}^{\lambda_{2}}}\right)\\ =\frac{1}{2}\left(\frac{\frac{1}{1+{\left(\frac{m}{x_{2}}\right)}^{\lambda }}+{\left(\frac{x_{1}}{m}\right)}^{\lambda }}{1+{\left(\frac{x_{1}}{m}\right)}^{\lambda }}+\frac{\frac{1}{1+{\left(\frac{m}{x_{1}}\right)}^{\lambda }}+{\left(\frac{x_{2}}{m}\right)}^{\lambda }}{1+{\left(\frac{x_{2}}{m}\right)}^{\lambda }}\right)-\left(\frac{{\left(\frac{x_{1}}{m}\right)}^{\lambda }{\left(\frac{x_{2}}{m}\right)}^{\lambda }+{\left(\frac{x_{1}}{m}\right)}^{\lambda }+{\left(\frac{x_{2}}{m}\right)}^{\lambda }}{\left(1+{\left(\frac{x_{1}}{m}\right)}^{\lambda }\right)\left(1+{\left(\frac{x_{2}}{m}\right)}^{\lambda }\right)}\right)\\ =\frac{1}{2}\left(\frac{2{\left(\frac{x_{1}}{m}\right)}^{\lambda }{\left(\frac{x_{2}}{m}\right)}^{\lambda }+{\left(\frac{x_{1}}{m}\right)}^{\lambda }+{\left(\frac{x_{2}}{m}\right)}^{\lambda }+\frac{1+{\left(\frac{x_{2}}{m}\right)}^{\lambda }}{1+{\left(\frac{m}{x_{2}}\right)}^{\lambda }}+\frac{1+{\left(\frac{x_{1}}{m}\right)}^{\lambda }}{1+{\left(\frac{m}{x_{1}}\right)}^{\lambda }}}{\left(1+{\left(\frac{x_{1}}{m}\right)}^{\lambda }\right)\left(1+{\left(\frac{x_{2}}{m}\right)}^{\lambda }\right)}\right)-\left(\frac{{\left(\frac{x_{1}}{m}\right)}^{\lambda }{\left(\frac{x_{2}}{m}\right)}^{\lambda }+{\left(\frac{x_{1}}{m}\right)}^{\lambda }+{\left(\frac{x_{2}}{m}\right)}^{\lambda }}{\left(1+{\left(\frac{x_{1}}{m}\right)}^{\lambda }\right)\left(1+{\left(\frac{x_{2}}{m}\right)}^{\lambda }\right)}\right)\text{.} \end{array}$$

It can be shown that:

(A2.3) $${\left(\frac{x_{1}}{m}\right)}^{\lambda }={\frac{1+\left(\frac{x_{1}}{m}\right)}{1+{\left(\frac{m}{x_{1}}\right)}^{\lambda }}}^{\lambda }$$

(A2.4) $${\left(\frac{x_{2}}{m}\right)}^{\lambda }={\frac{1+\left(\frac{x_{2}}{m}\right)}{1+{\left(\frac{m}{x_{2}}\right)}^{\lambda }}}^{\lambda }\text{.}$$

Placing Eqs. (A2.3), (A2.4) back into Eq. (A2.2) gives:

$$\delta =\left(\frac{{\left(\frac{x_{1}}{m}\right)}^{\lambda }+{\left(\frac{x_{1}}{m}\right)}^{\lambda }{\left(\frac{x_{2}}{m}\right)}^{\lambda }+{\left(\frac{x_{2}}{m}\right)}^{\lambda }}{\left(1+{\left(\frac{x_{1}}{m}\right)}^{\lambda }\right)\left(1+{\left(\frac{x_{2}}{m}\right)}^{\lambda }\right)}\right)-\left(\frac{{\left(\frac{x_{1}}{m}\right)}^{\lambda }{\left(\frac{x_{2}}{m}\right)}^{\lambda }+{\left(\frac{x_{1}}{m}\right)}^{\lambda }+{\left(\frac{x_{2}}{m}\right)}^{\lambda }}{\left(1+{\left(\frac{x_{1}}{m}\right)}^{\lambda }\right)\left(1+{\left(\frac{x_{2}}{m}\right)}^{\lambda }\right)}\right)=0\text{.}\text{Q.E.D}$$

### Appendix A3

The derivation of the *δ* score for a full 4-parameter log-logistic function $y=\frac{E_{\text{min}}+E_{\text{max}}{\left(\frac{x}{m}\right)}^{\lambda }}{1+{\left(\frac{x}{m}\right)}^{\lambda }}\text{:}$

$$\begin{array}{l} \delta =\frac{1}{2}\left(\frac{\frac{E_{\text{min}}+E_{\text{max}}{\left(\frac{x_{2}}{m_{2}}\right)}^{\lambda_{2}}}{1+{\left(\frac{x_{2}}{m_{2}}\right)}^{\lambda_{2}}}+E_{\text{max}}{\left(\frac{x_{1}}{m_{2\to 1}}\right)}^{\lambda_{2\to 1}}}{1+{\left(\frac{x_{1}}{m_{2\to 1}}\right)}^{\lambda_{2\to 1}}}+\frac{\frac{E_{\text{min}}+E_{\text{max}}{\left(\frac{x_{1}}{m_{1}}\right)}^{\lambda_{1}}}{1+{\left(\frac{x_{1}}{m_{1}}\right)}^{\lambda_{1}}}+E_{\text{max}}{\left(\frac{x_{2}}{m_{1\to 2}}\right)}^{\lambda_{1\to 2}}}{1+{\left(\frac{x_{2}}{m_{1\to 2}}\right)}^{\lambda_{1\to 2}}}\right)-\\ \left(\frac{E_{\text{min}}+E_{\text{max}}{\left(\frac{x_{1}}{m_{1}}\right)}^{\lambda_{1}}}{1+{\left(\frac{x_{1}}{m_{1}}\right)}^{\lambda_{1}}}+\frac{E_{\text{min}}+E_{\text{max}}{\left(\frac{x_{2}}{m_{2}}\right)}^{\lambda_{2}}}{1+{\left(\frac{x_{2}}{m_{2}}\right)}^{\lambda_{2}}}-\frac{E_{\text{min}}+E_{\text{max}}{\left(\frac{x_{1}}{m_{1}}\right)}^{\lambda_{1}}}{1+{\left(\frac{x_{1}}{m_{1}}\right)}^{\lambda_{1}}}\frac{E_{\text{min}}+E_{\text{max}}{\left(\frac{x_{2}}{m_{2}}\right)}^{\lambda_{2}}}{1+{\left(\frac{x_{2}}{m_{2}}\right)}^{\lambda_{2}}}\right)\text{.} \end{array}$$

## References

[1] Flaherty K.T., Infante J.R., Daud A., Gonzalez R., Kefford R.F. (2012). Combined BRAF and MEK inhibition in melanoma with BRAF V600 mutations. N Engl J Med.

[2] Fitzgerald J.B., Schoeberl B., Nielsen U.B., Sorger P.K. (2006). Systems biology and combination therapy in the quest for clinical efficacy. Nat Chem Biol.

[3] Lehár J., Krueger A.S., Avery W., Heilbut A.M., Johansen L.M. (2009). Synergistic drug combinations tend to improve therapeutically relevant selectivity. Nat Biotechnol.

[4] Borisy A.A., Elliott P.J., Hurst N.W., Lee M.S., Lehar J. (2003). Systematic discovery of multicomponent therapeutics. Proc Natl Acad Sci U S A.

[5] Berenbaum M.C. (1989). What is synergy?. Pharmacol Rev.

[6] Loewe S. (1953). The problem of synergism and antagonism of combined drugs. Arzneimittelforschung.

[7] Bliss C.I. (1939). The toxicity of poisons applied jointly. Ann Appl Biol.

[8] Greco W.R., Bravo G., Parsons J.C. (1995). The search for synergy: a critical review from a response surface perspective. Pharmacol Rev.

[9] Chou T.-C. (2010). Drug combination studies and their synergy quantification using the Chou–Talalay method. Cancer Res.

[10] Lee S. (2010). Drug interaction: focusing on response surface models. Korean J Anesthesiol.

[11] Chou T.-C. (2006). Theoretical basis, experimental design, and computerized simulation of synergism and antagonism in drug combination studies. Pharmacol Rev.

[12] Ritz C., Streibig J.C. (2005). Bioassay analysis using R. J Stat Softw.

[13] Lee J.J., Kong M., Ayers G.D., Lotan R. (2007). Interaction index and different methods for determining drug interaction in combination therapy. J Biopharm Stat.

[14] Mathews Griner L.A., Guha R., Shinn P., Young R.M., Keller J.M. (2014). High-throughput combinatorial screening identifies drugs that cooperate with ibrutinib to kill activated B-cell-like diffuse large B-cell lymphoma cells. Proc Natl Acad Sci U S A.

[15] Tallarida R.J. (2006). An overview of drug combination analysis with isobolograms. J Pharmacol Exp Ther.

[16] Byrd J.C., Furman R.R., Coutre S.E., Flinn I.W., Burger J.A. (2013). Targeting BTK with ibrutinib in relapsed chronic lymphocytic leukemia. N Engl J Med.

[17] Zhao W., Sachsenmeier K., Zhang L., Sult E., Hollingsworth R.E. (2014). A new bliss independence model to analyze drug combination data. J Biomol Screen.

[18] Jansen G., Lee A.Y., Epp E., Fredette A., Surprenant J. (2009). Chemogenomic profiling predicts antifungal synergies. Mol Syst Biol.

[19] Rath O., Kozielski F. (2012). Kinesins and cancer. Nat Rev Cancer.

[20] Stuhlmiller T.J., Miller S.M., Zawistowski J.S., Nakamura K., Beltran A.S. (2015). Inhibition of lapatinib-induced kinome reprogramming in ERBB2-positive breast cancer by targeting BET family bromodomains. Cell Rep.

