# Corrigendum to “Searching for drug synergy in complex dose–response landscapes using an interaction potency model” [Comput. Struct. Biotechnol. J. 13 (2015) 504–513]

**DOI:** 10.1016/j.csbj.2017.07.003

**Published:** 2017-07-25

**Authors:** Bhagwan Yadav, Krister Wennerberg, Tero Aittokallio, Jing Tang

**Affiliations:** Institute for Molecular Medicine Finland (FIMM), FI-00014, University of Helsinki, Finland

The authors regret that there was a typo in Eq. (8) and that *y*_***LOEWE***_ should be *y*_*c*_. The corrected equation is:

(8) $$\mathrm{CI}=\frac{x_{1}}{m_{1}{\left(\frac{y_{c}-E_{\min}^{1}}{E_{\max}^{1}-y_{c}}\right)}^{1/\lambda_{1}}}+\frac{x_{2}}{m_{2}{\left(\frac{y_{c}-E_{\min}^{2}}{E_{\max}^{2}-y_{c}}\right)}^{1/\lambda_{2}}},$$

Eq. (8) was used for introducing the concept of the Combination Index (CI). Calculation of CI used the R package SYNERGY which was based on the correct equation as above and therefore this typo won't affect any results and conclusions in the paper. The authors would like to apologise for any inconvenience caused.

