# Supplementary figures and images for: Searching for Drug Synergy in Complex Dose–Response Landscapes Using an Interaction Potency Model
Source: Comput Struct Biotechnol J. 2015 Sep 25;13:504–13. doi: 10.1016/j.csbj.2015.09.001 (PMC4759128; doi:10.1016/j.csbj.2015.09.001)

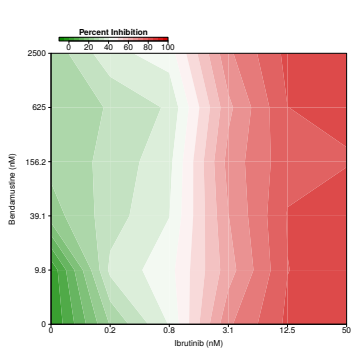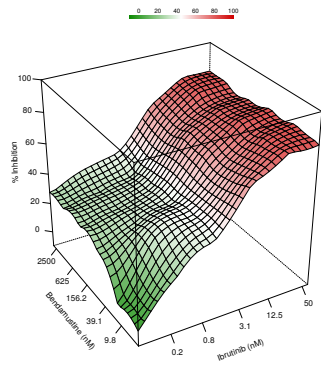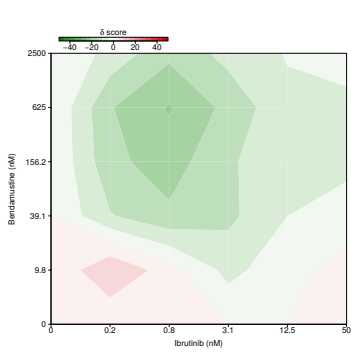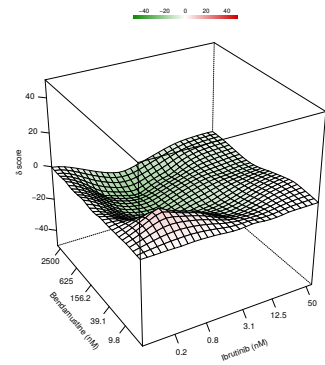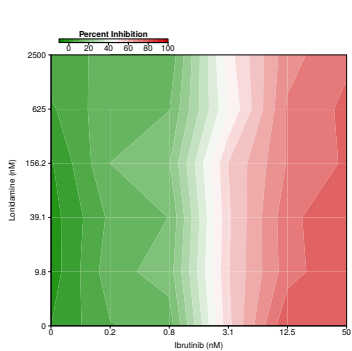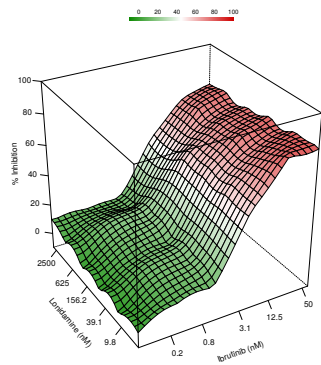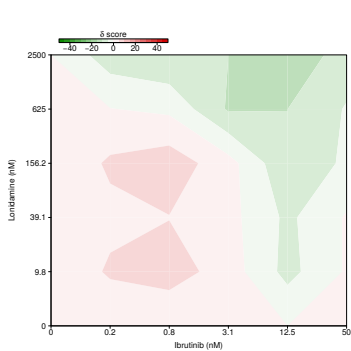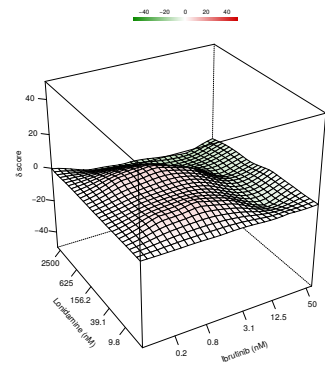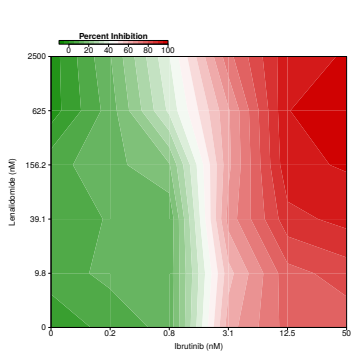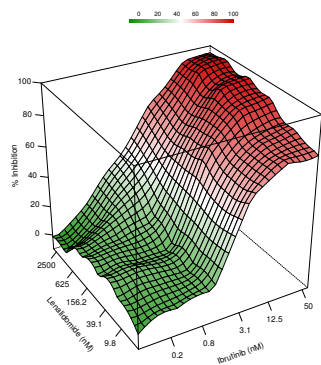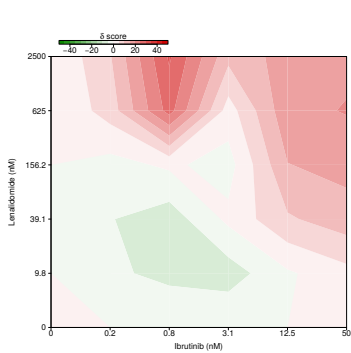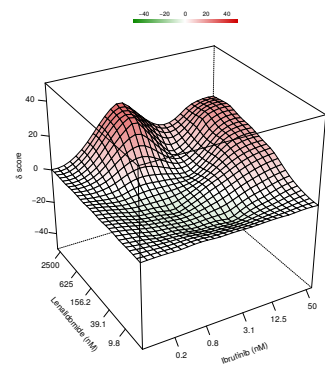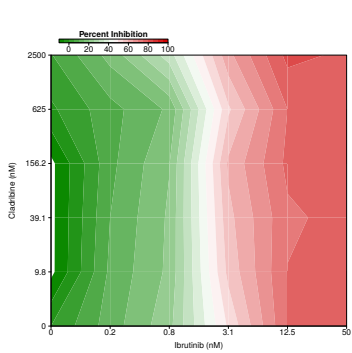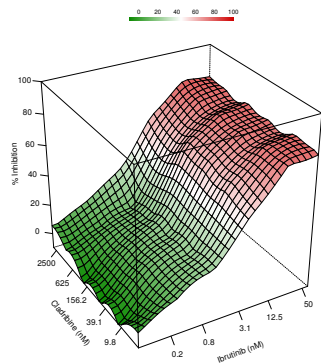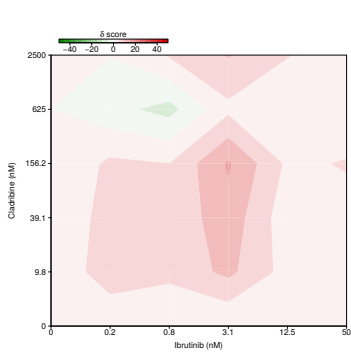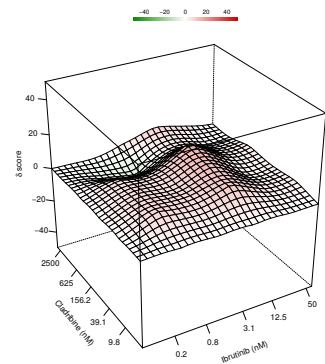

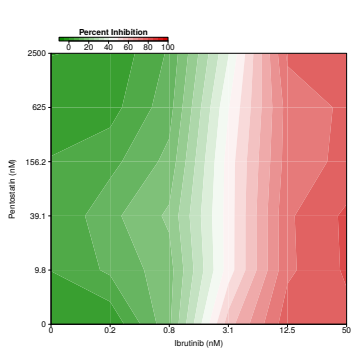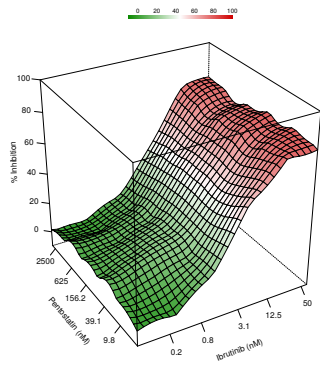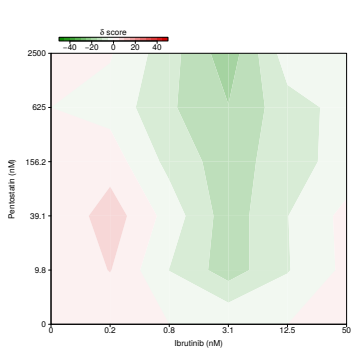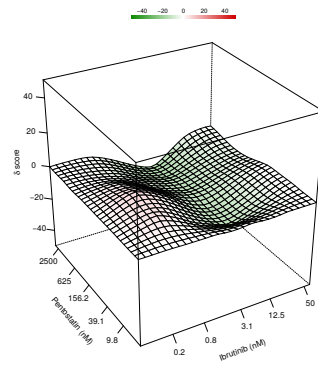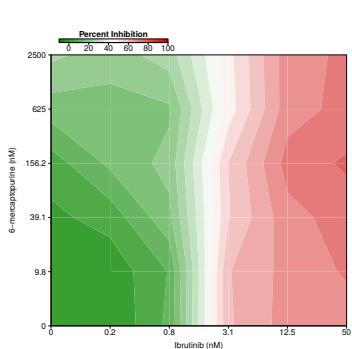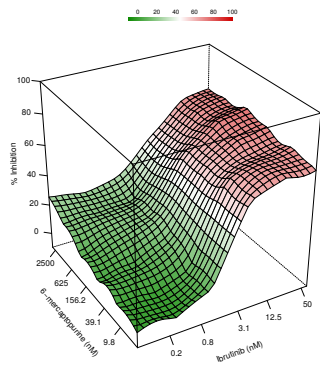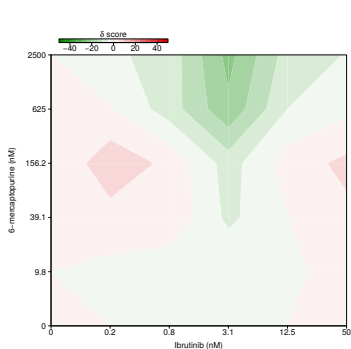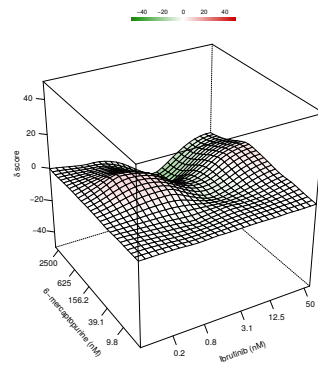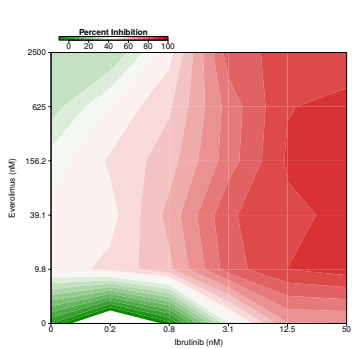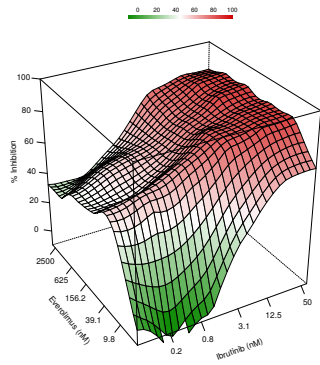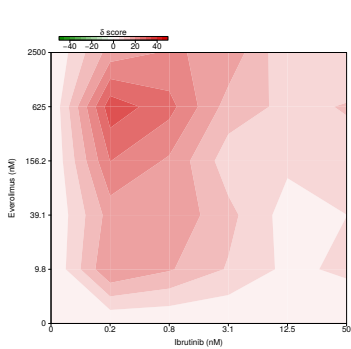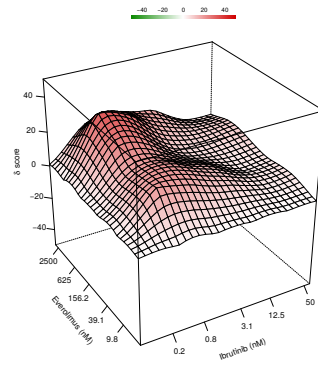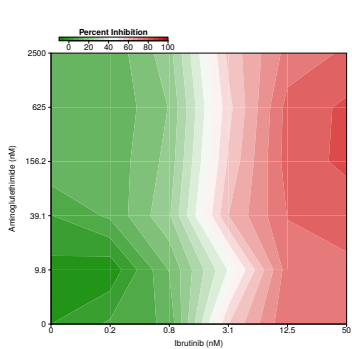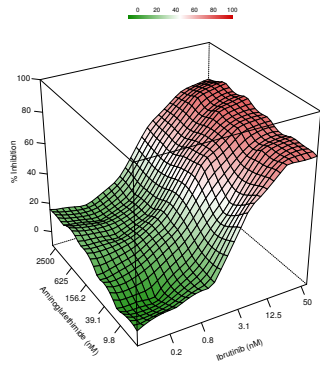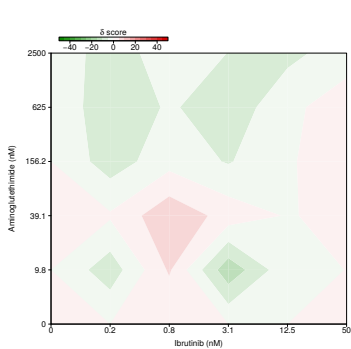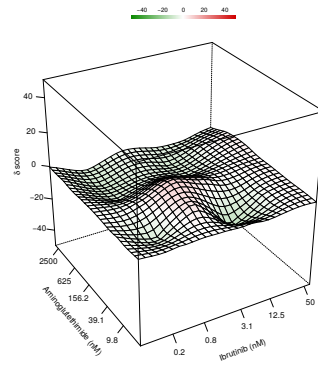

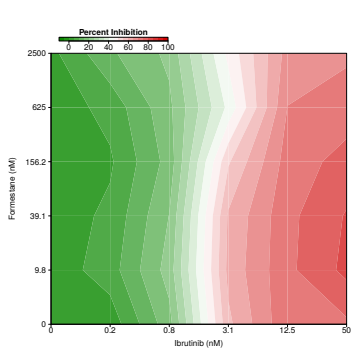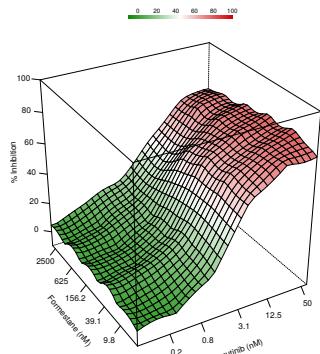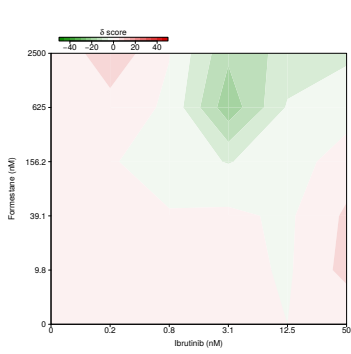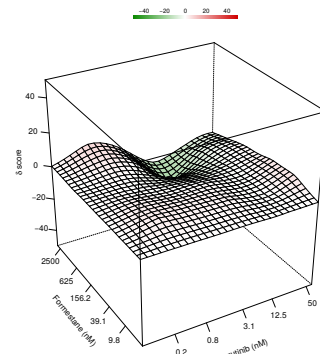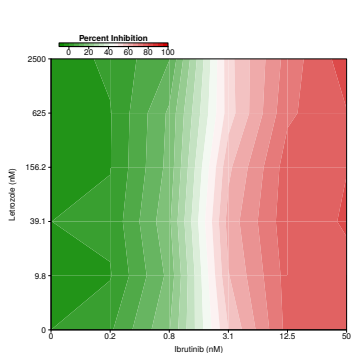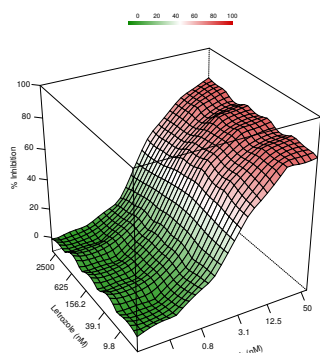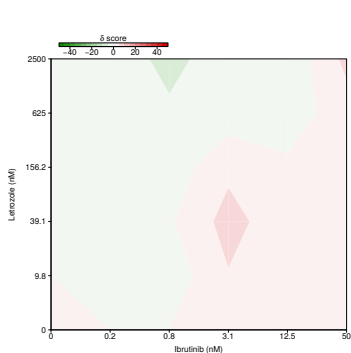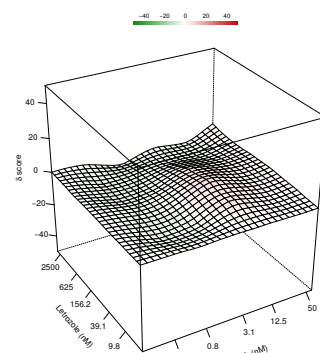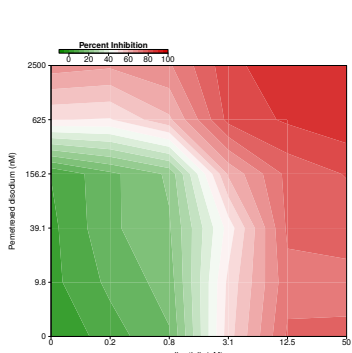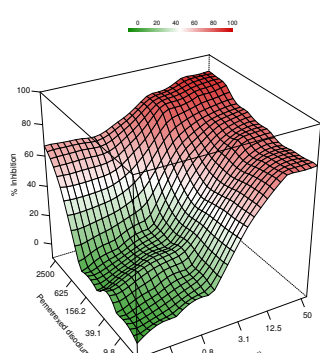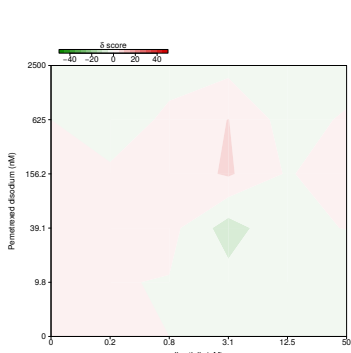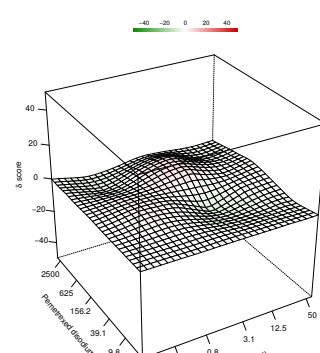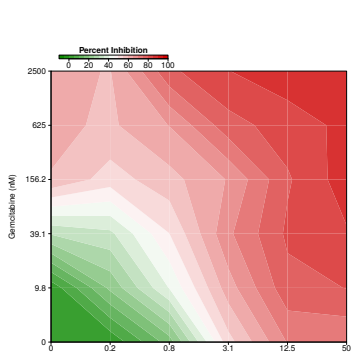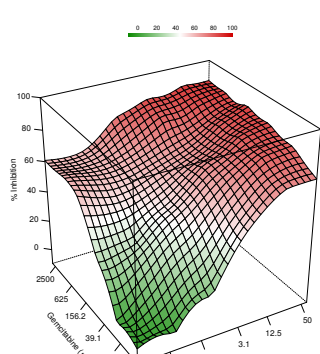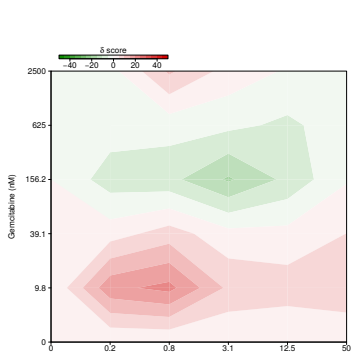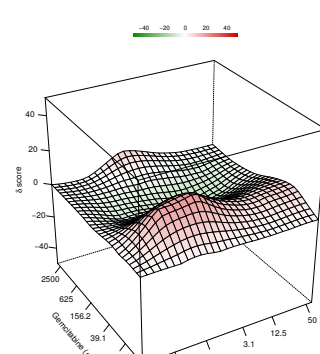



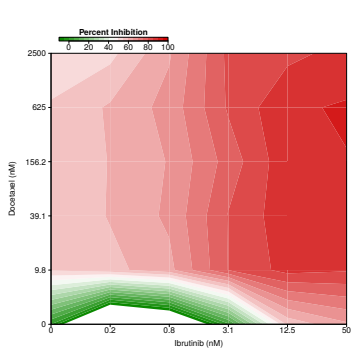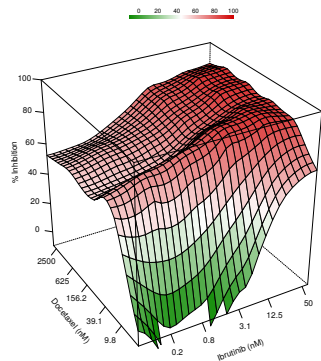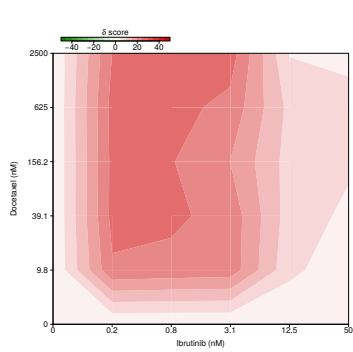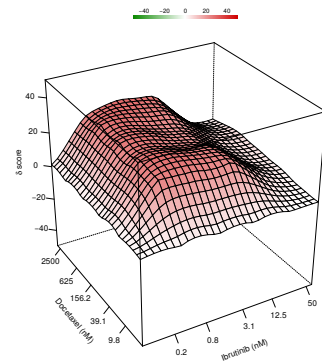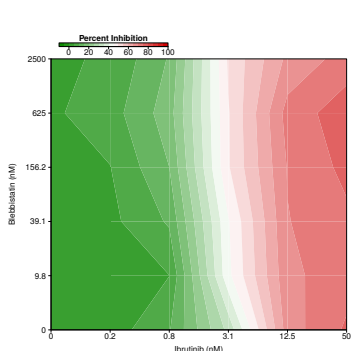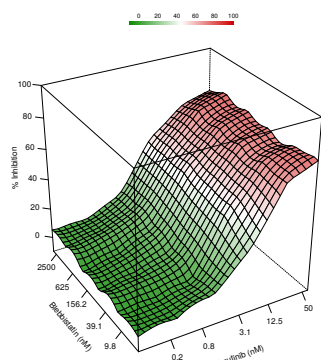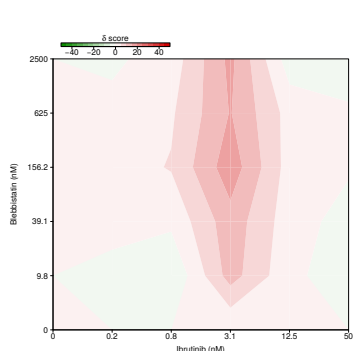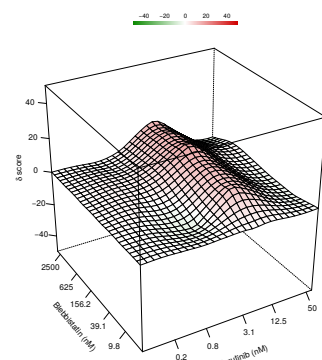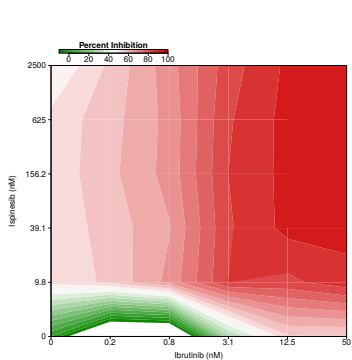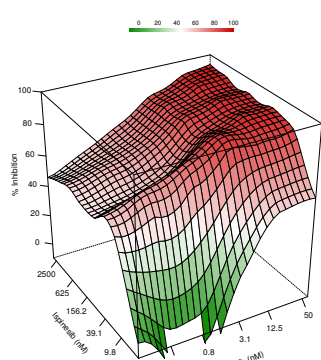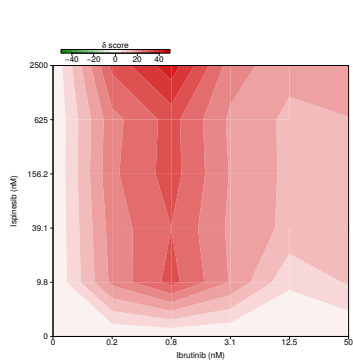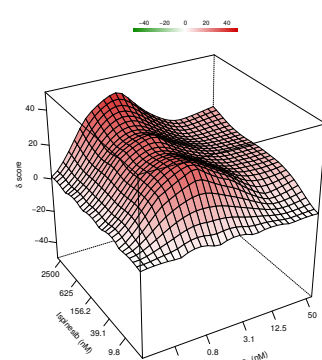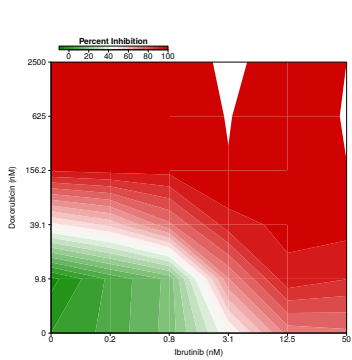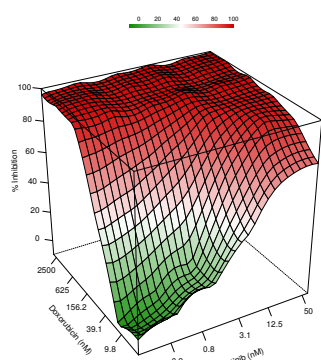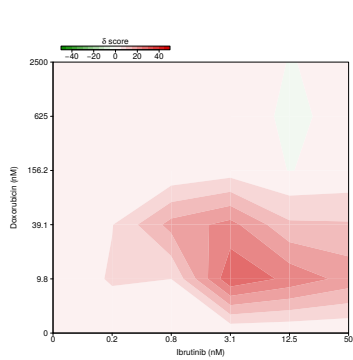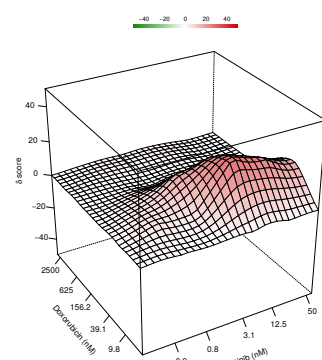

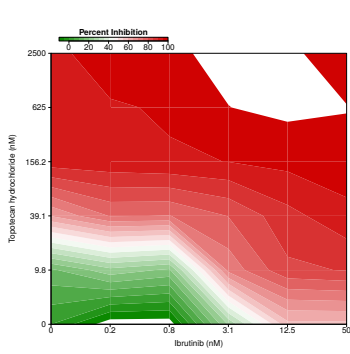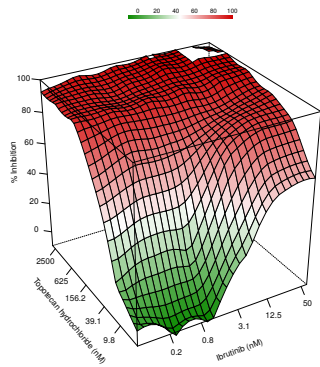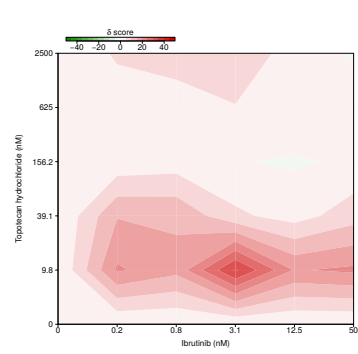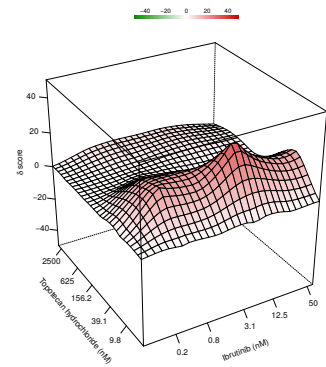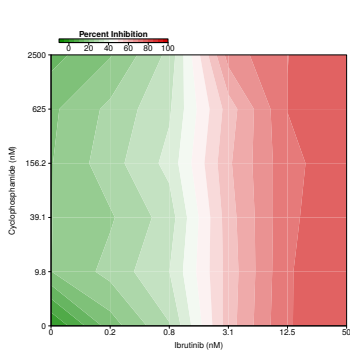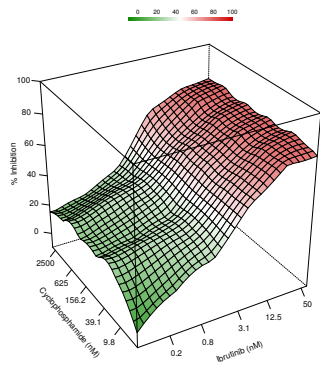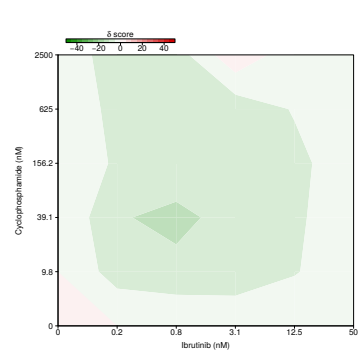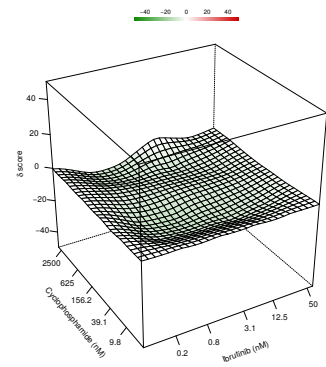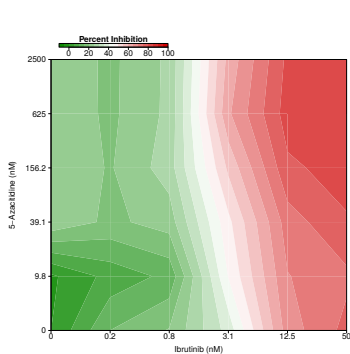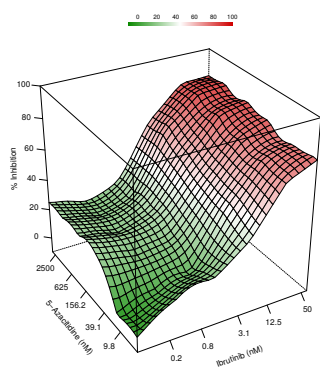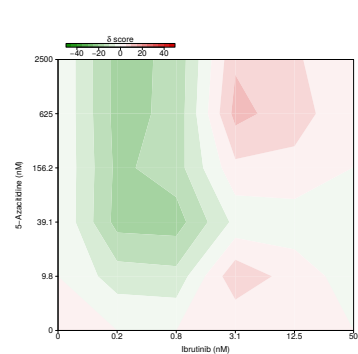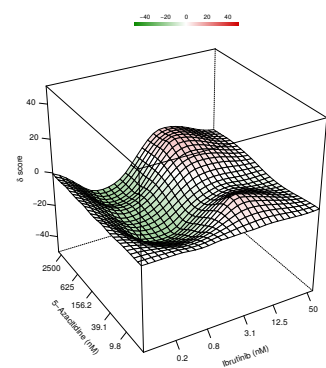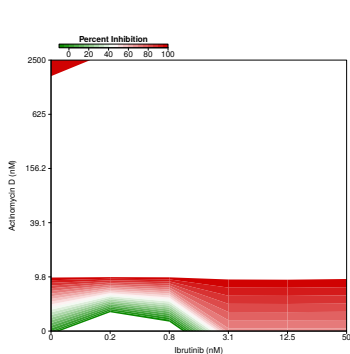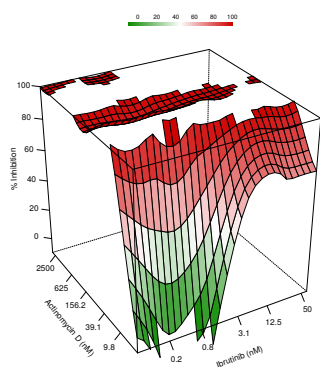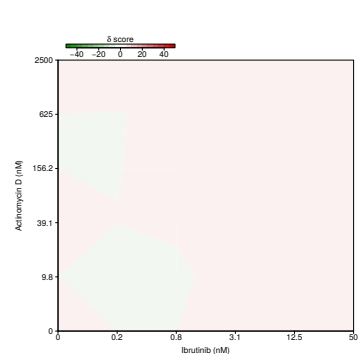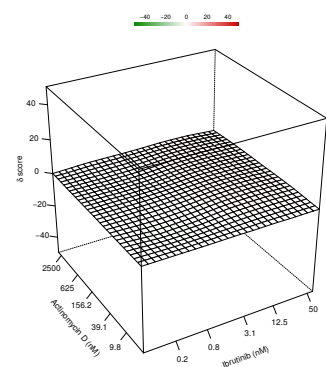

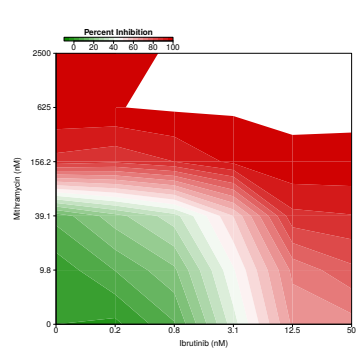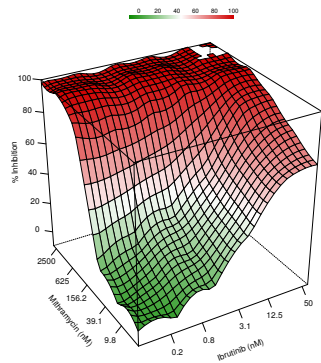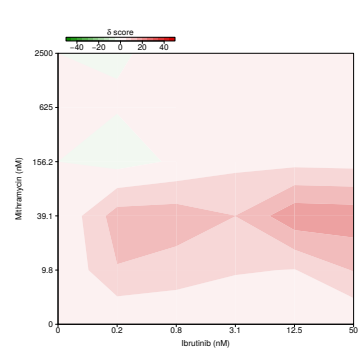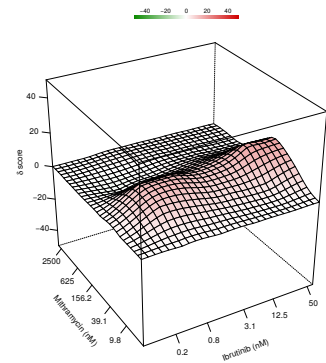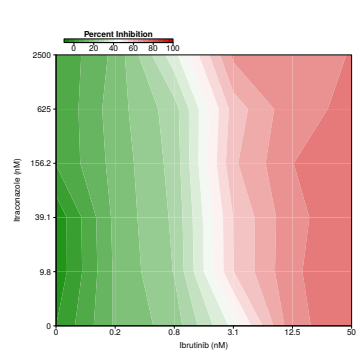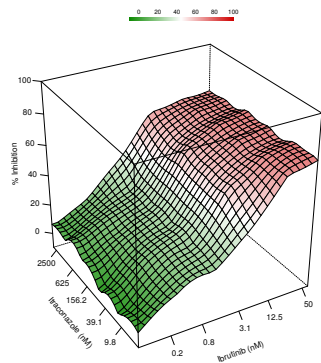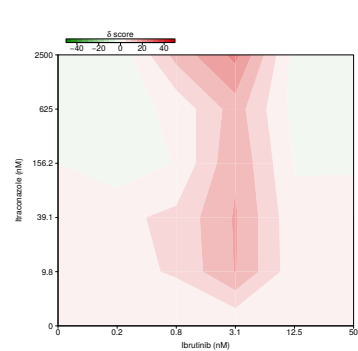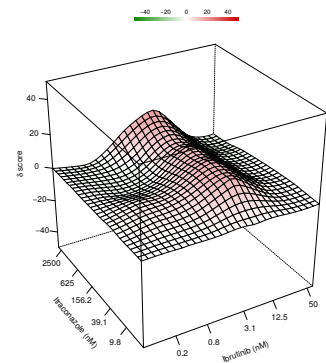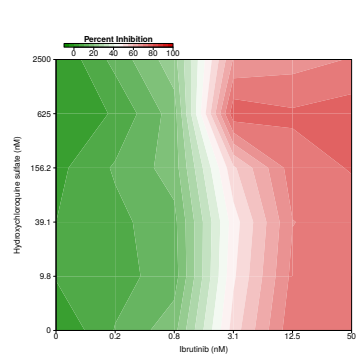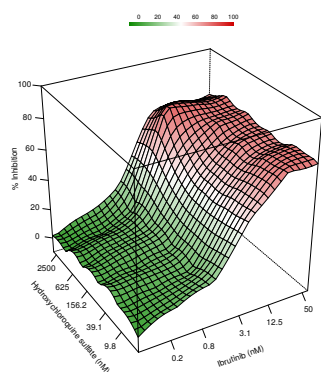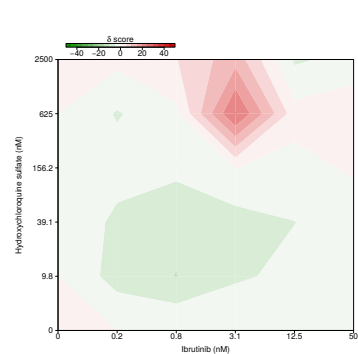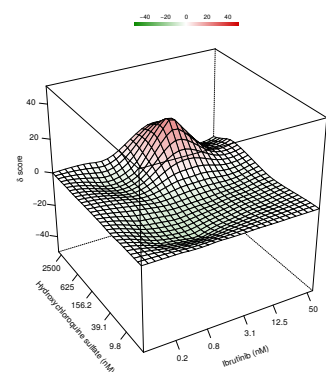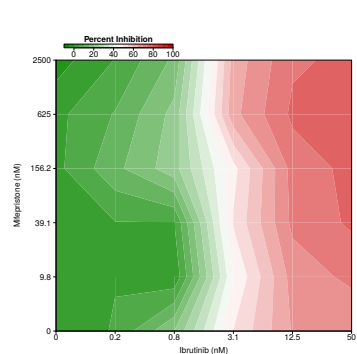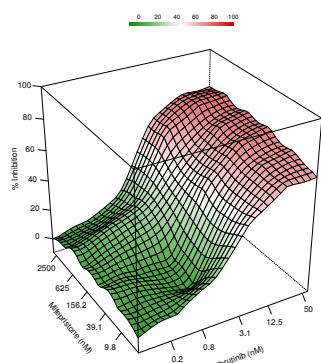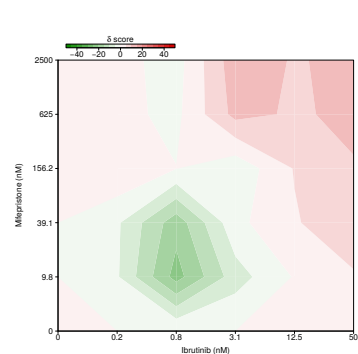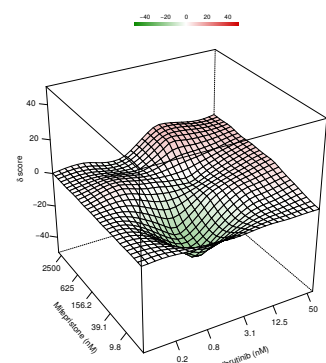

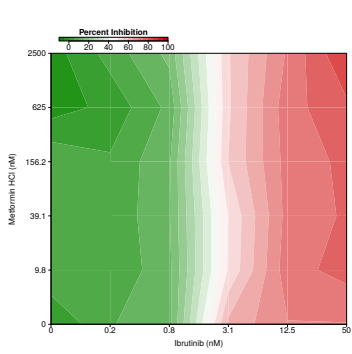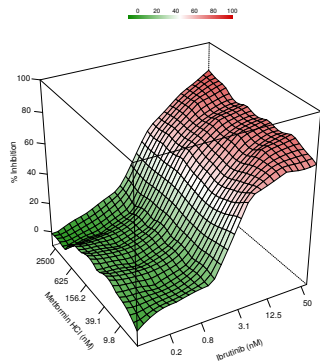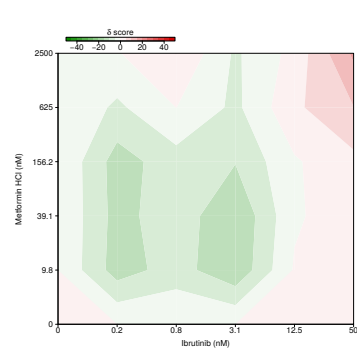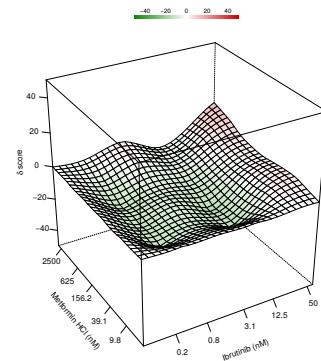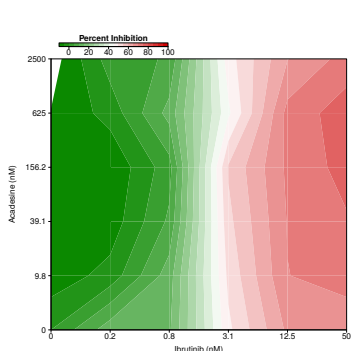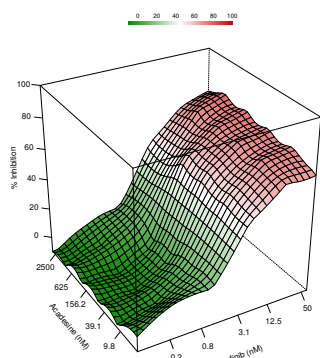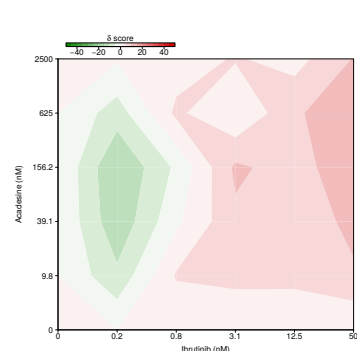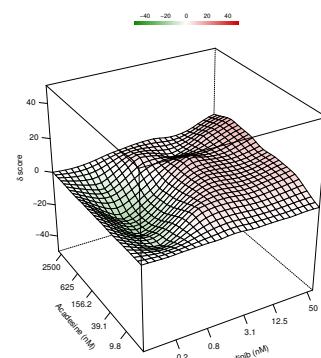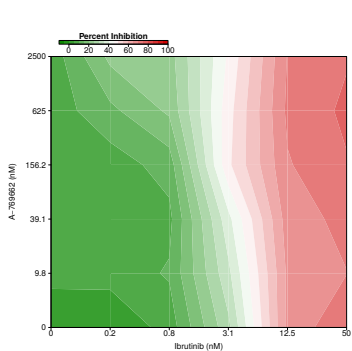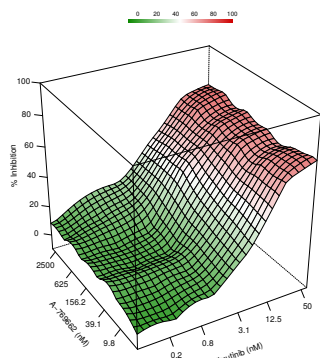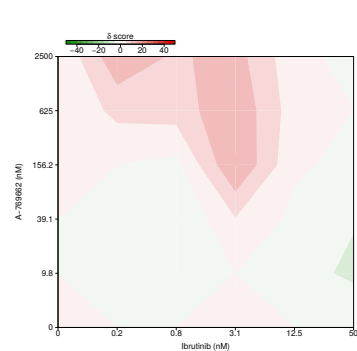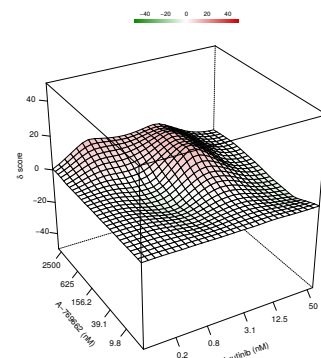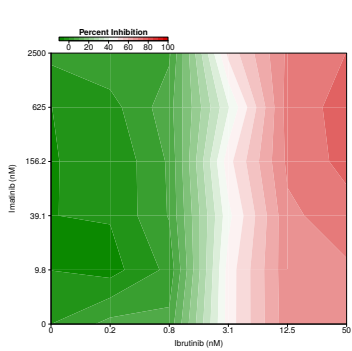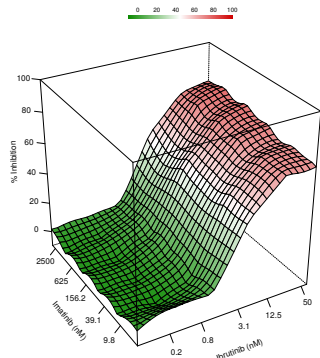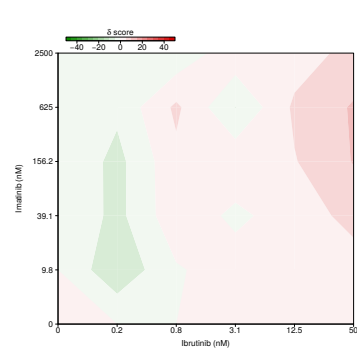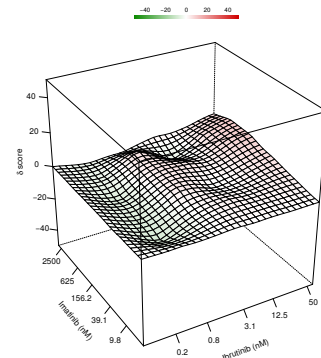

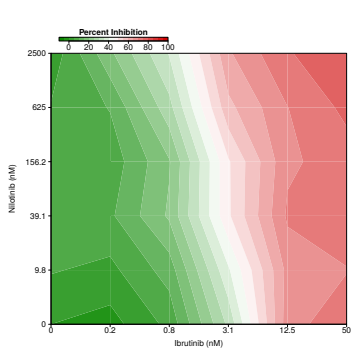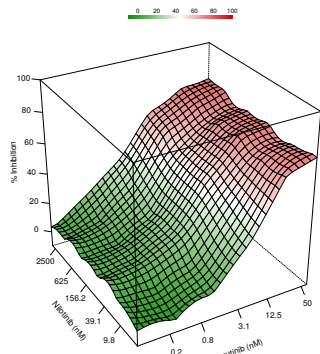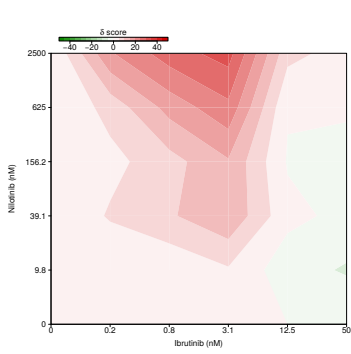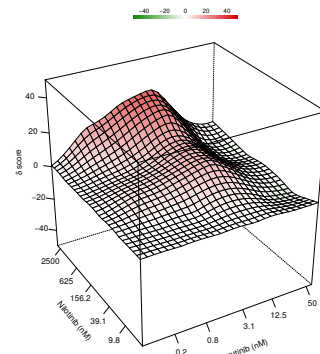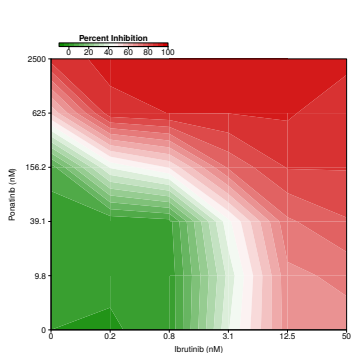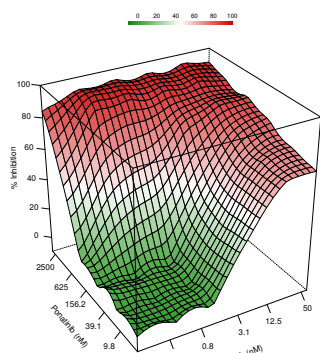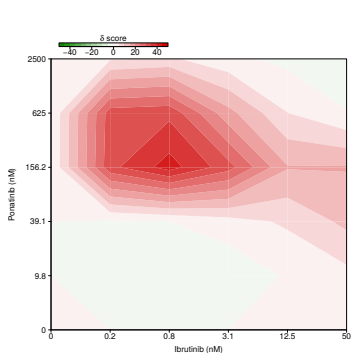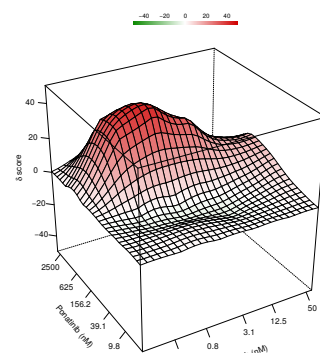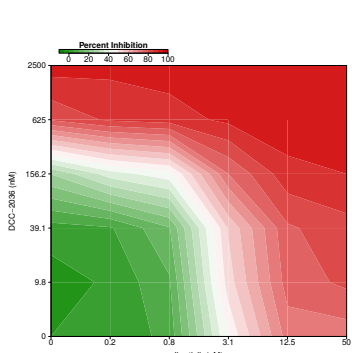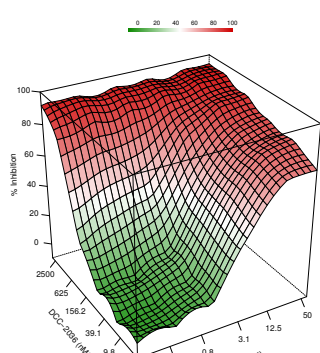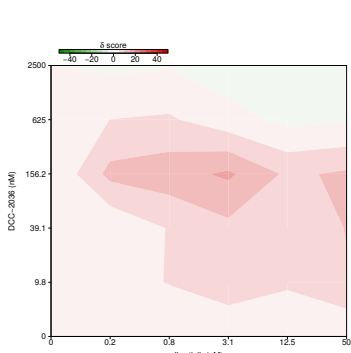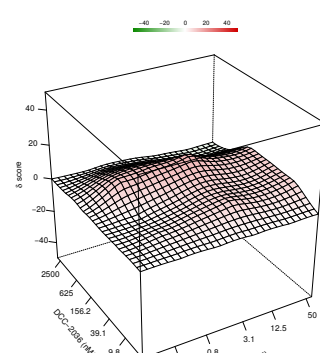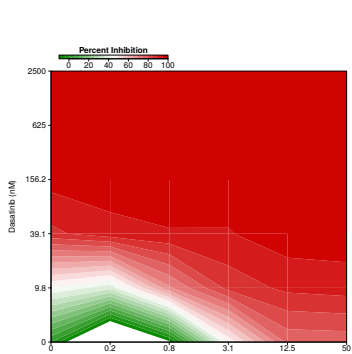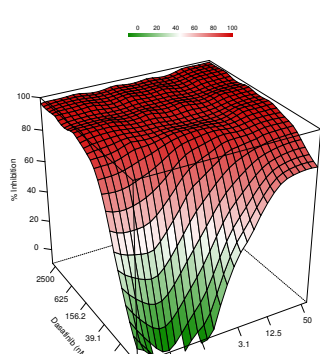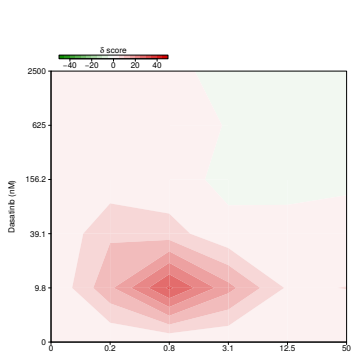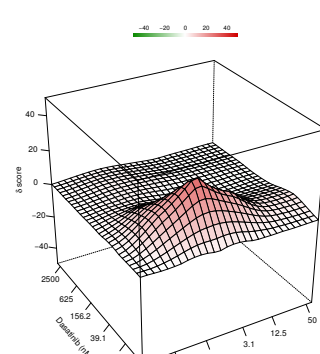

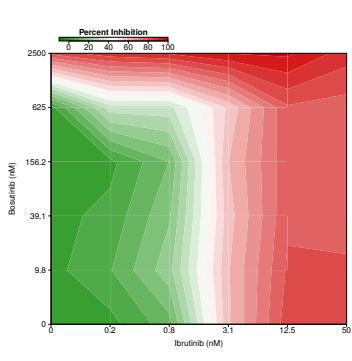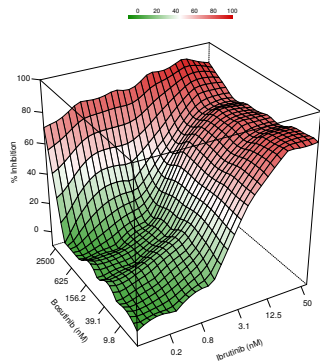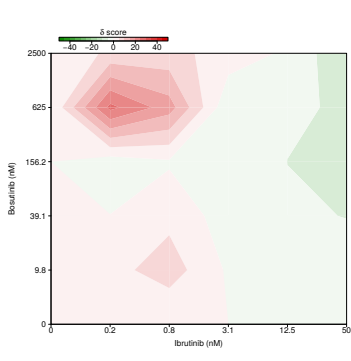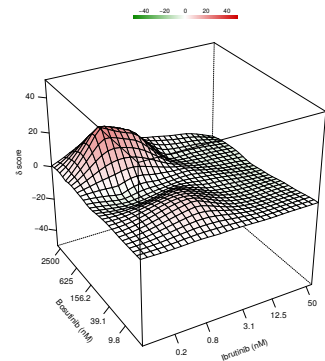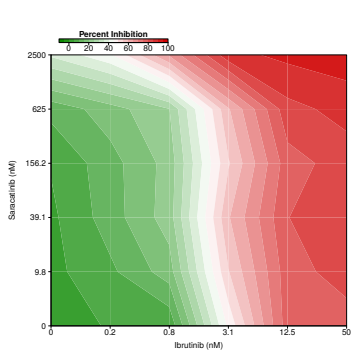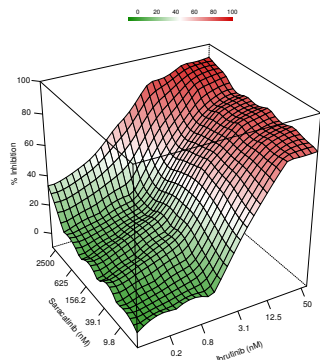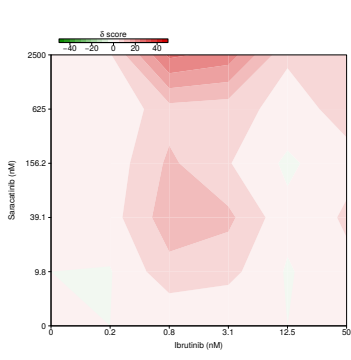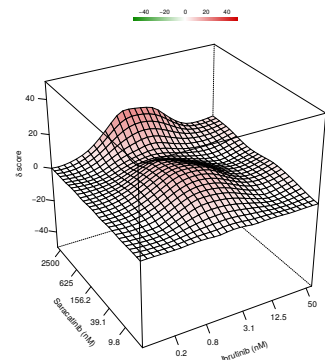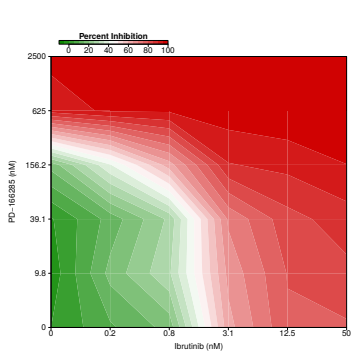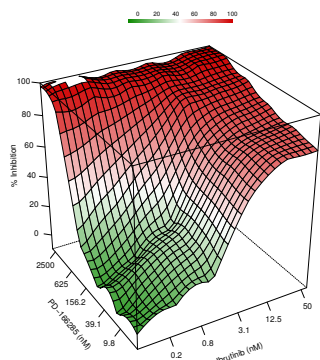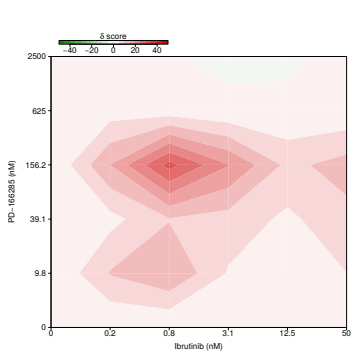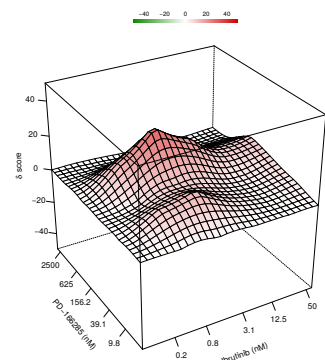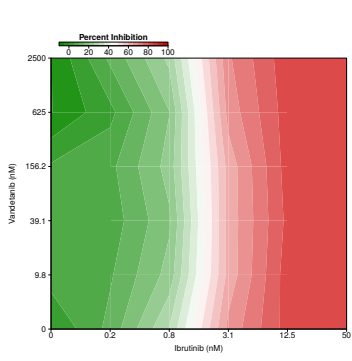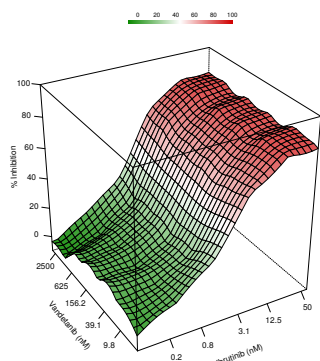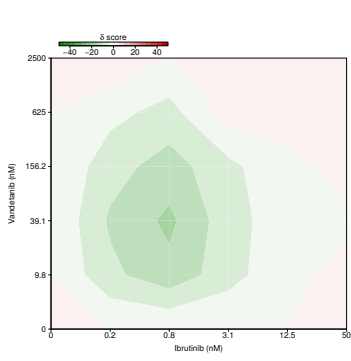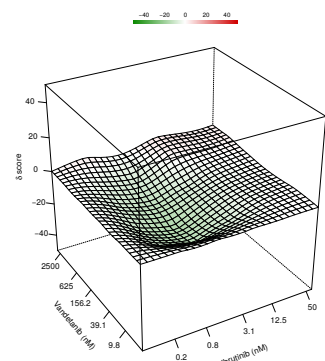

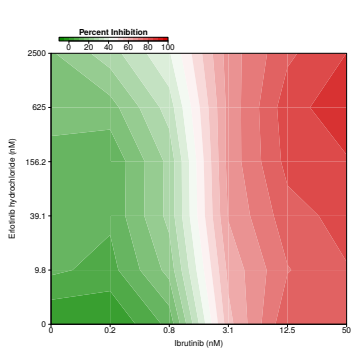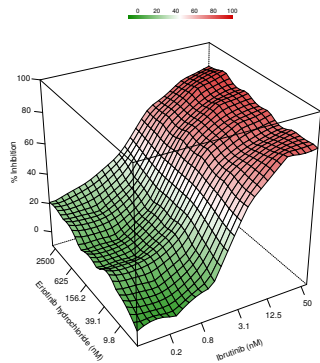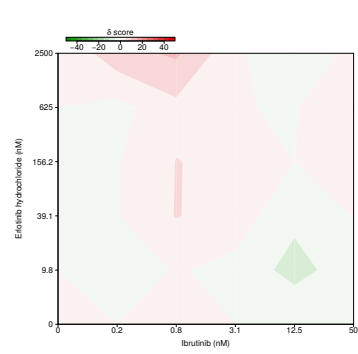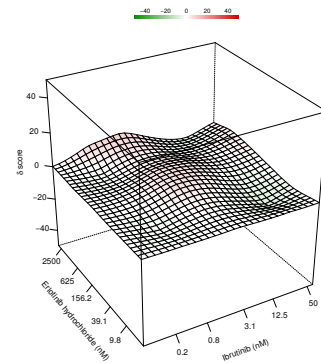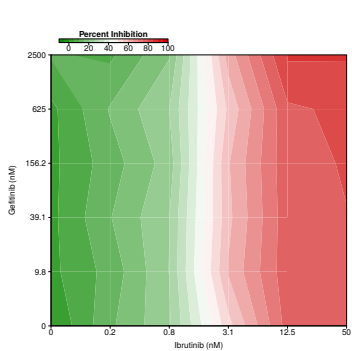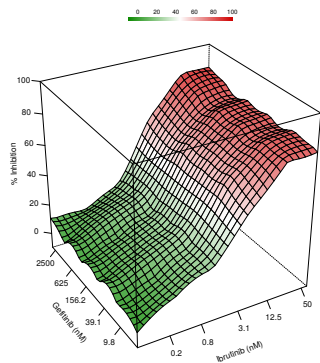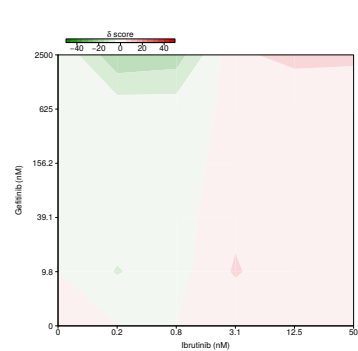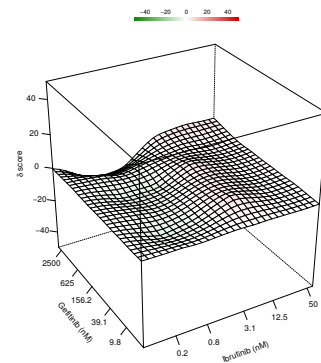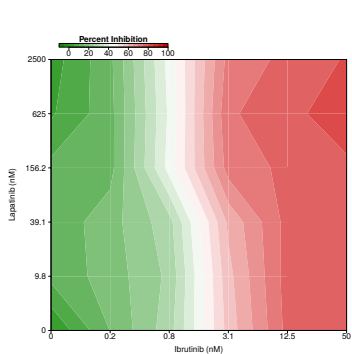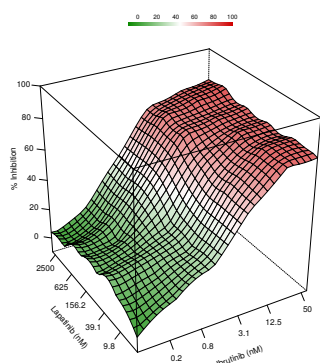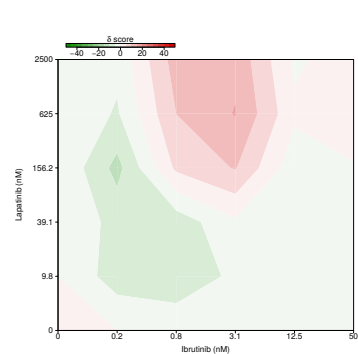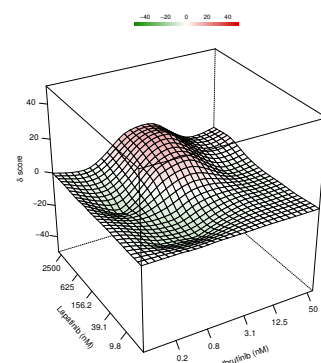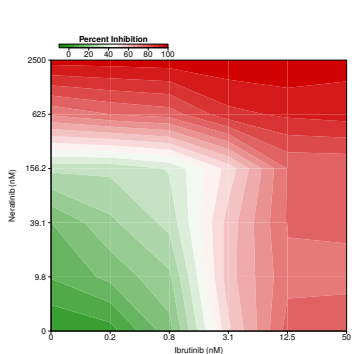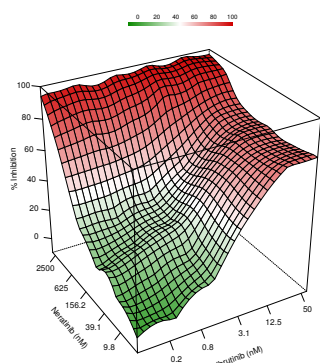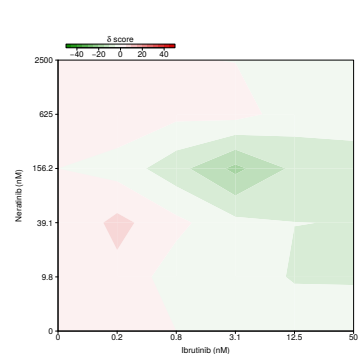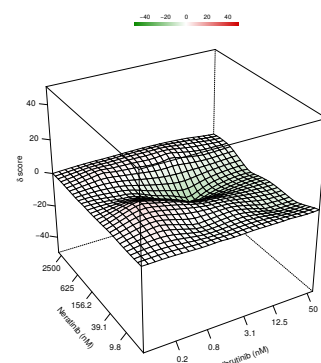

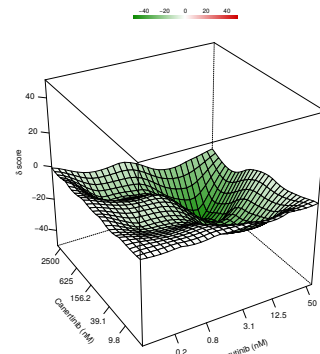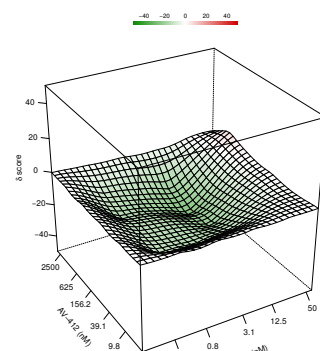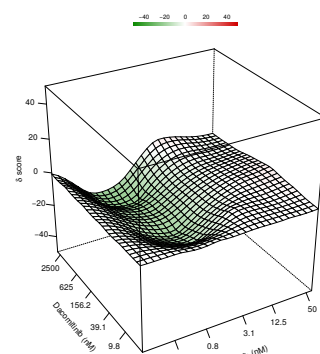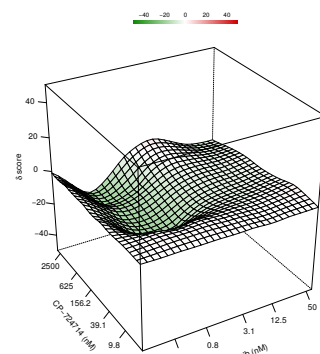

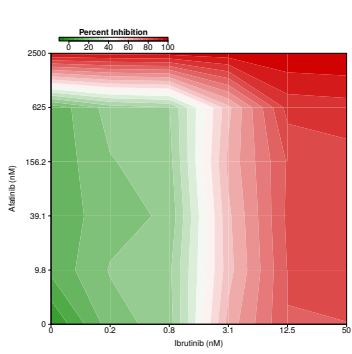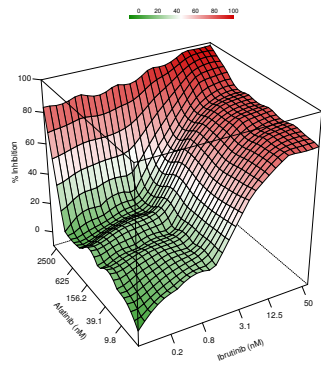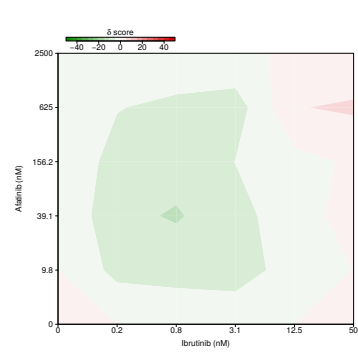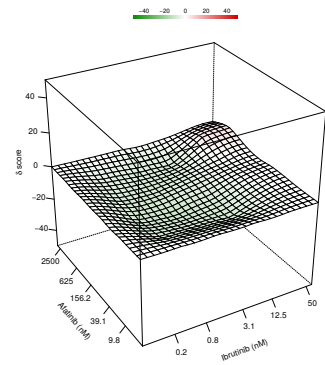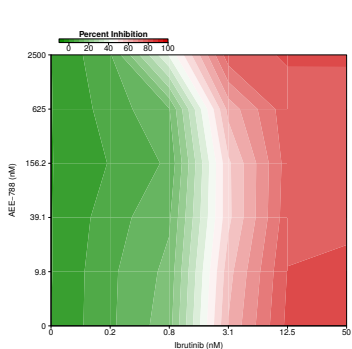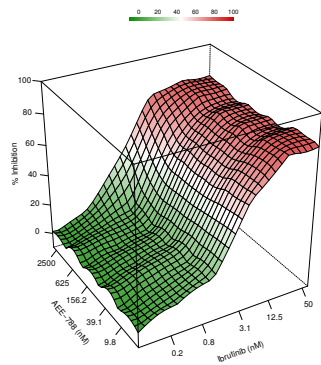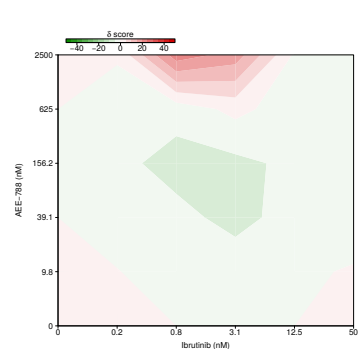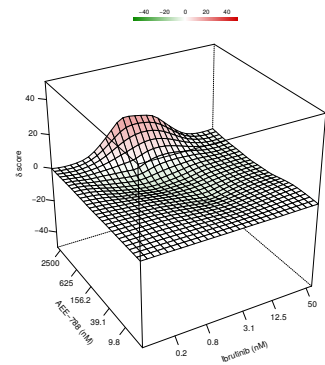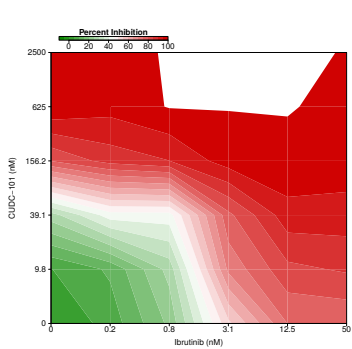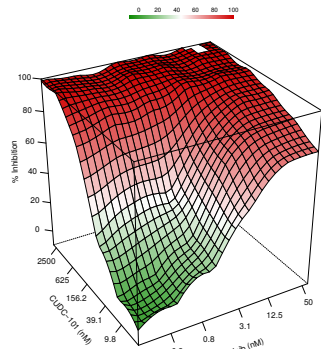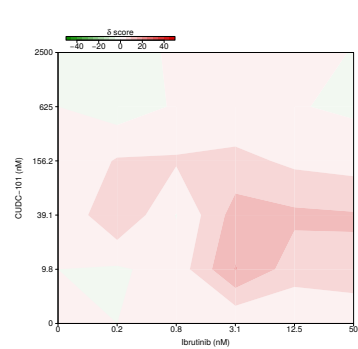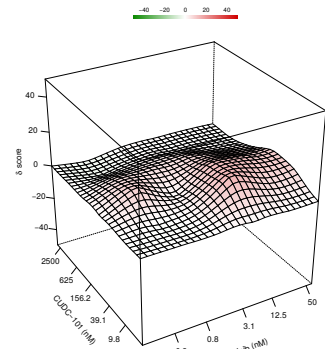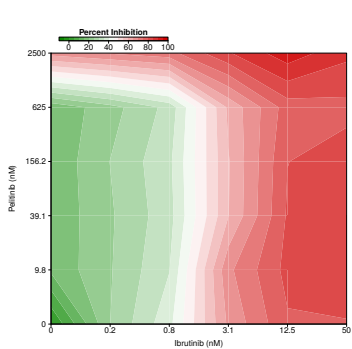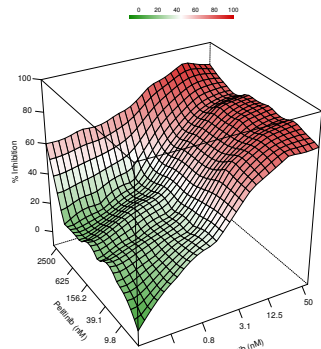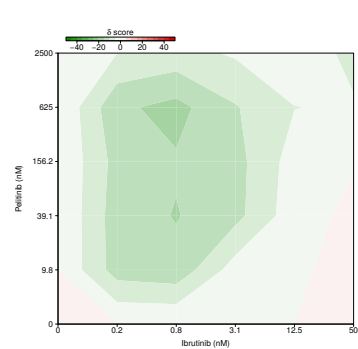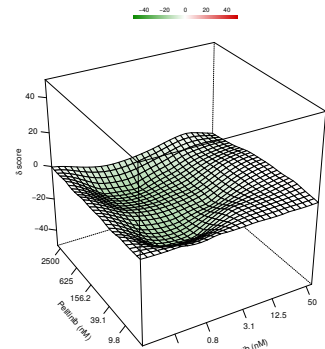

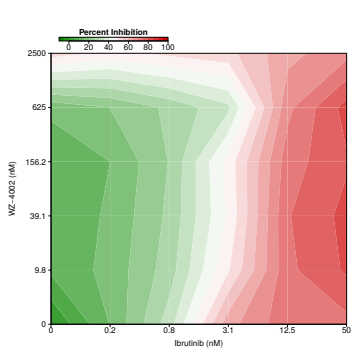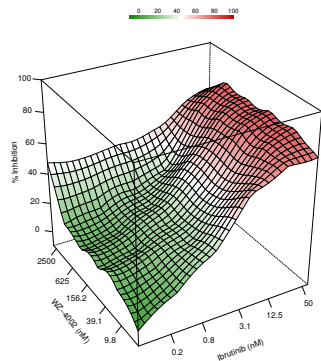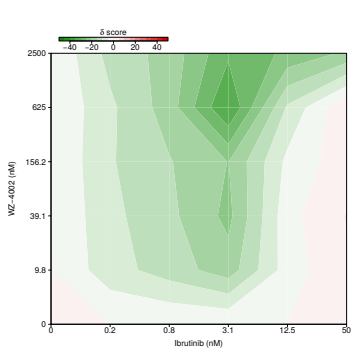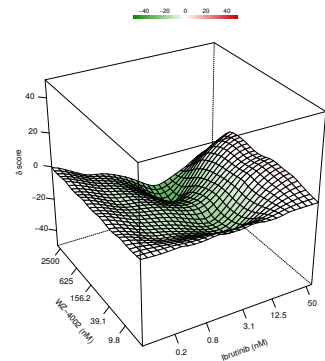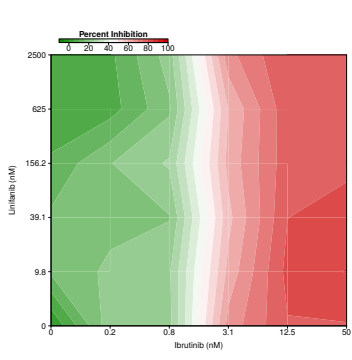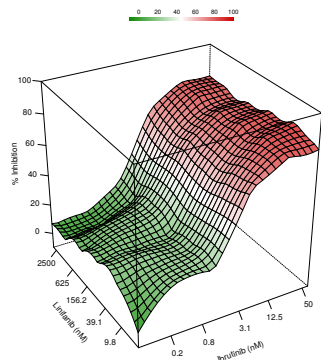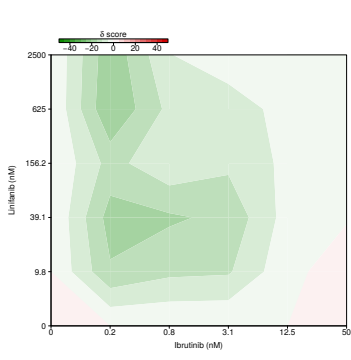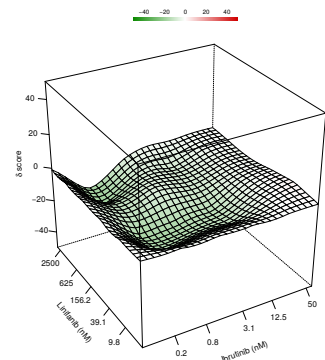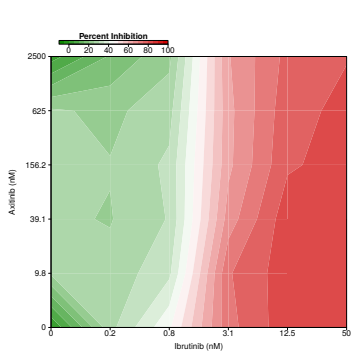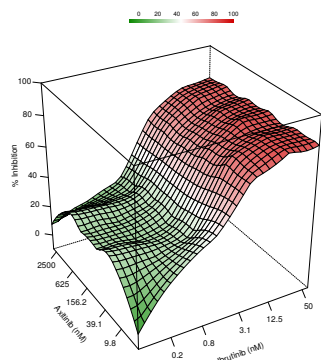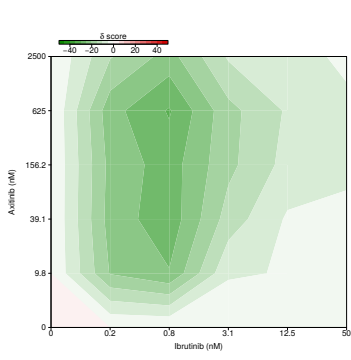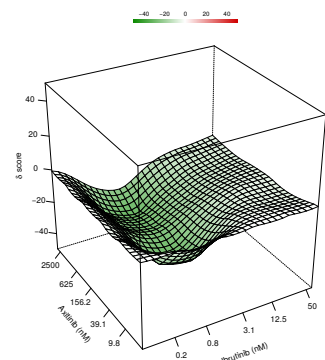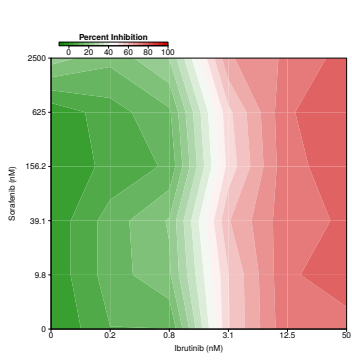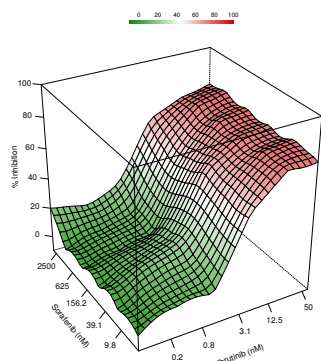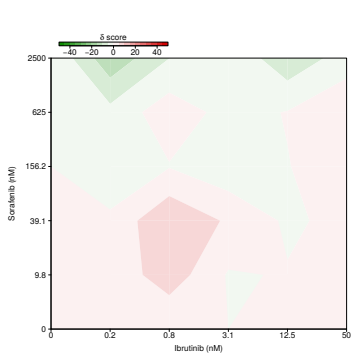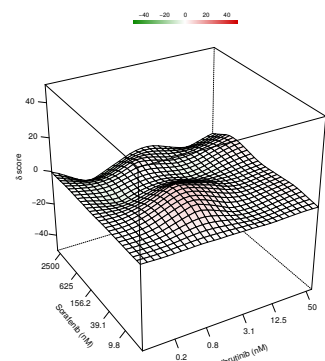

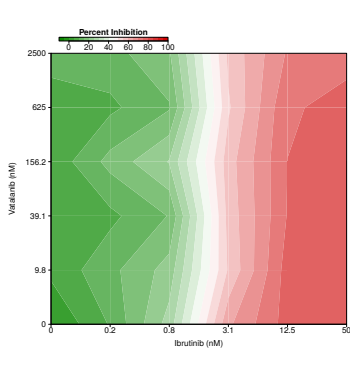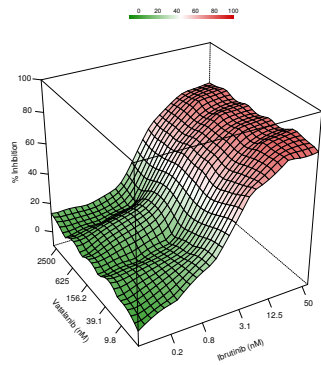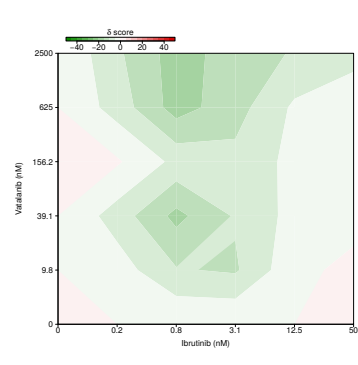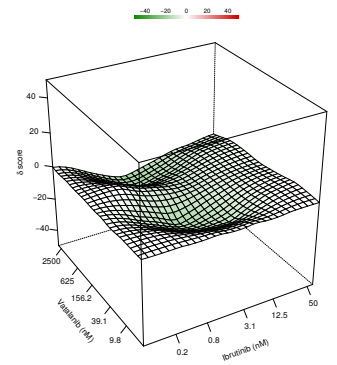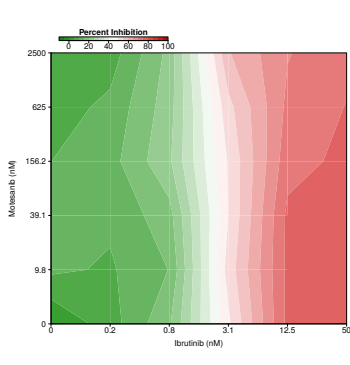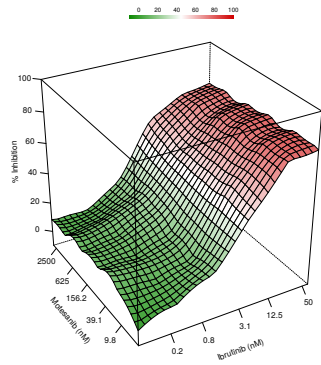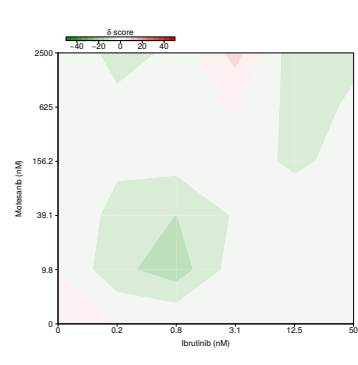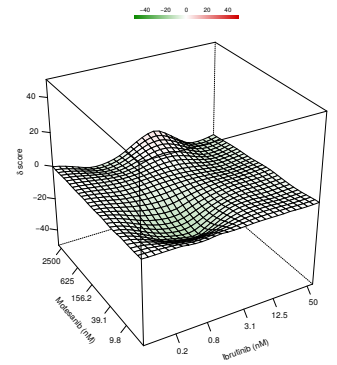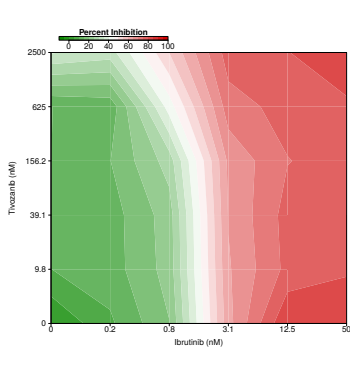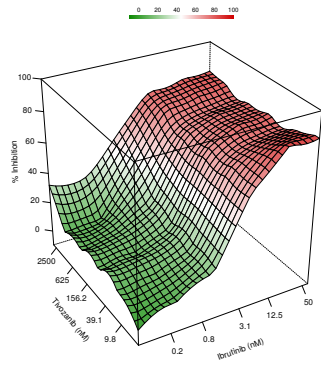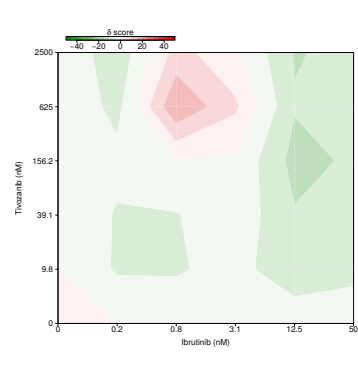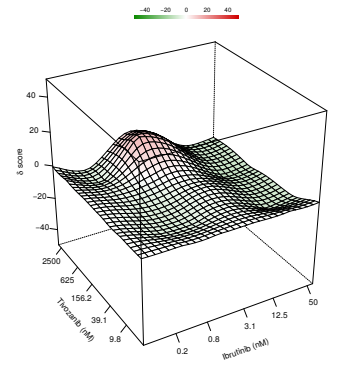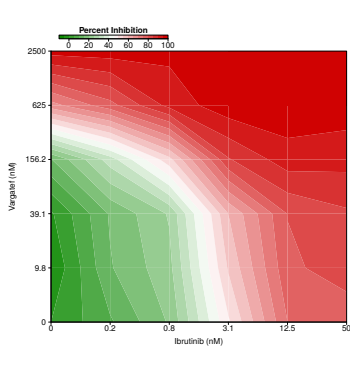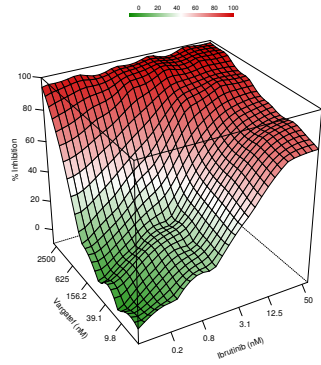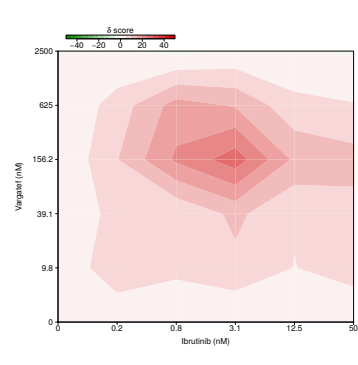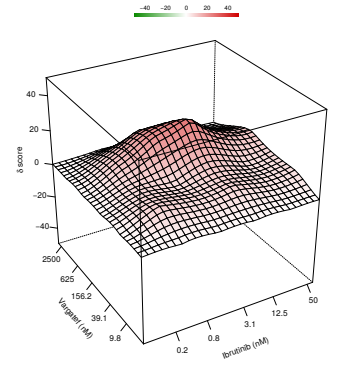

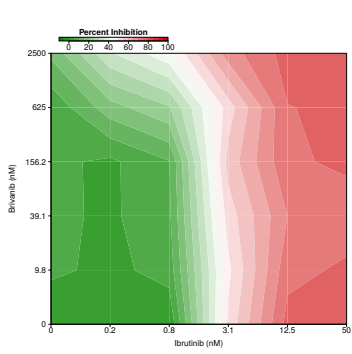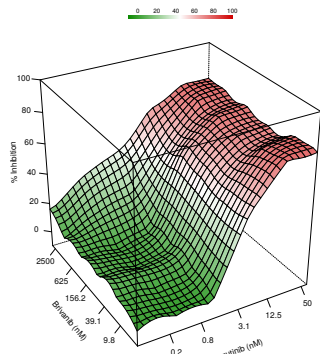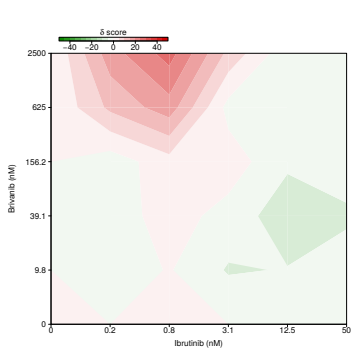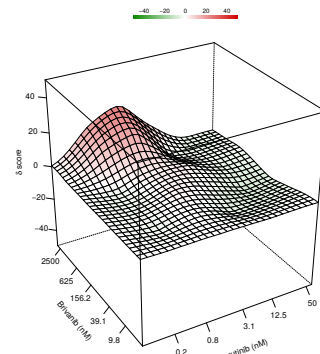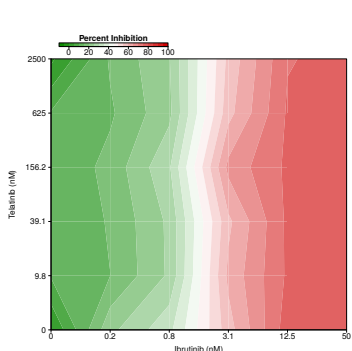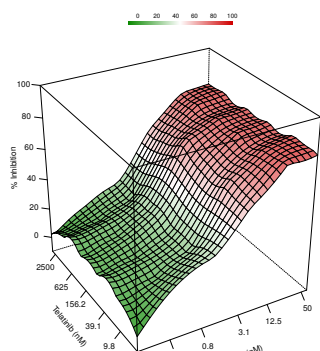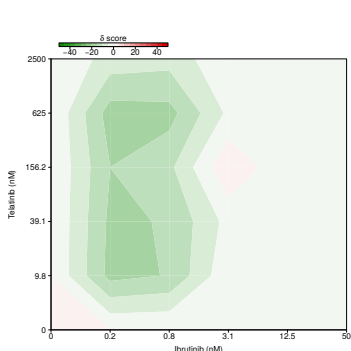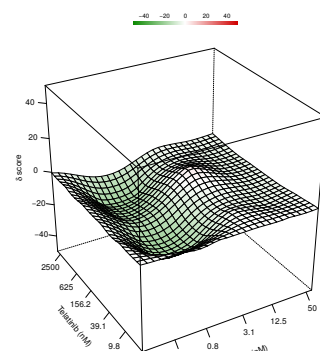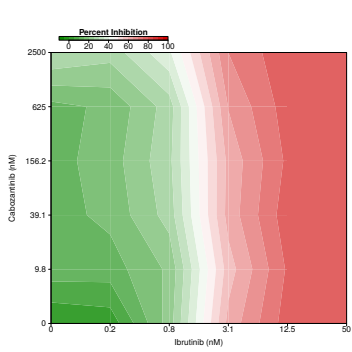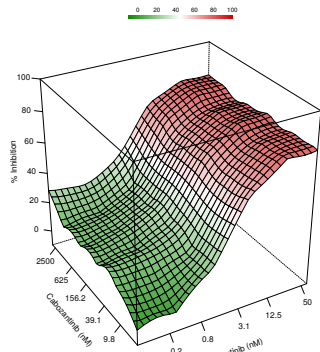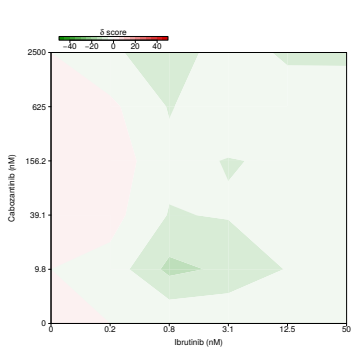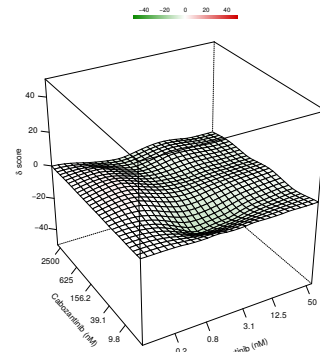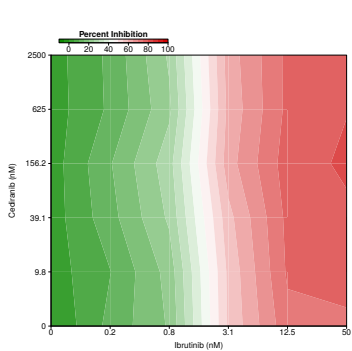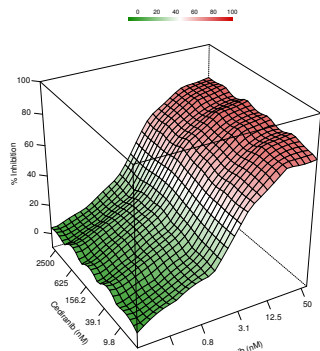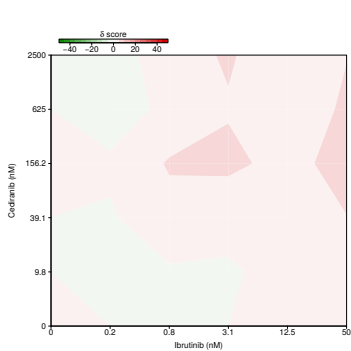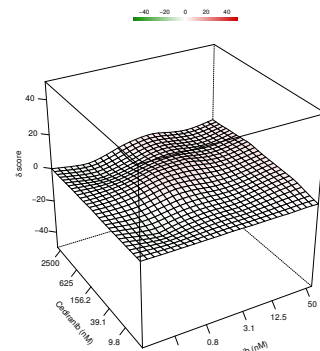

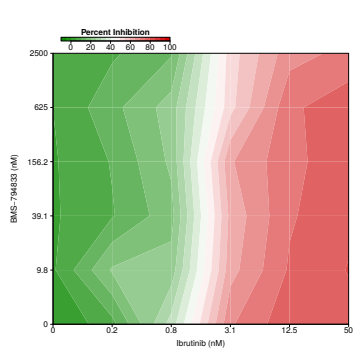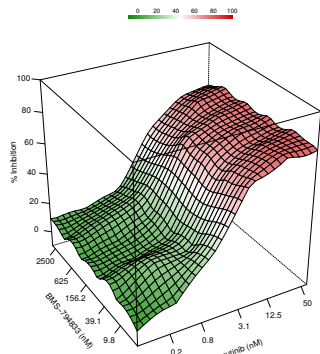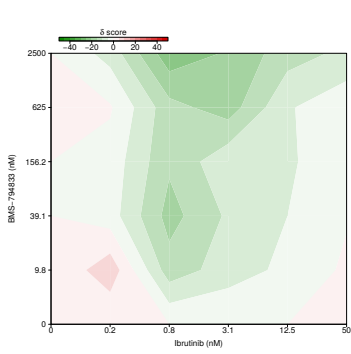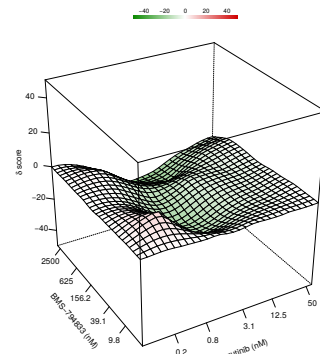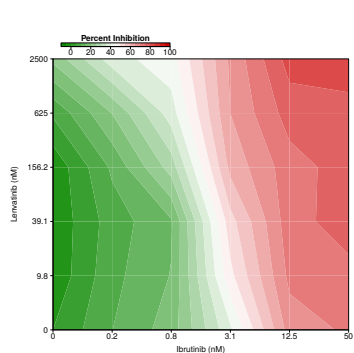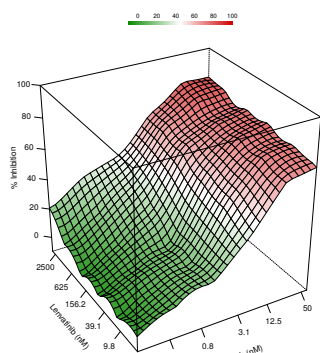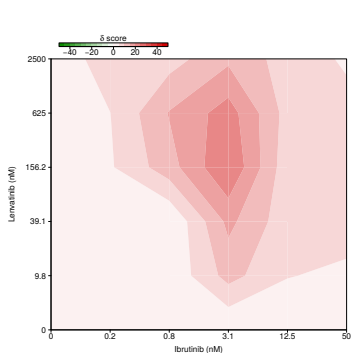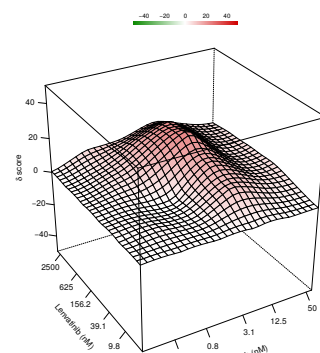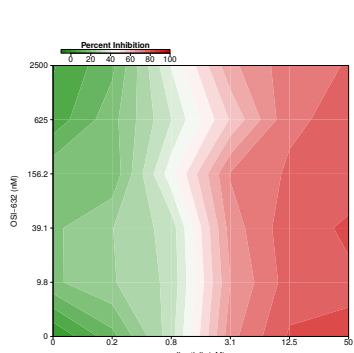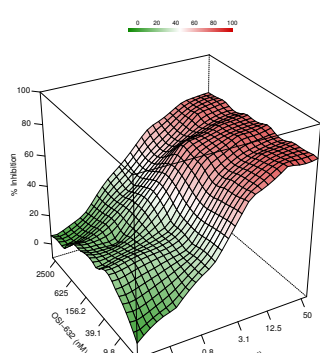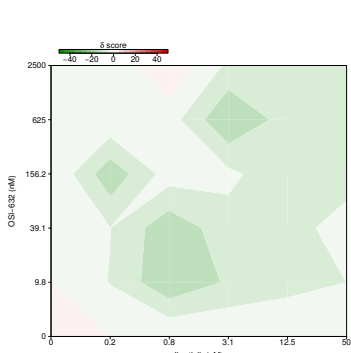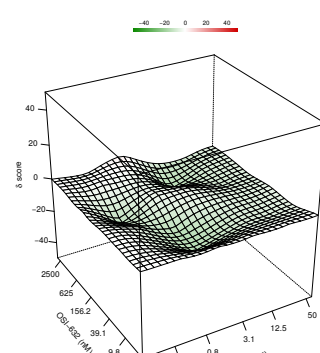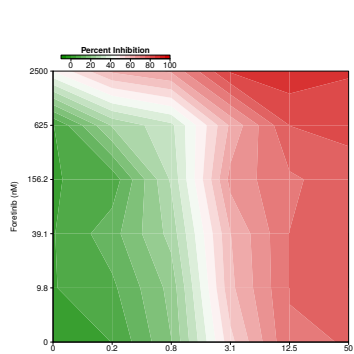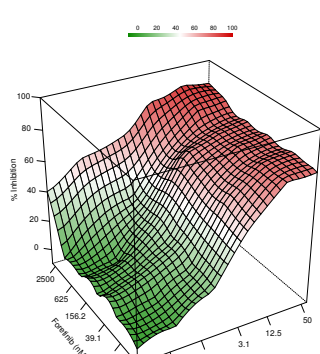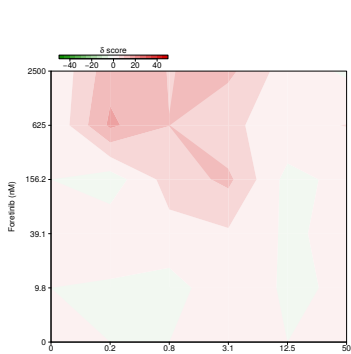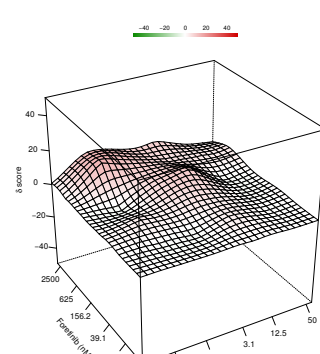

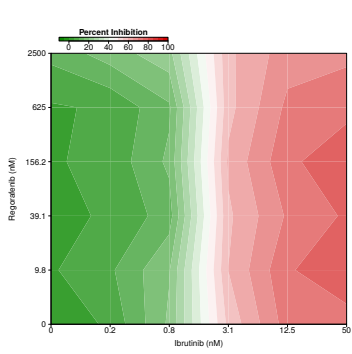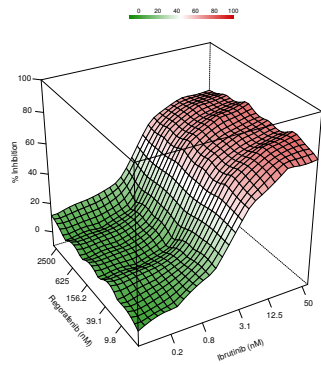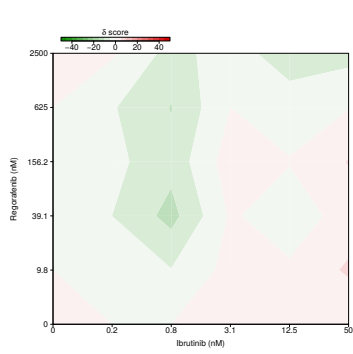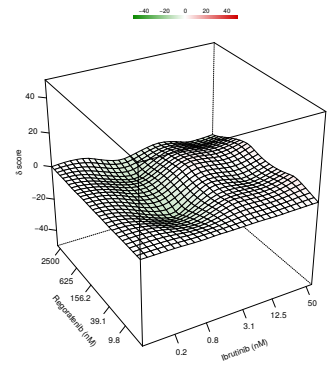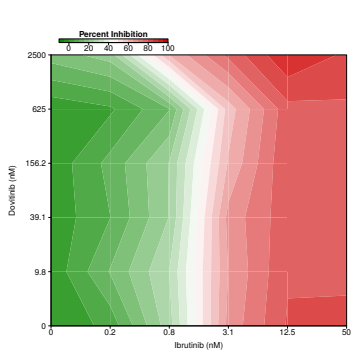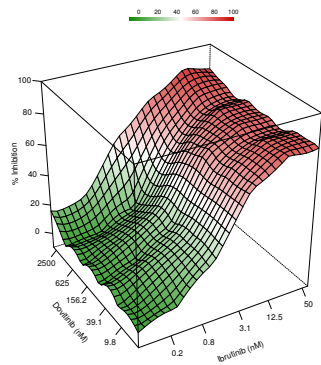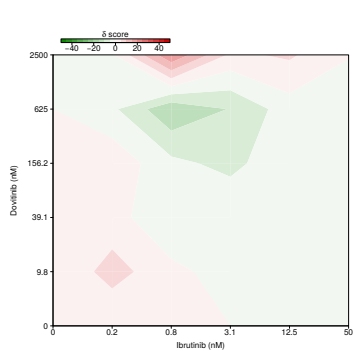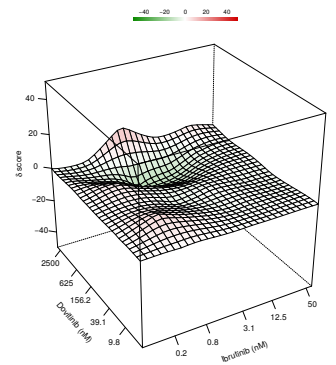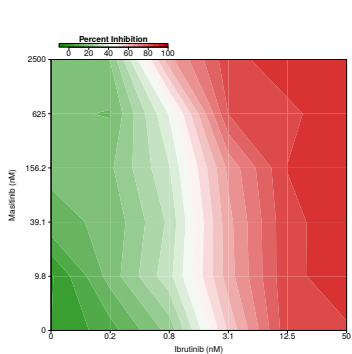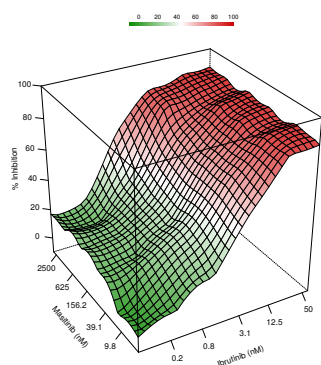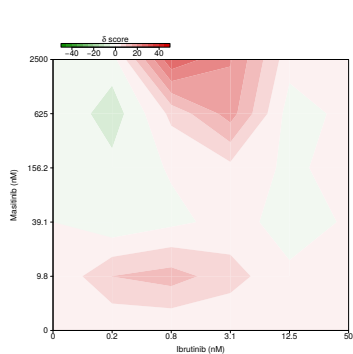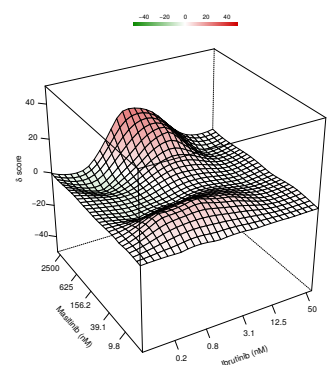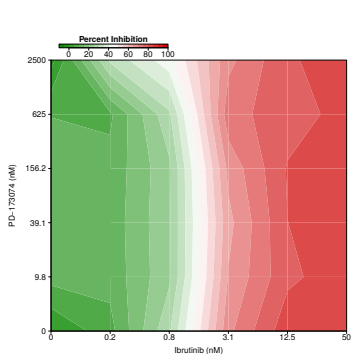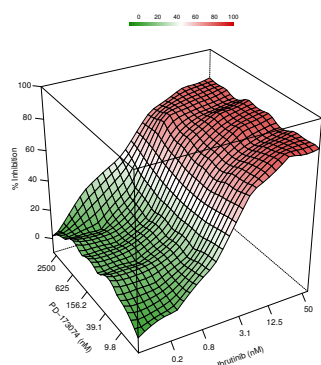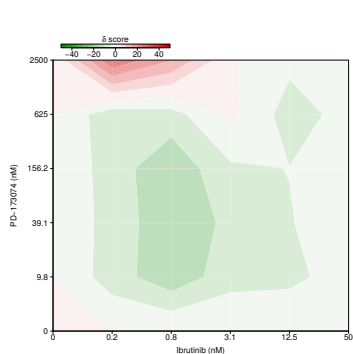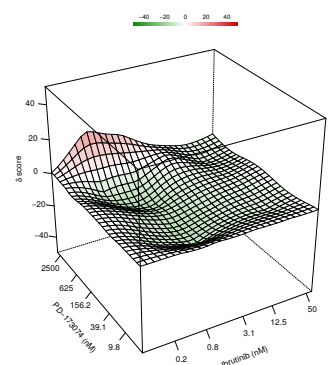

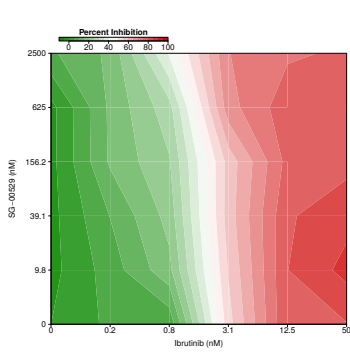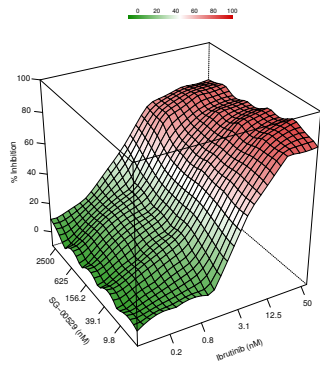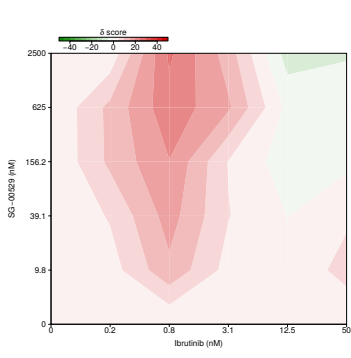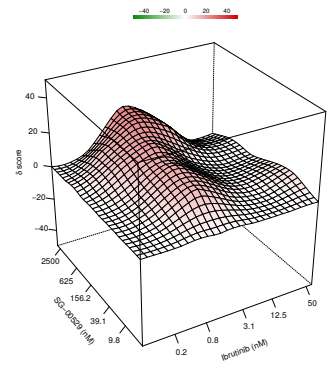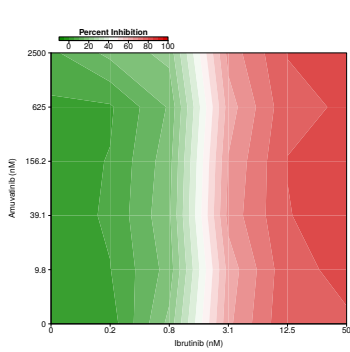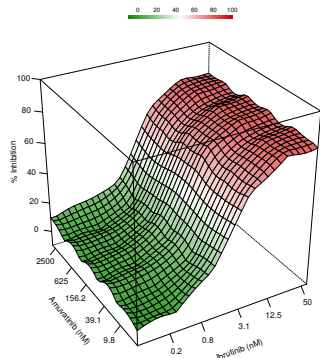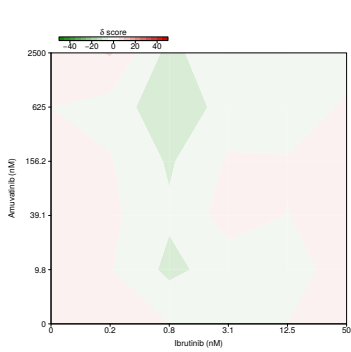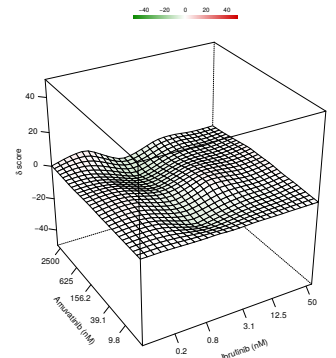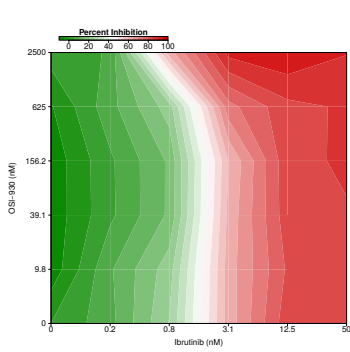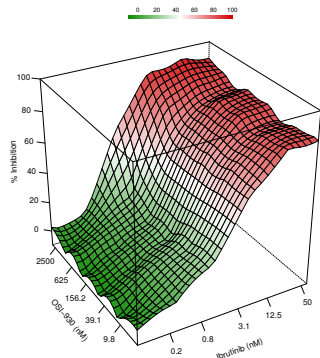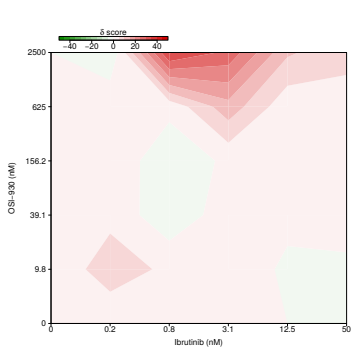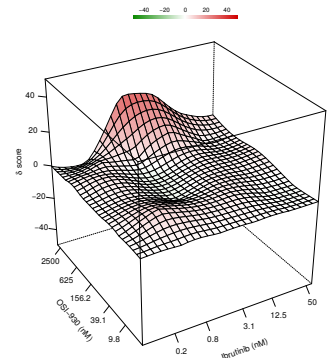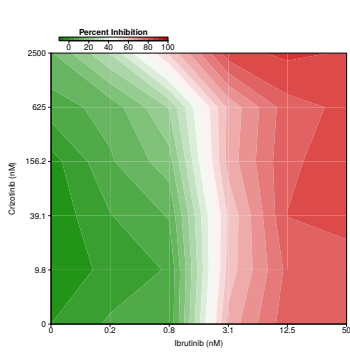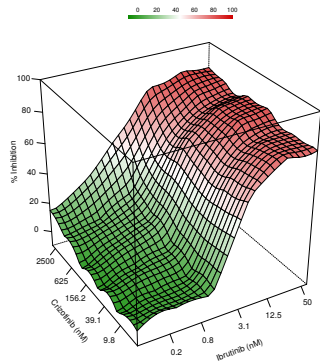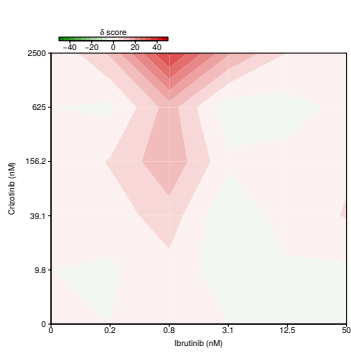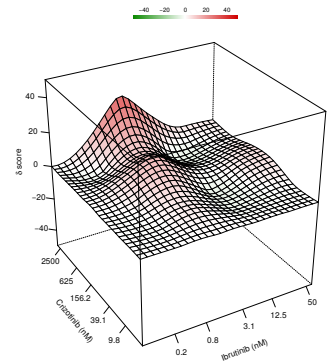

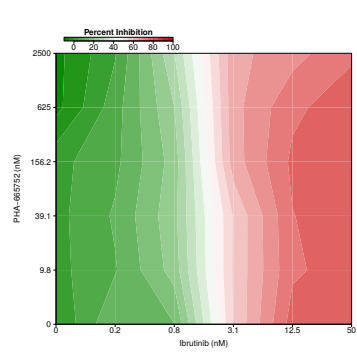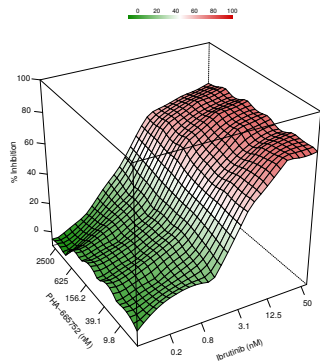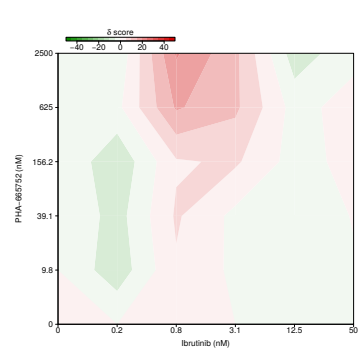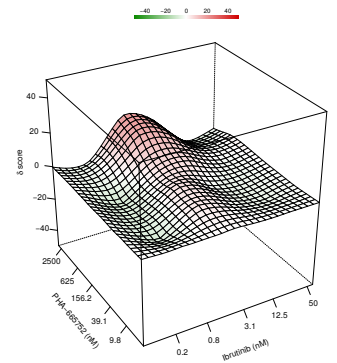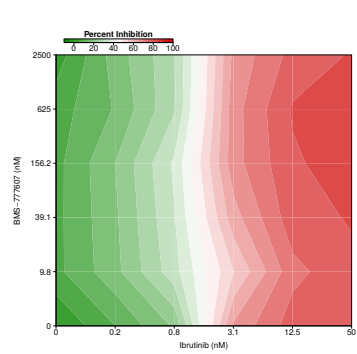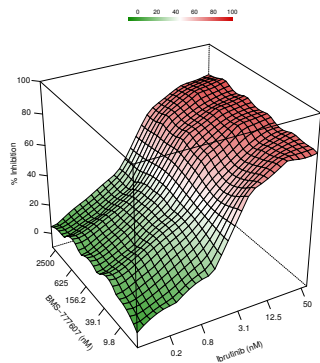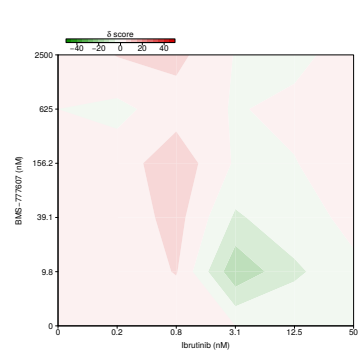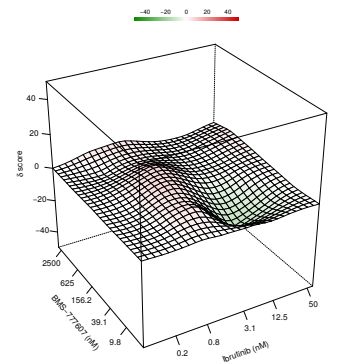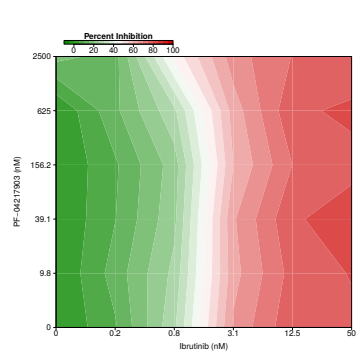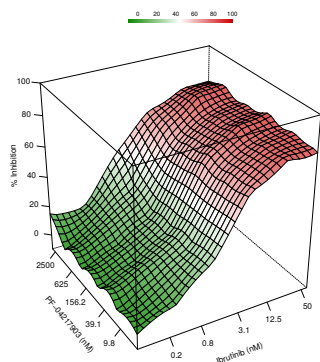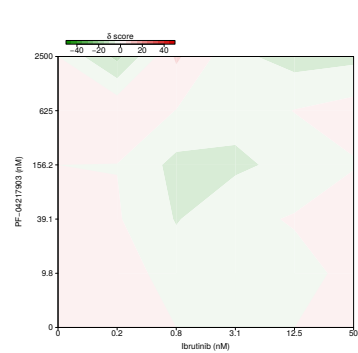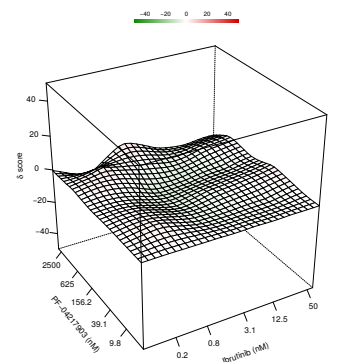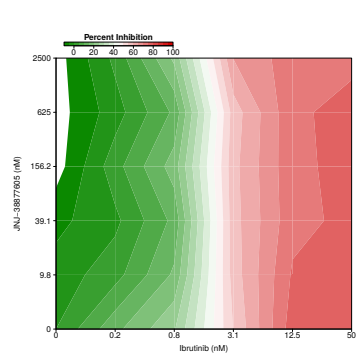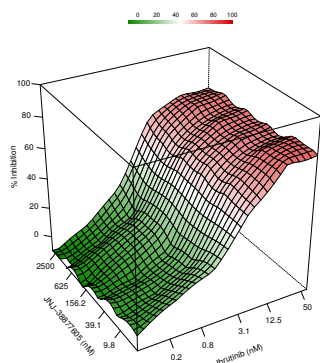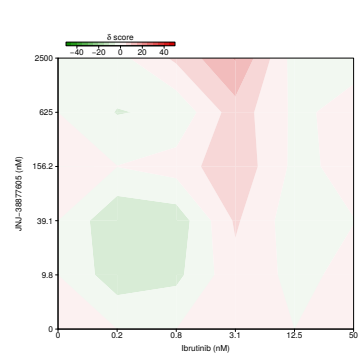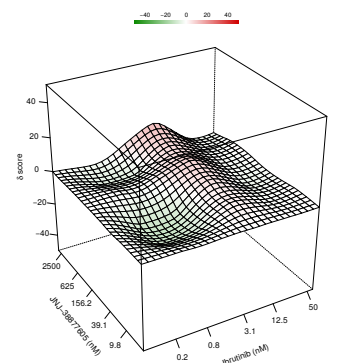

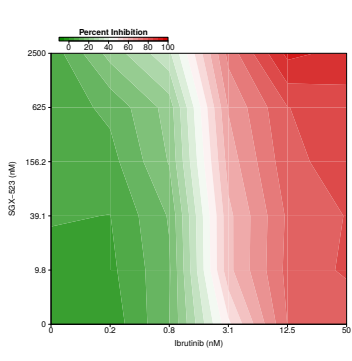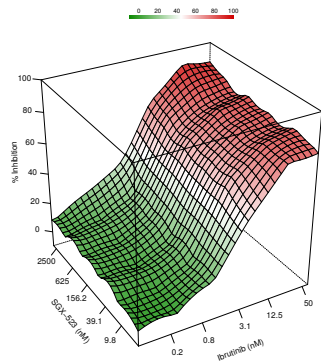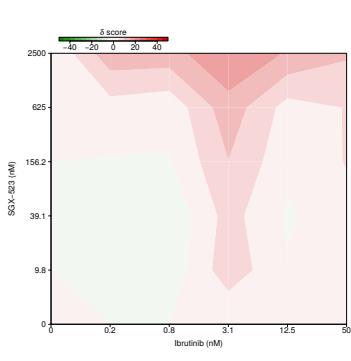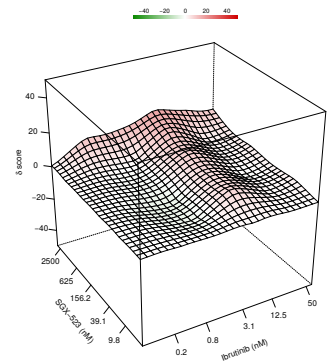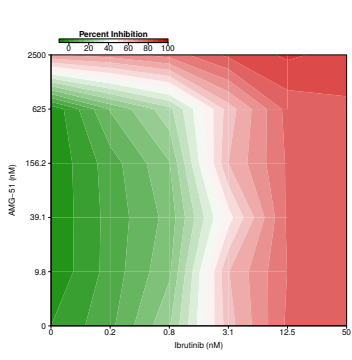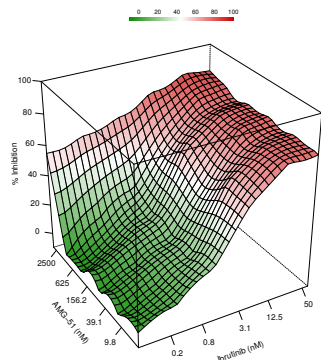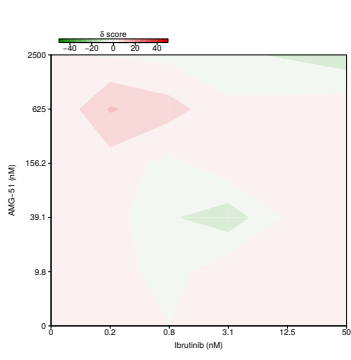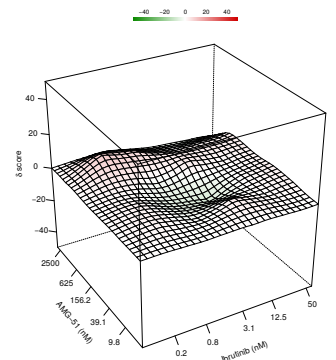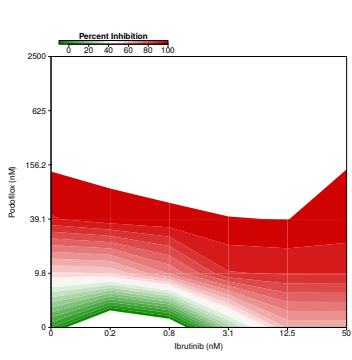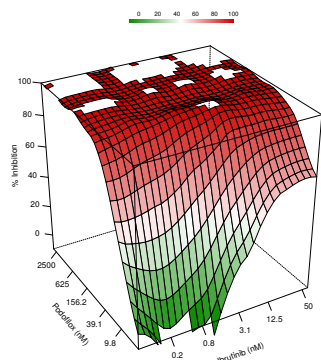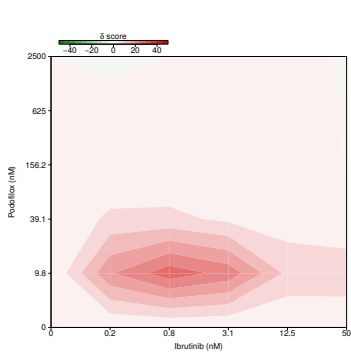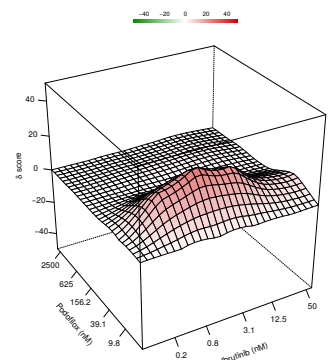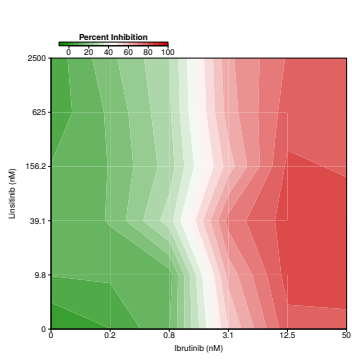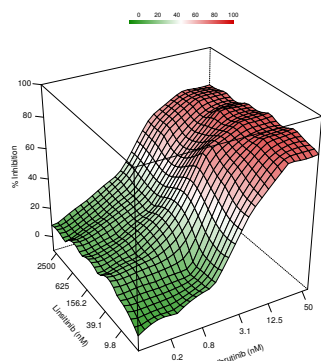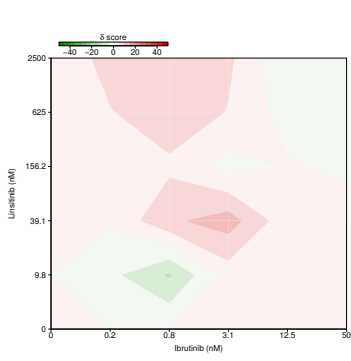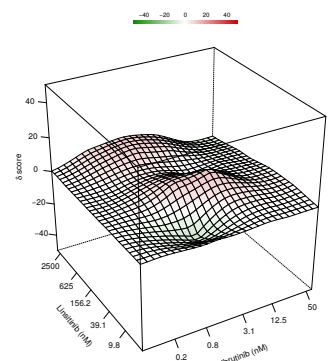

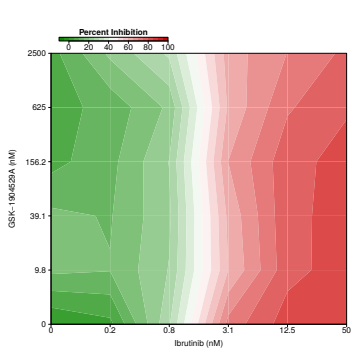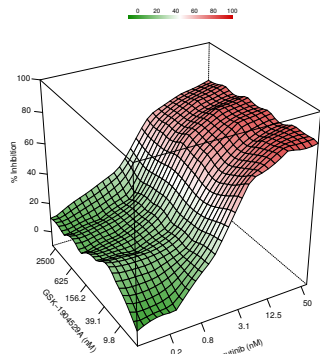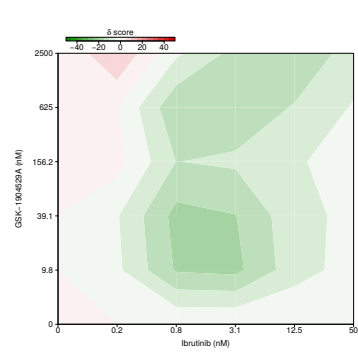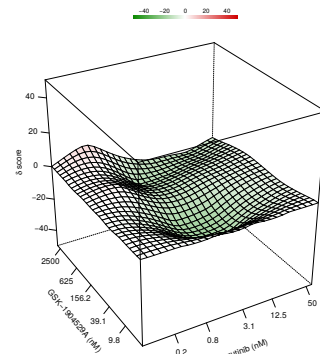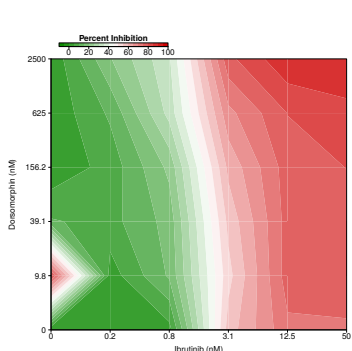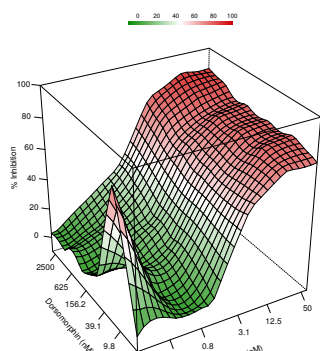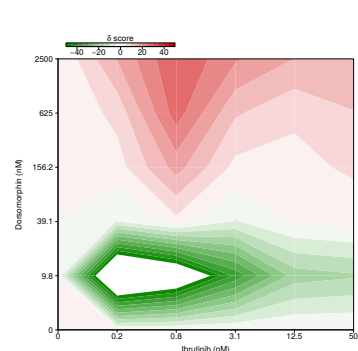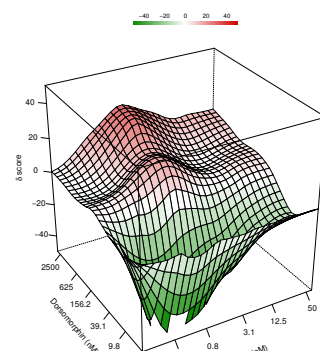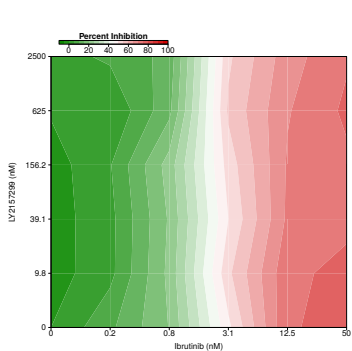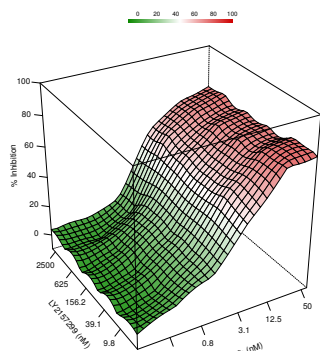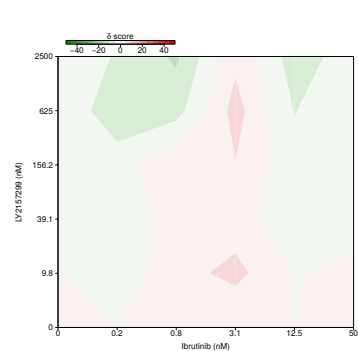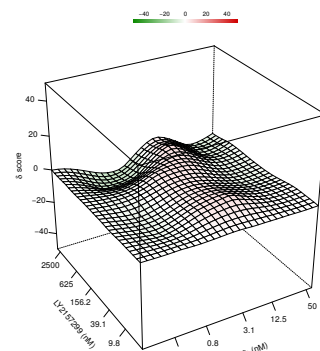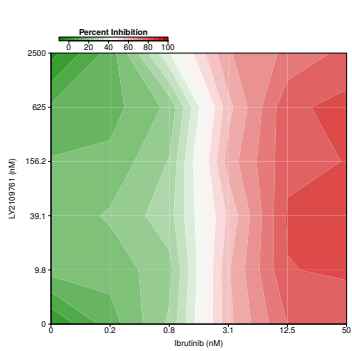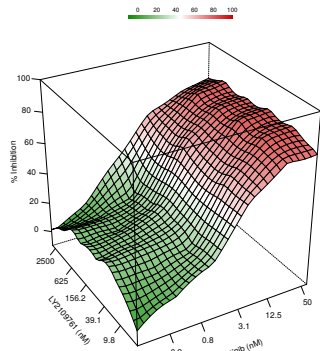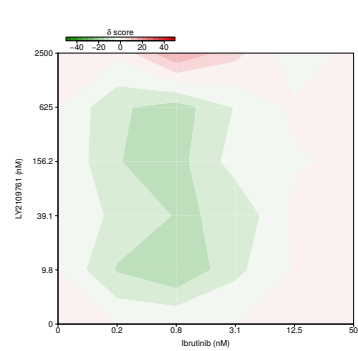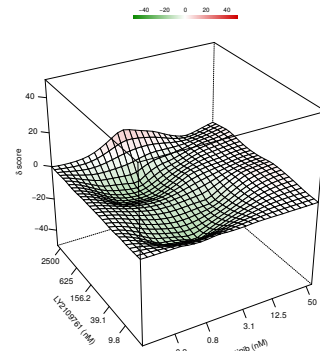

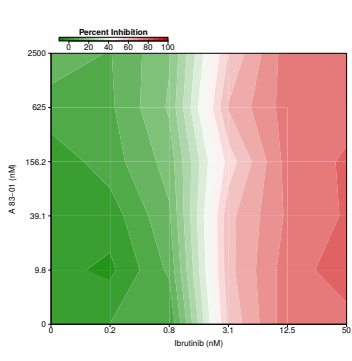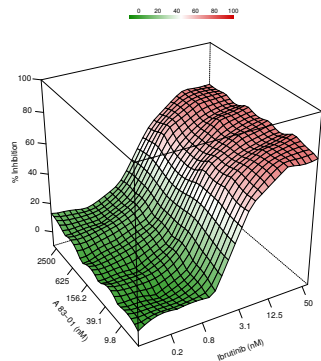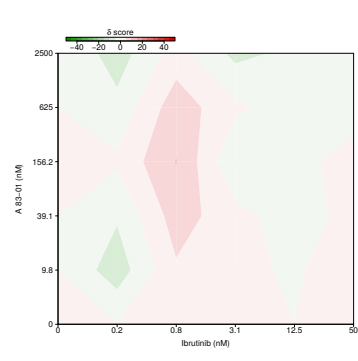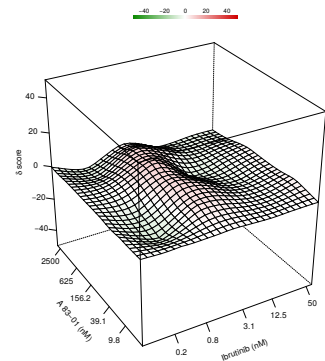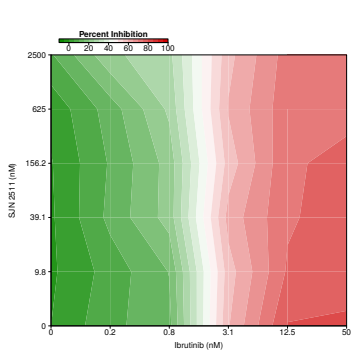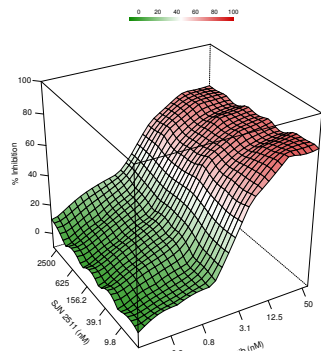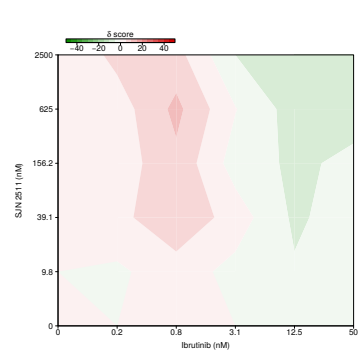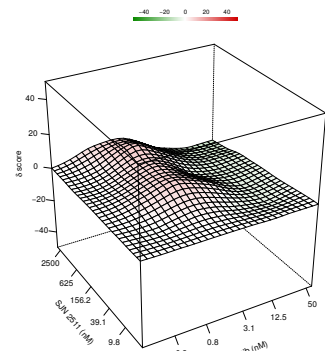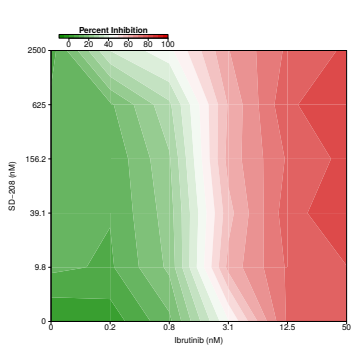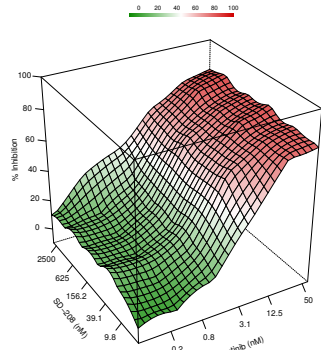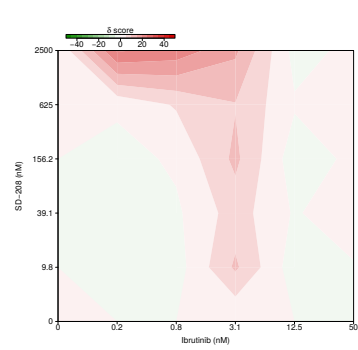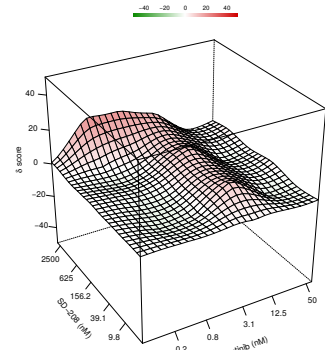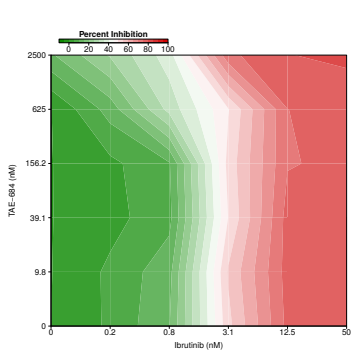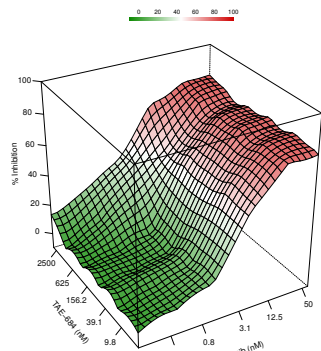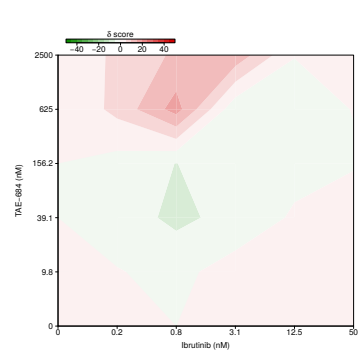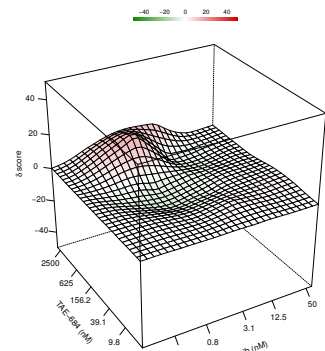

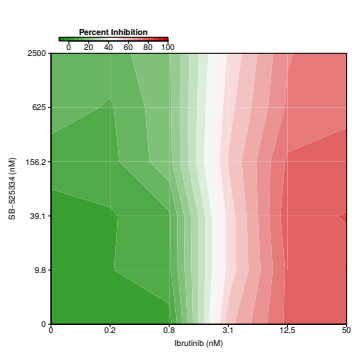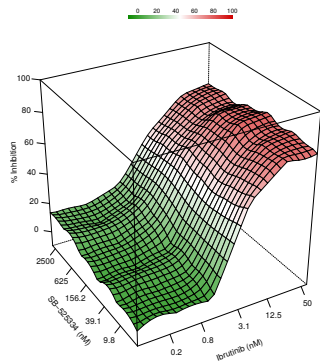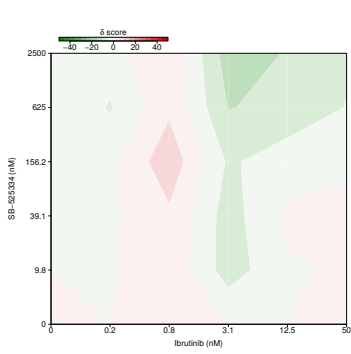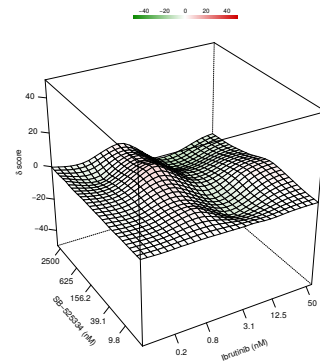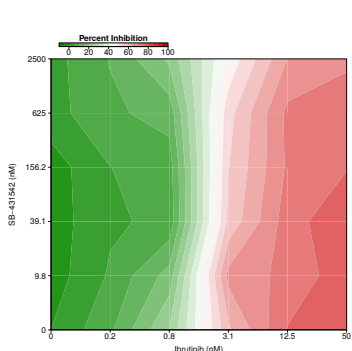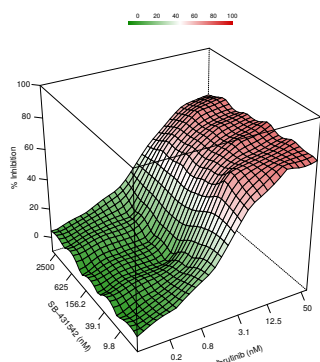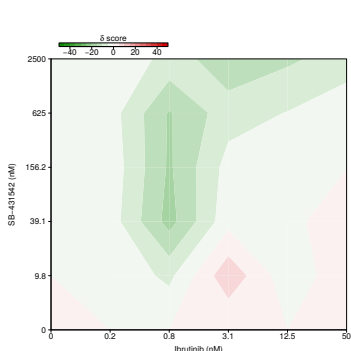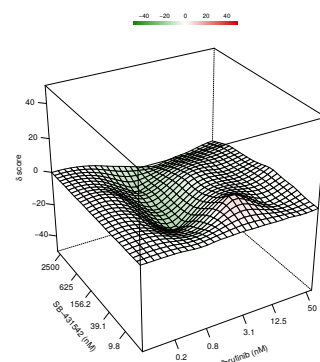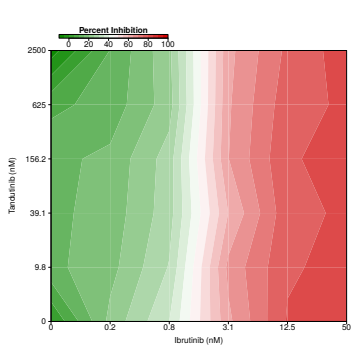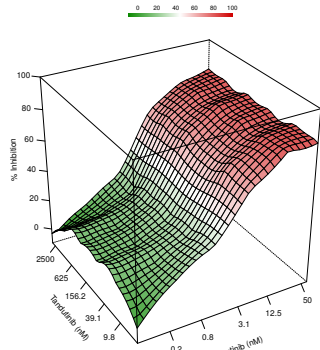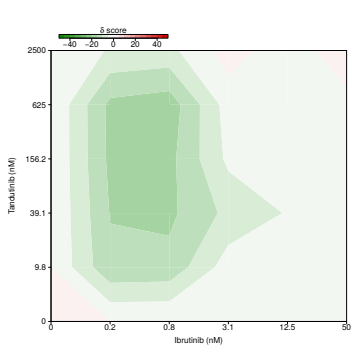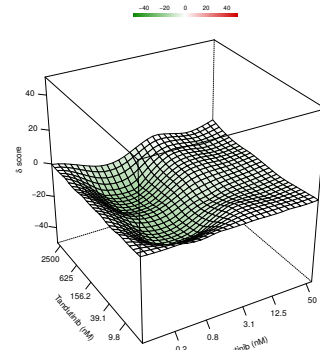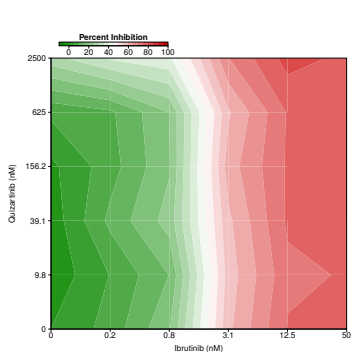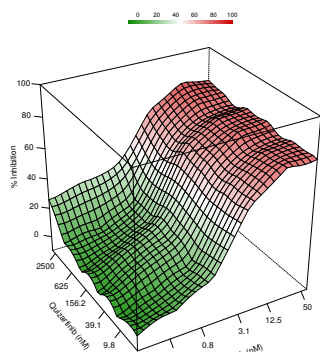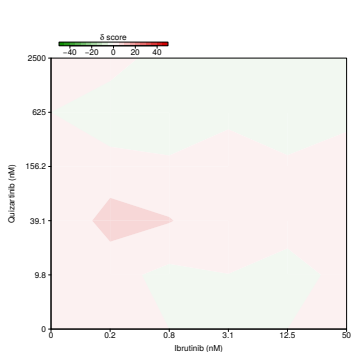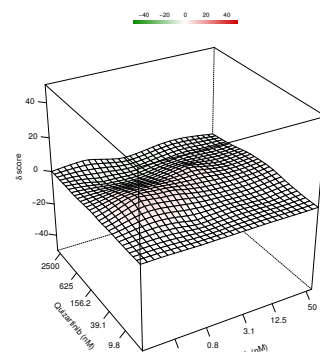

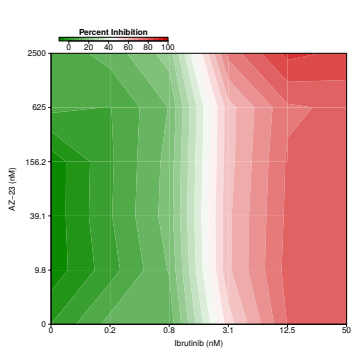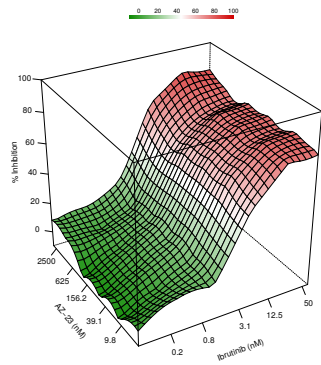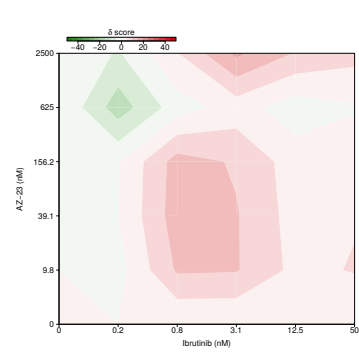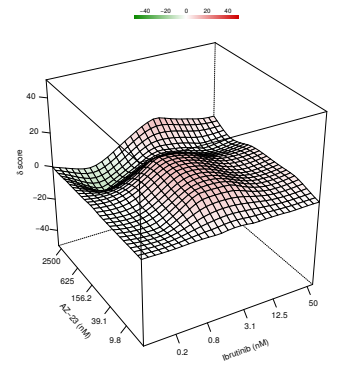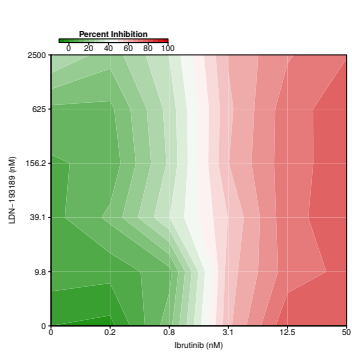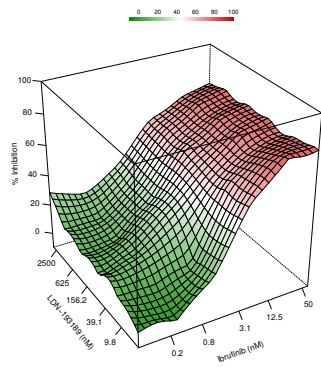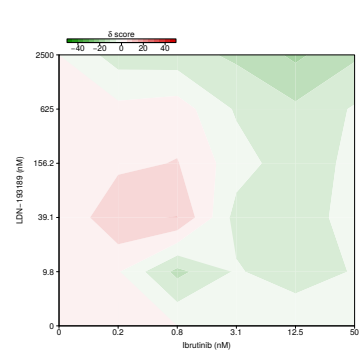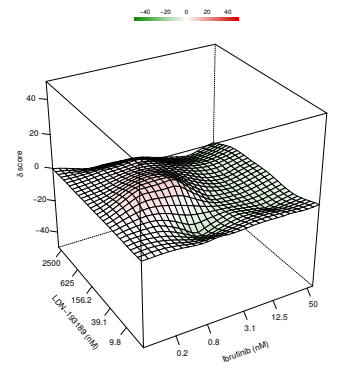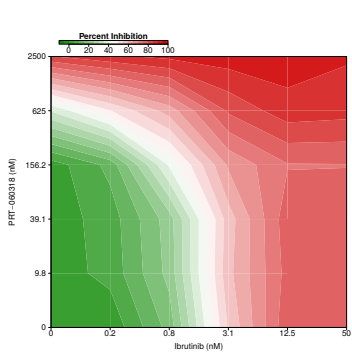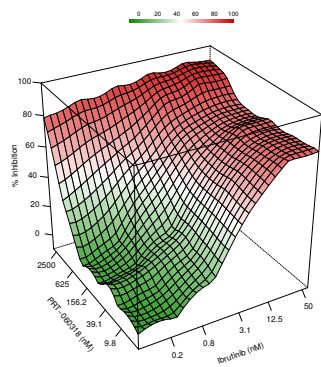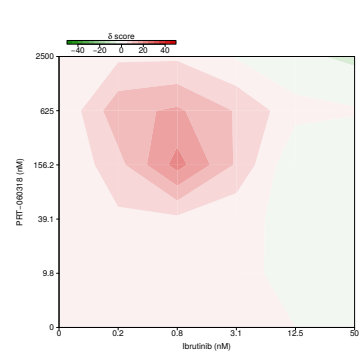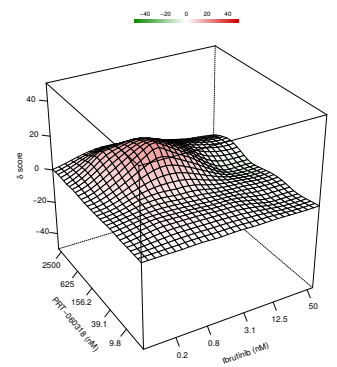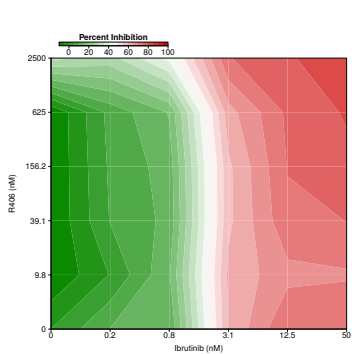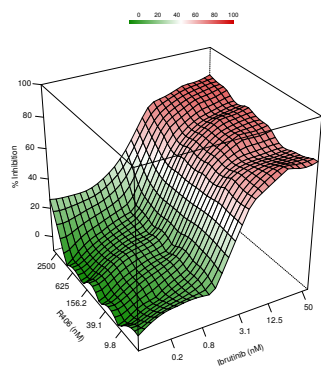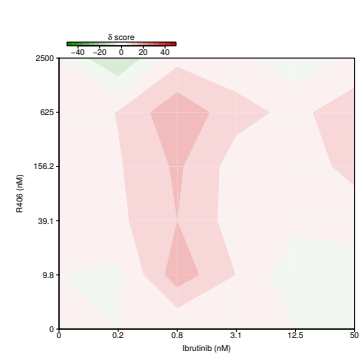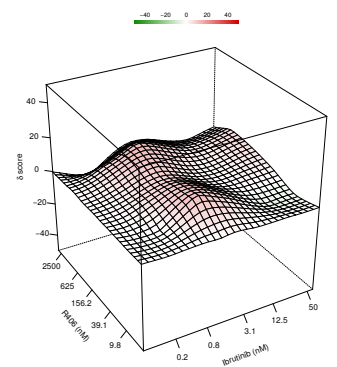

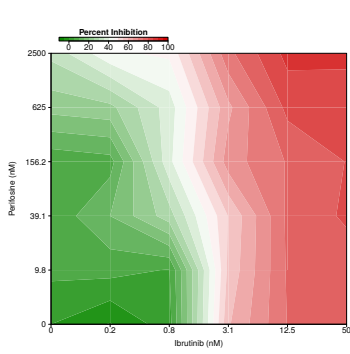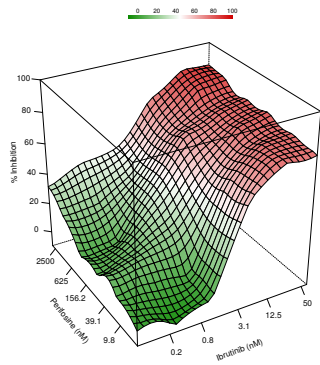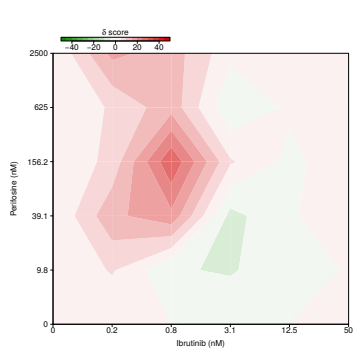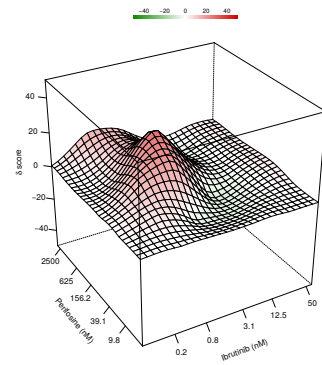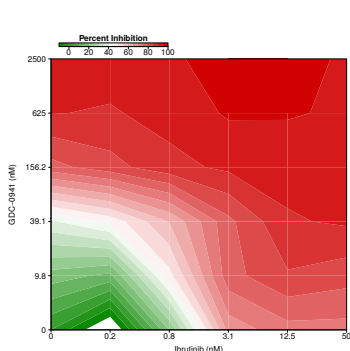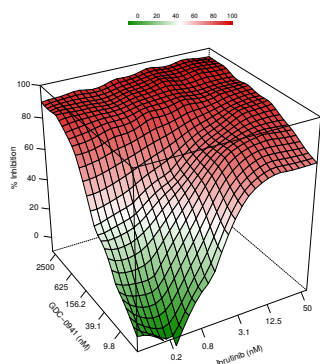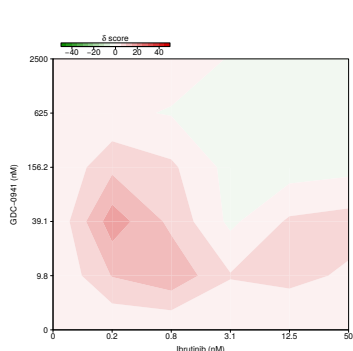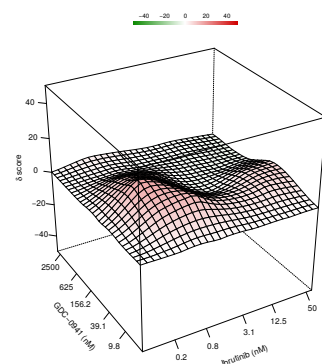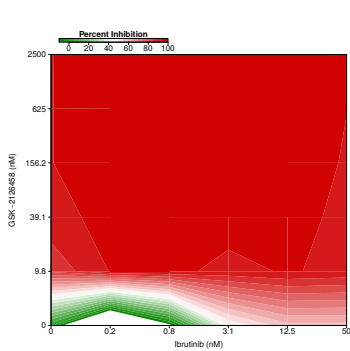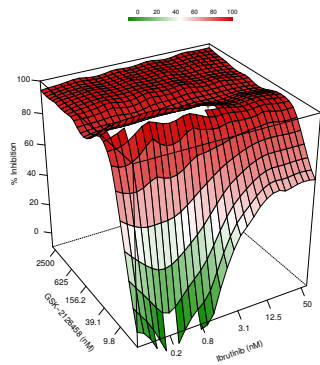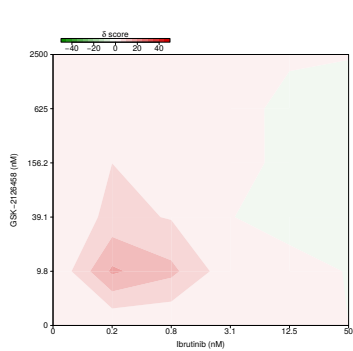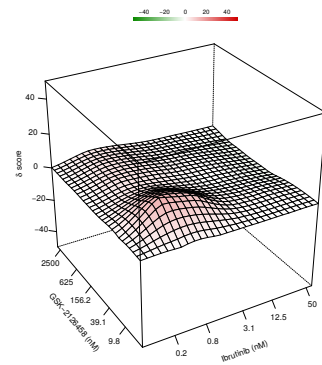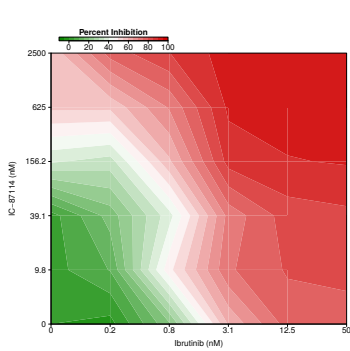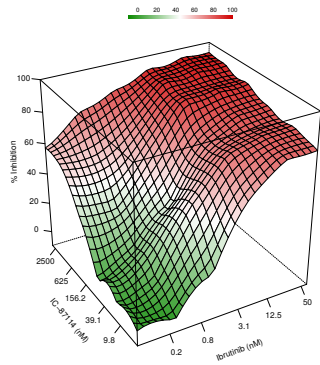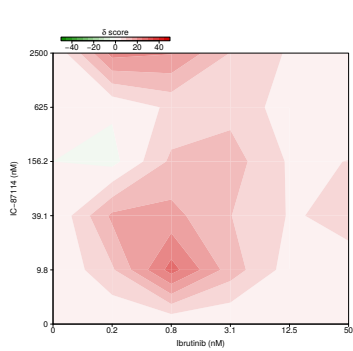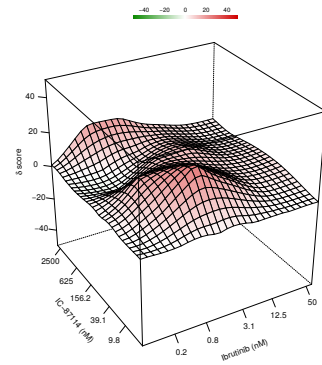

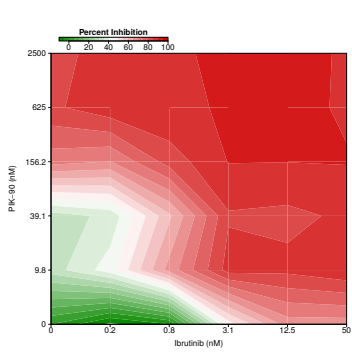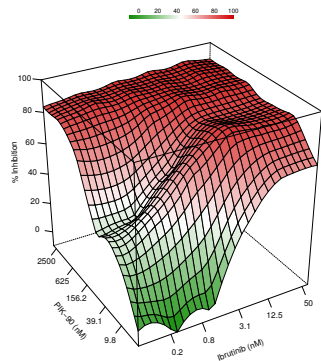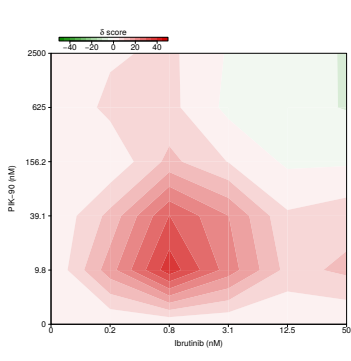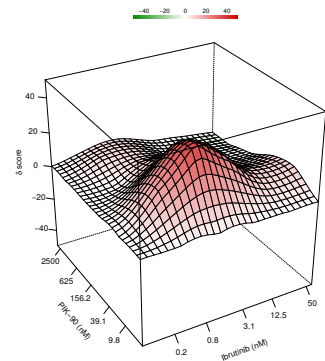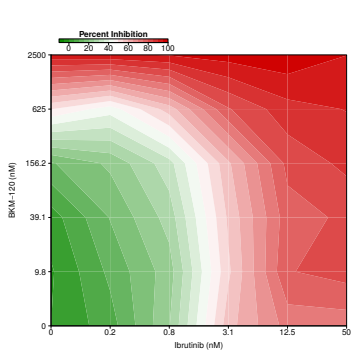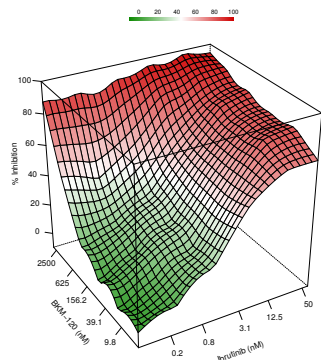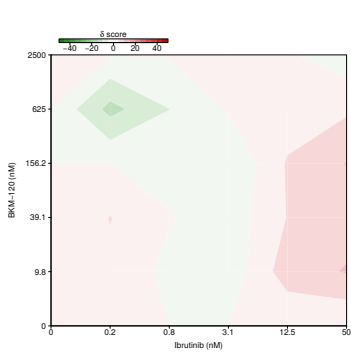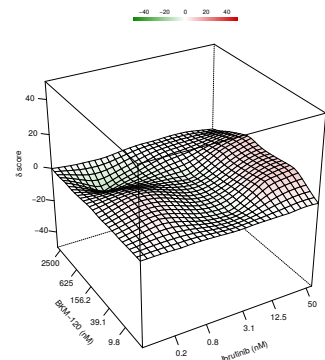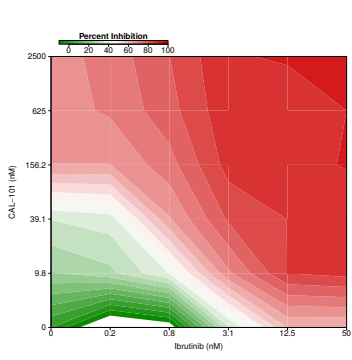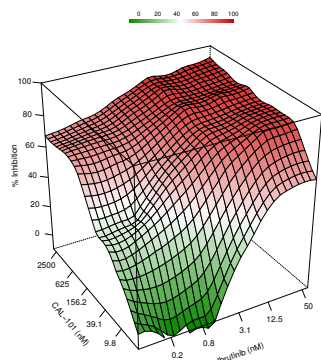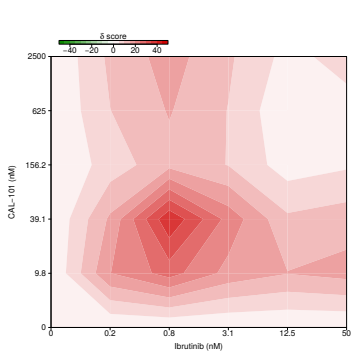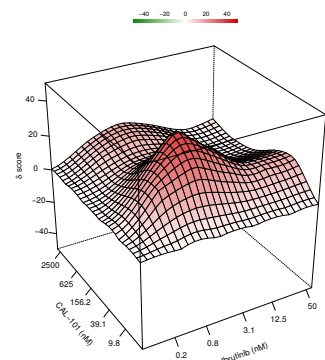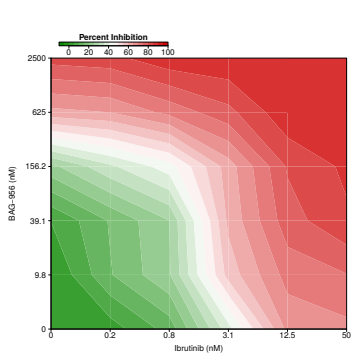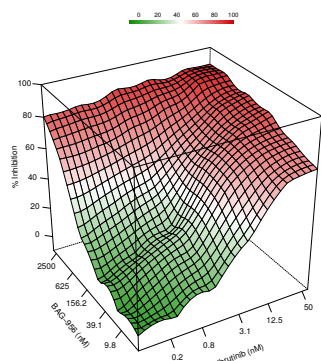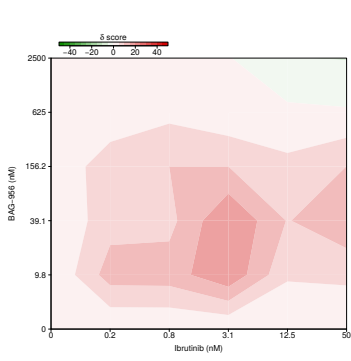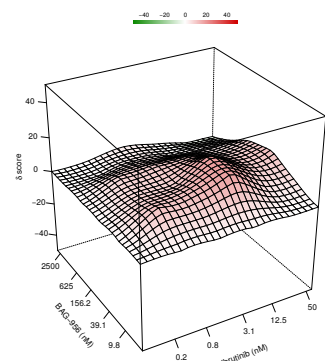



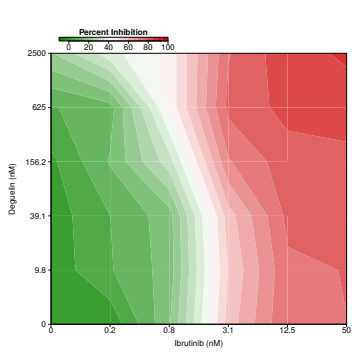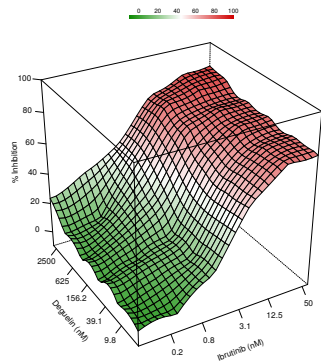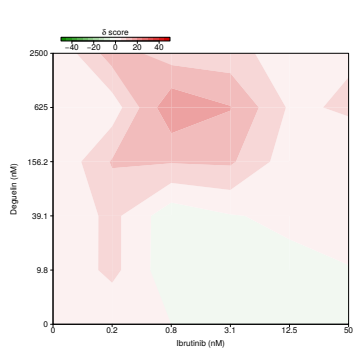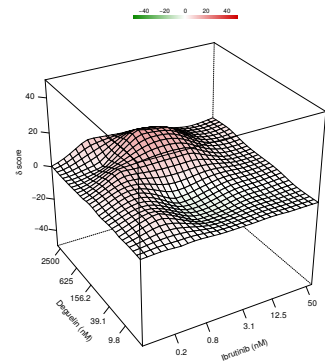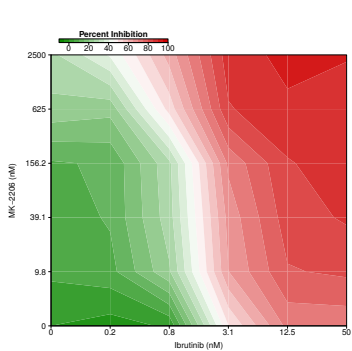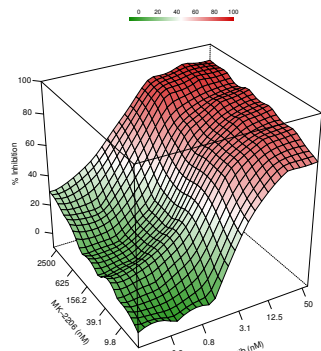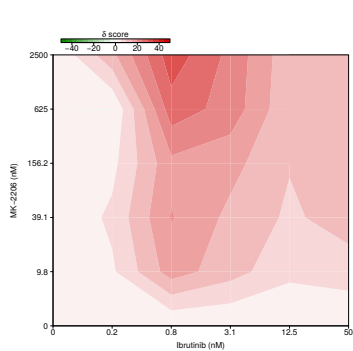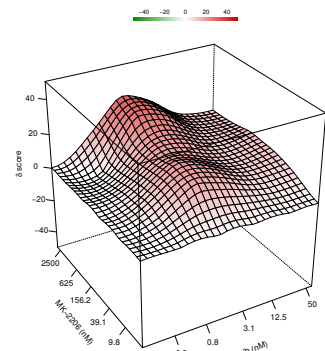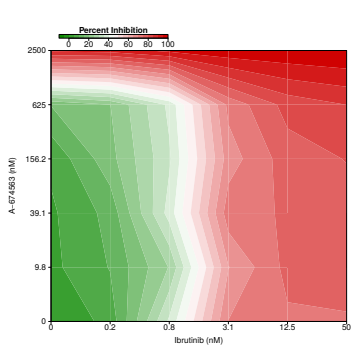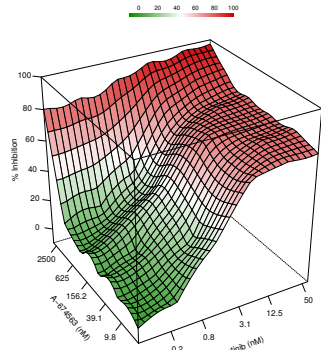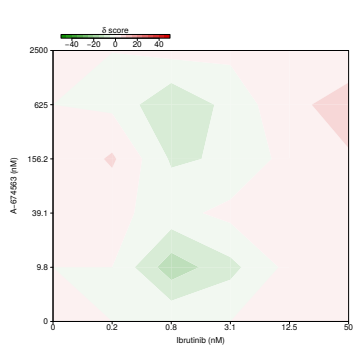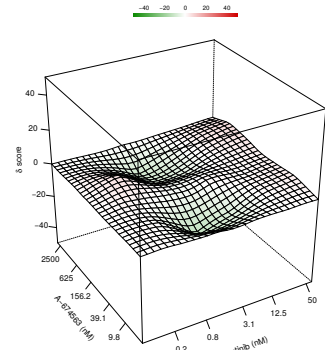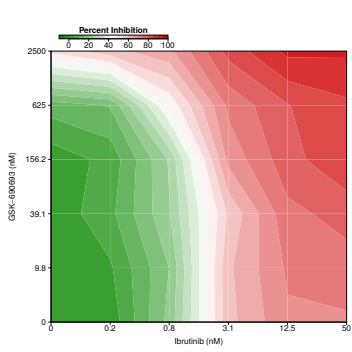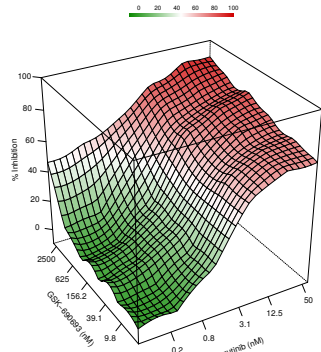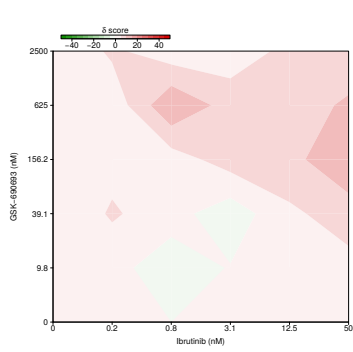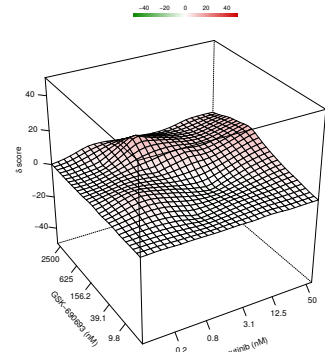



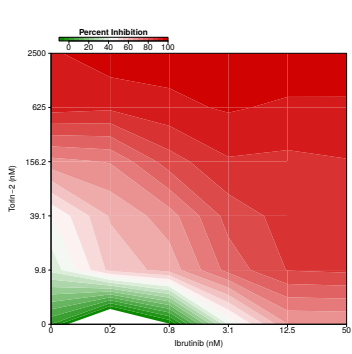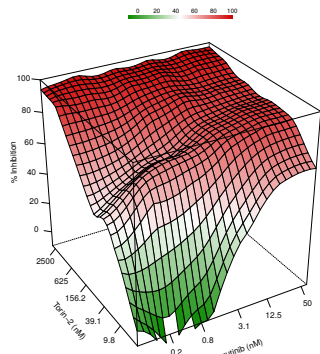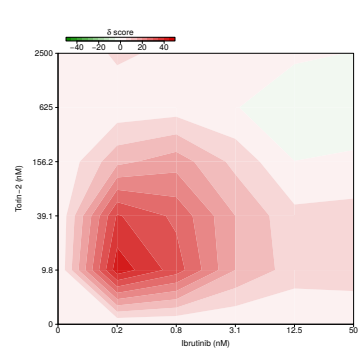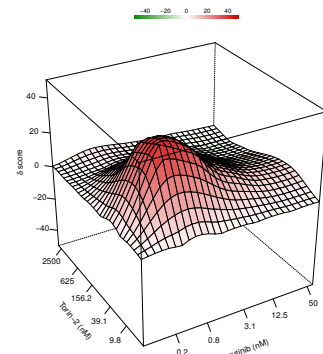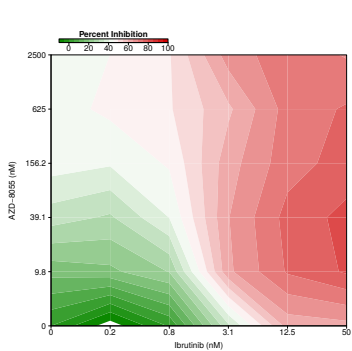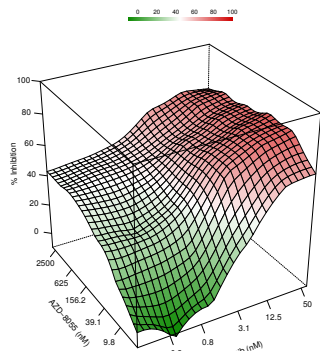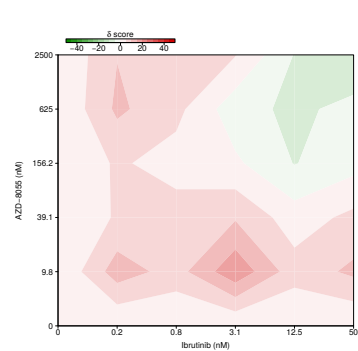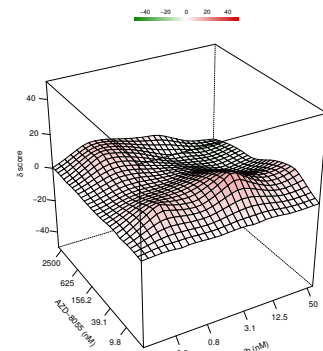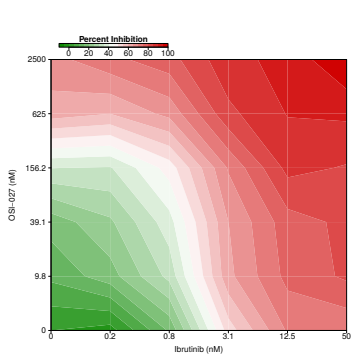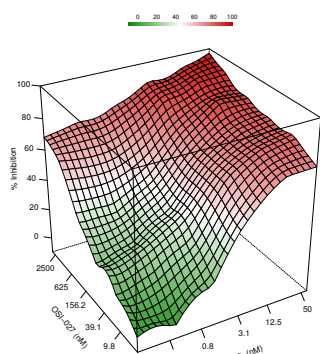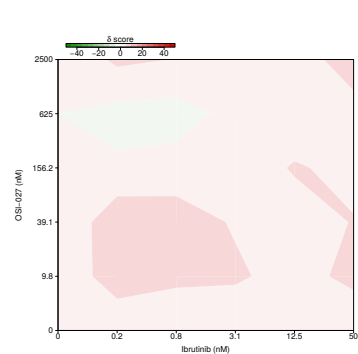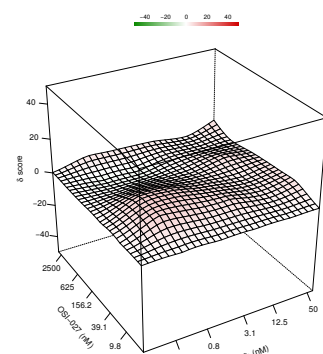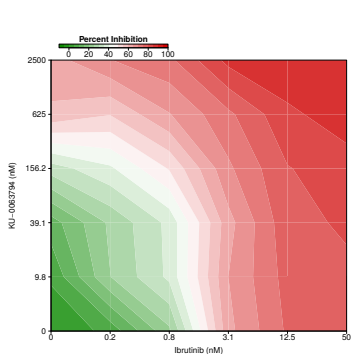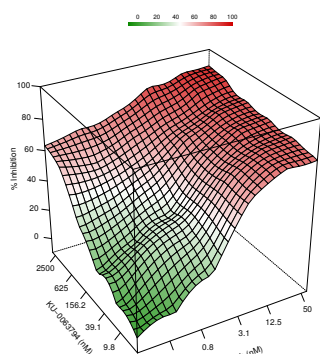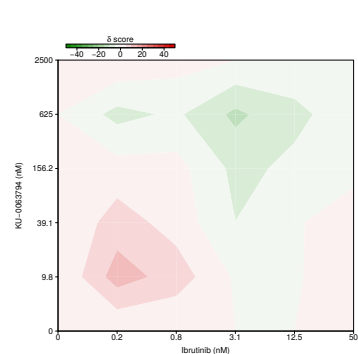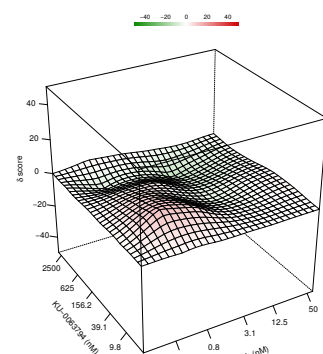



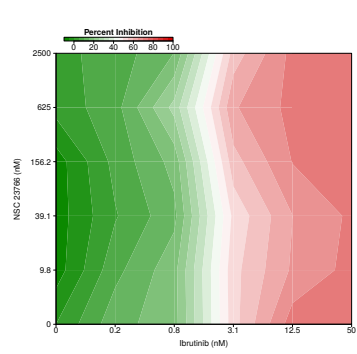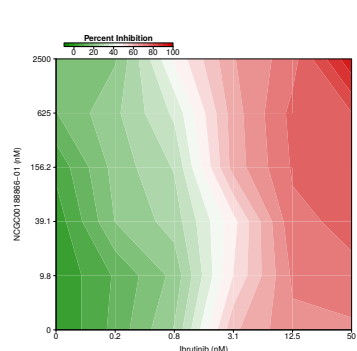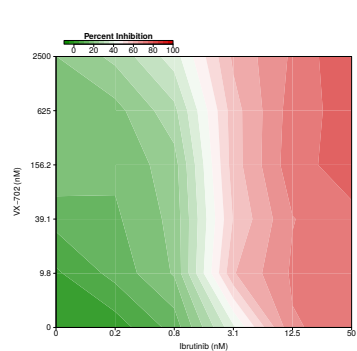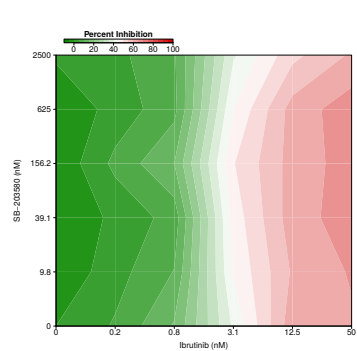

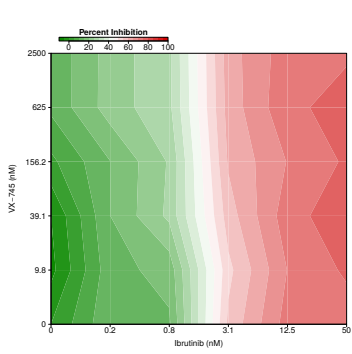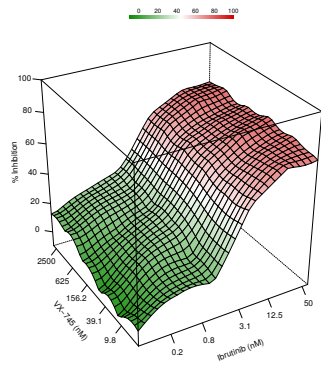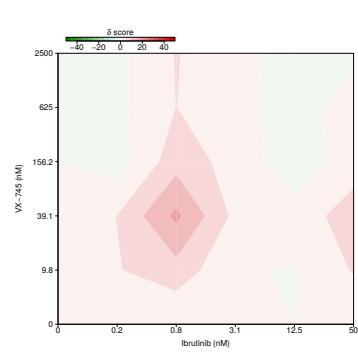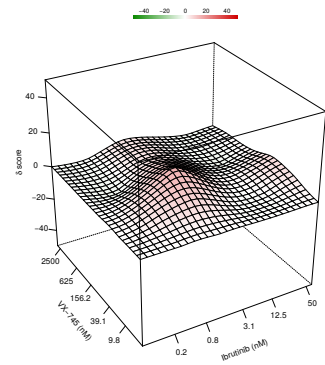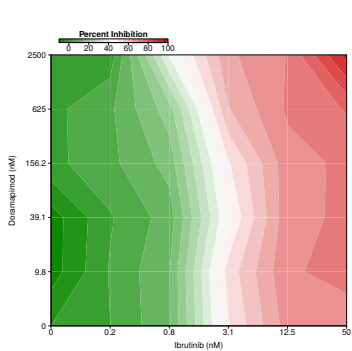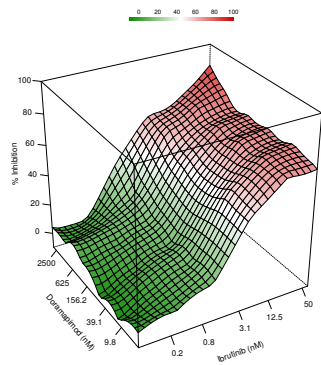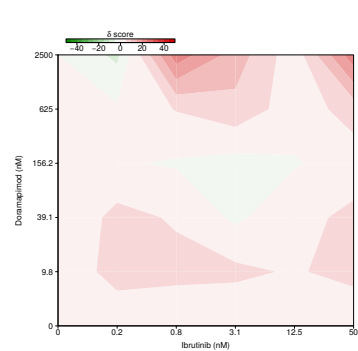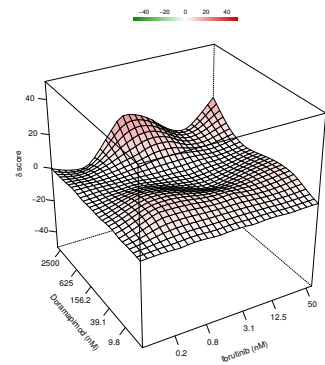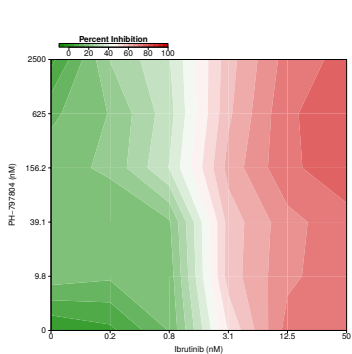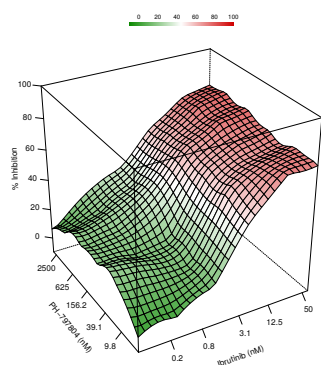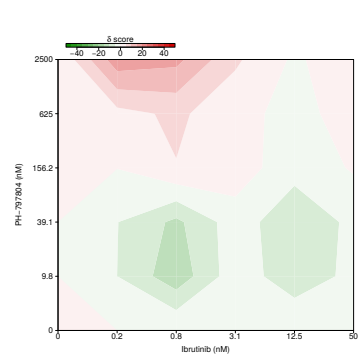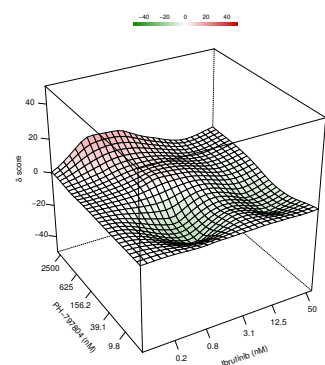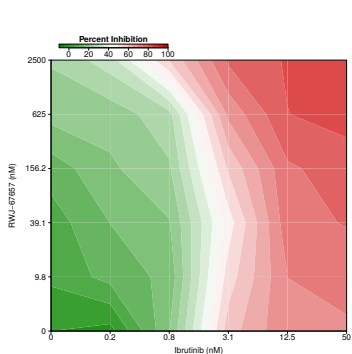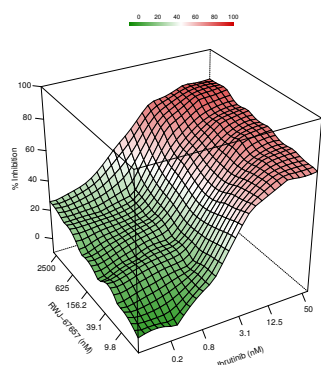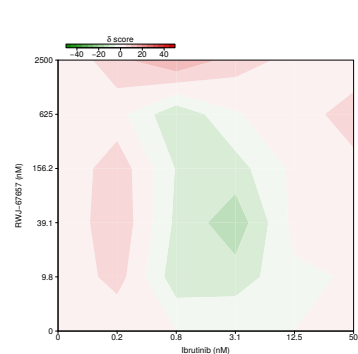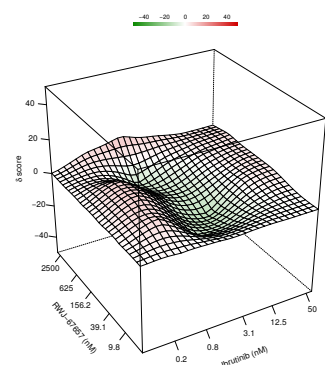

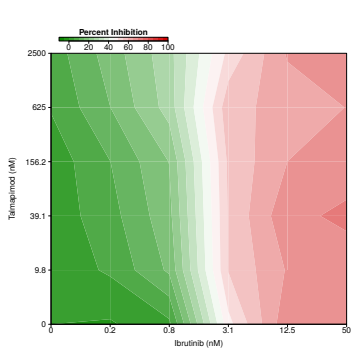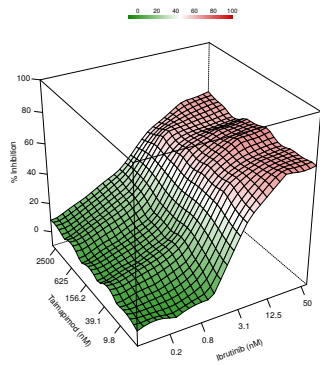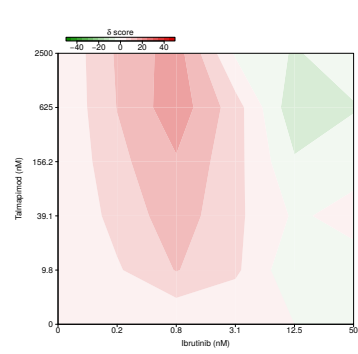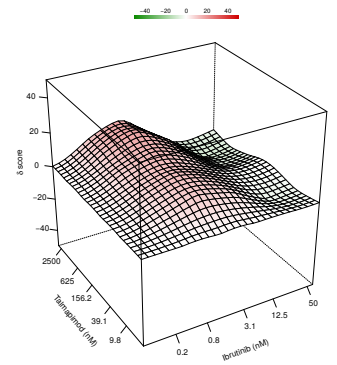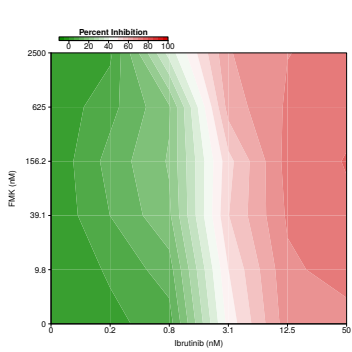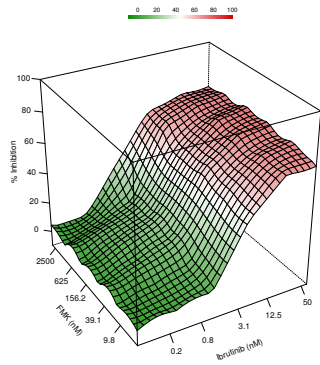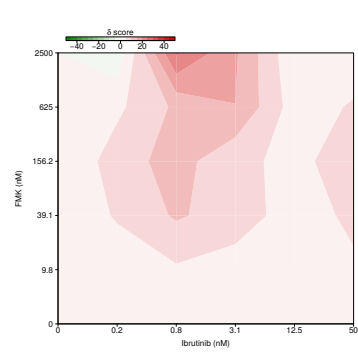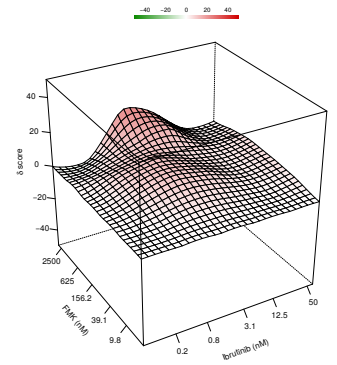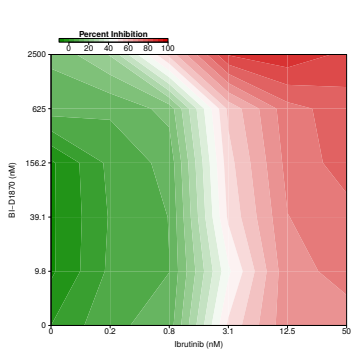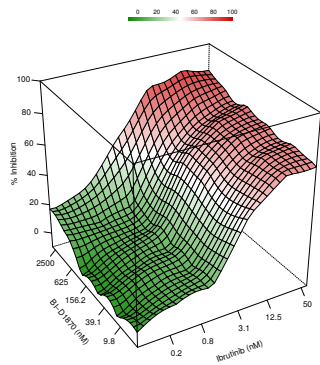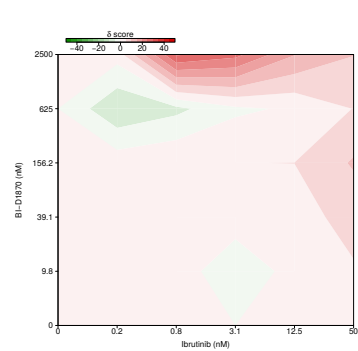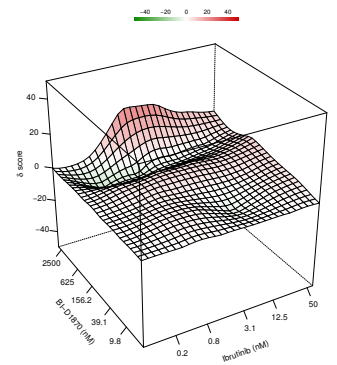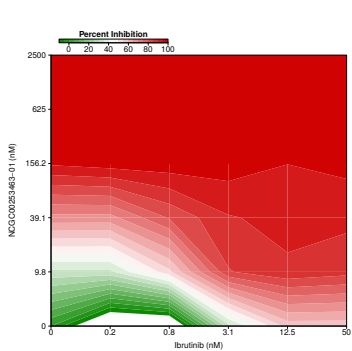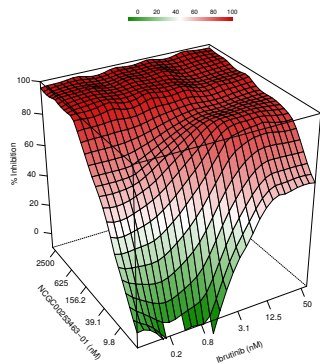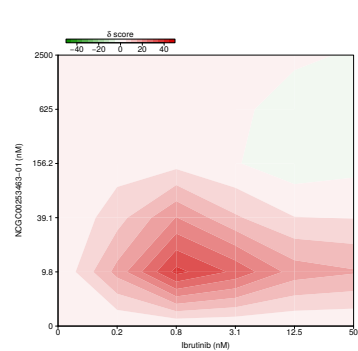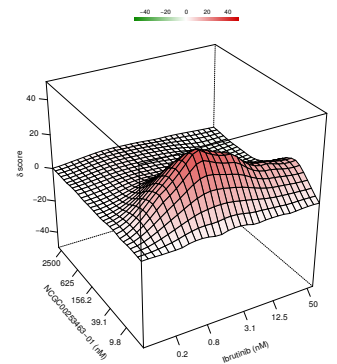

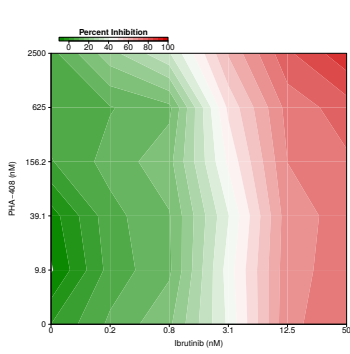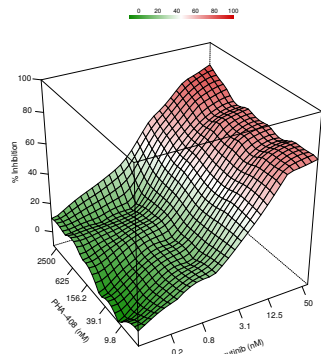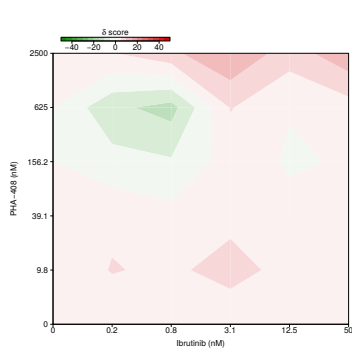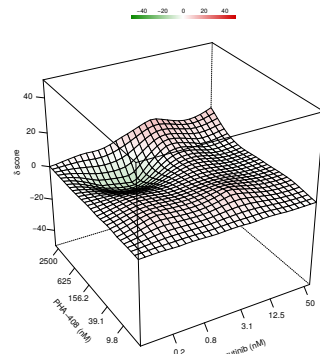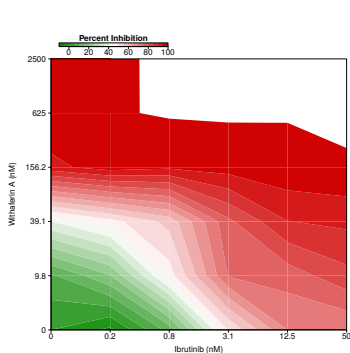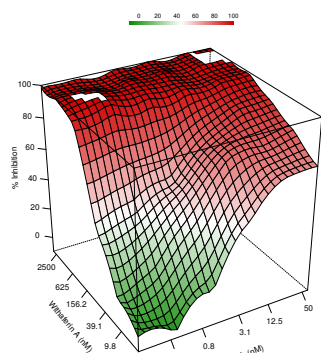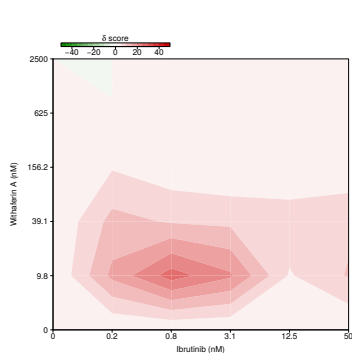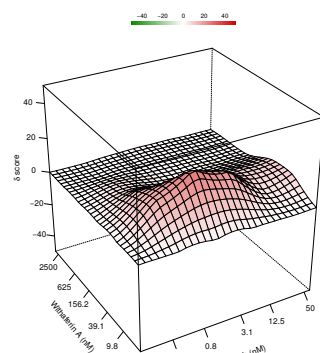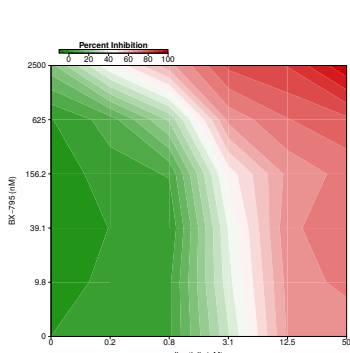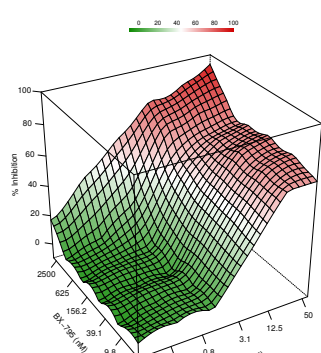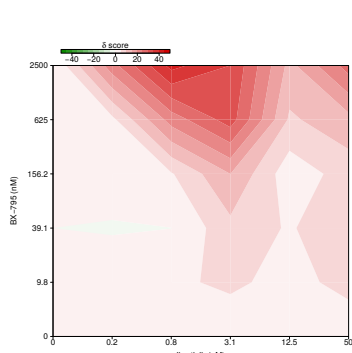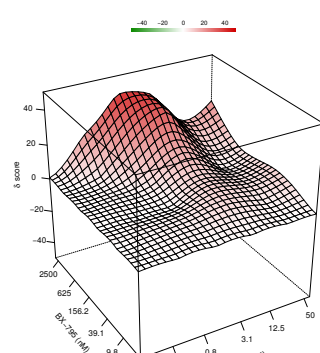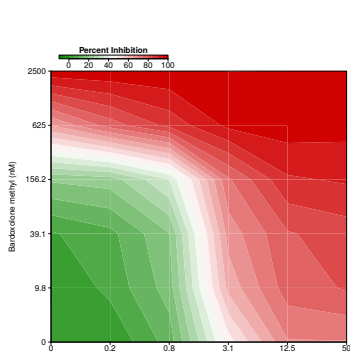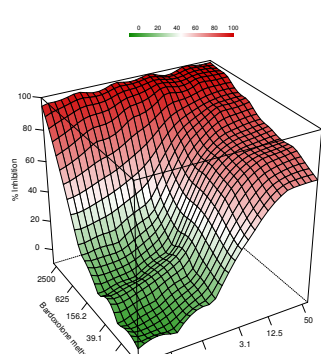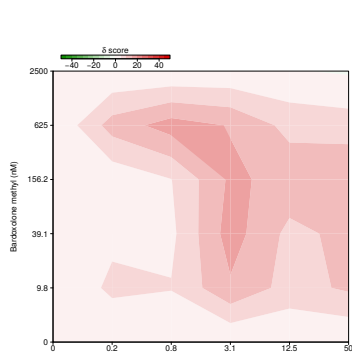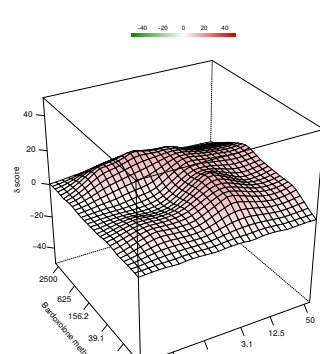

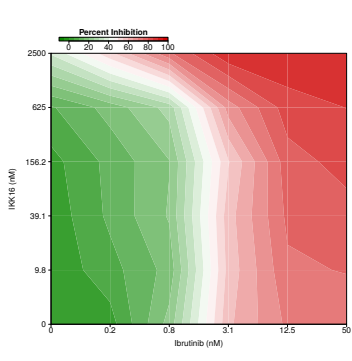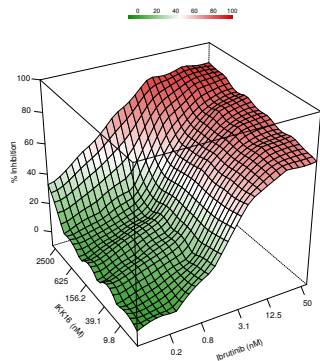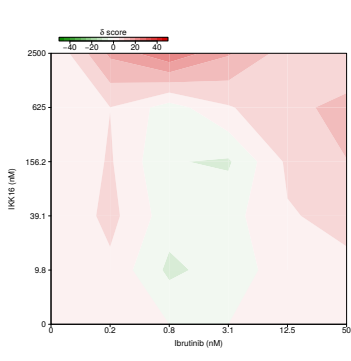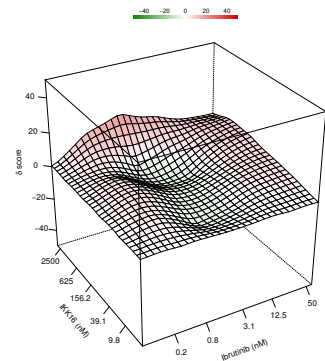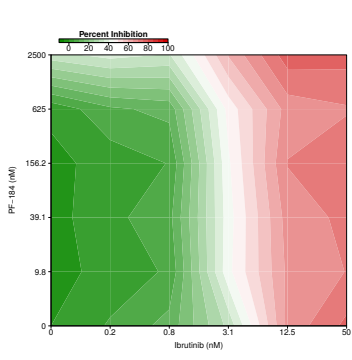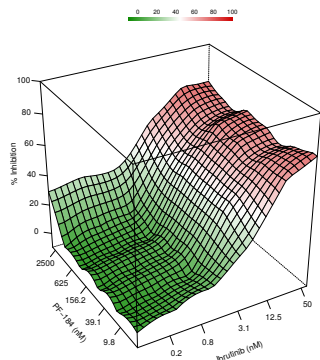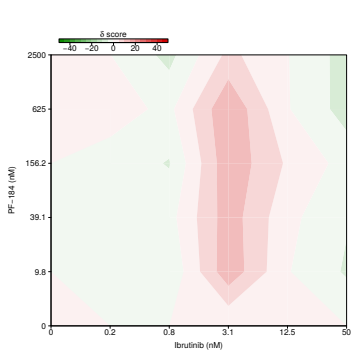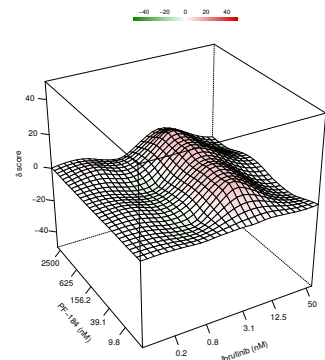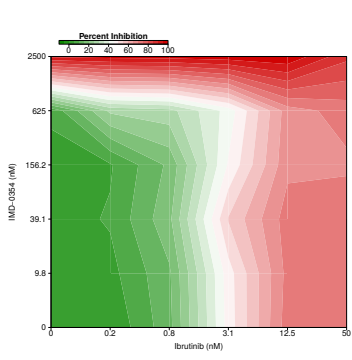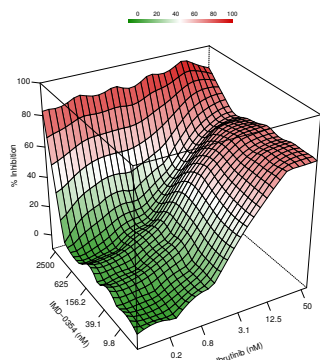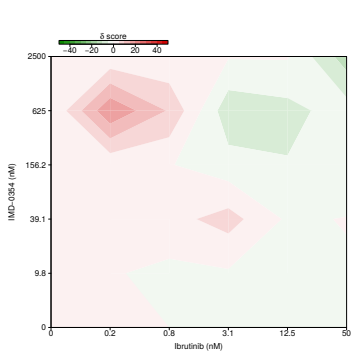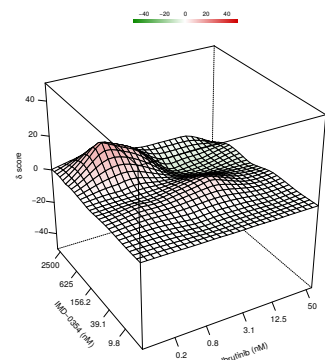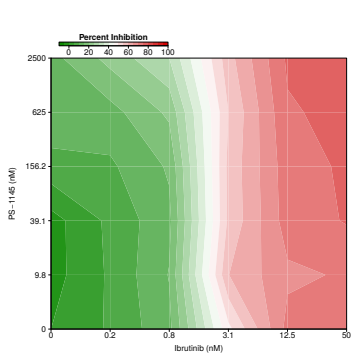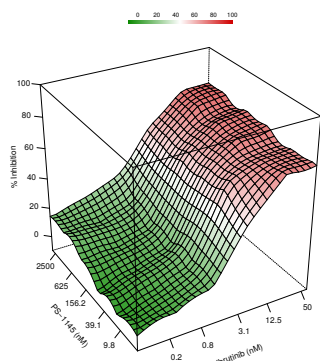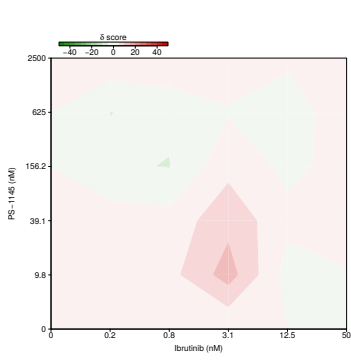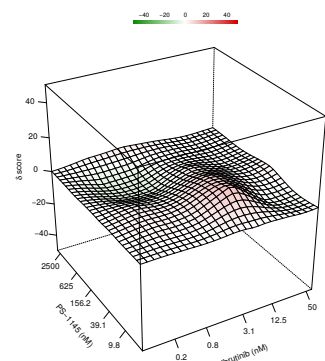

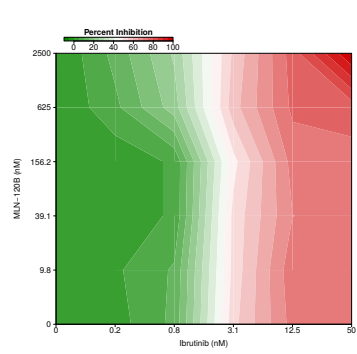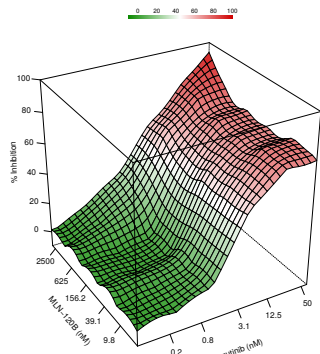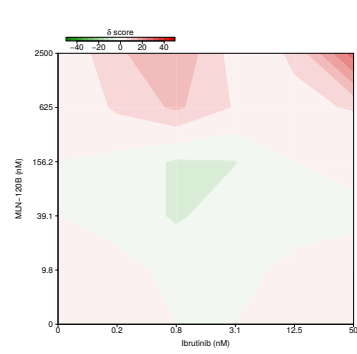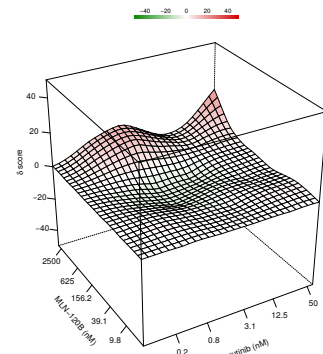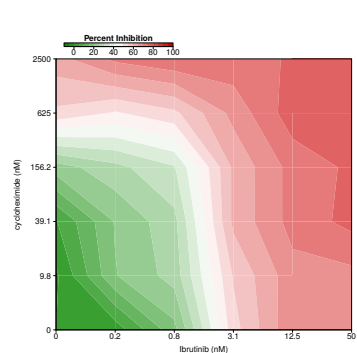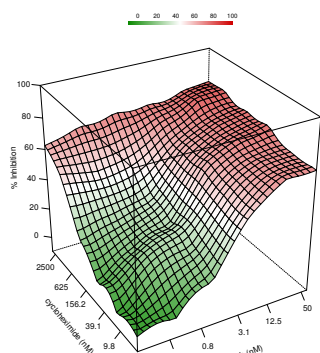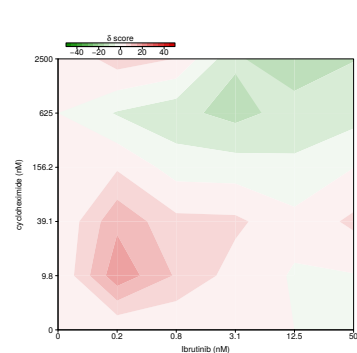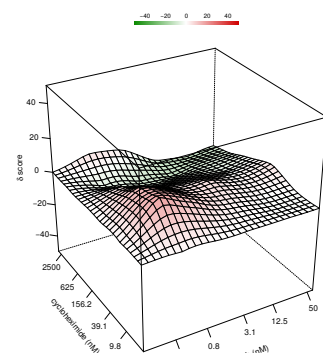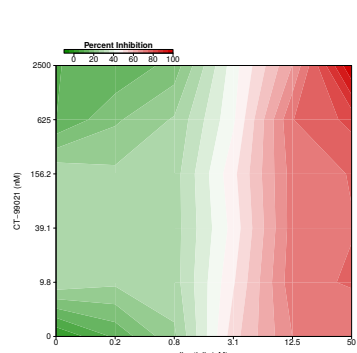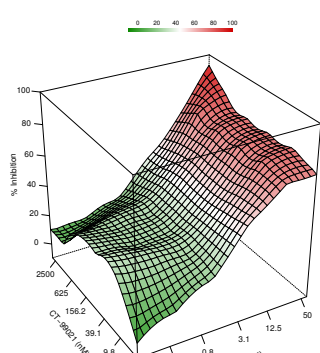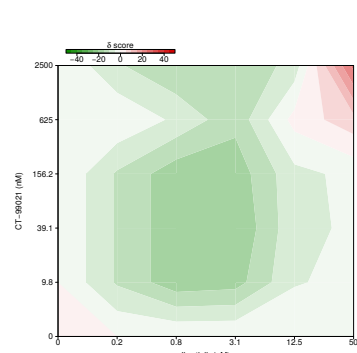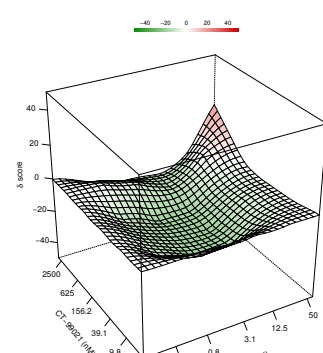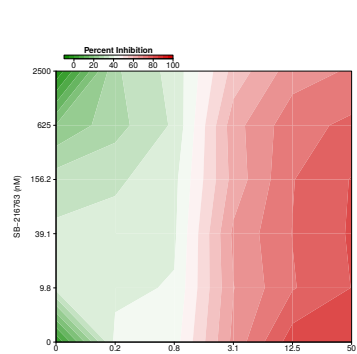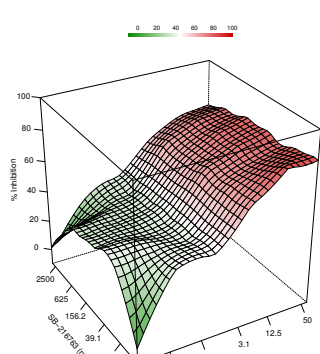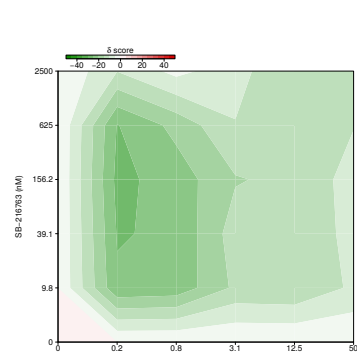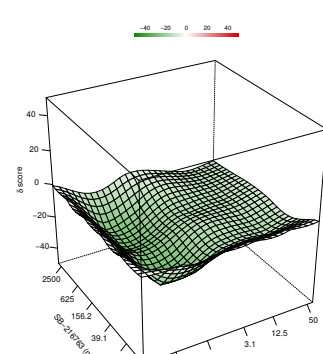

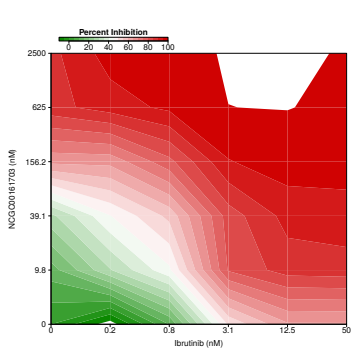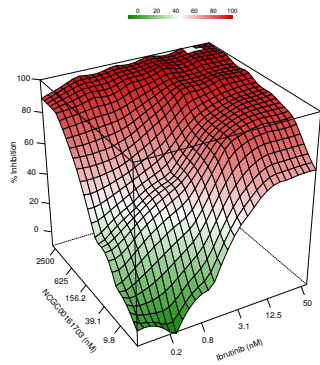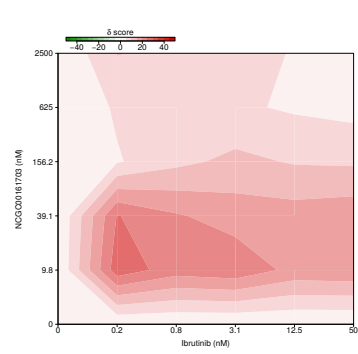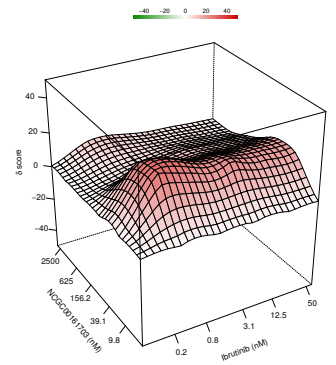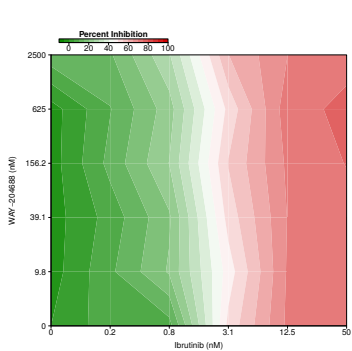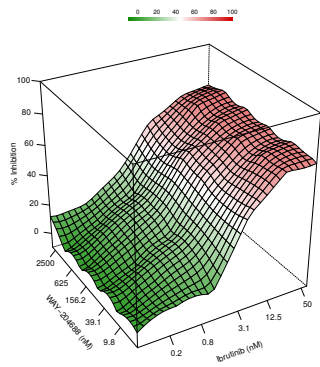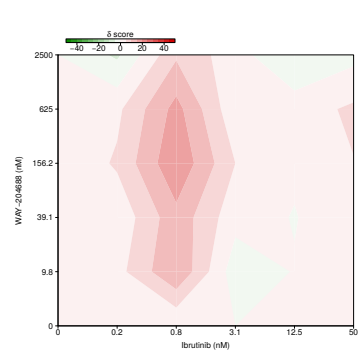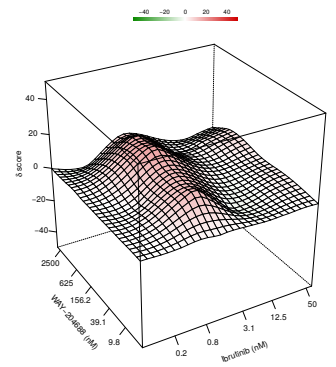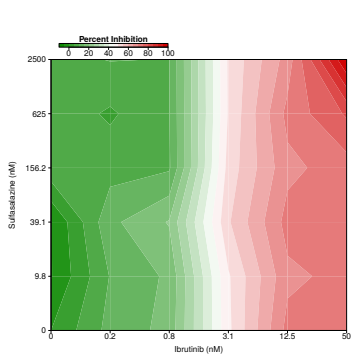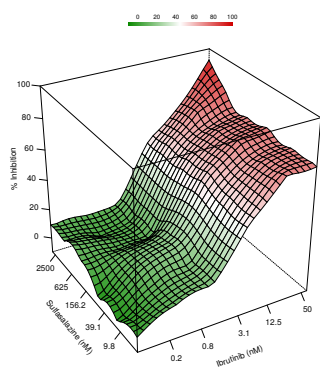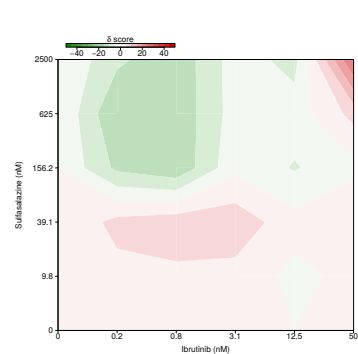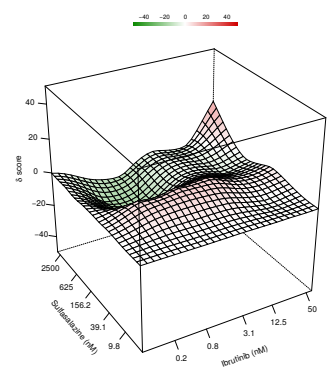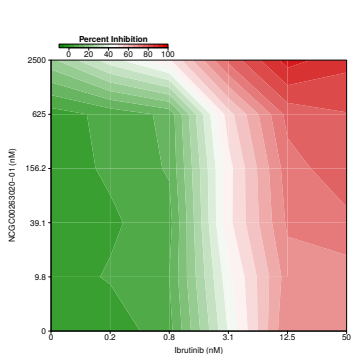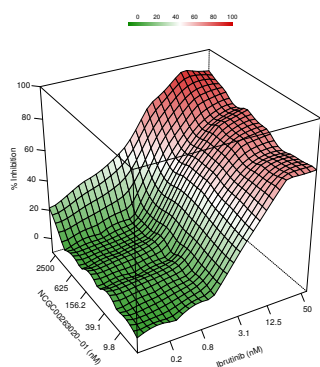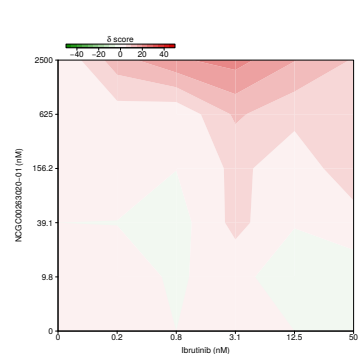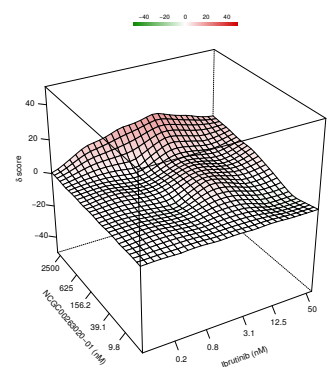

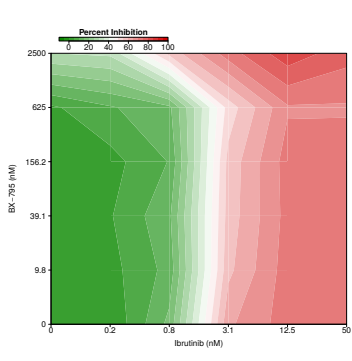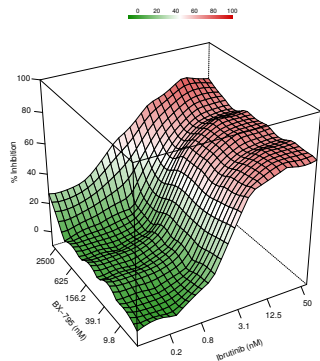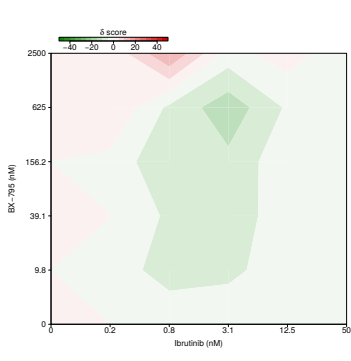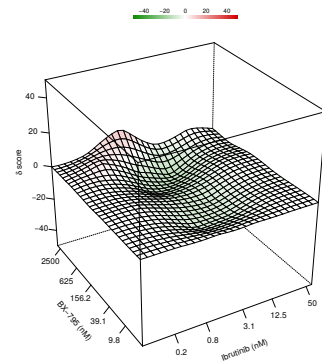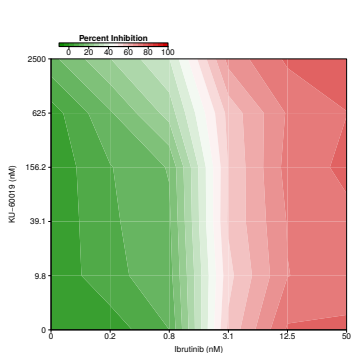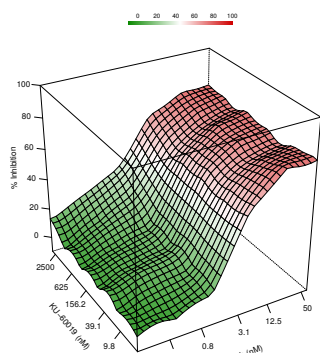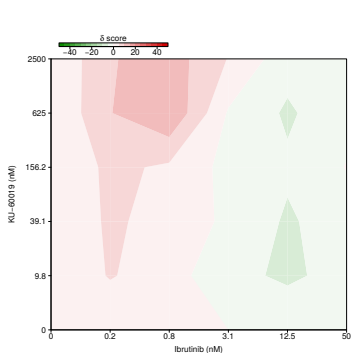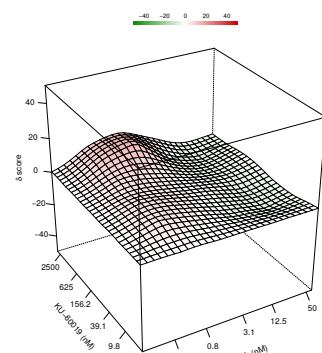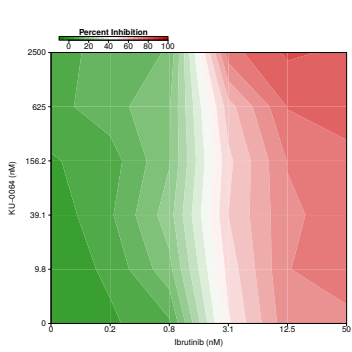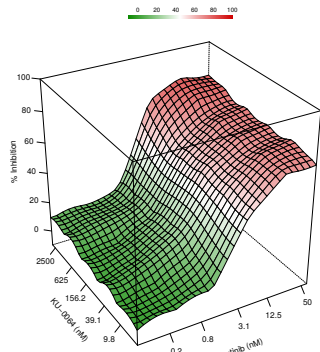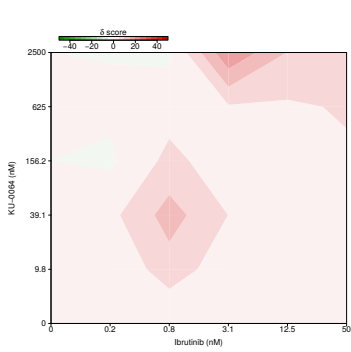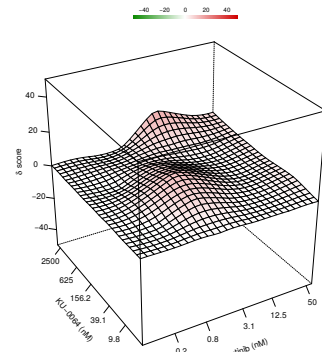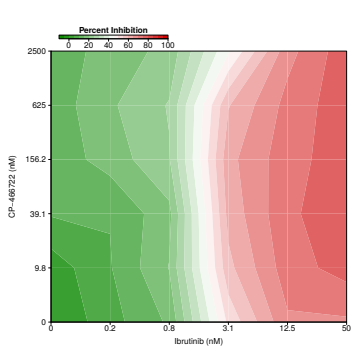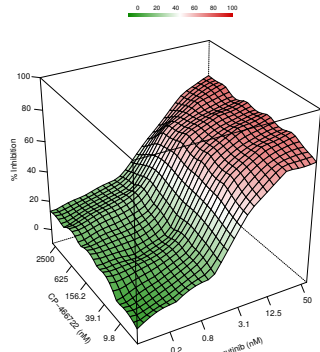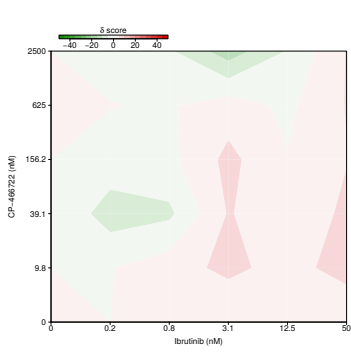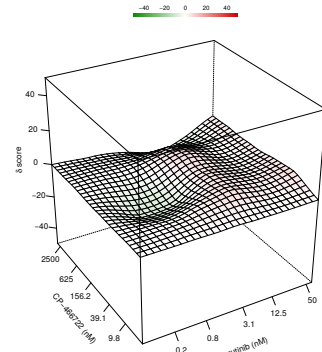

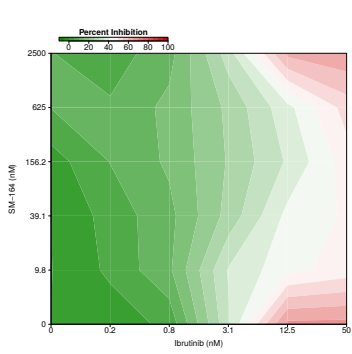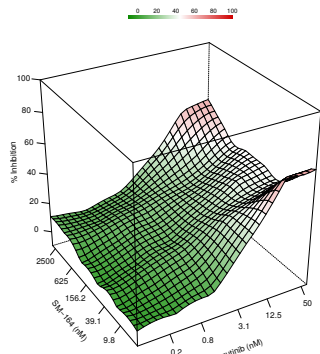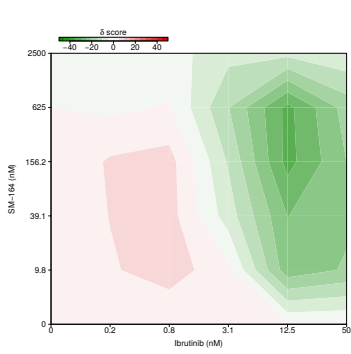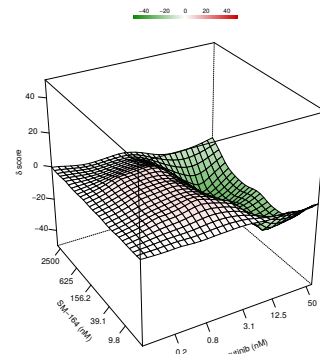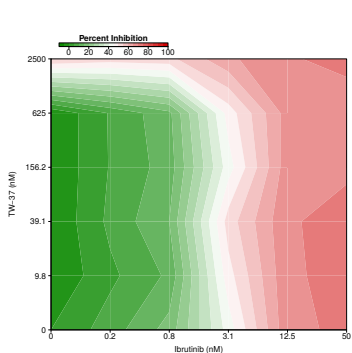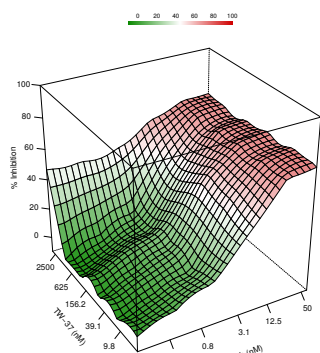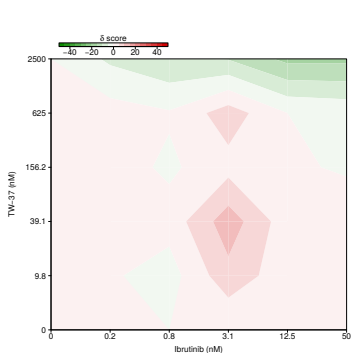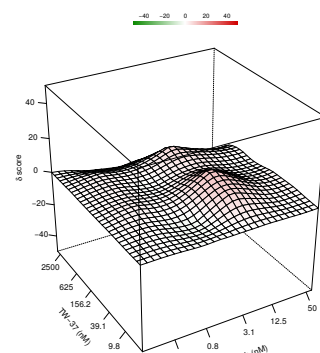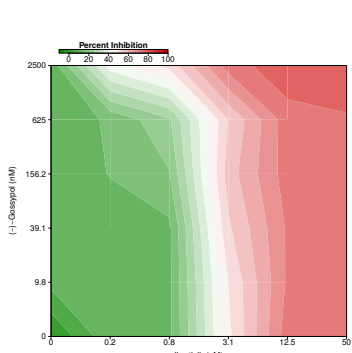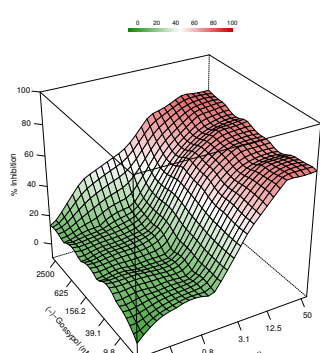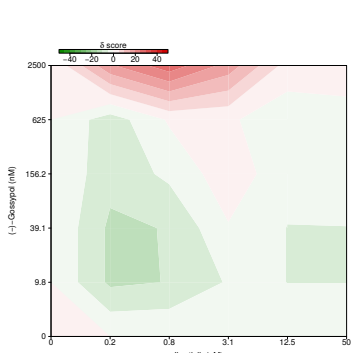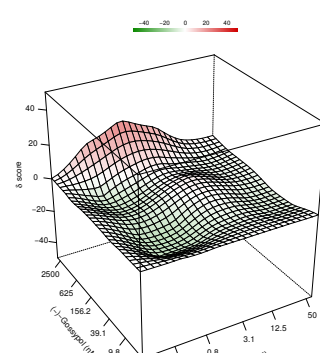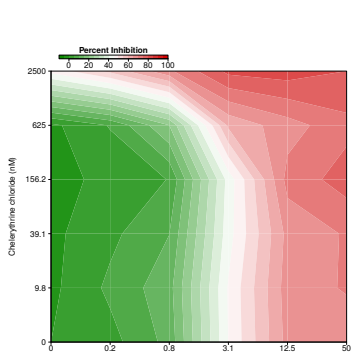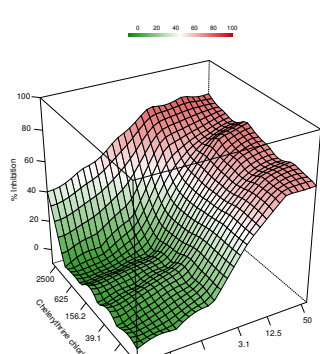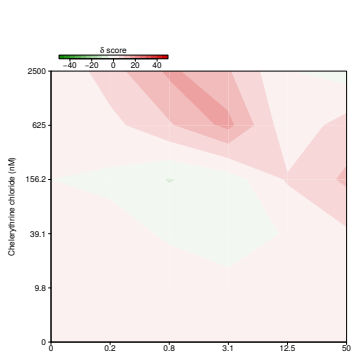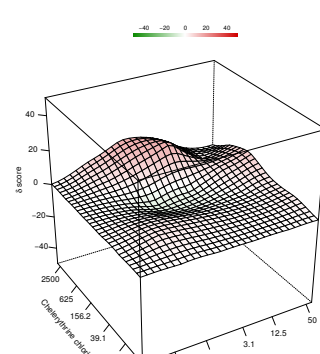

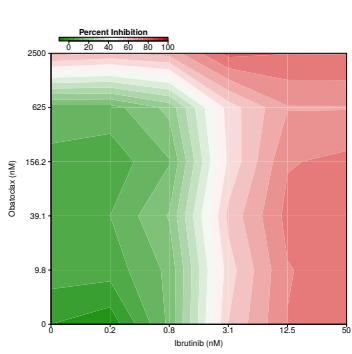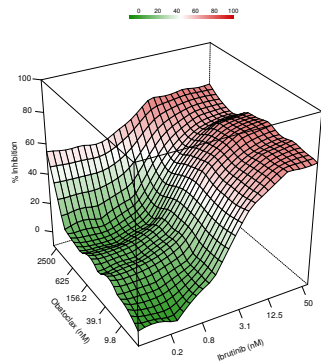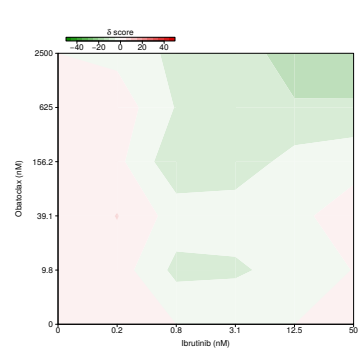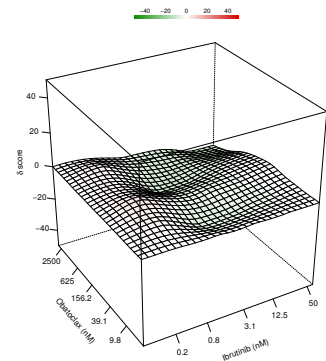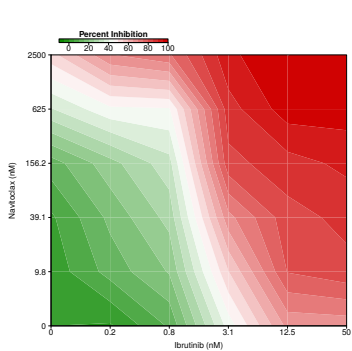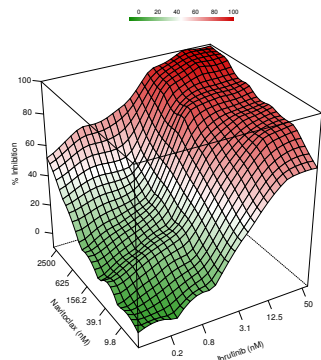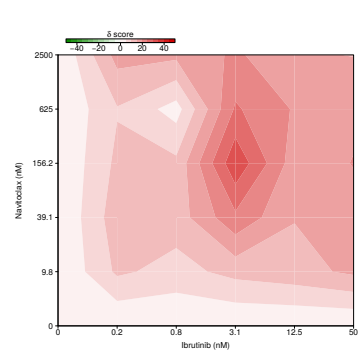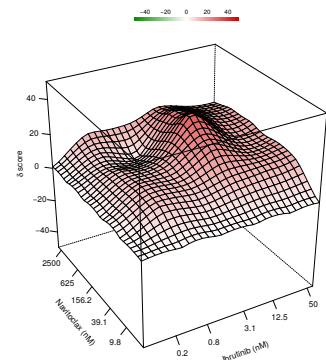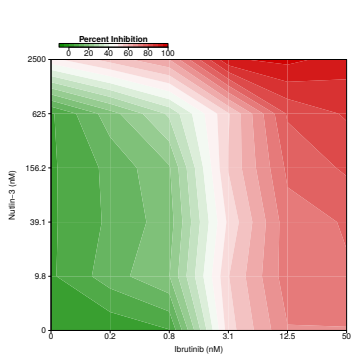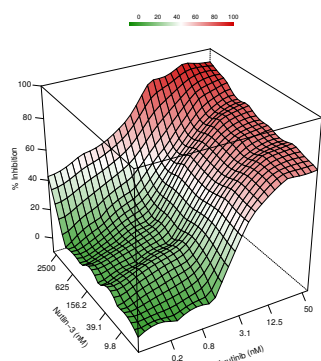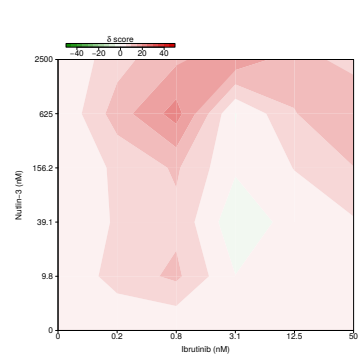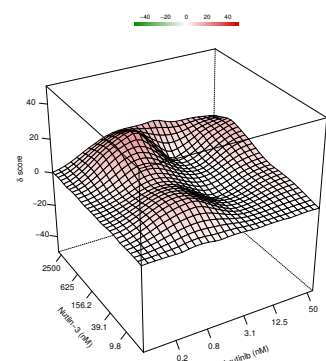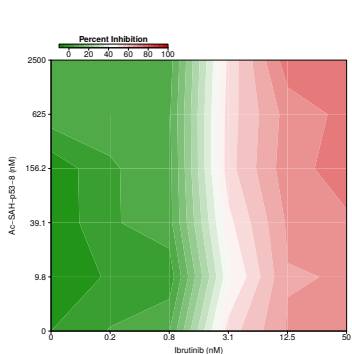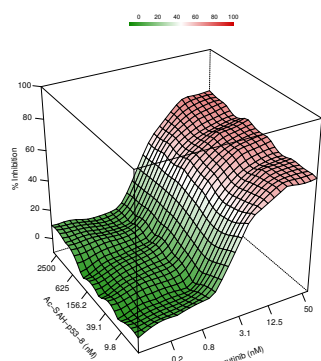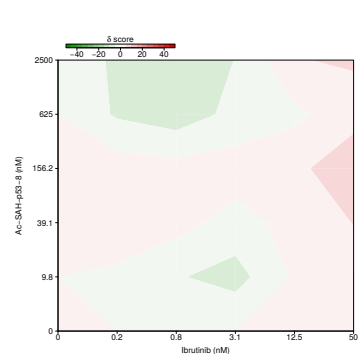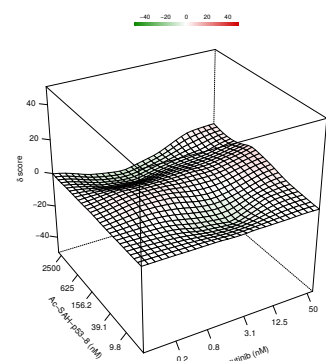

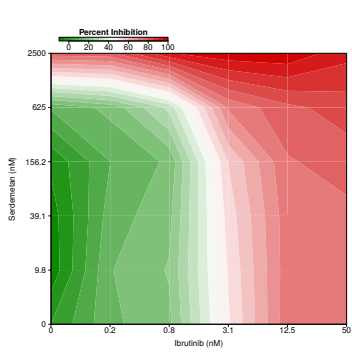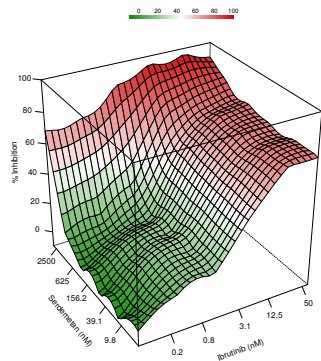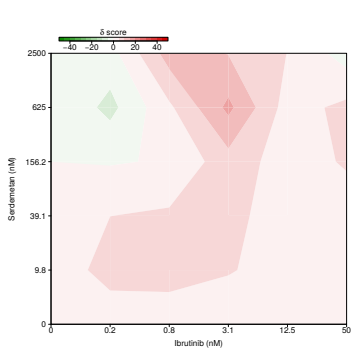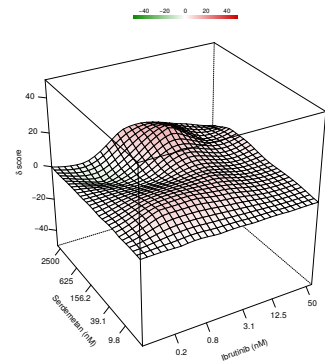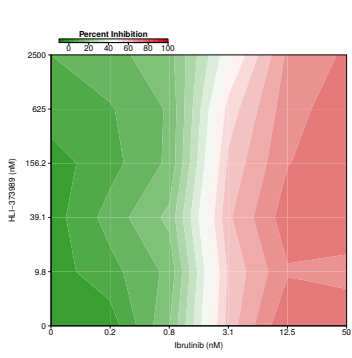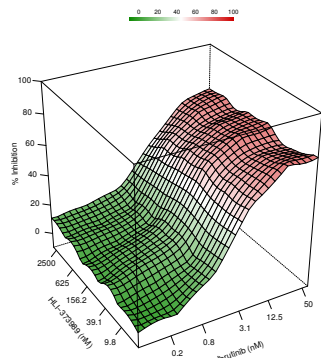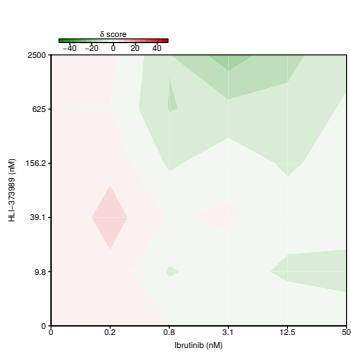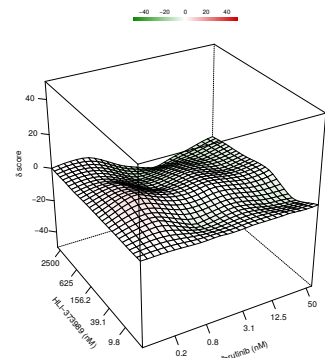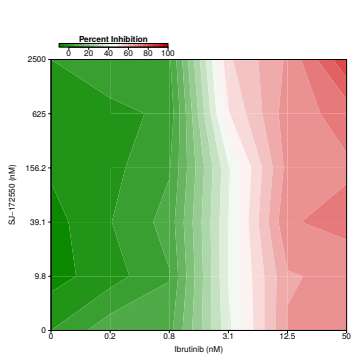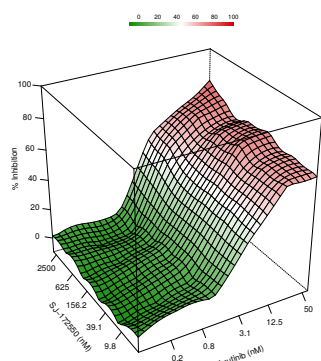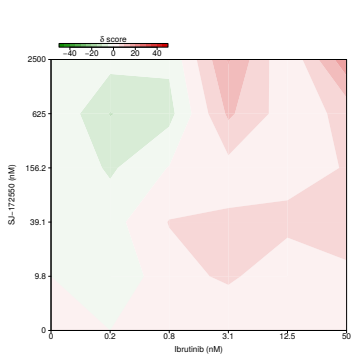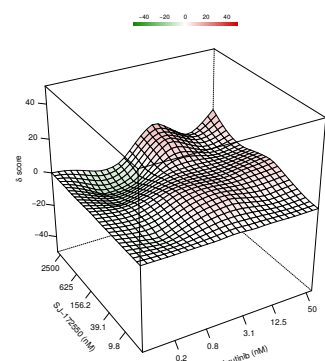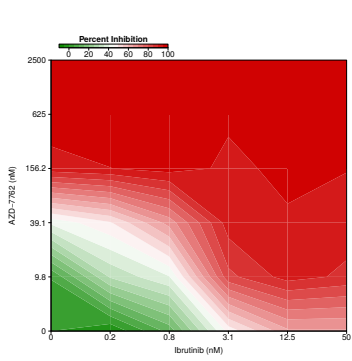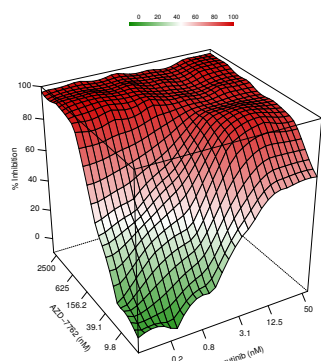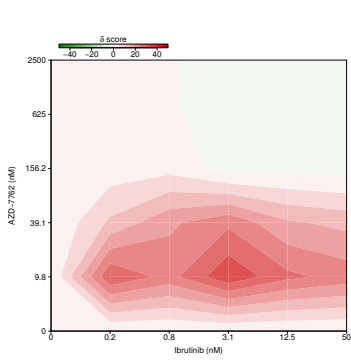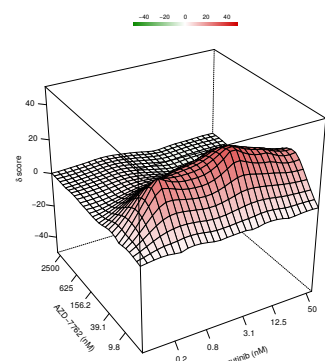

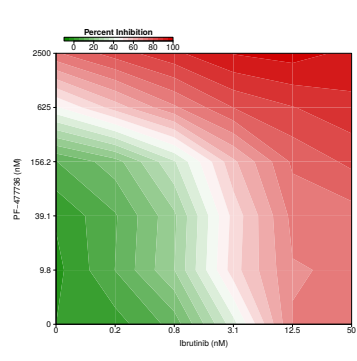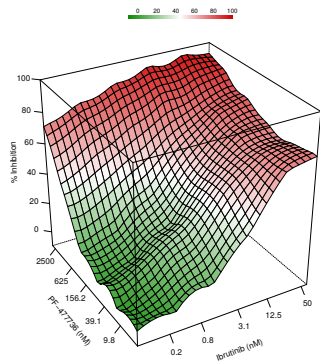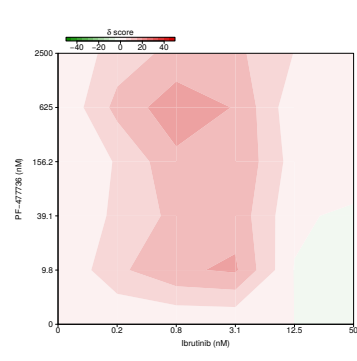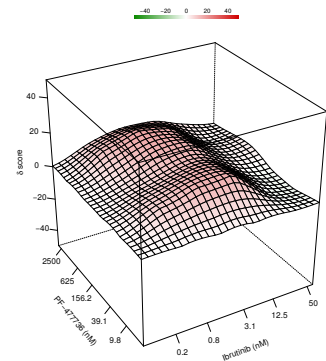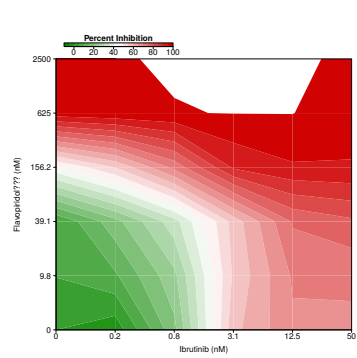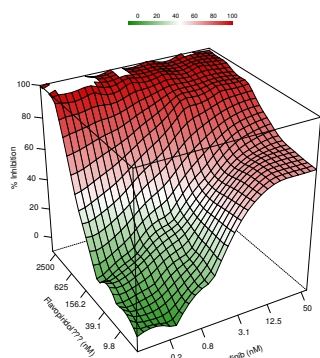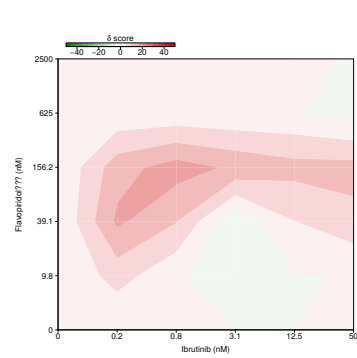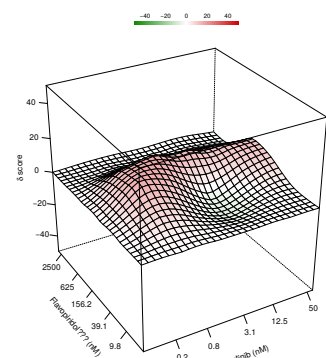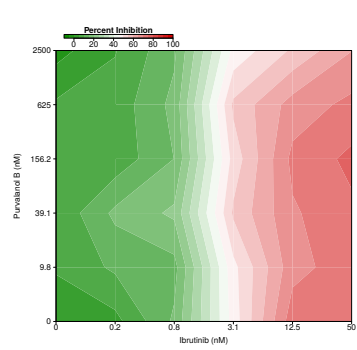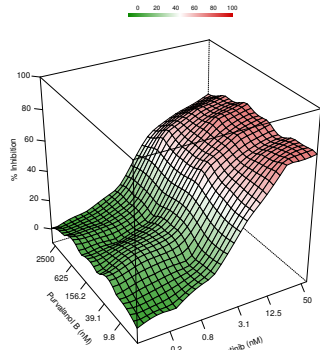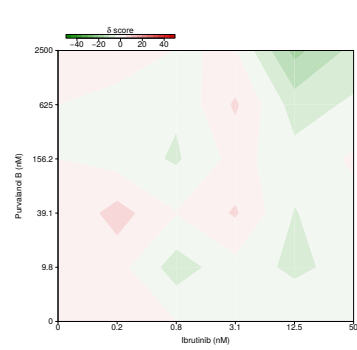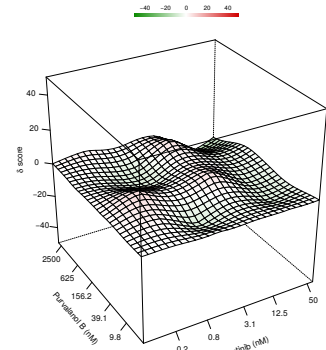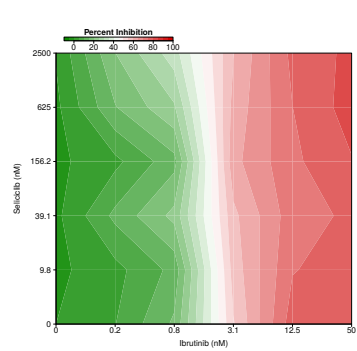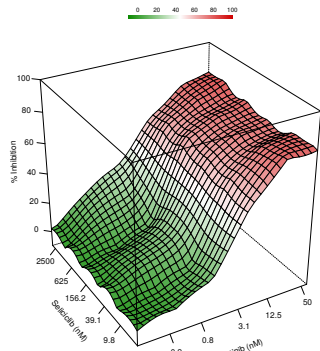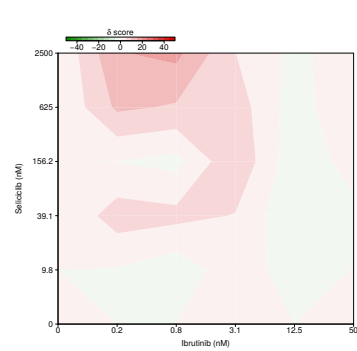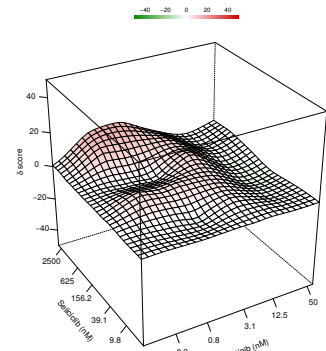

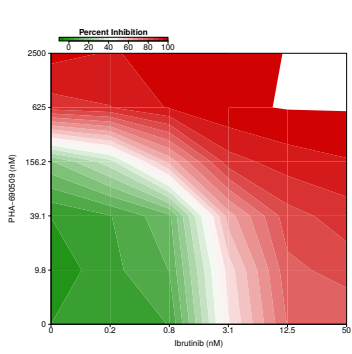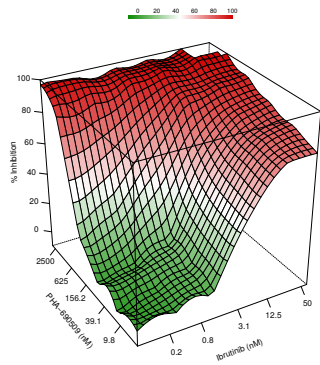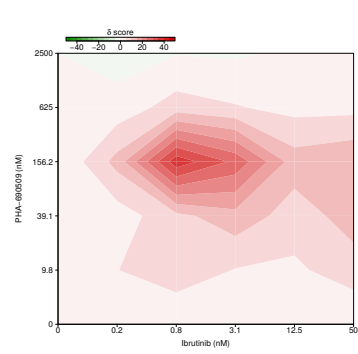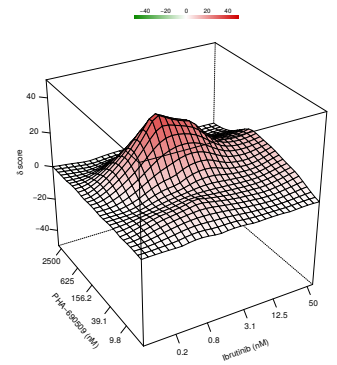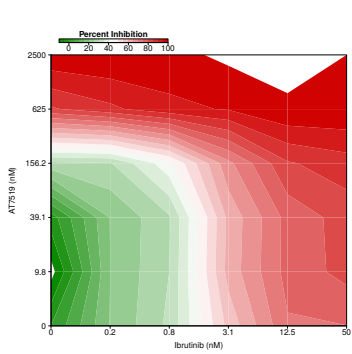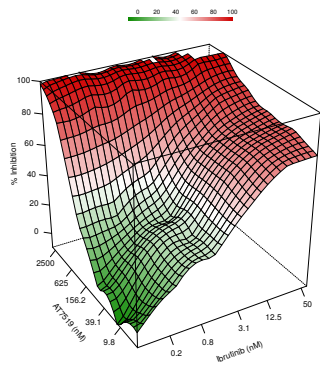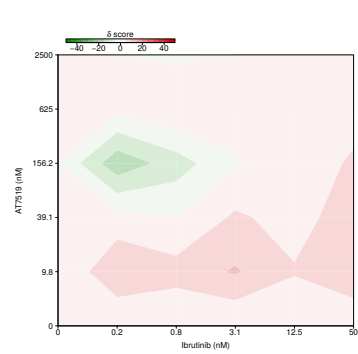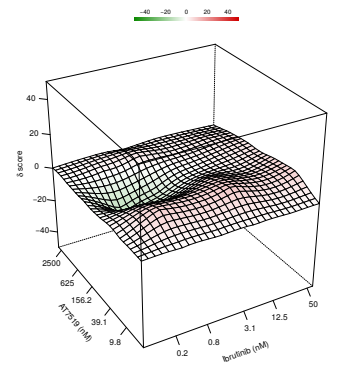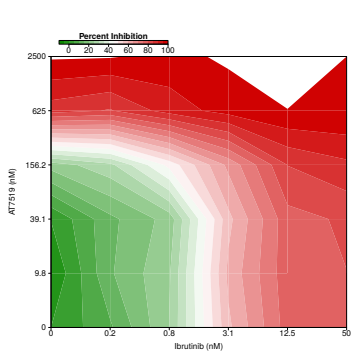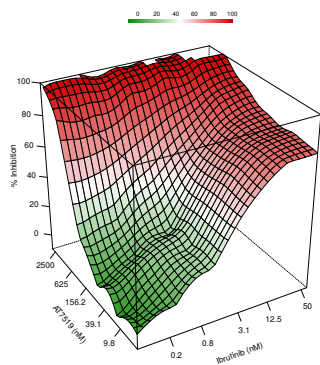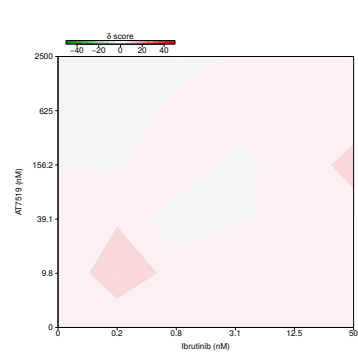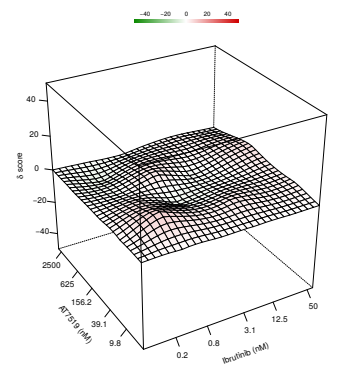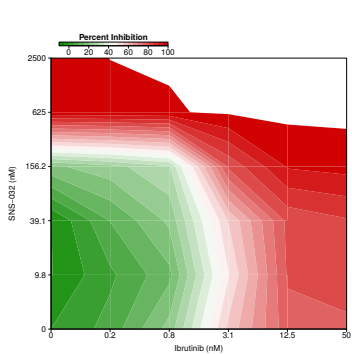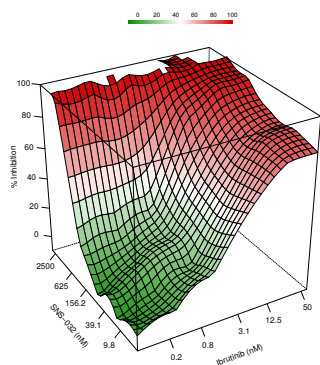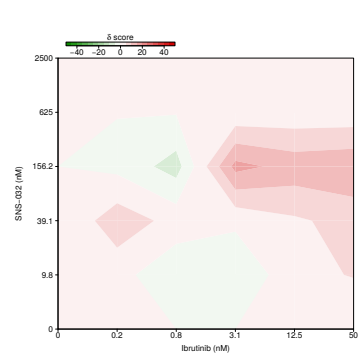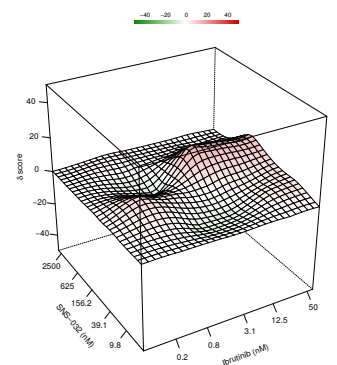

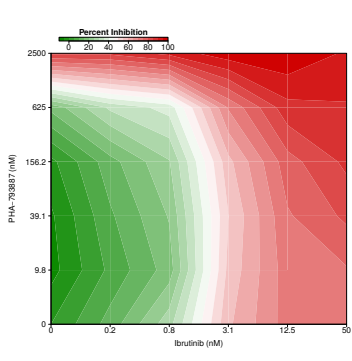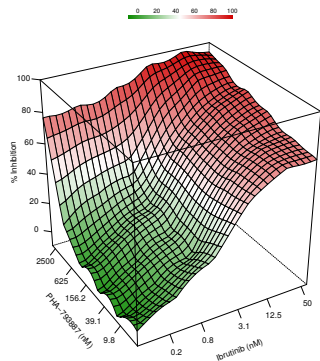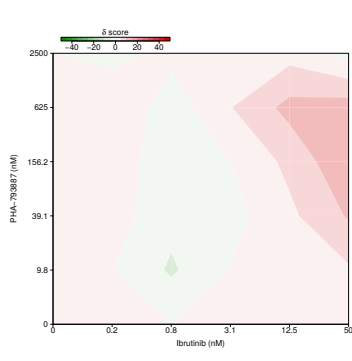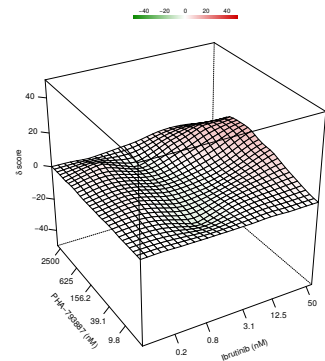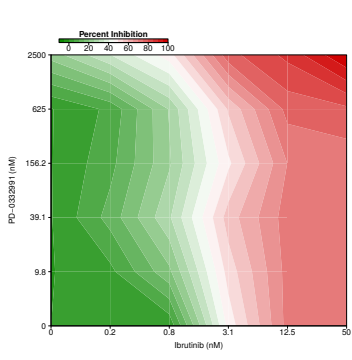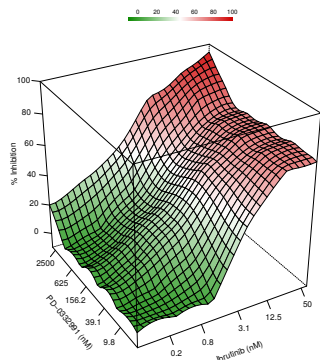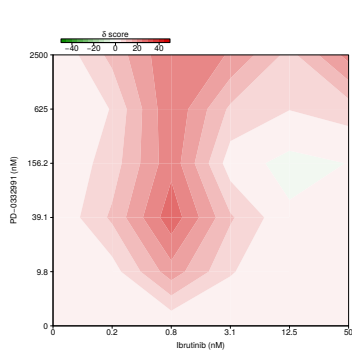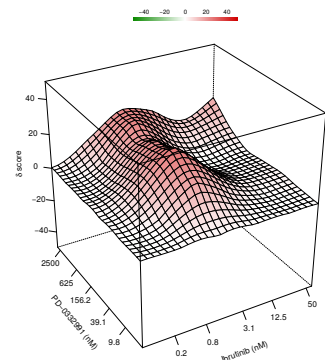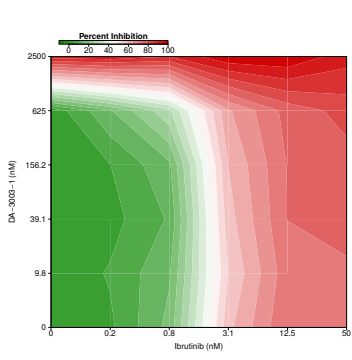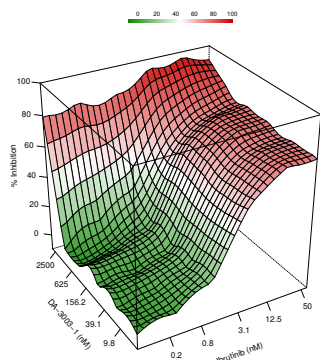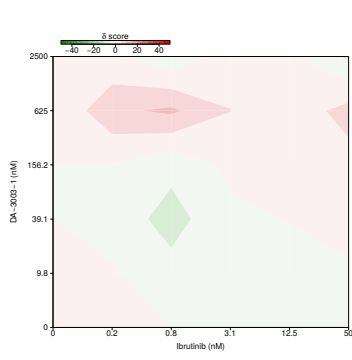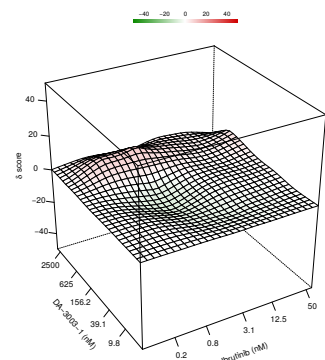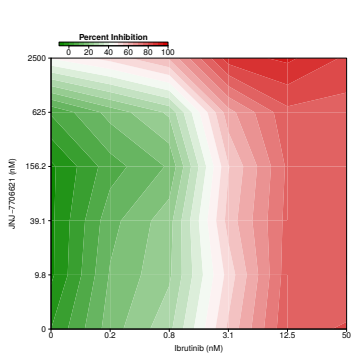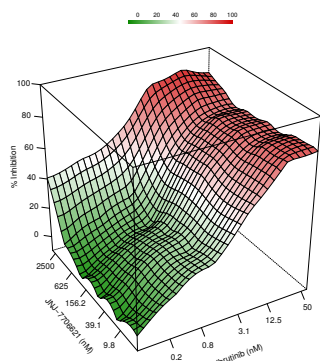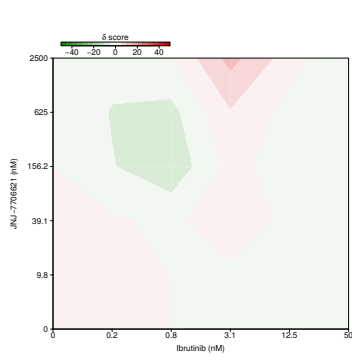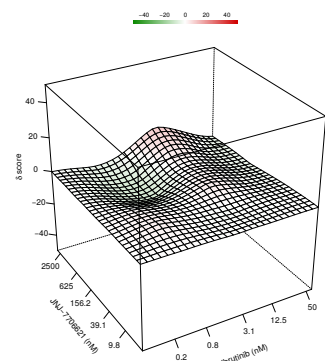

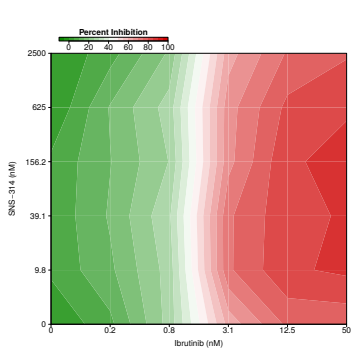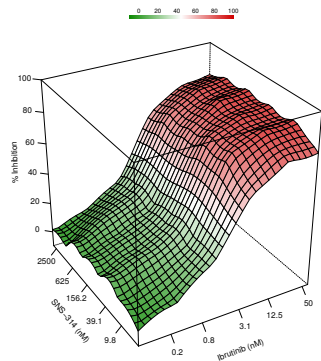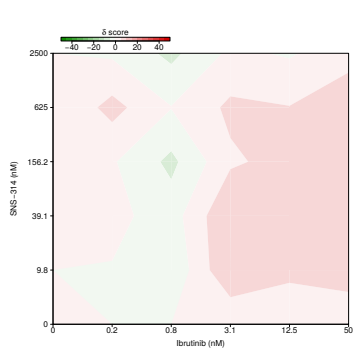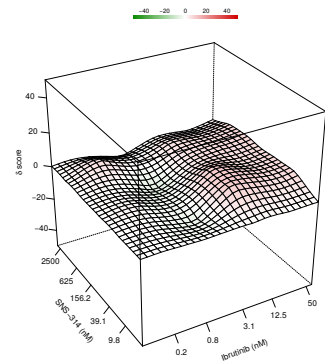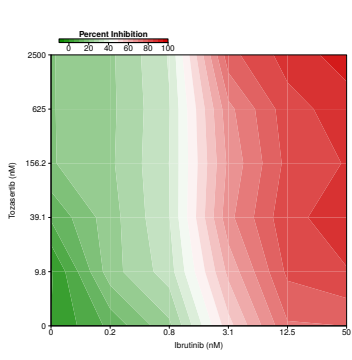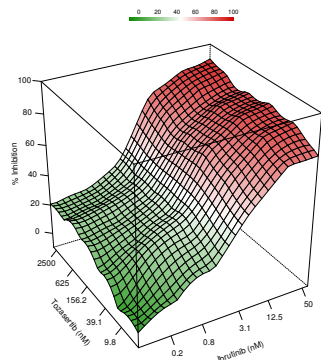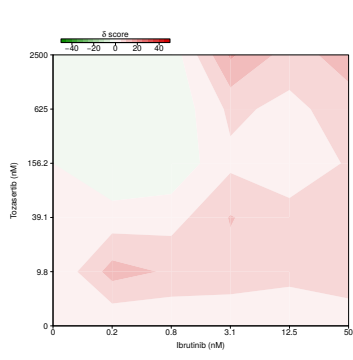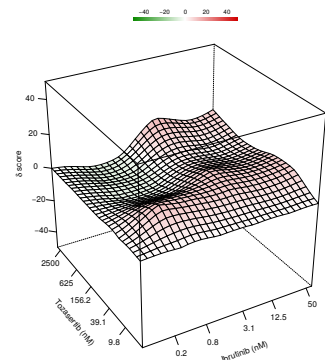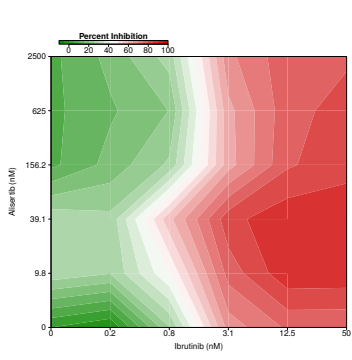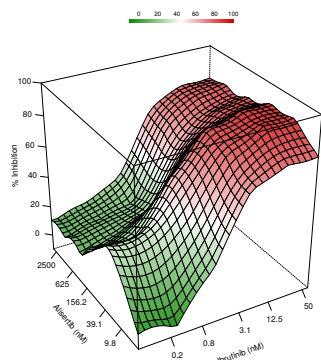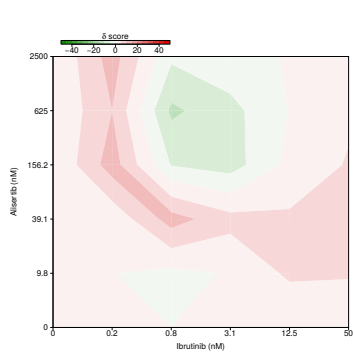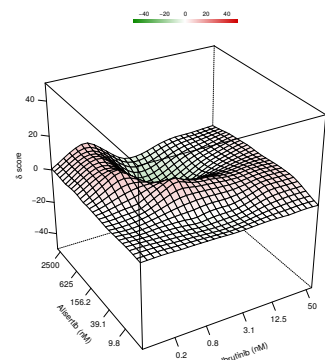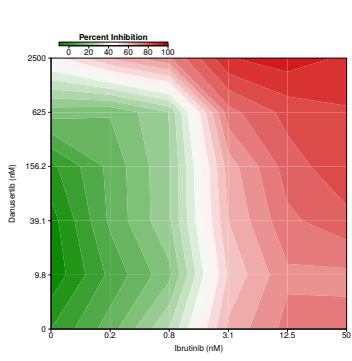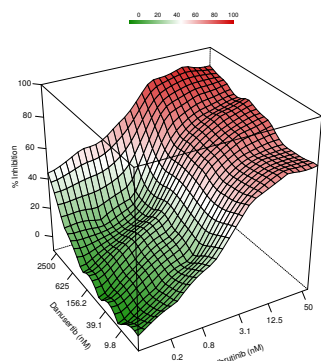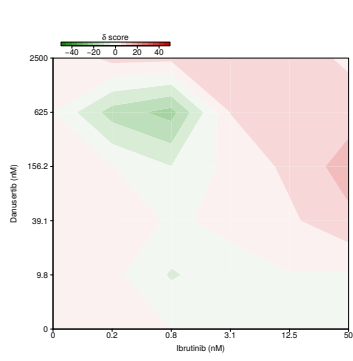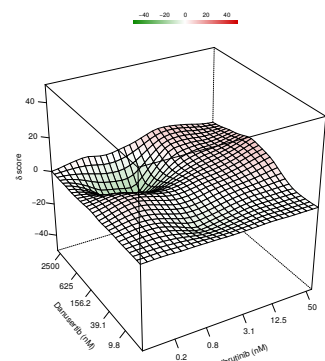



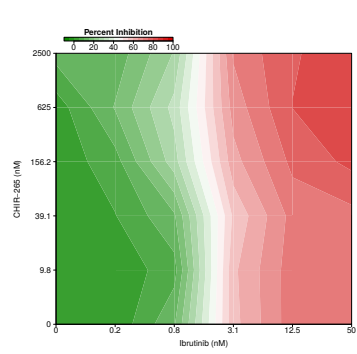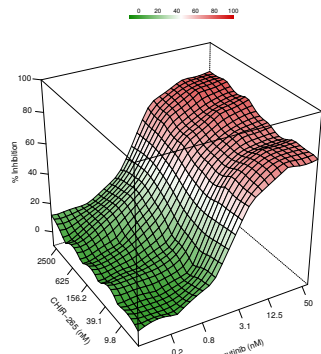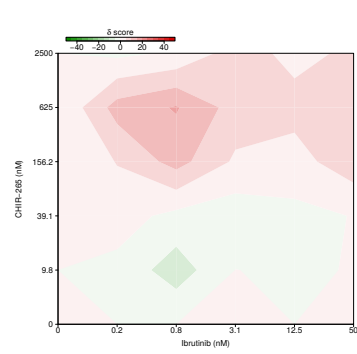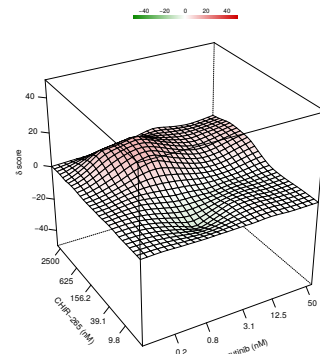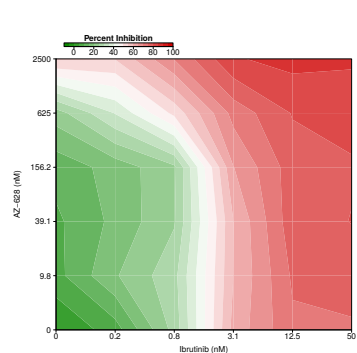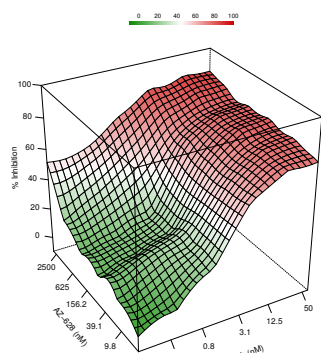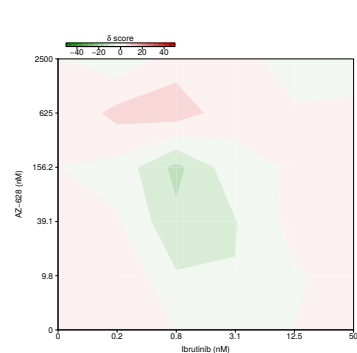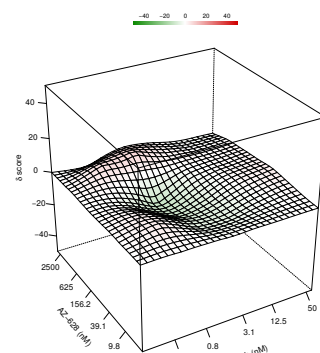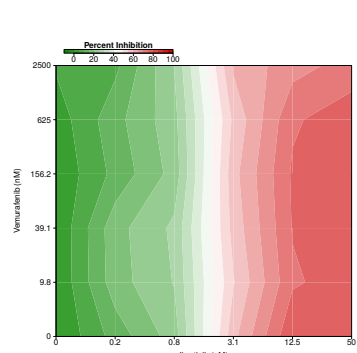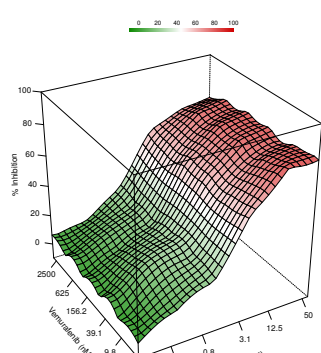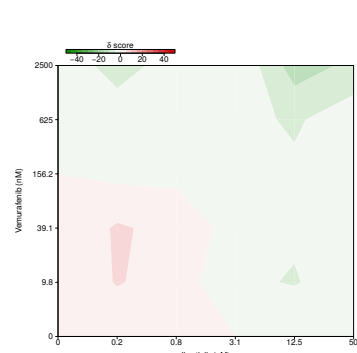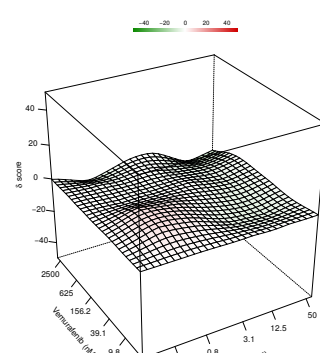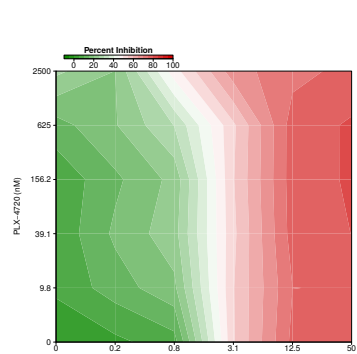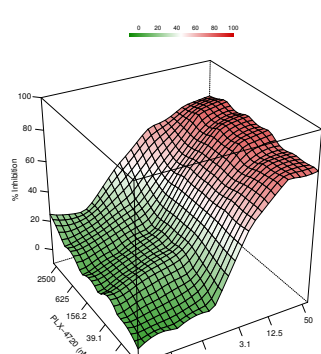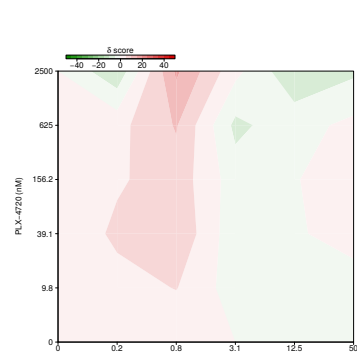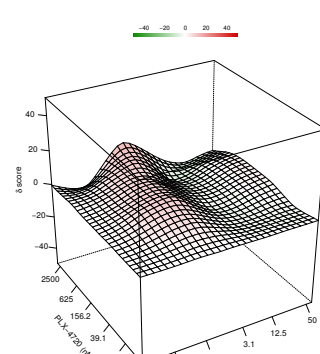



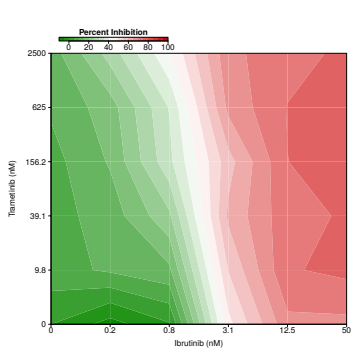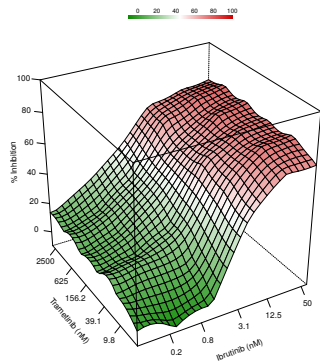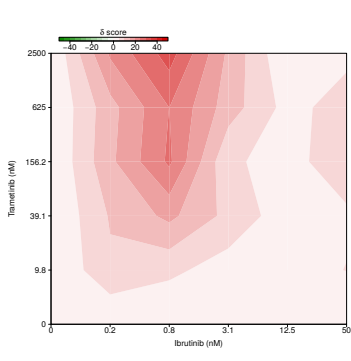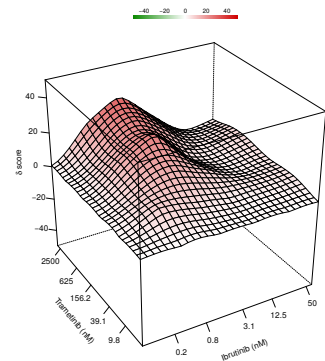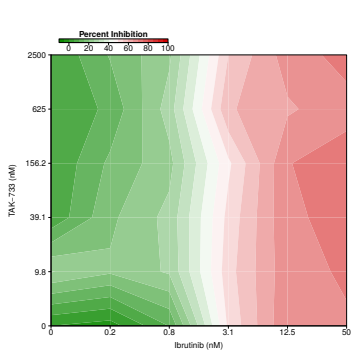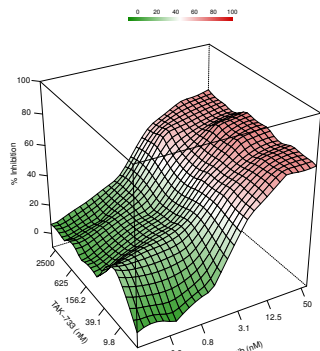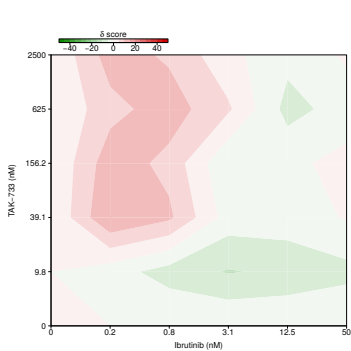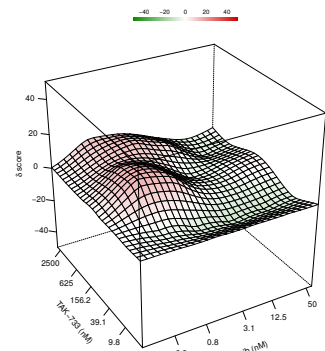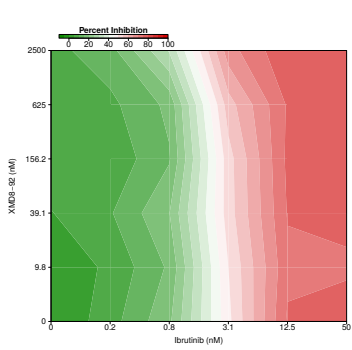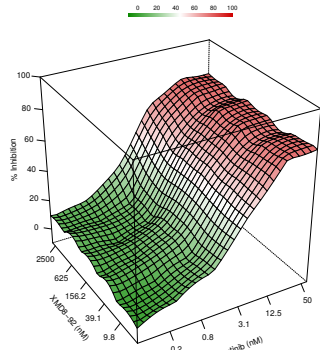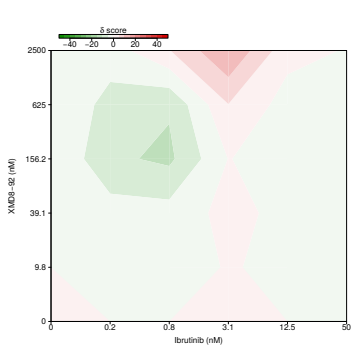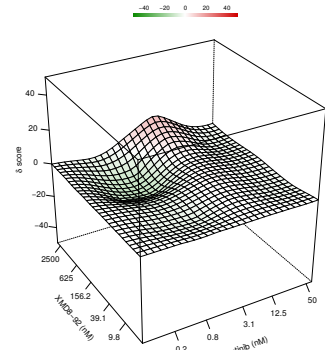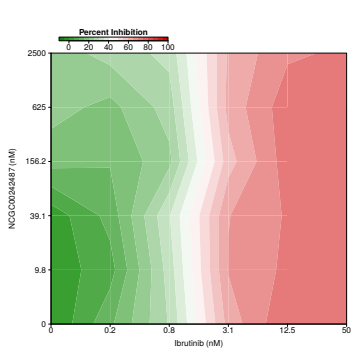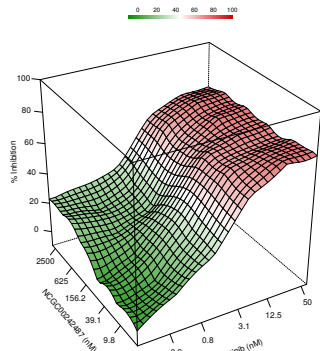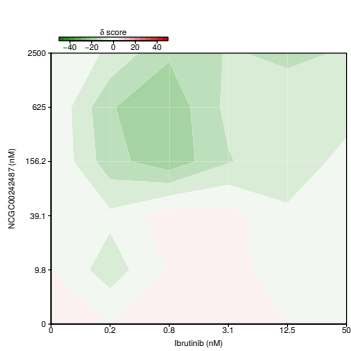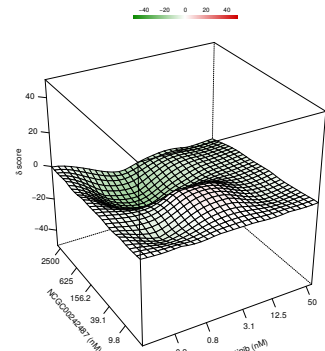

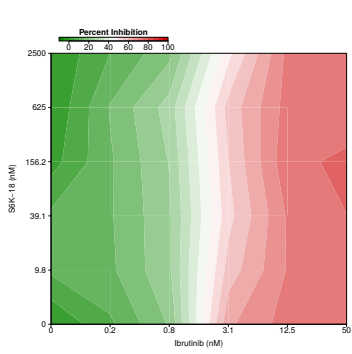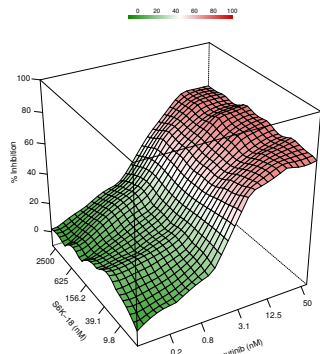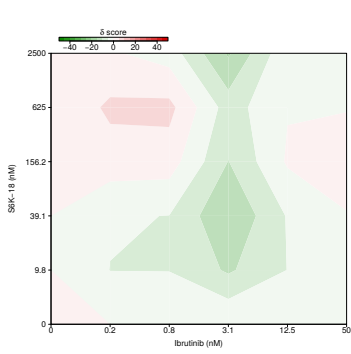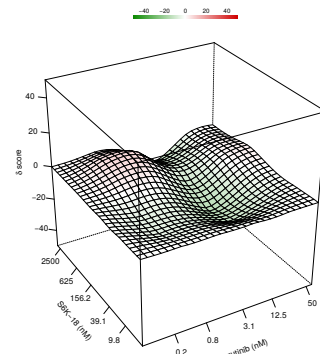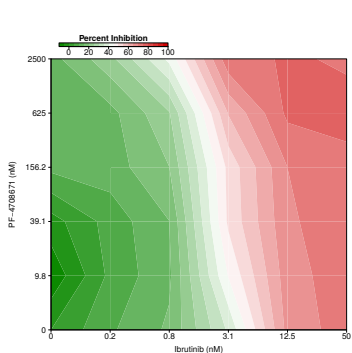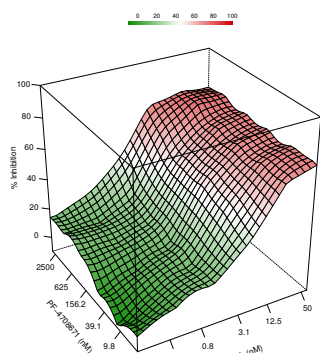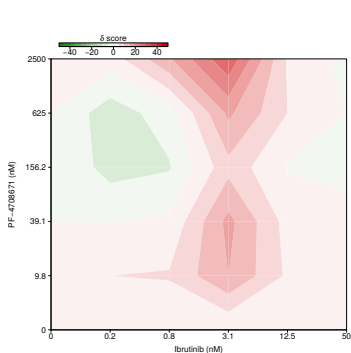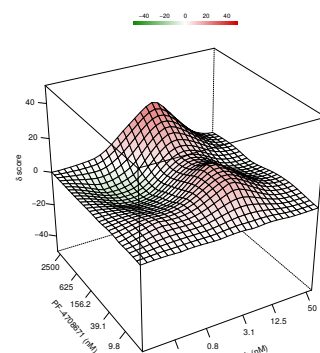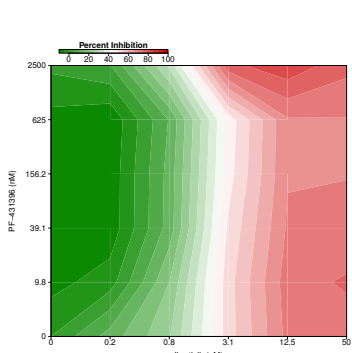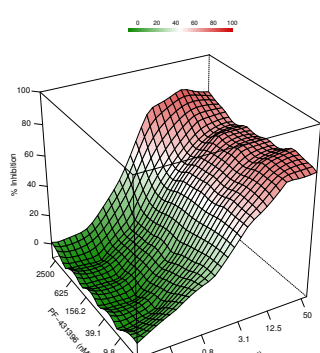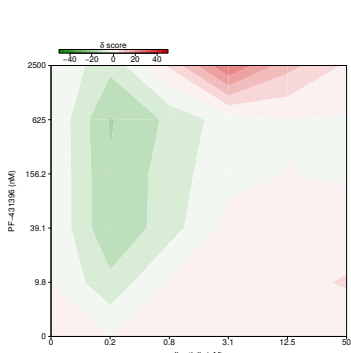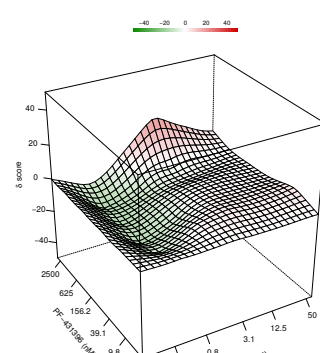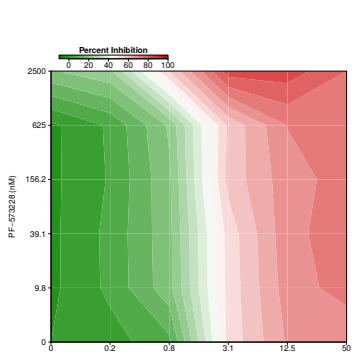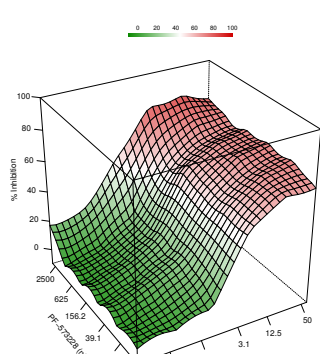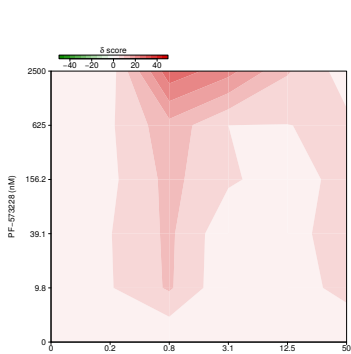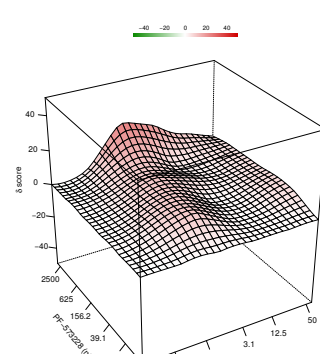

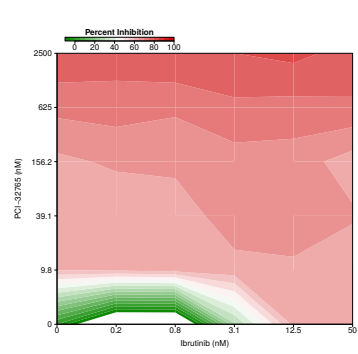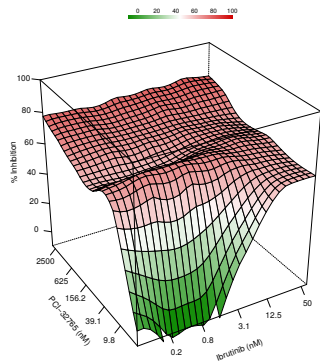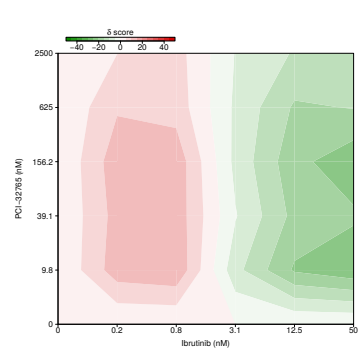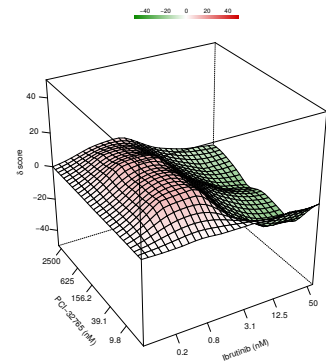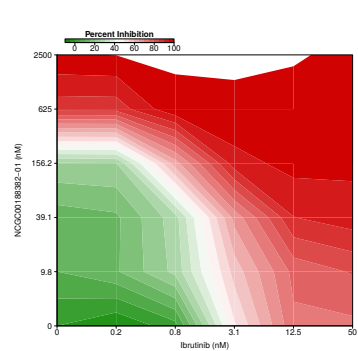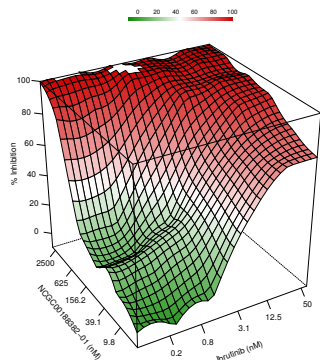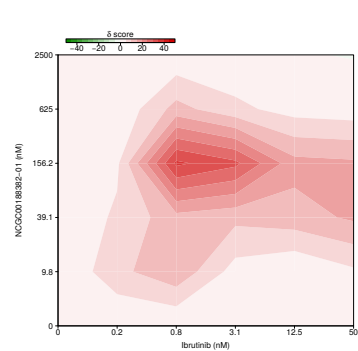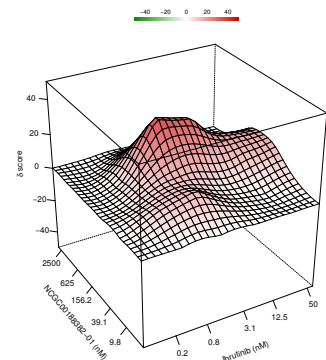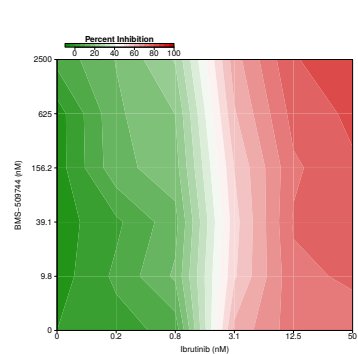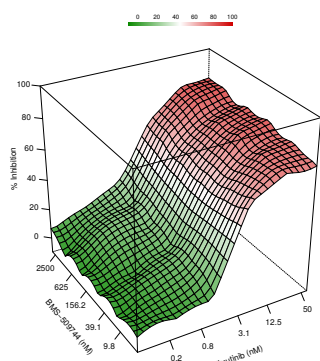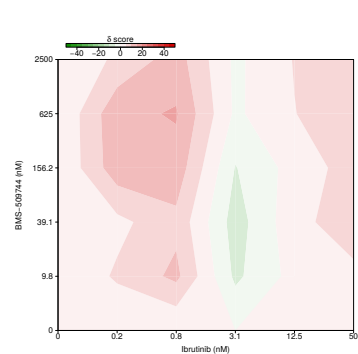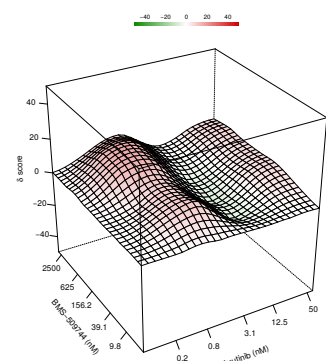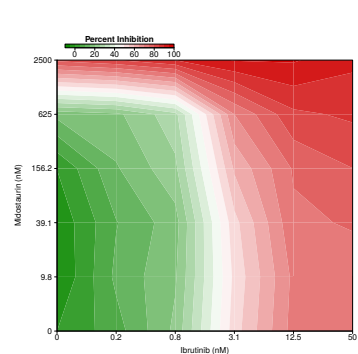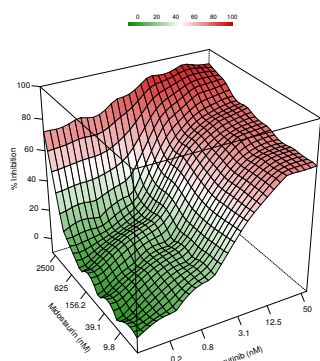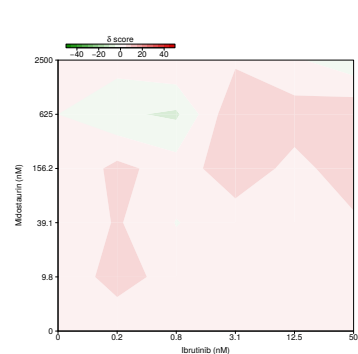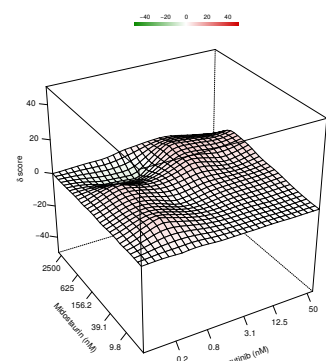

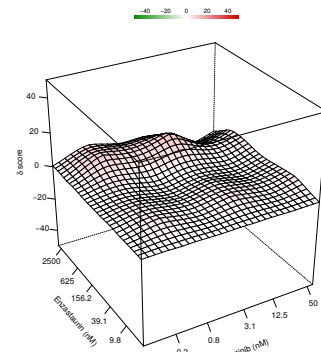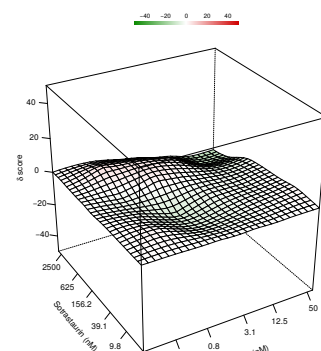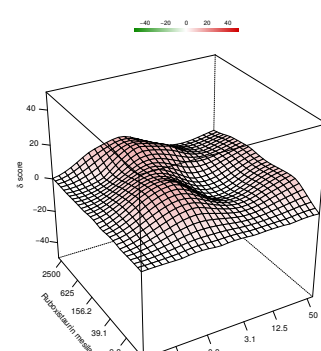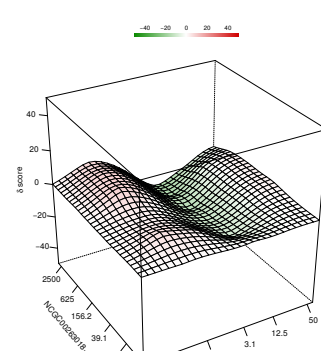

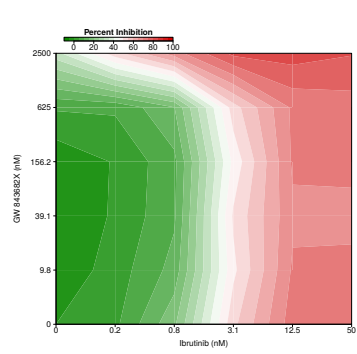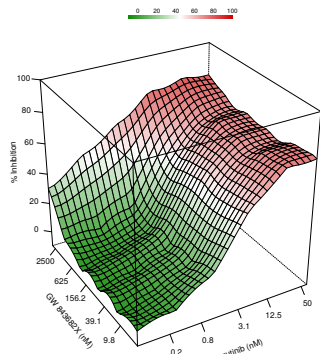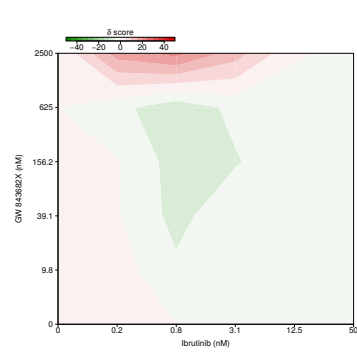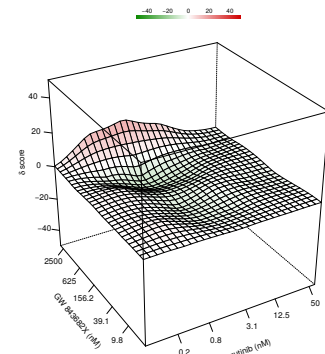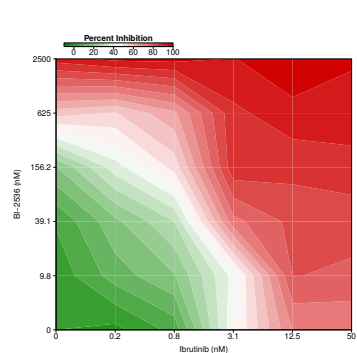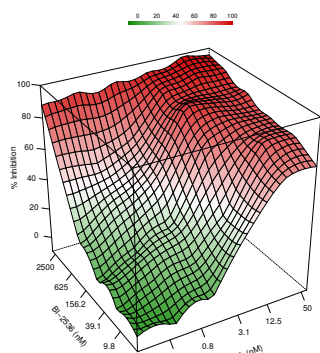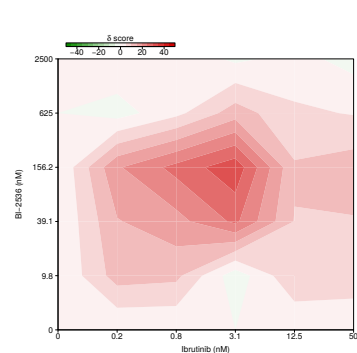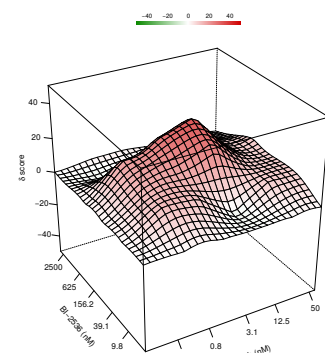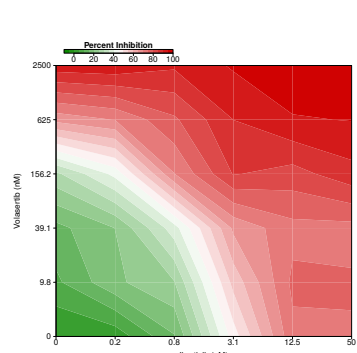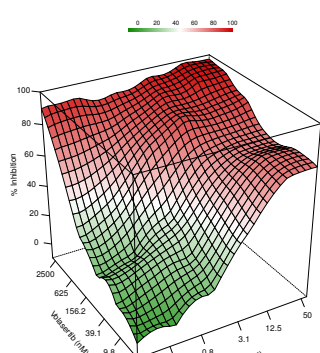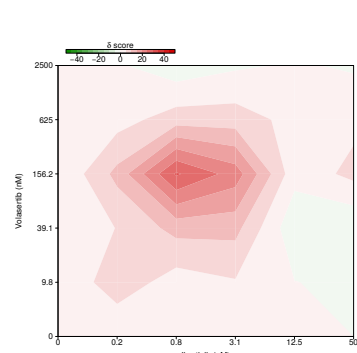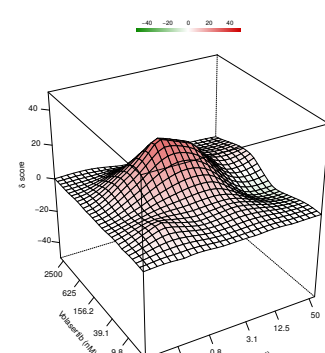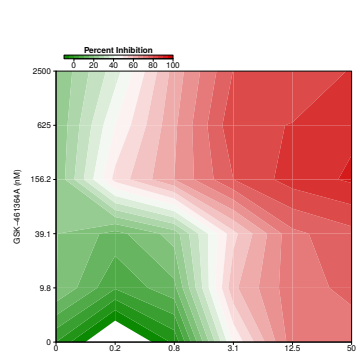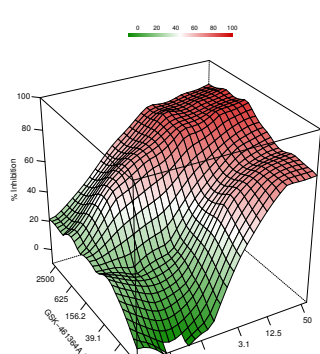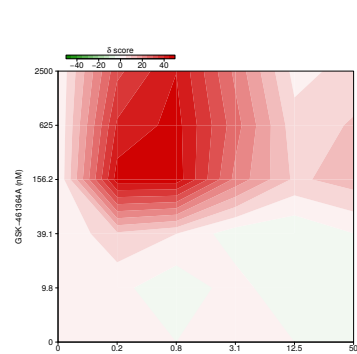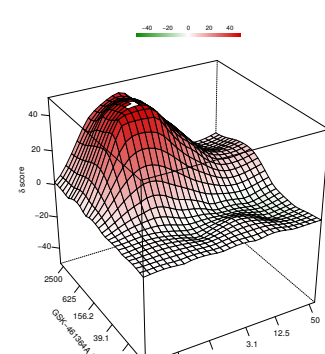

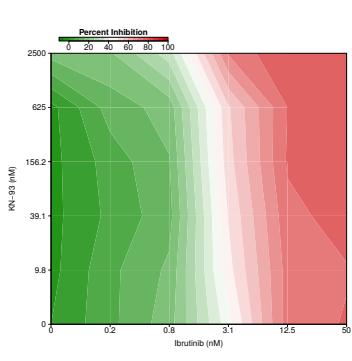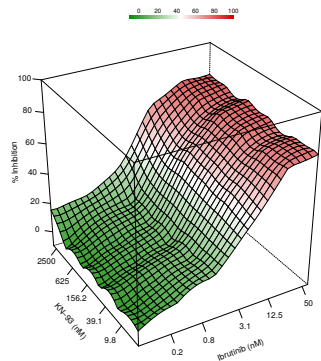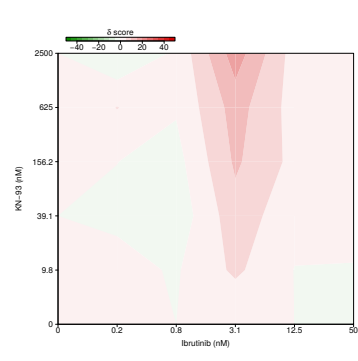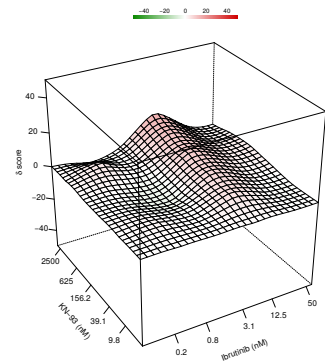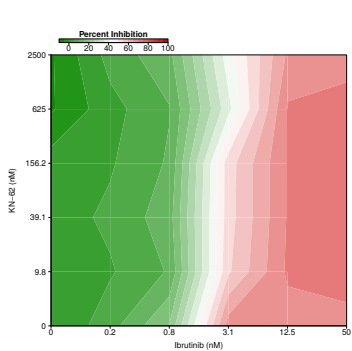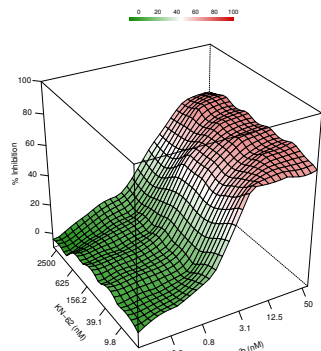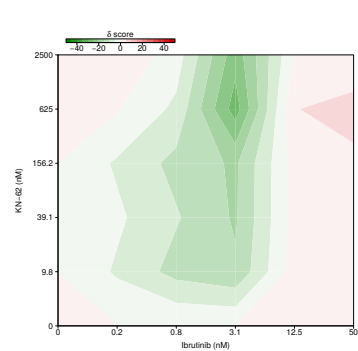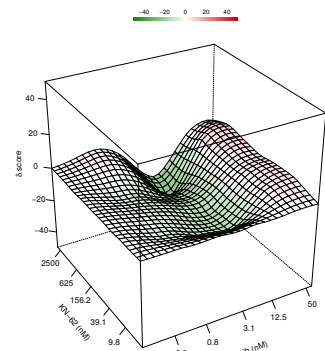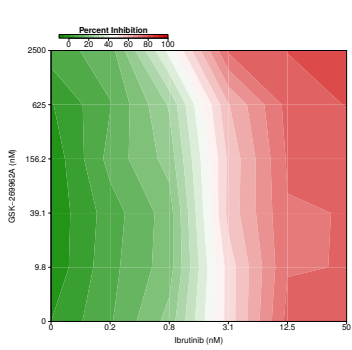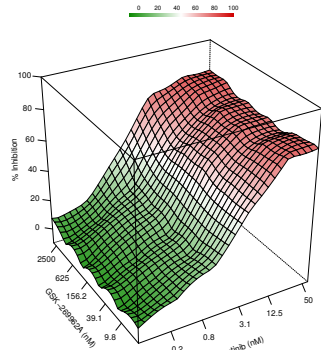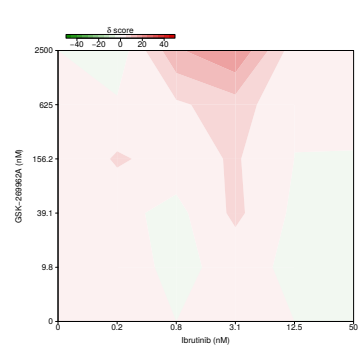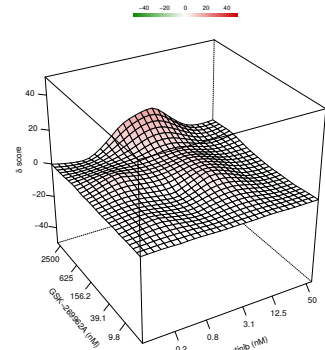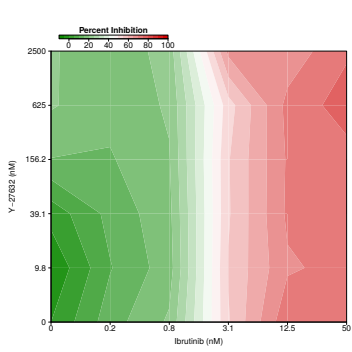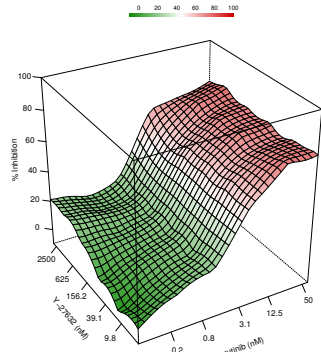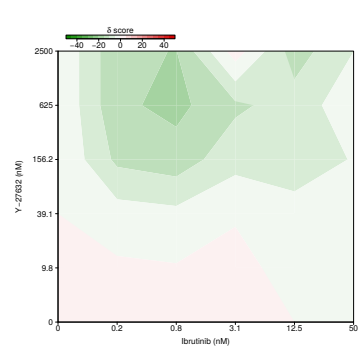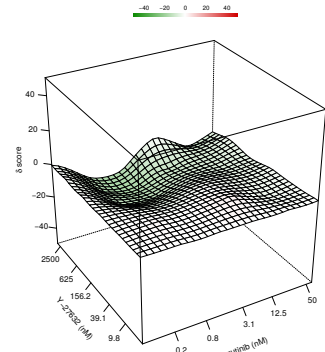

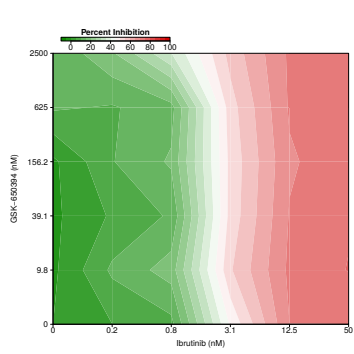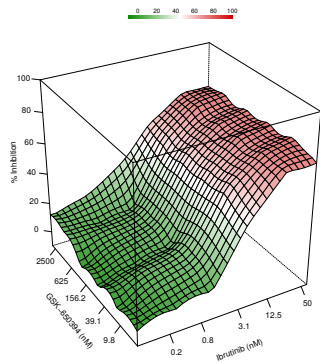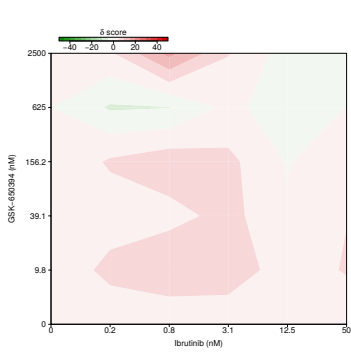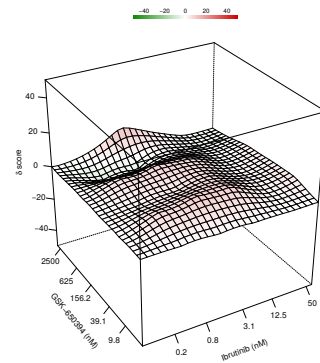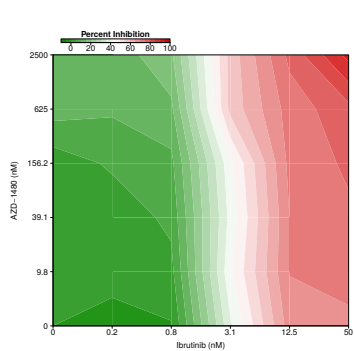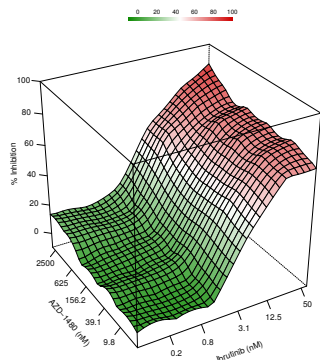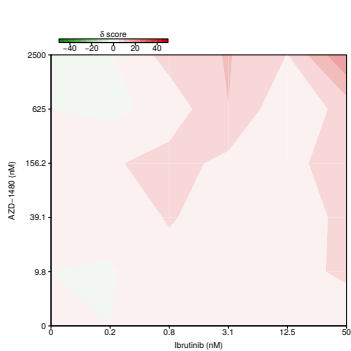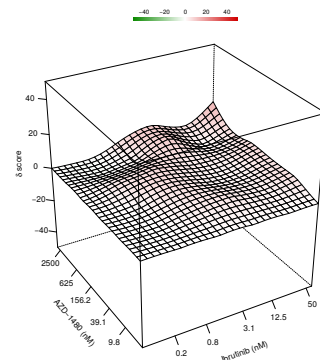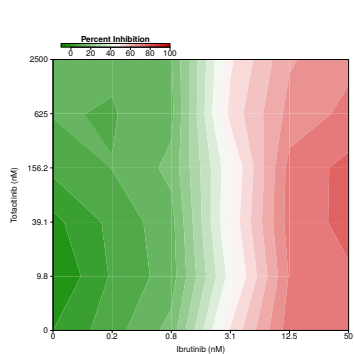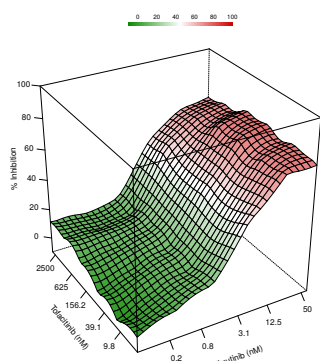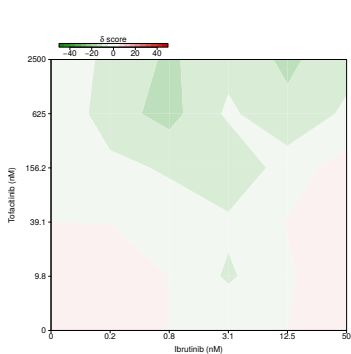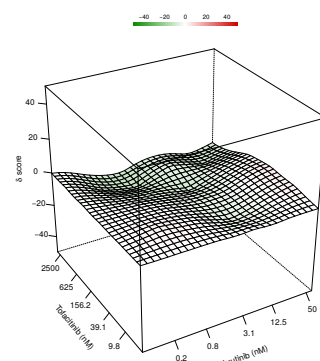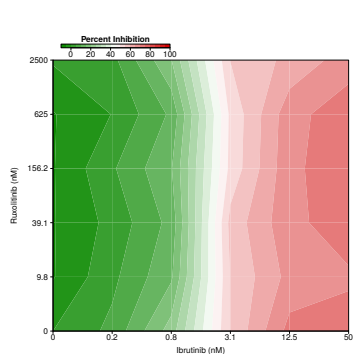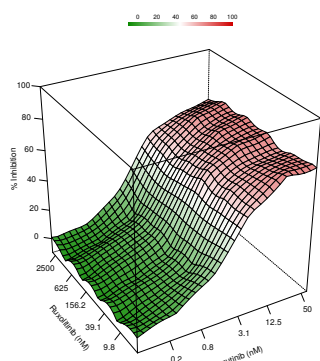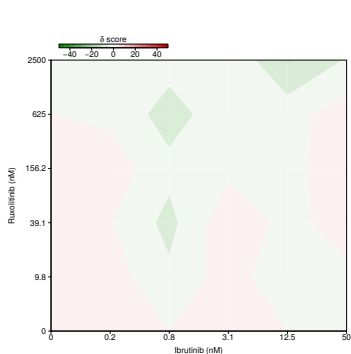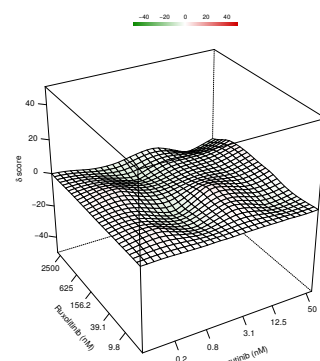

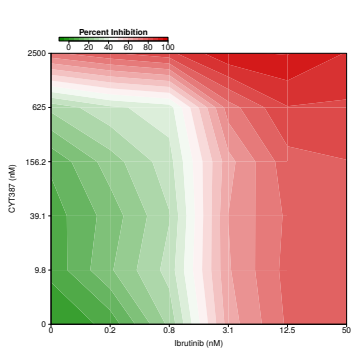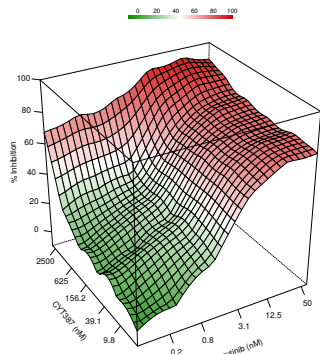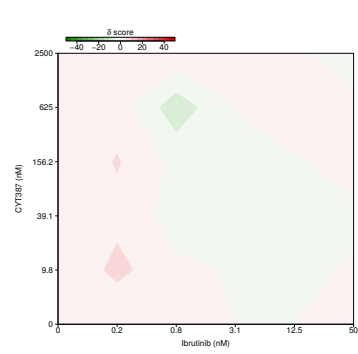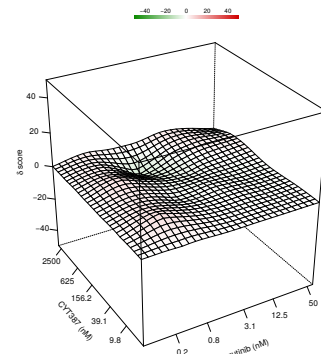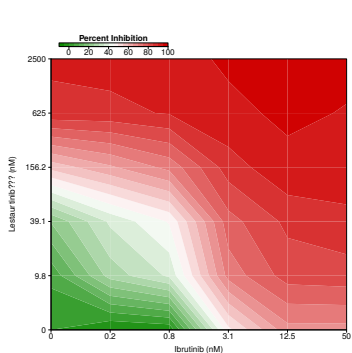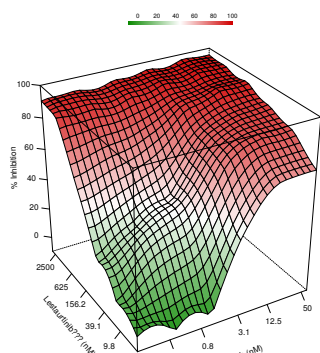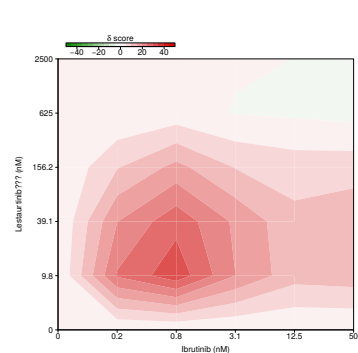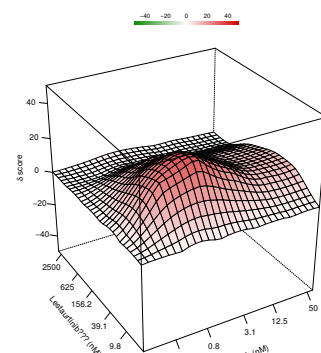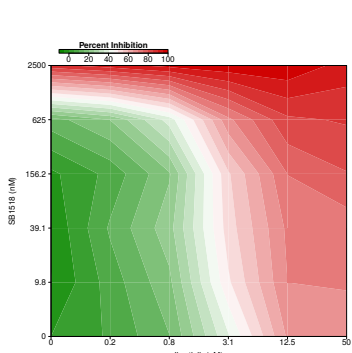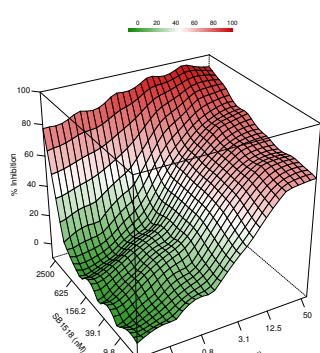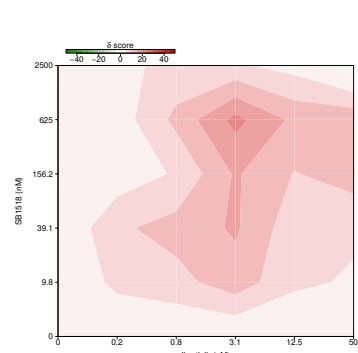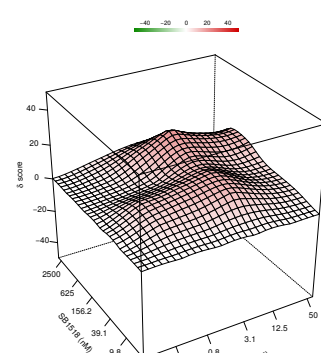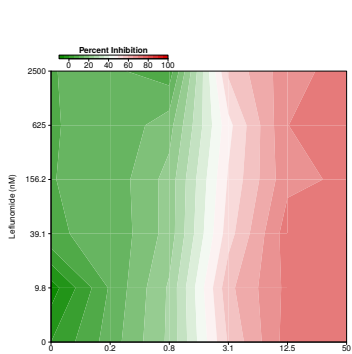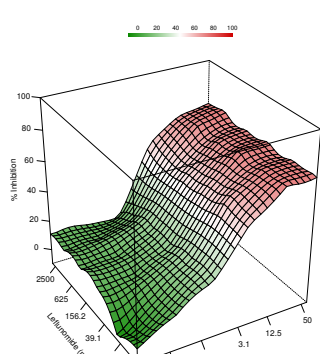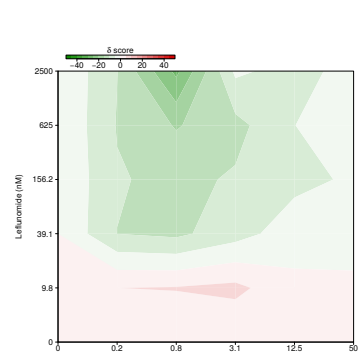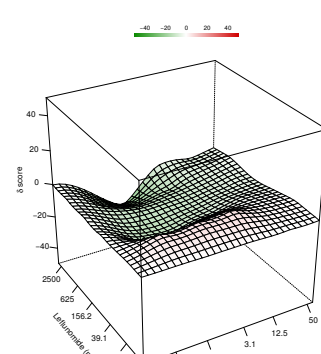

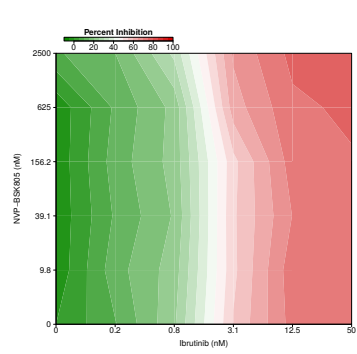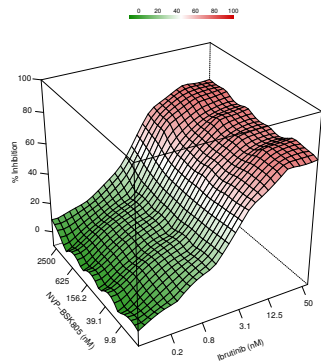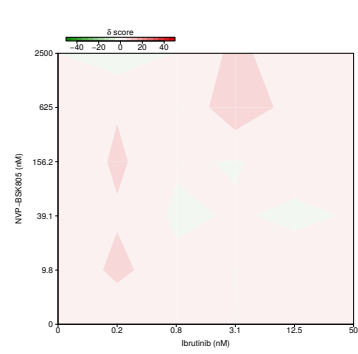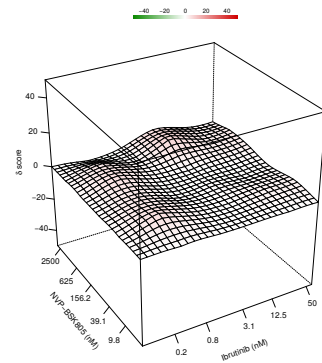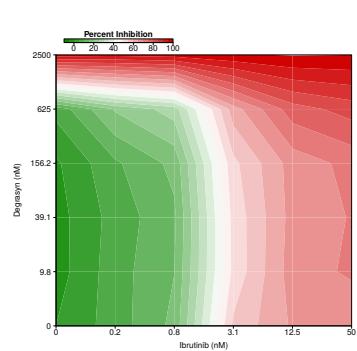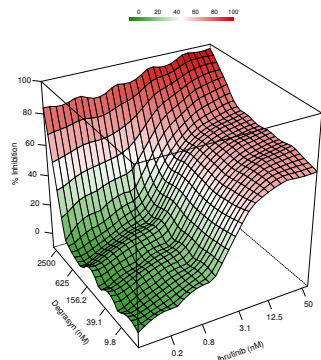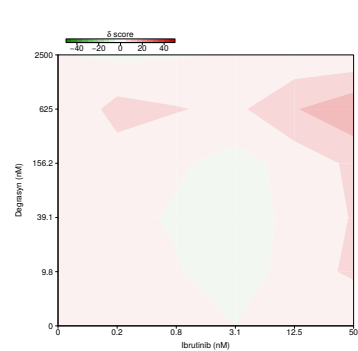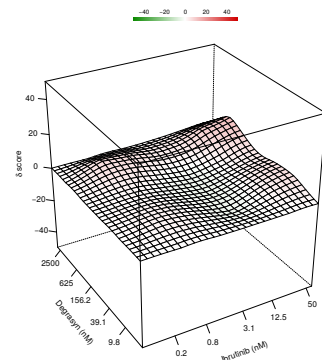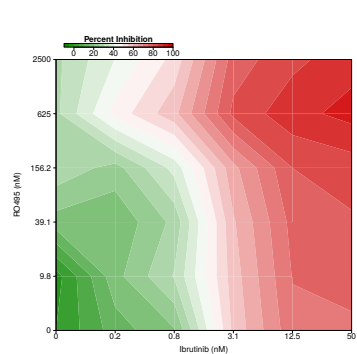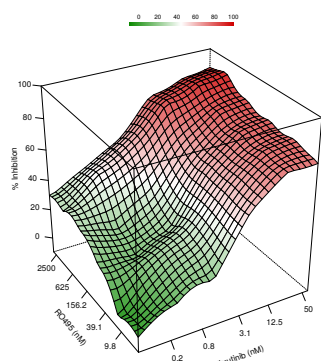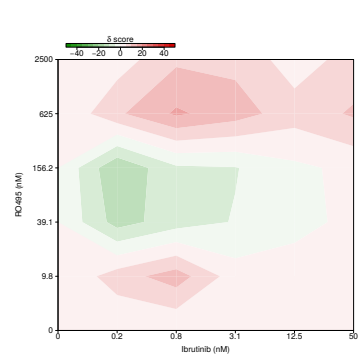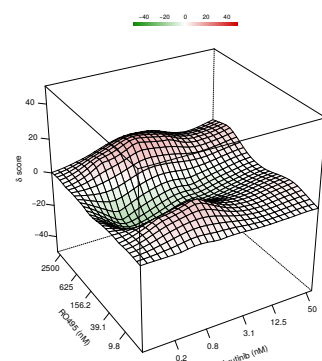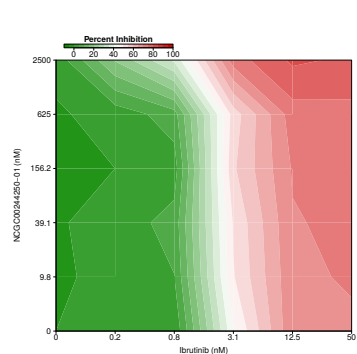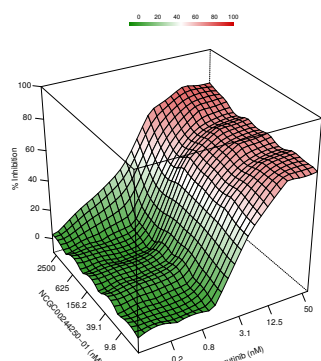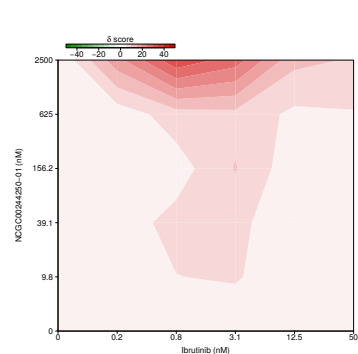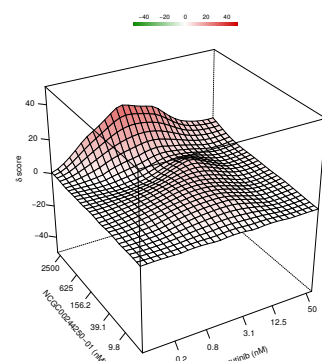

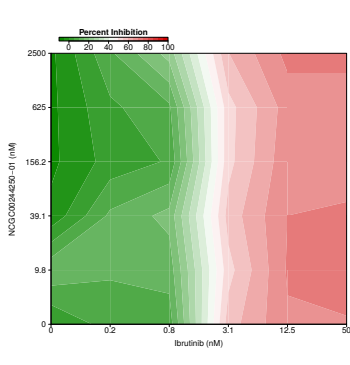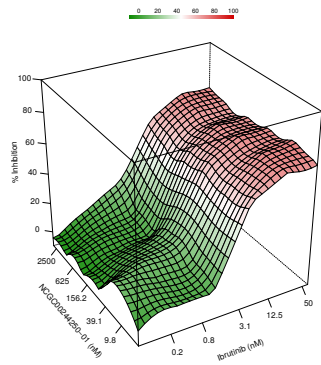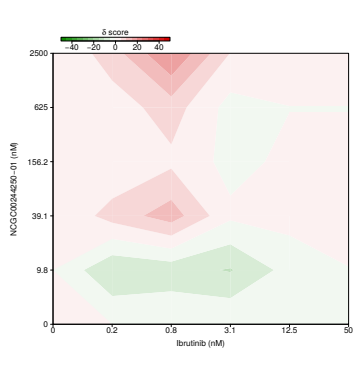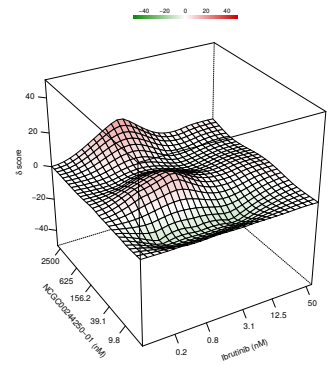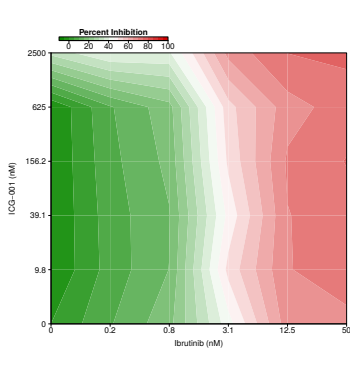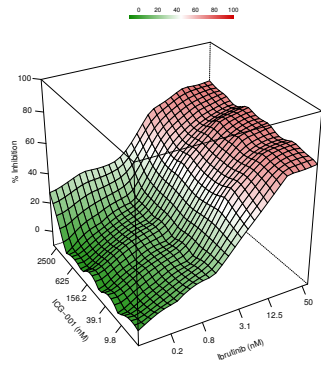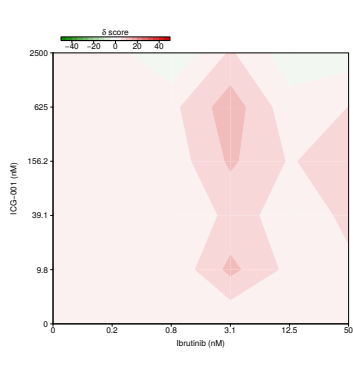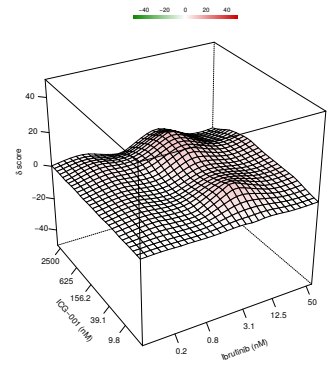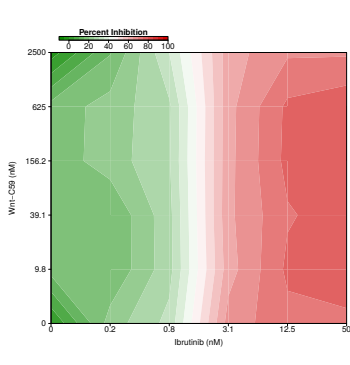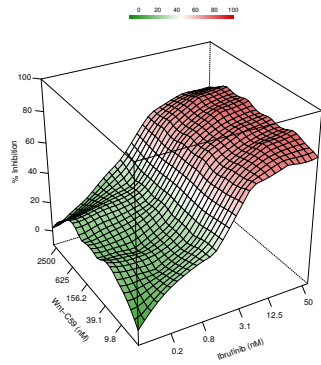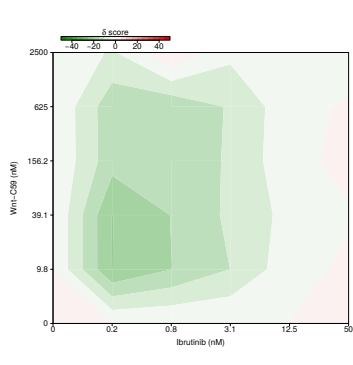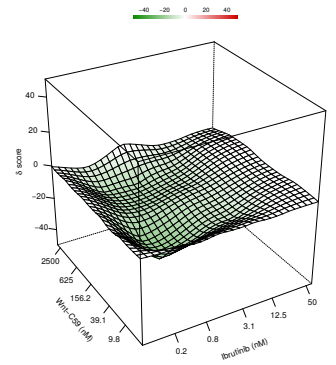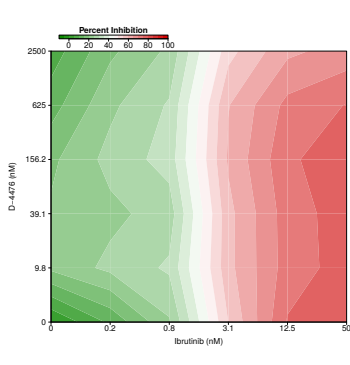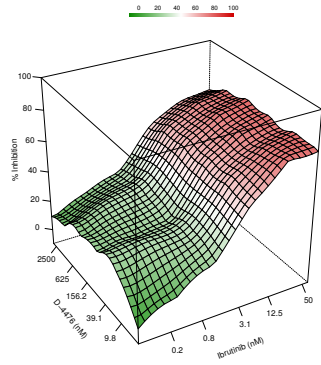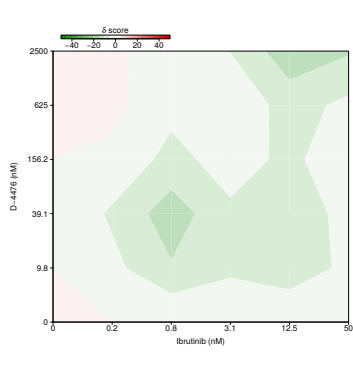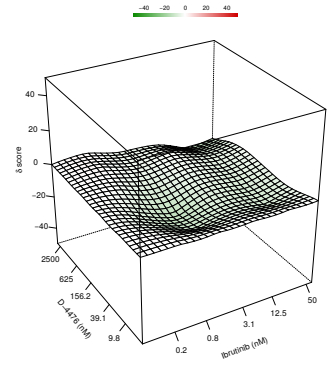

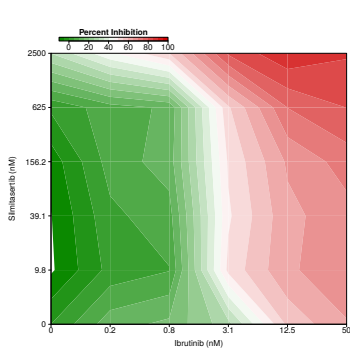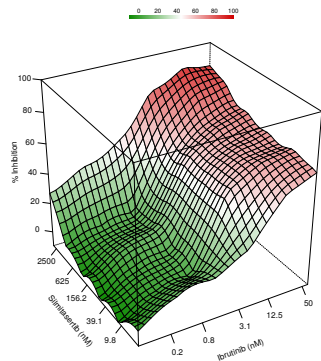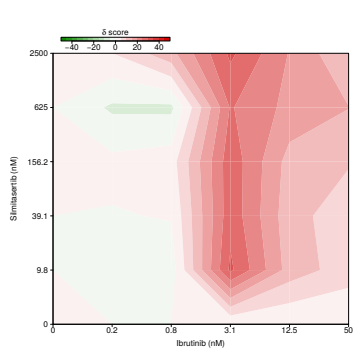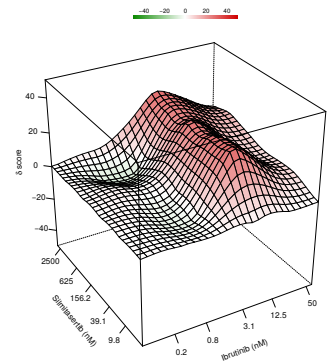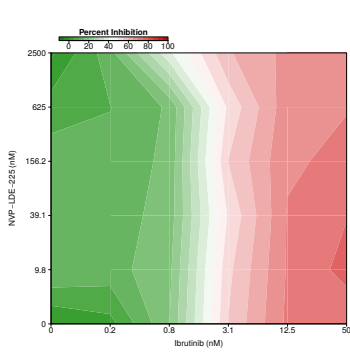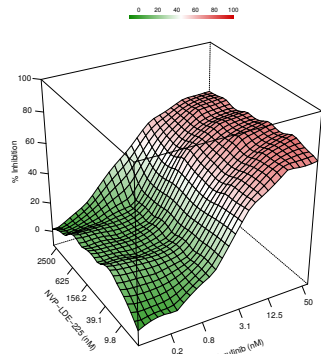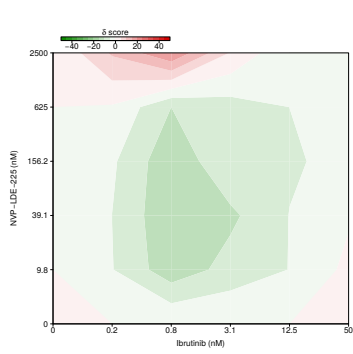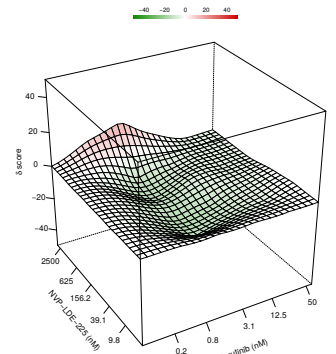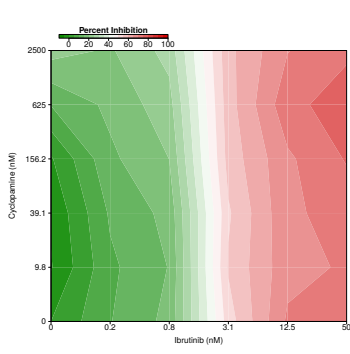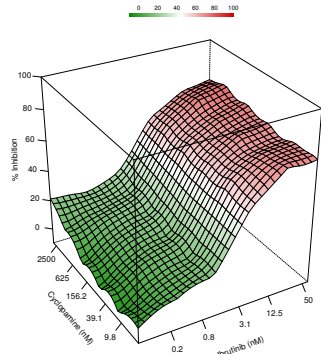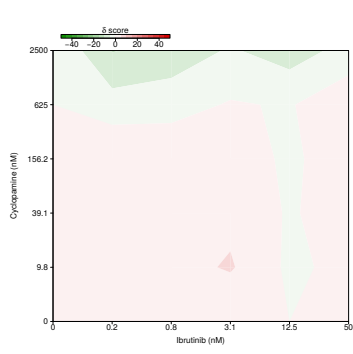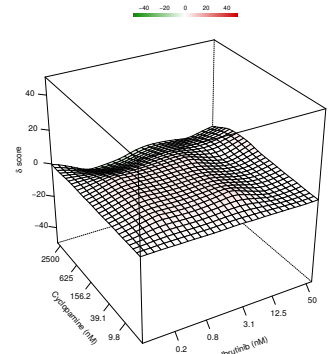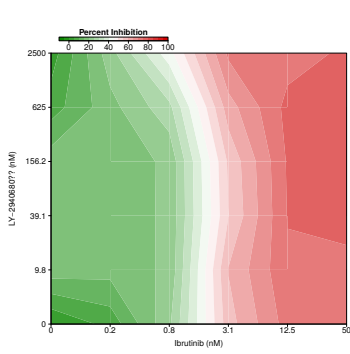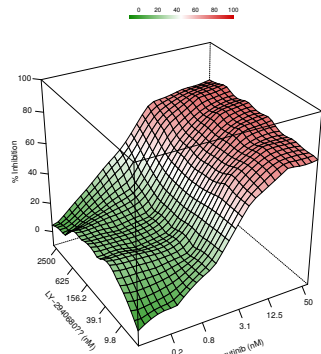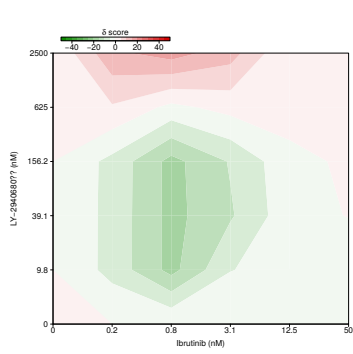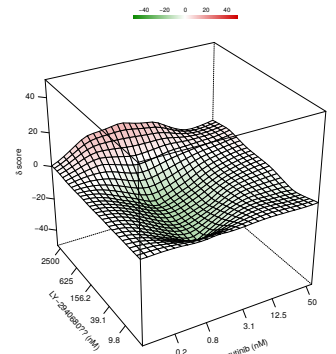

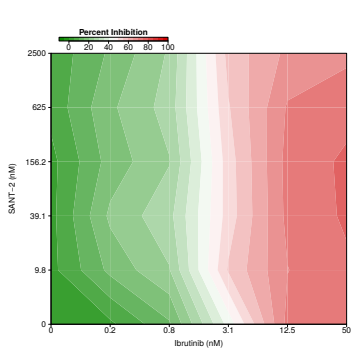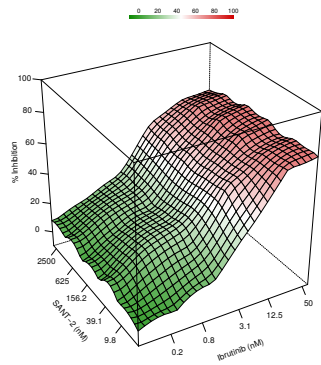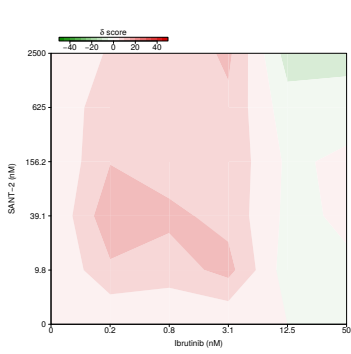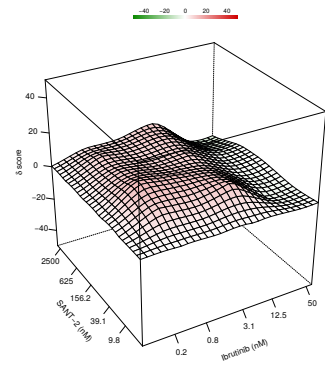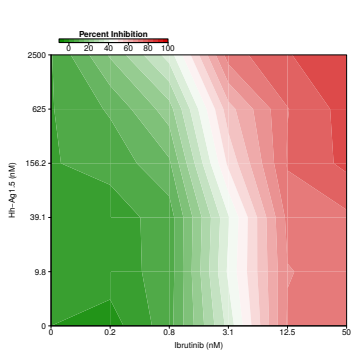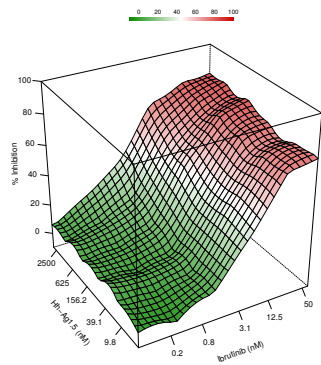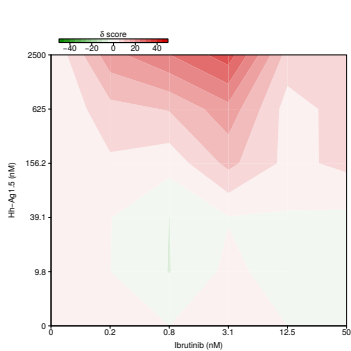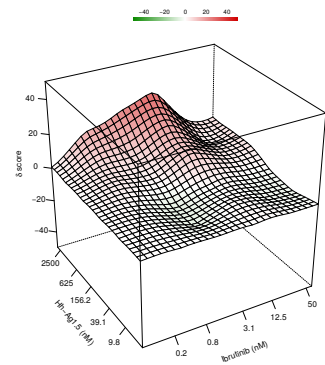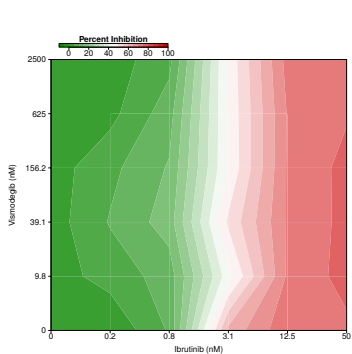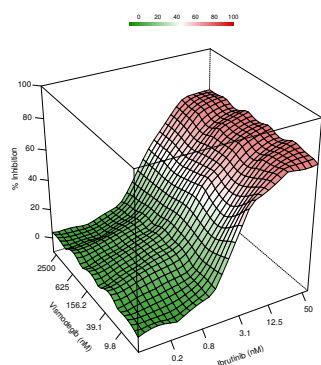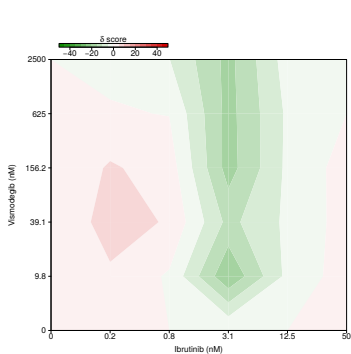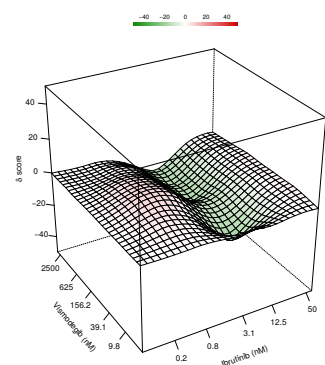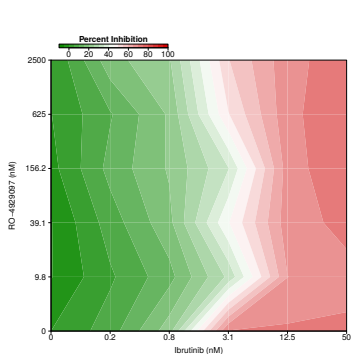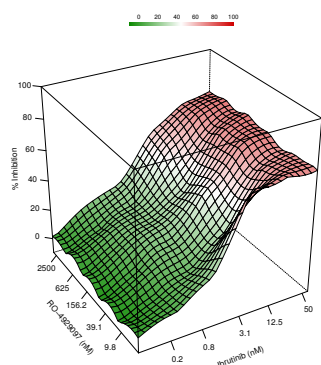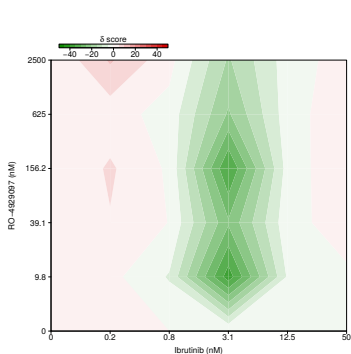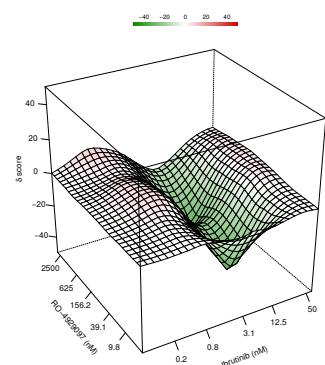

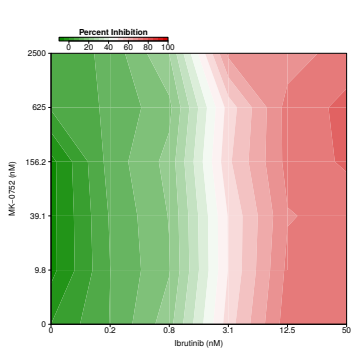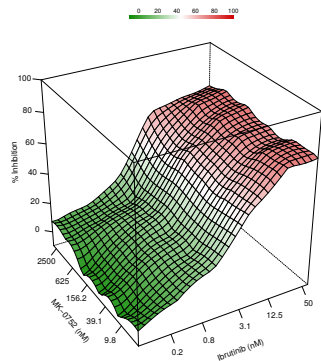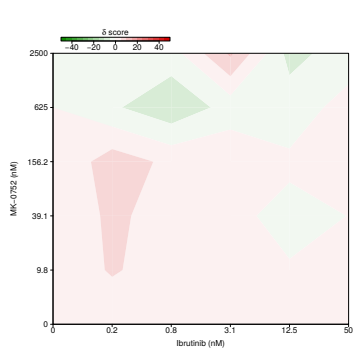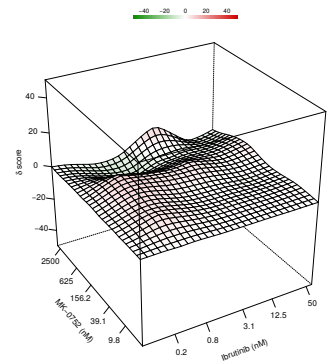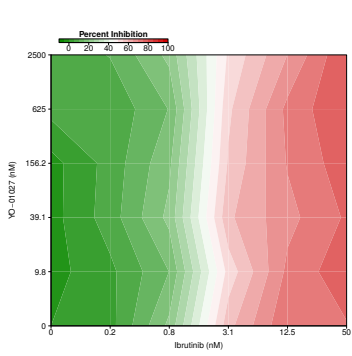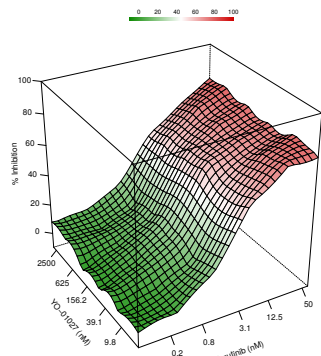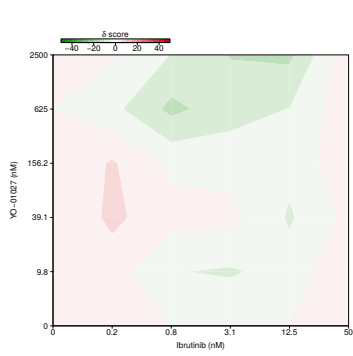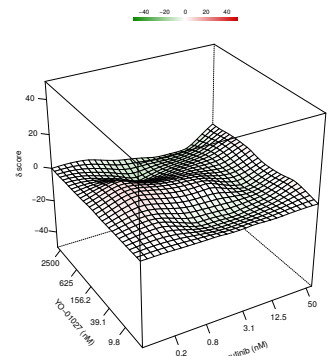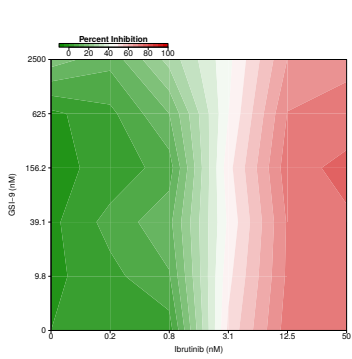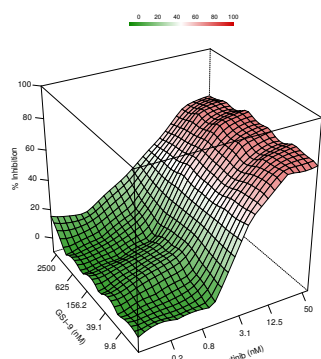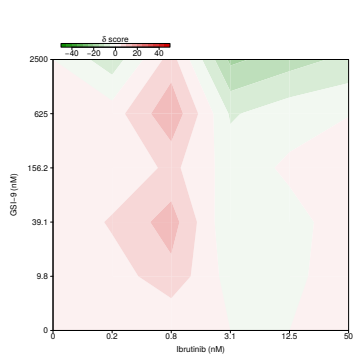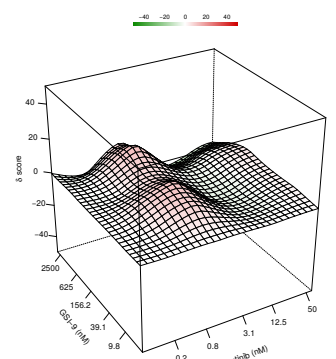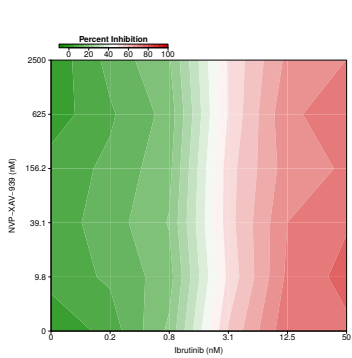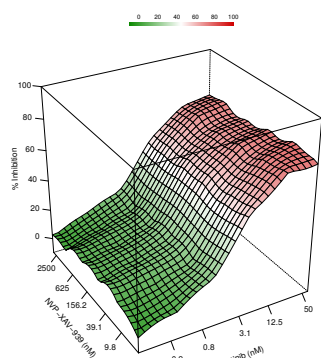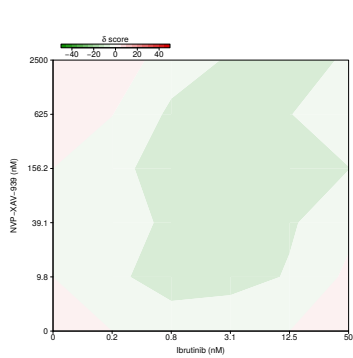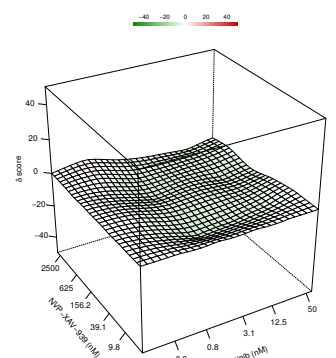

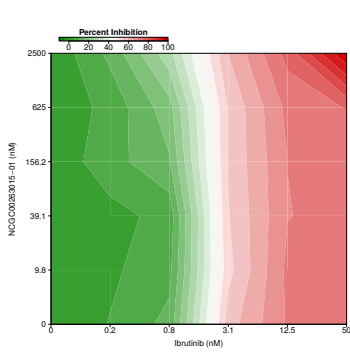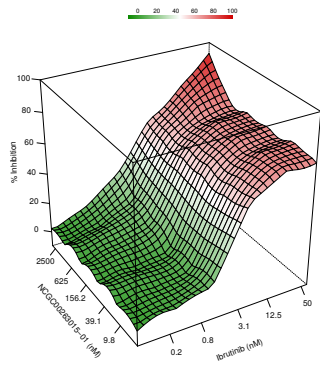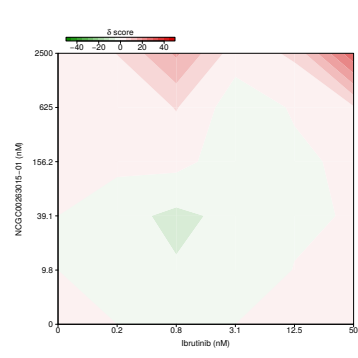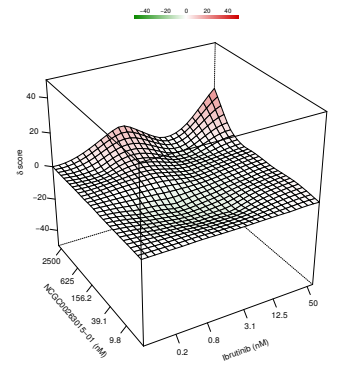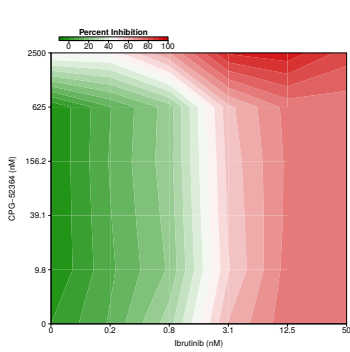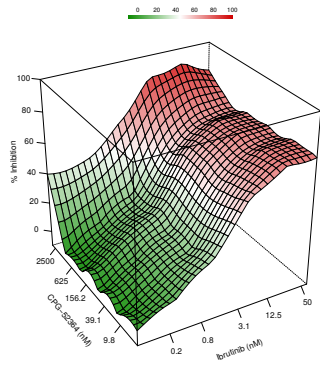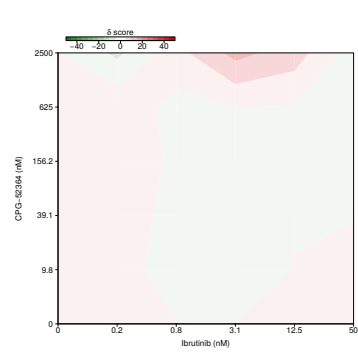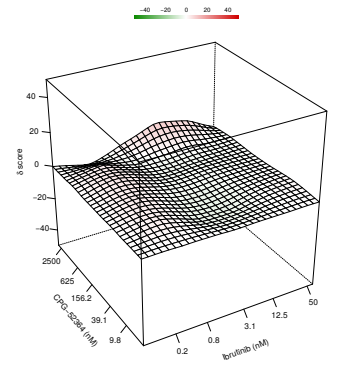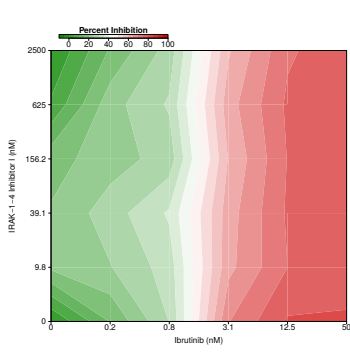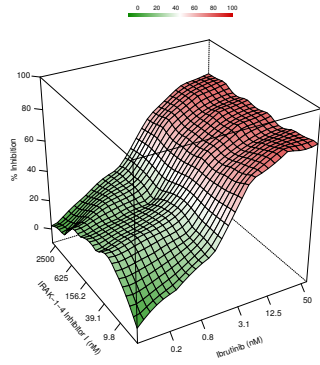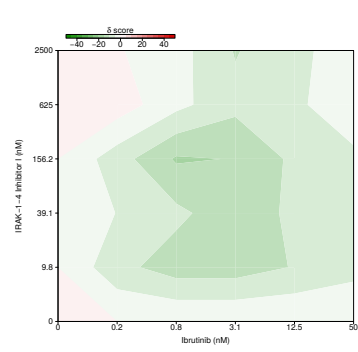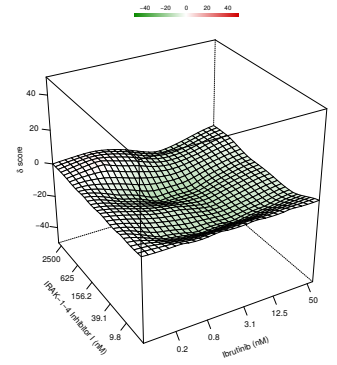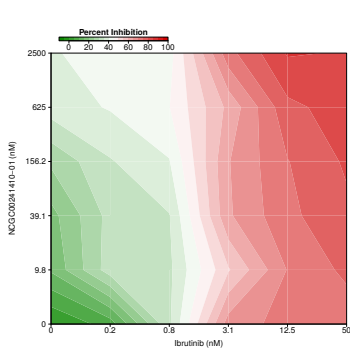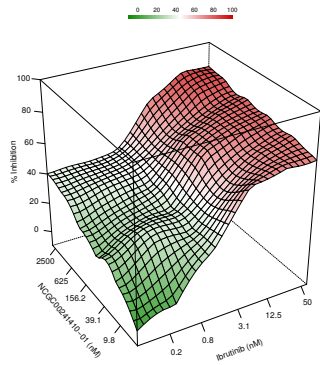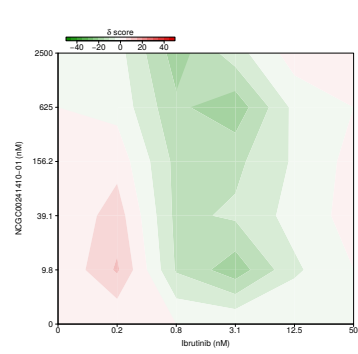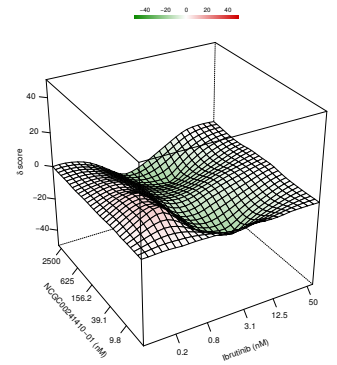

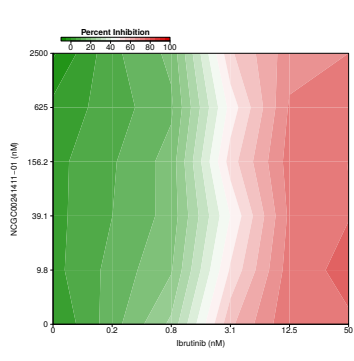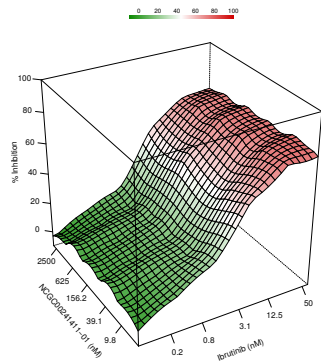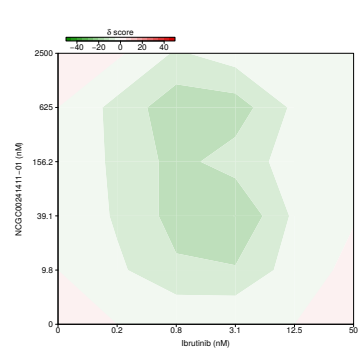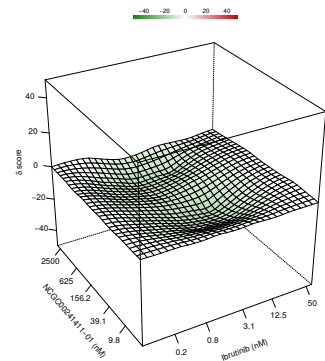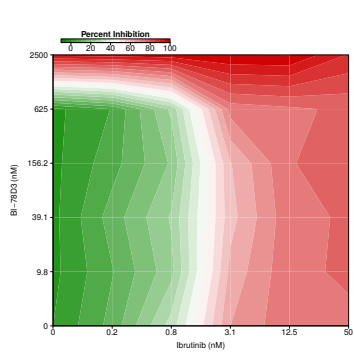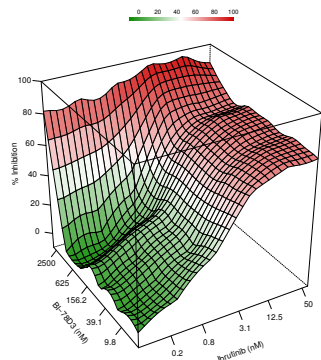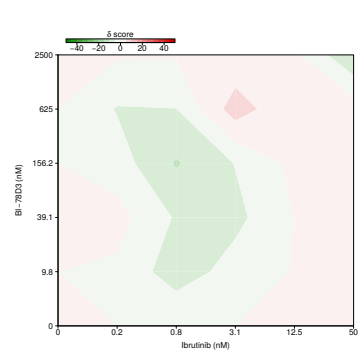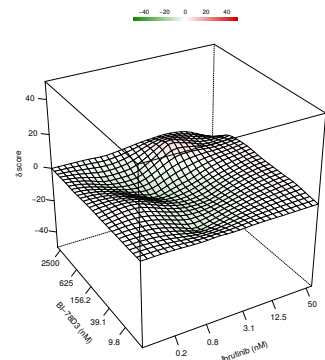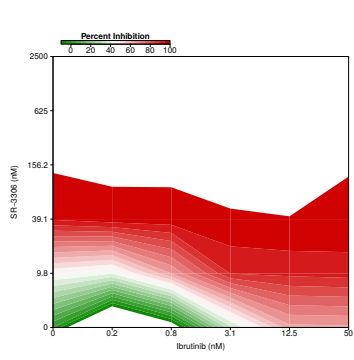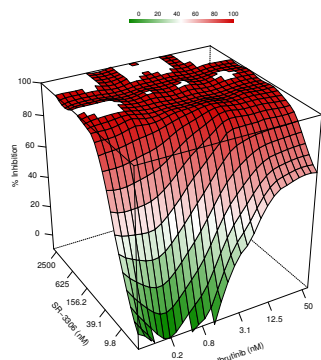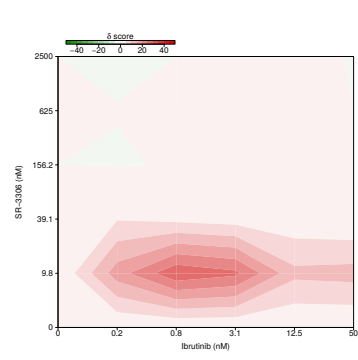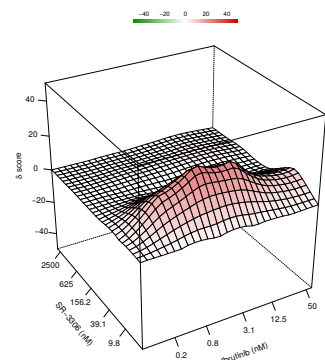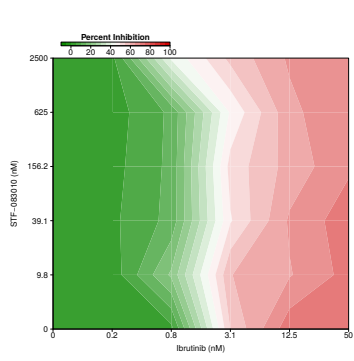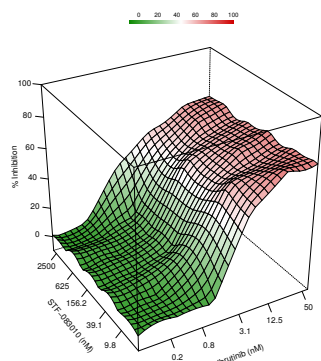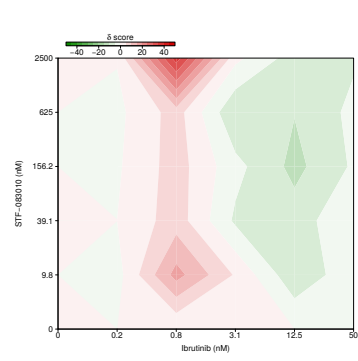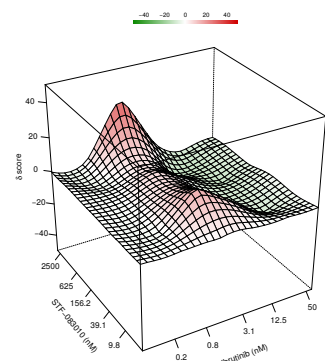

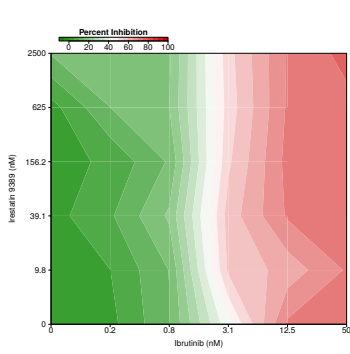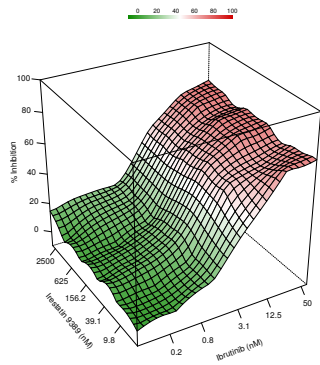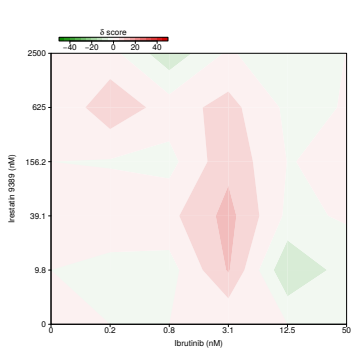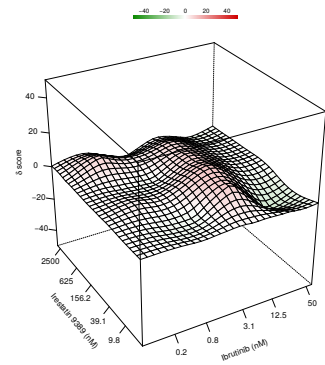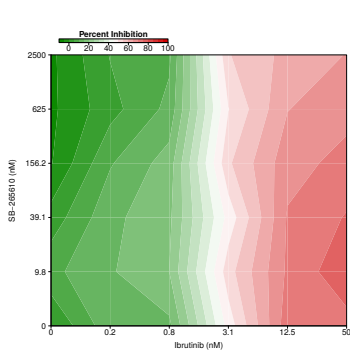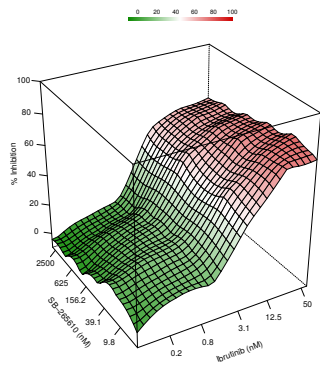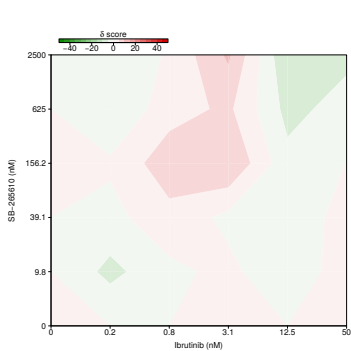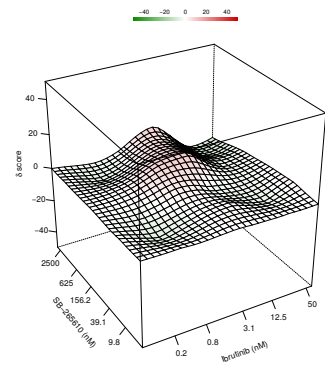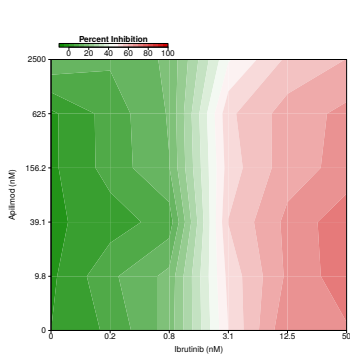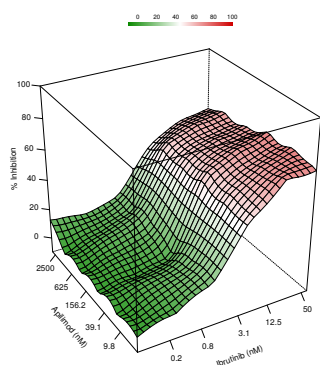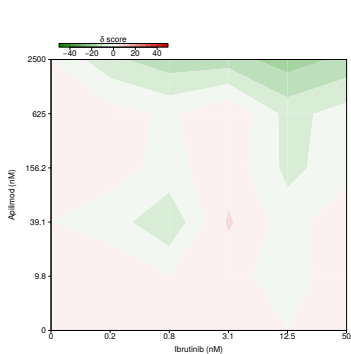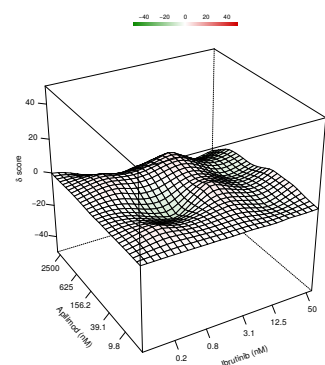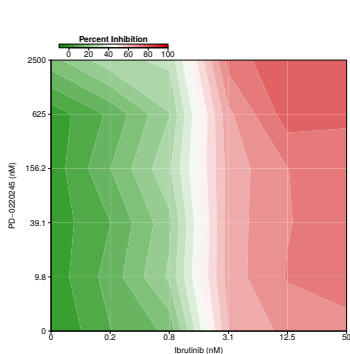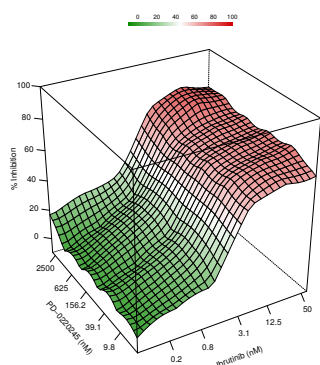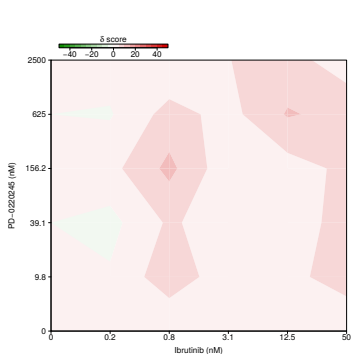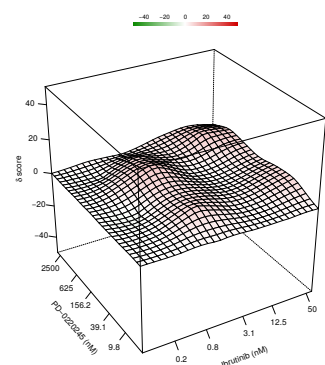

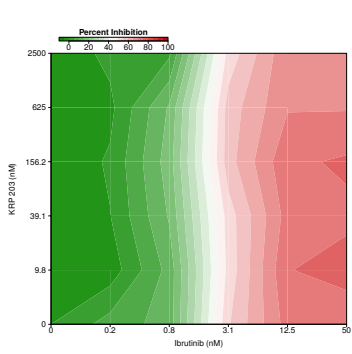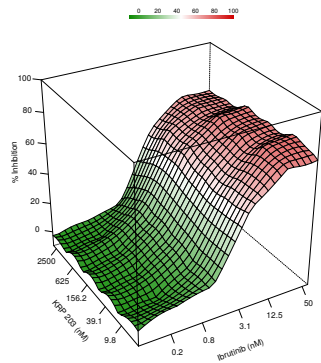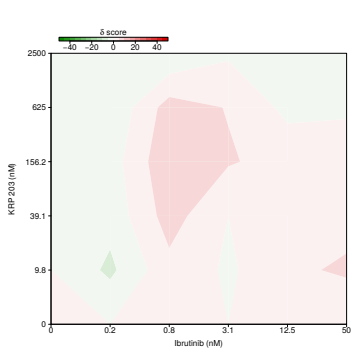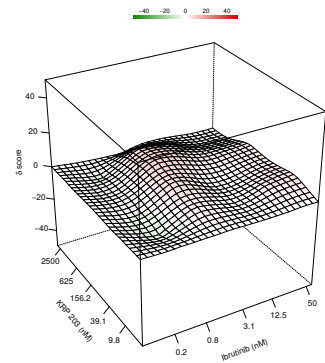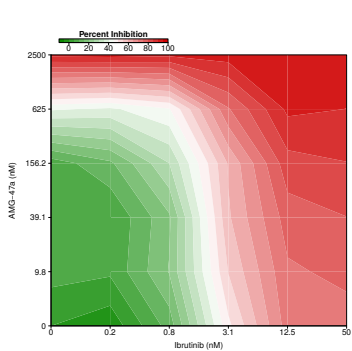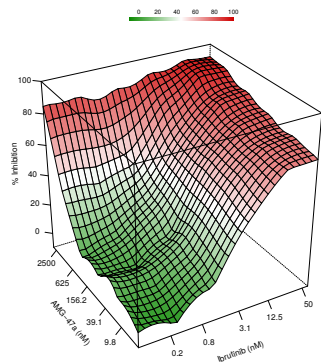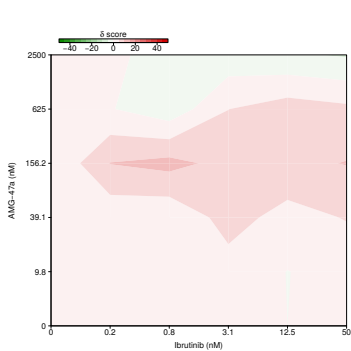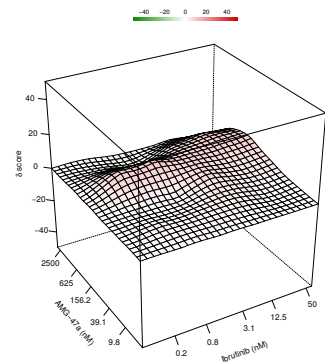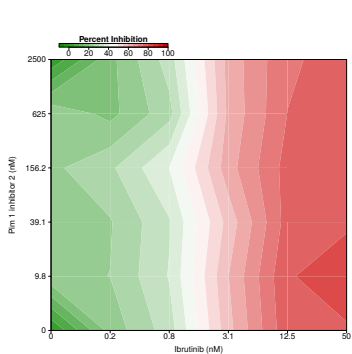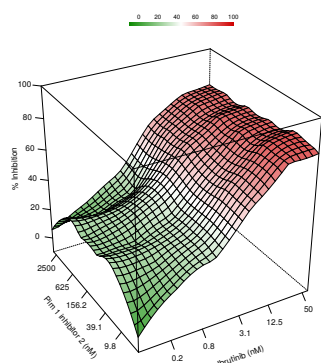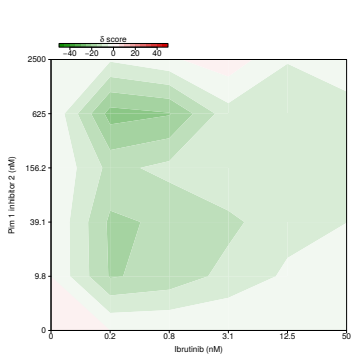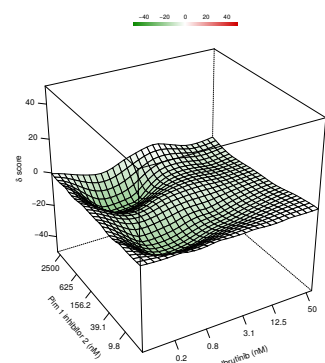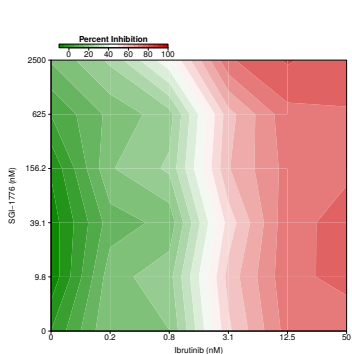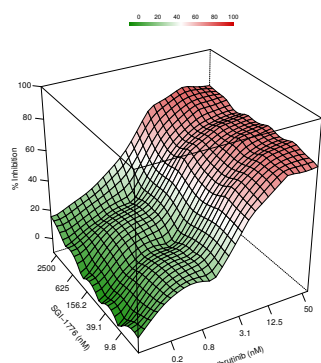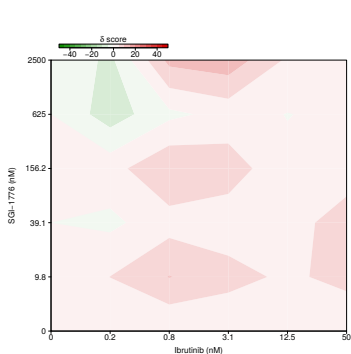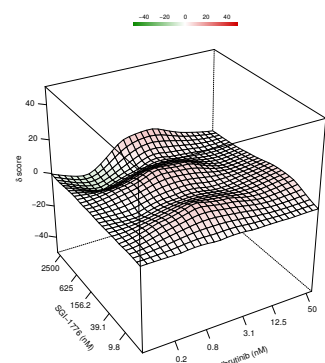

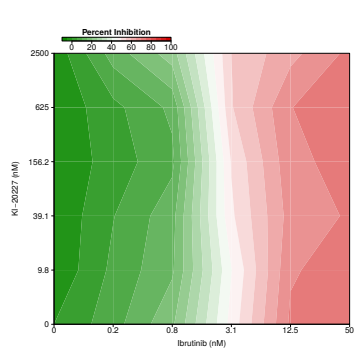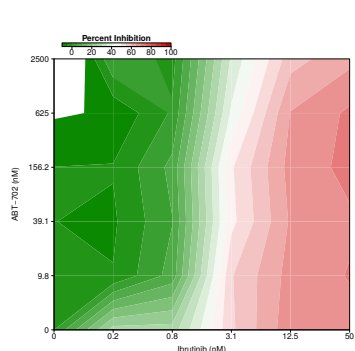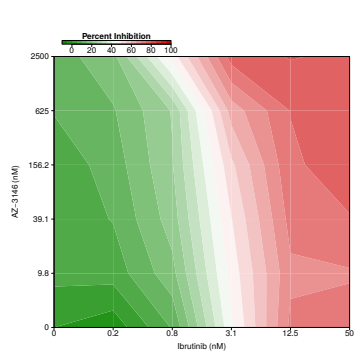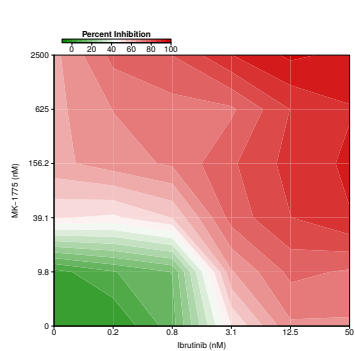

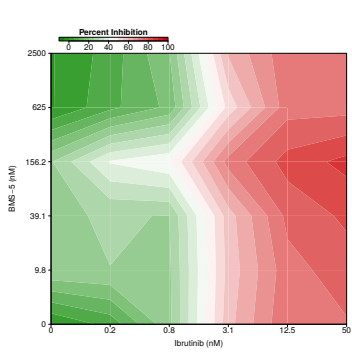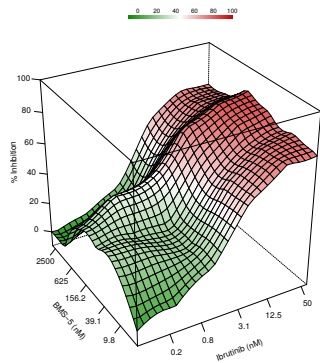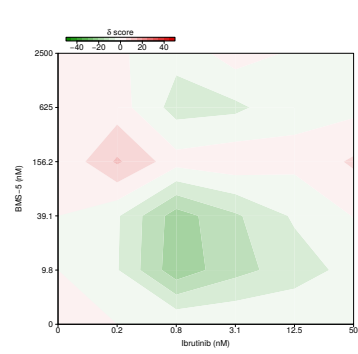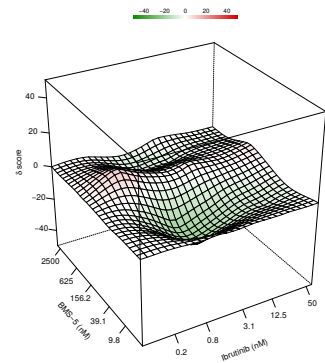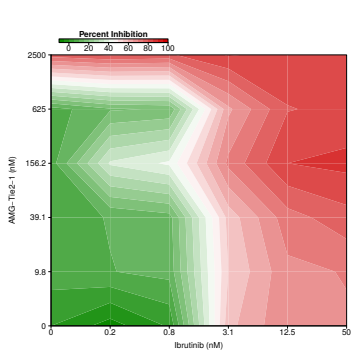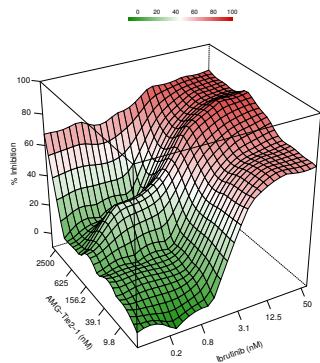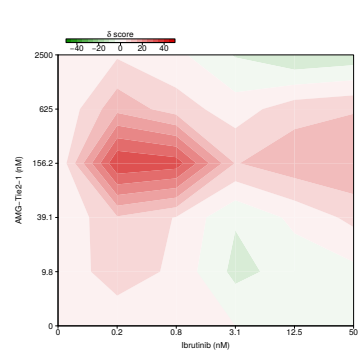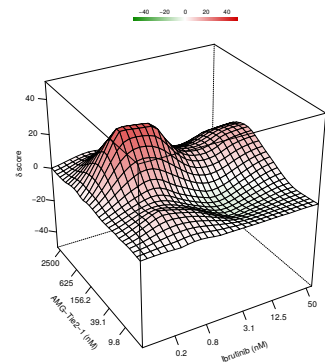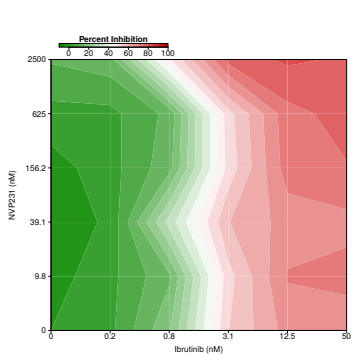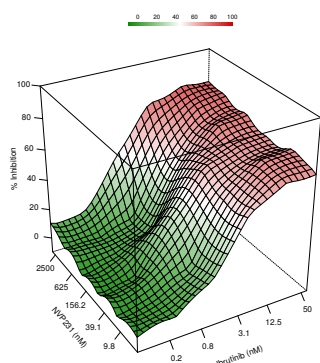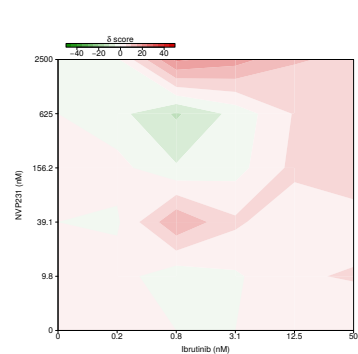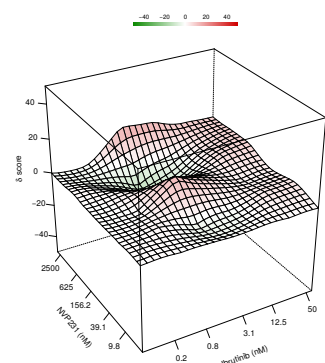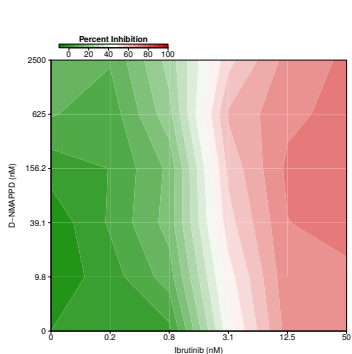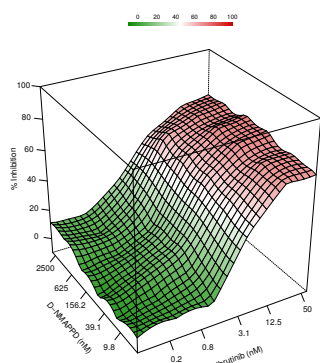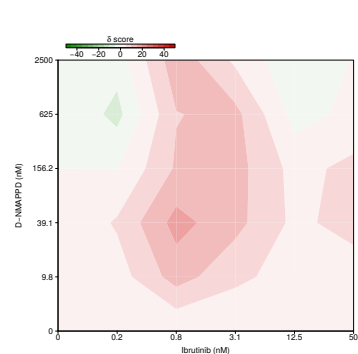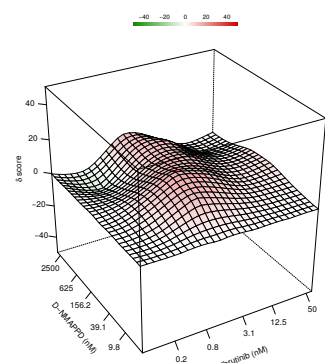

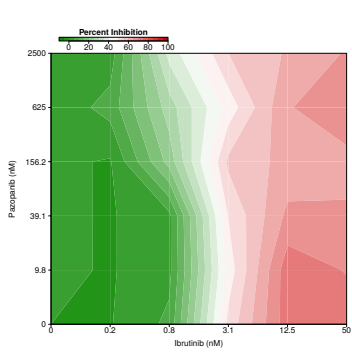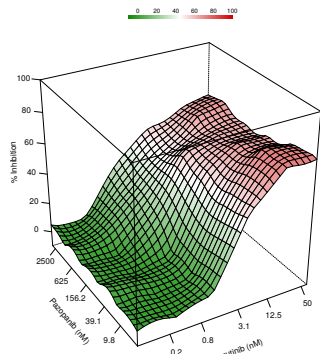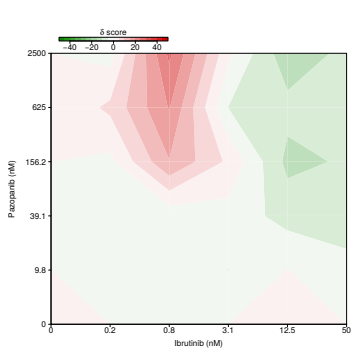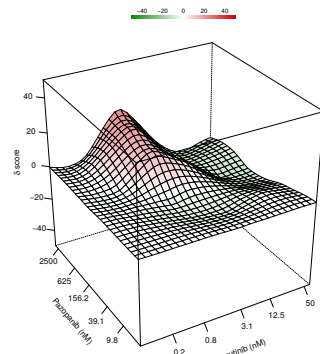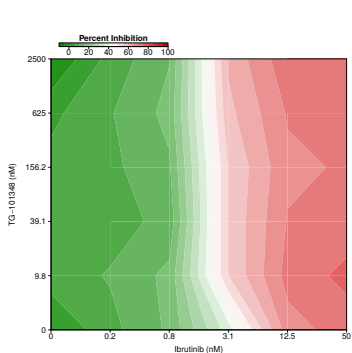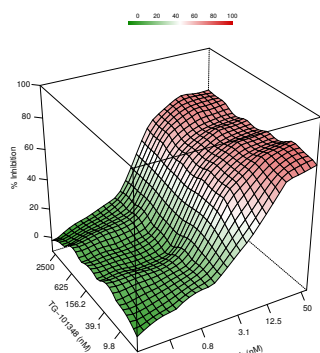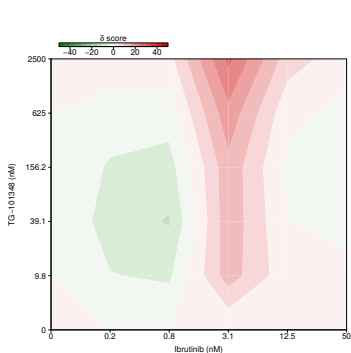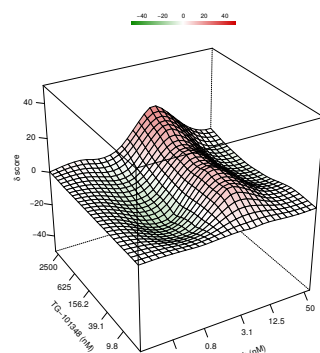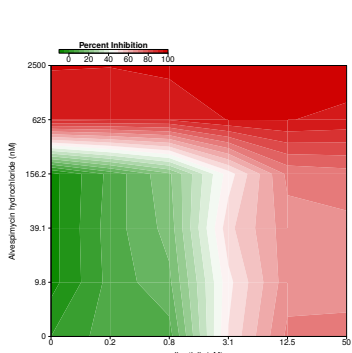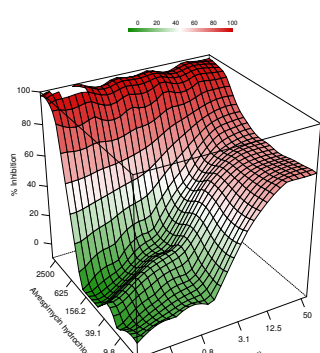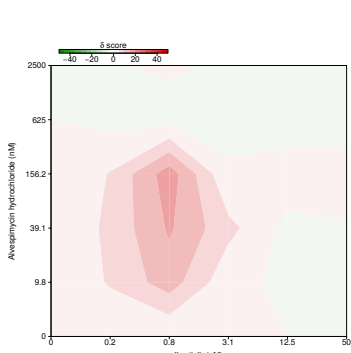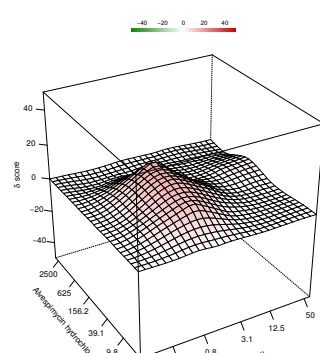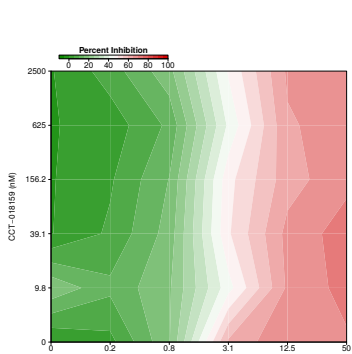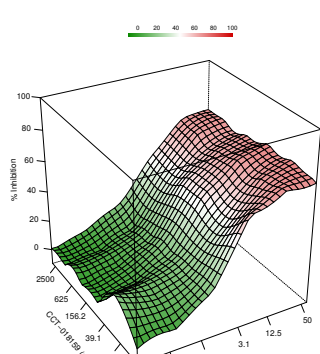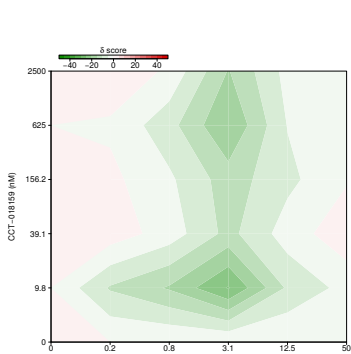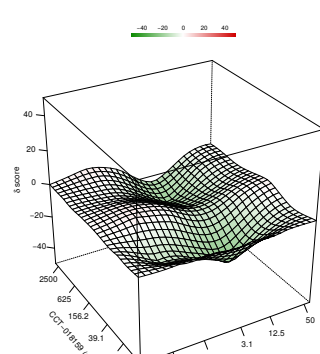

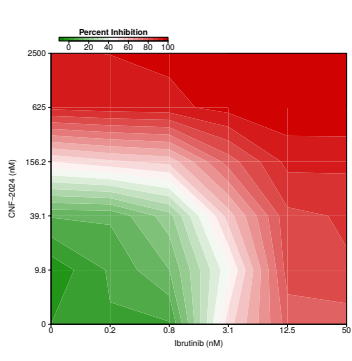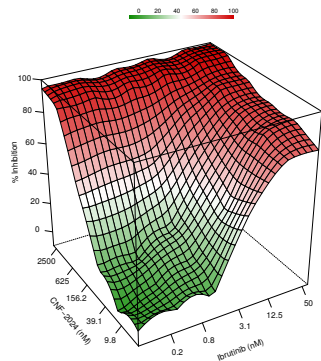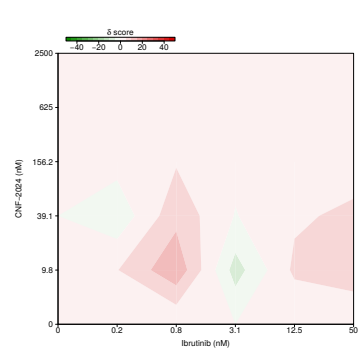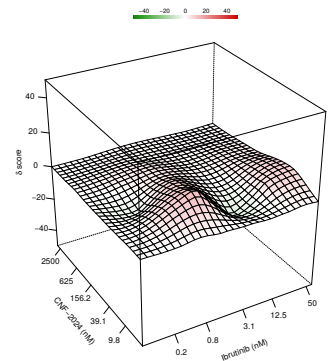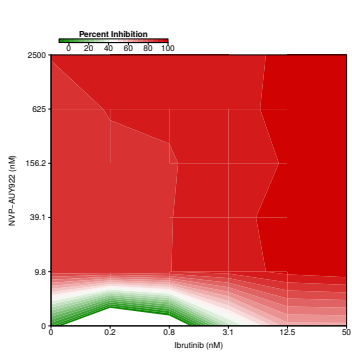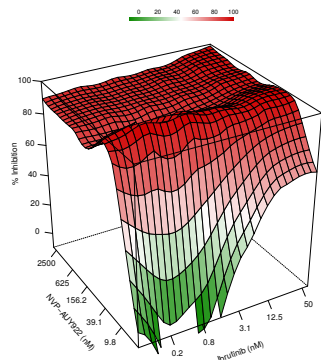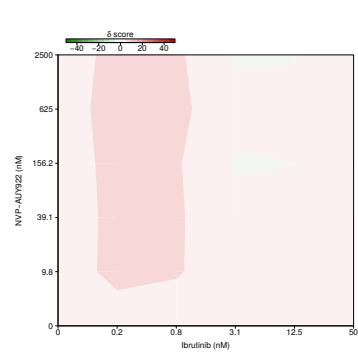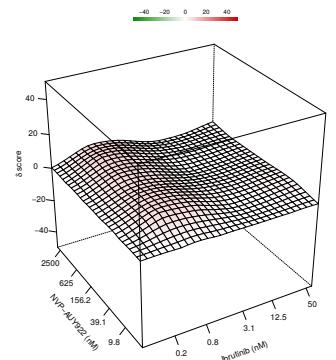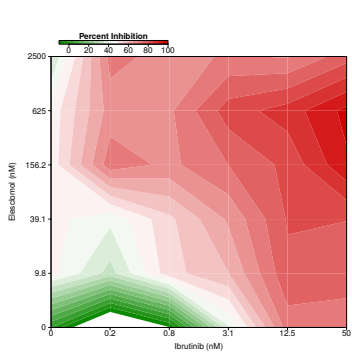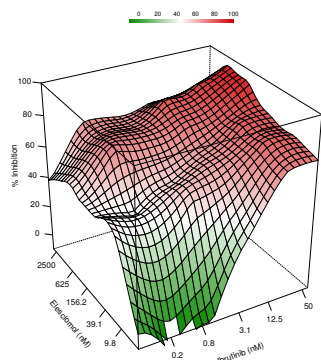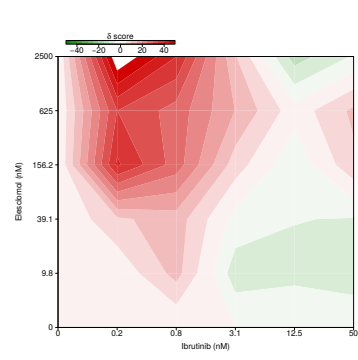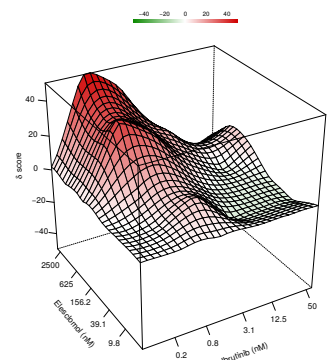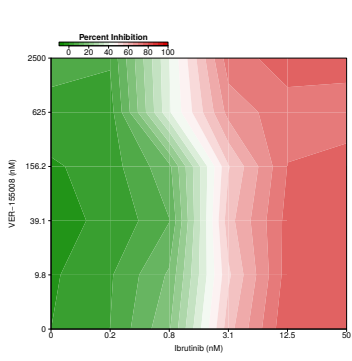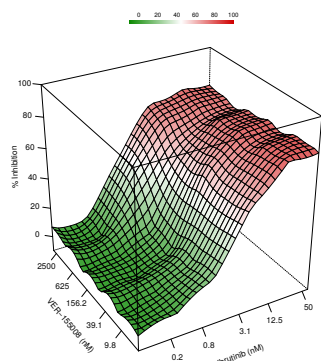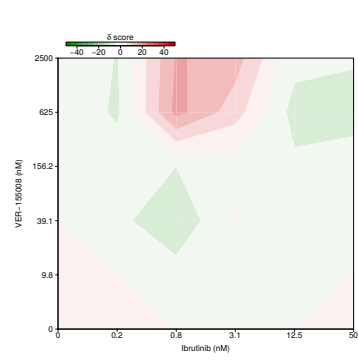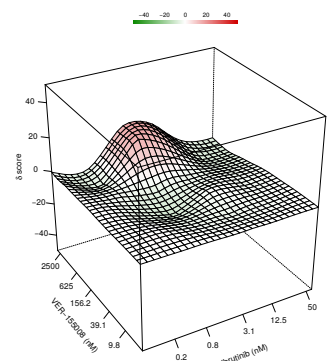

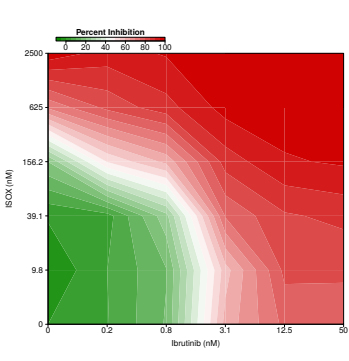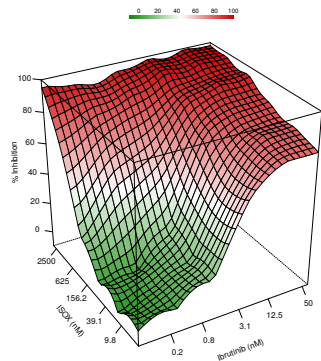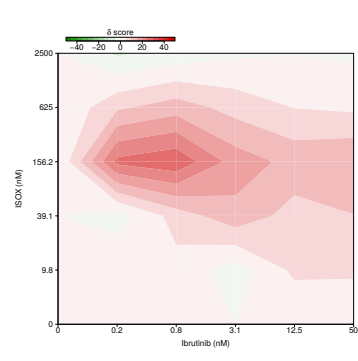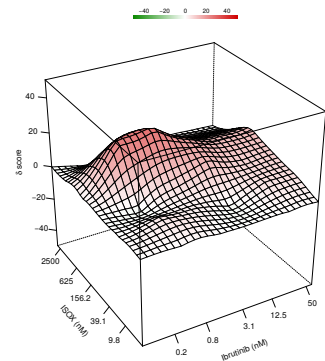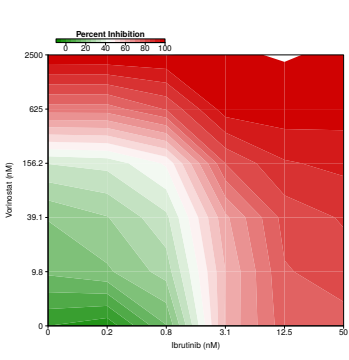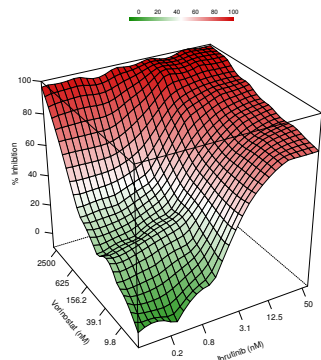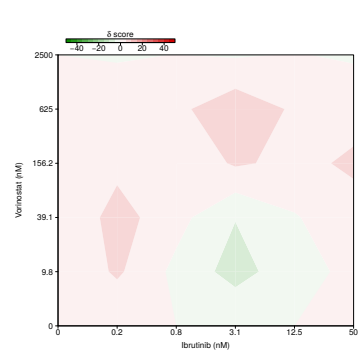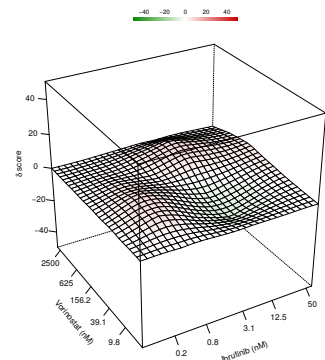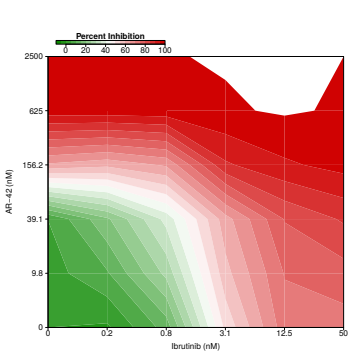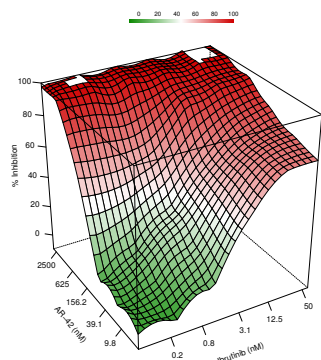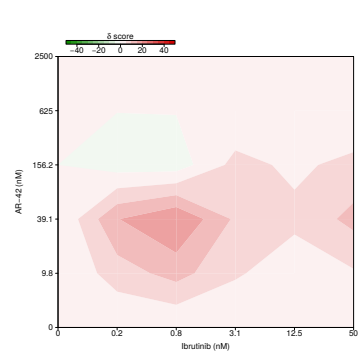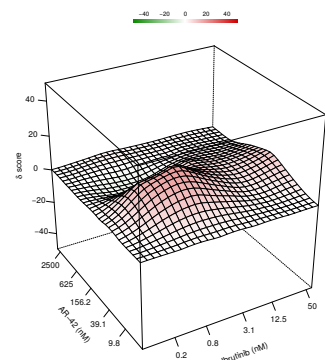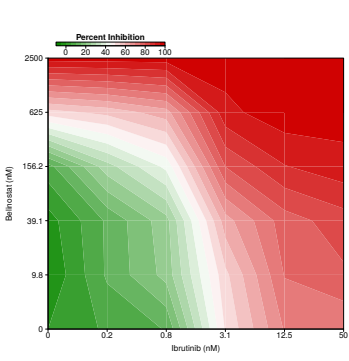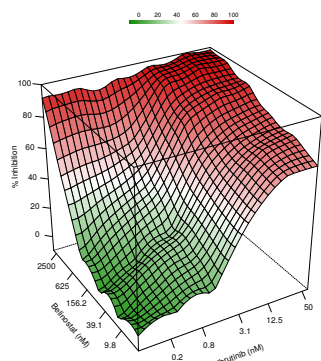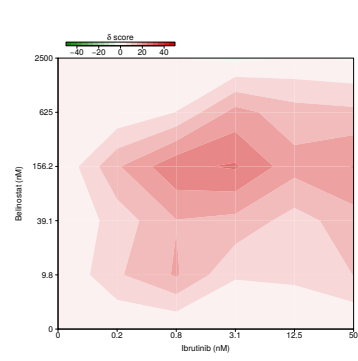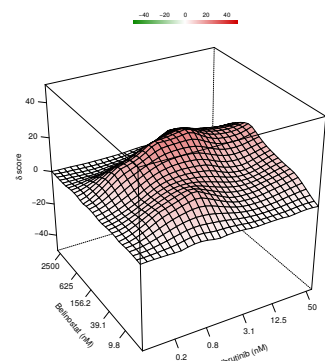

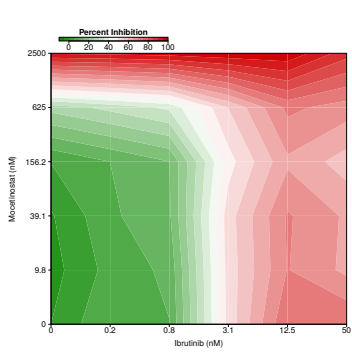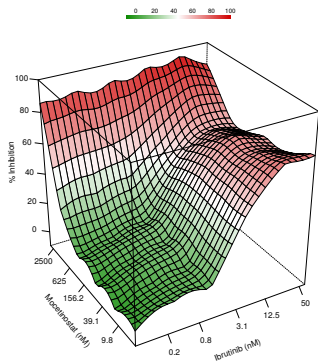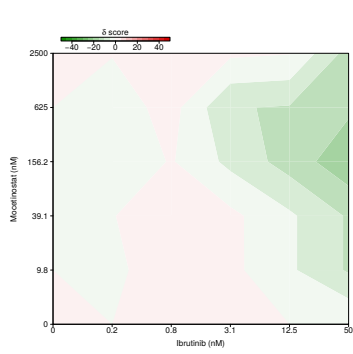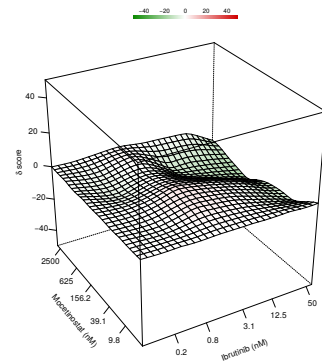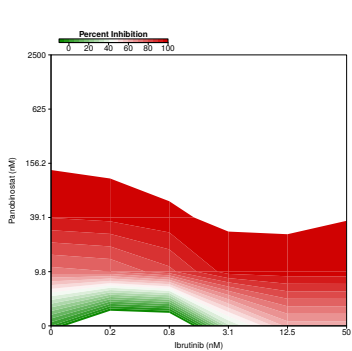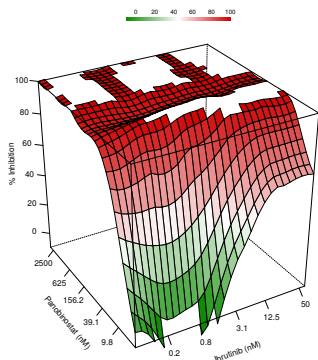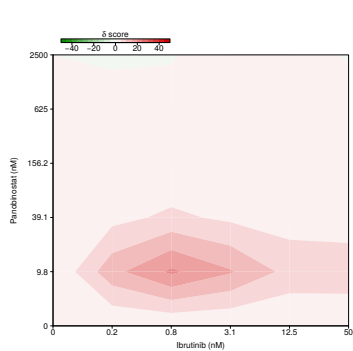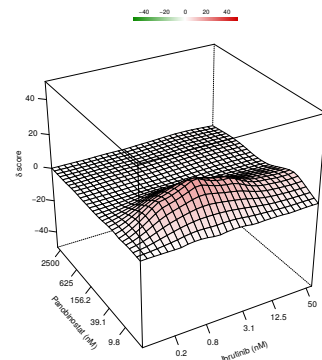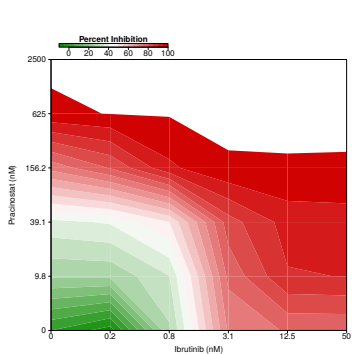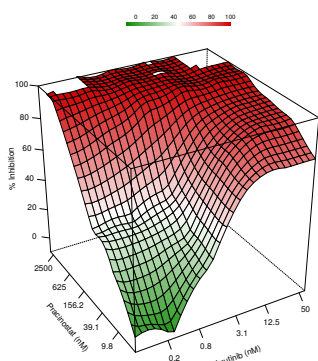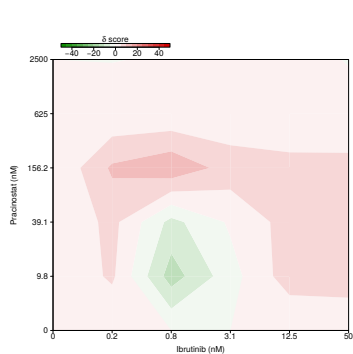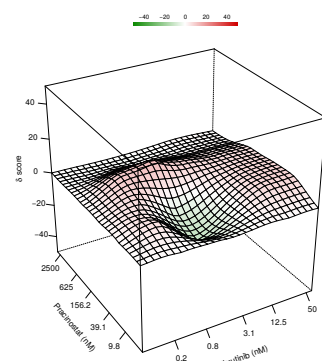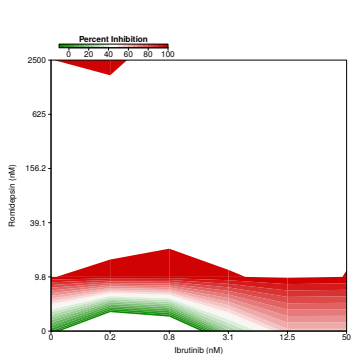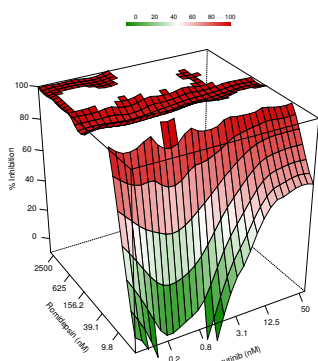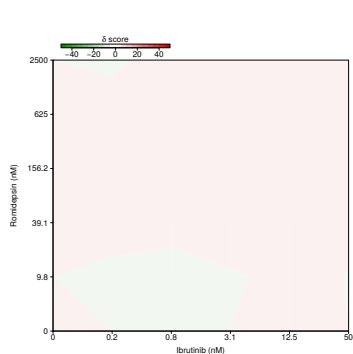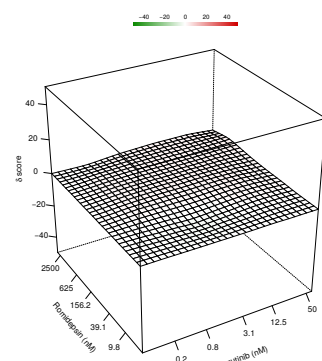

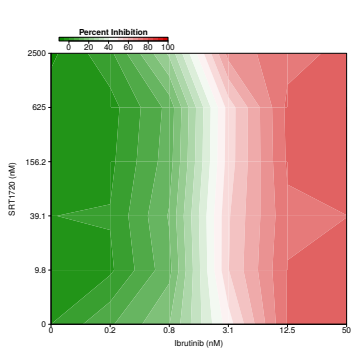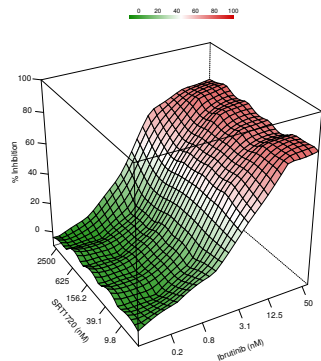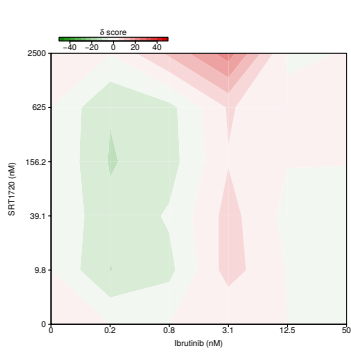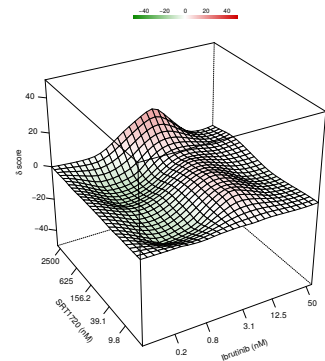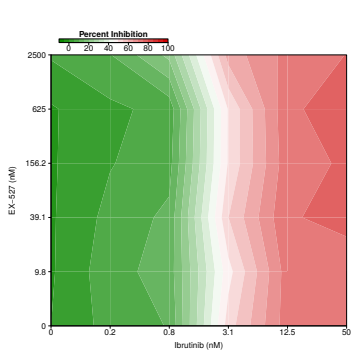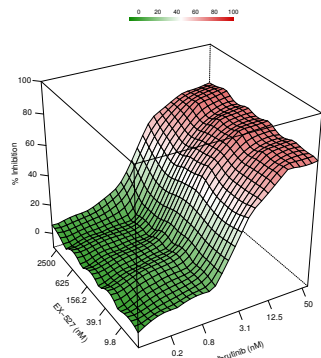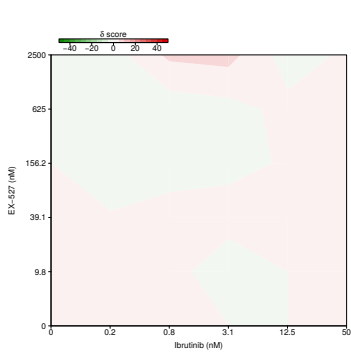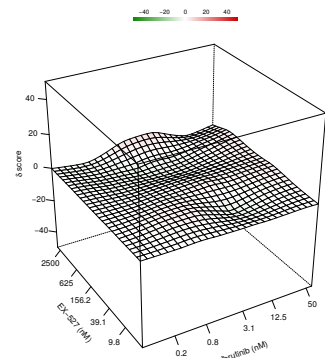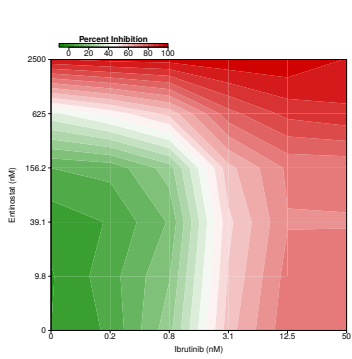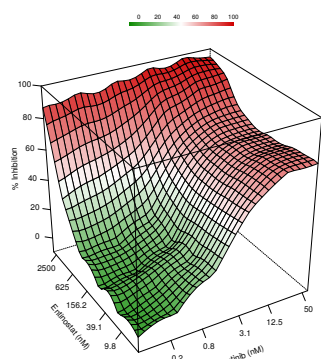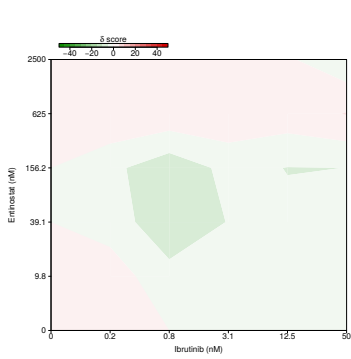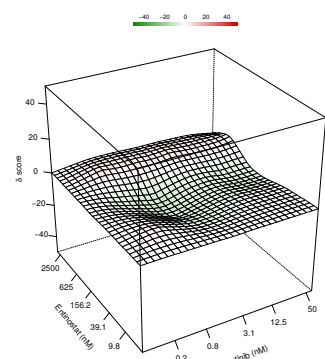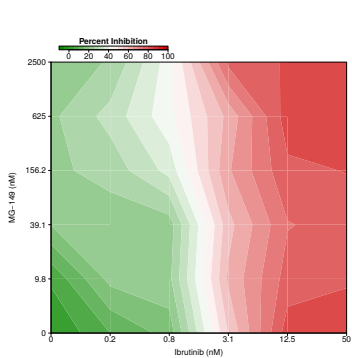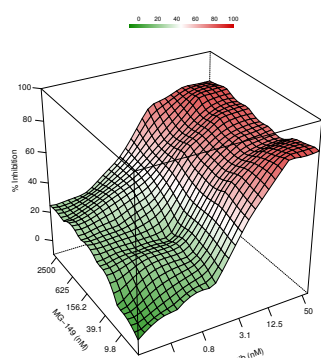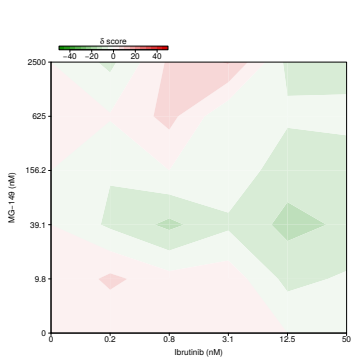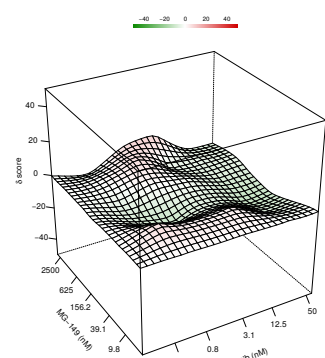



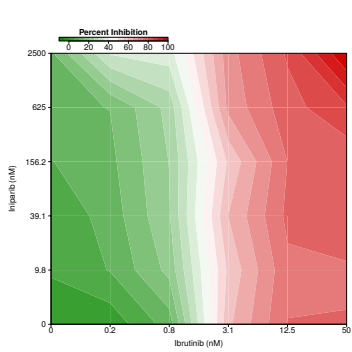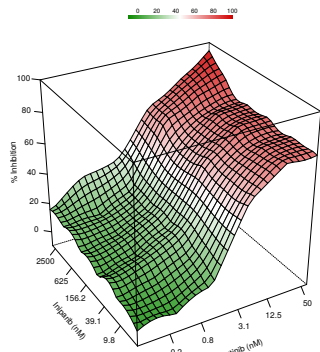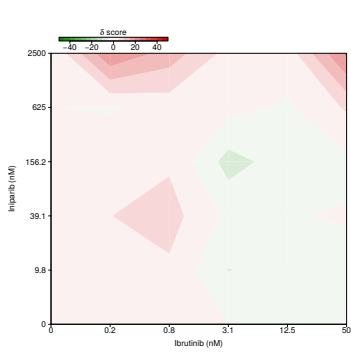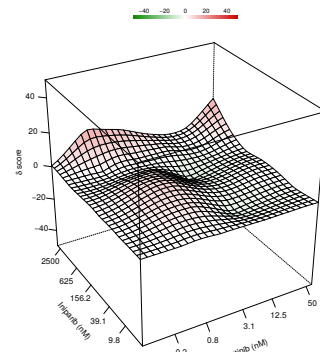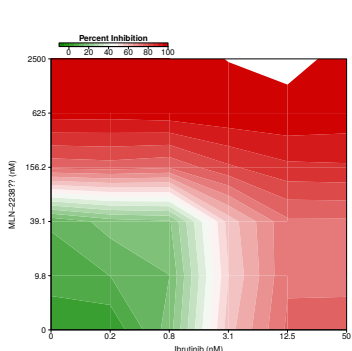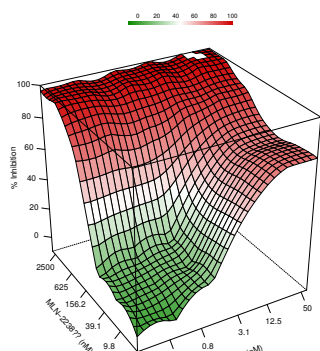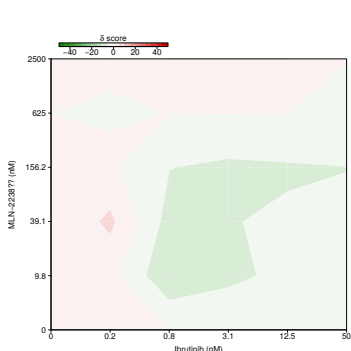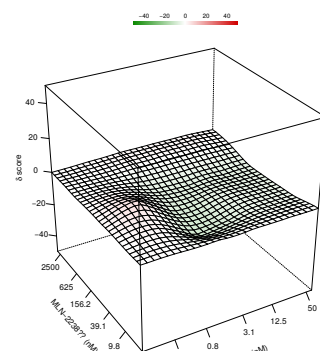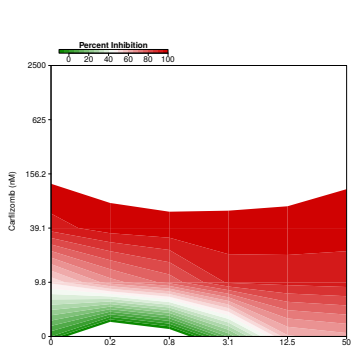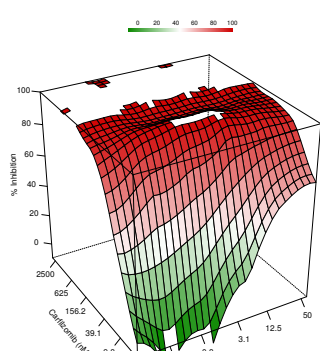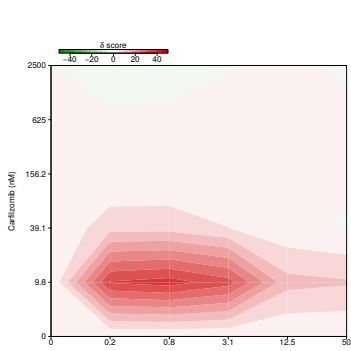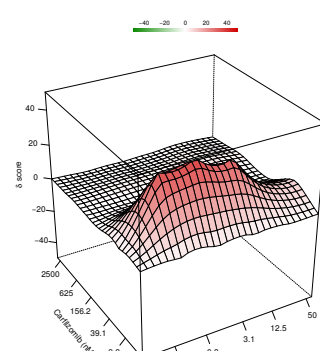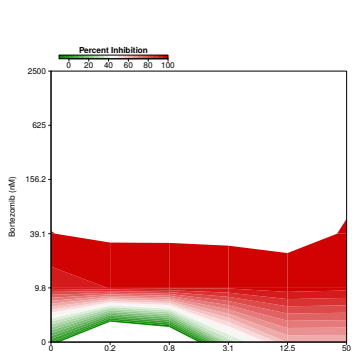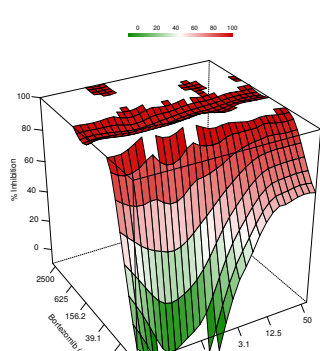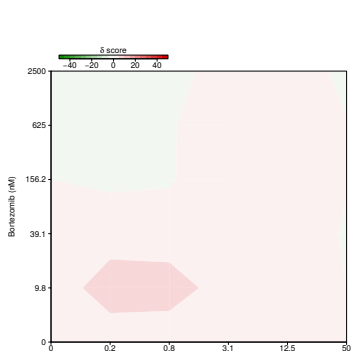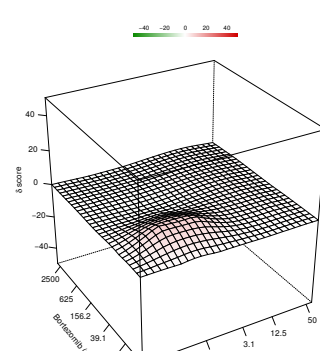

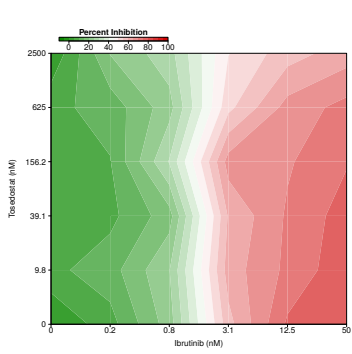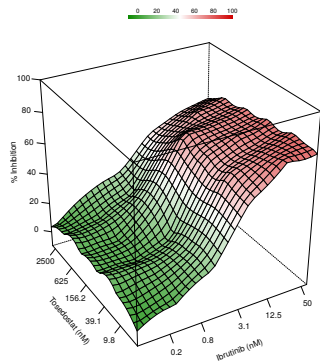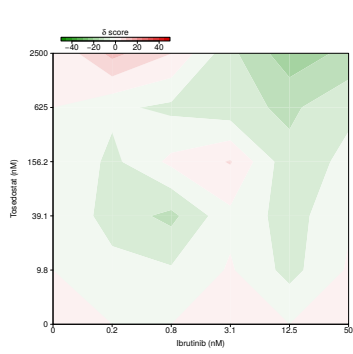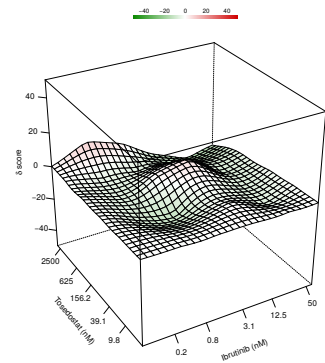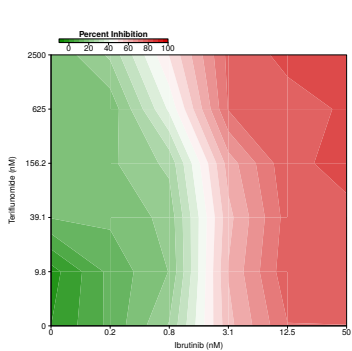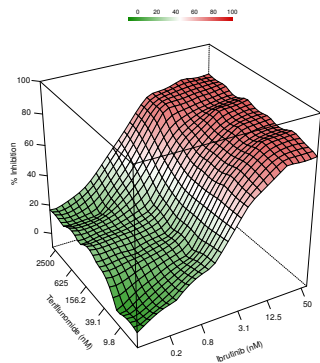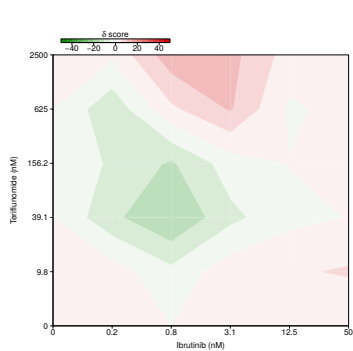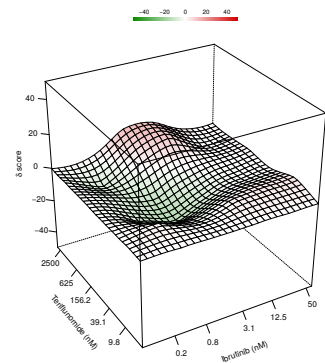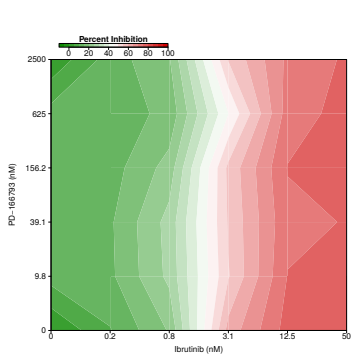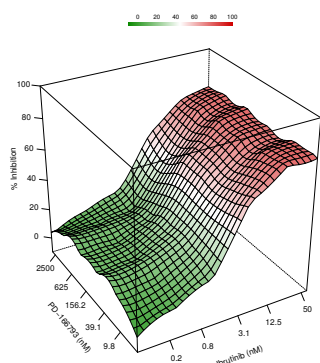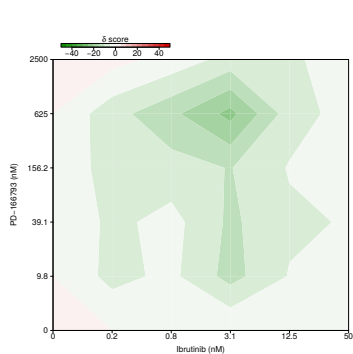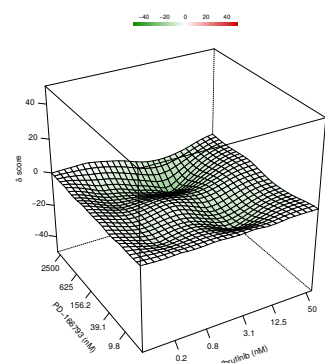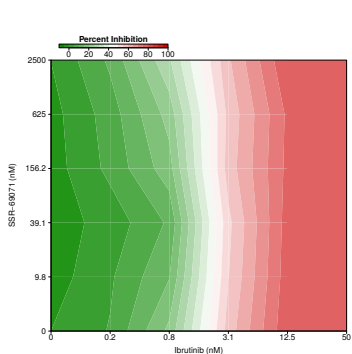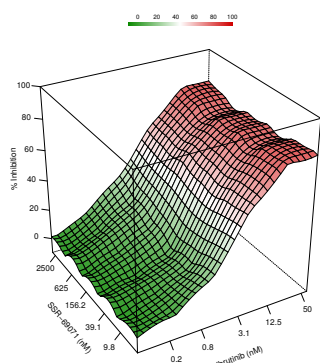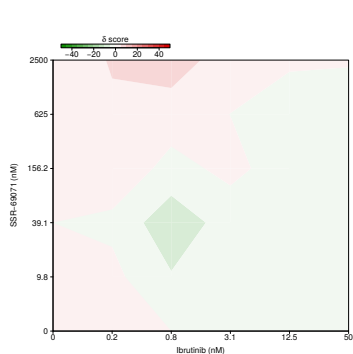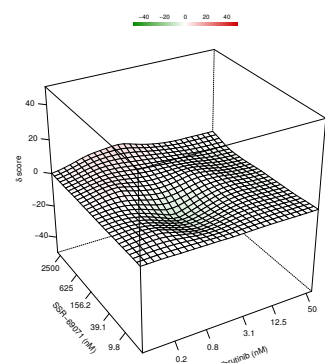

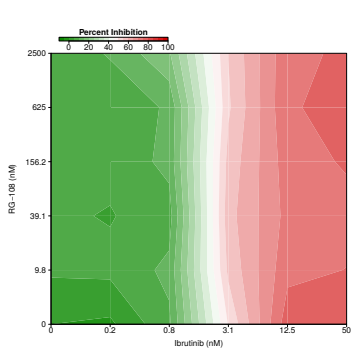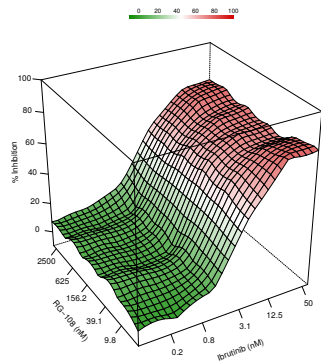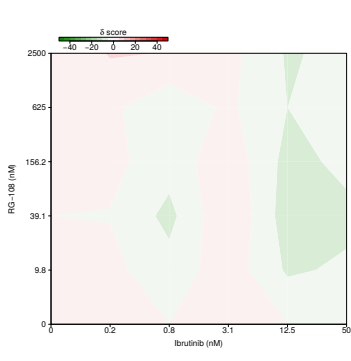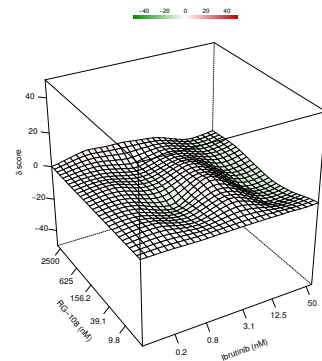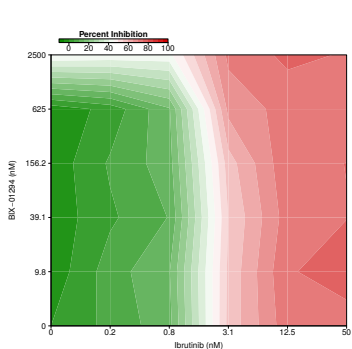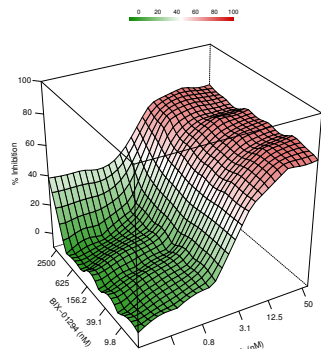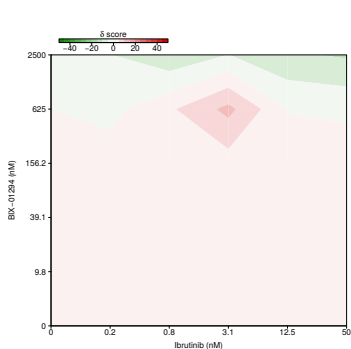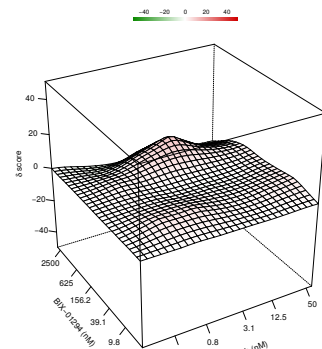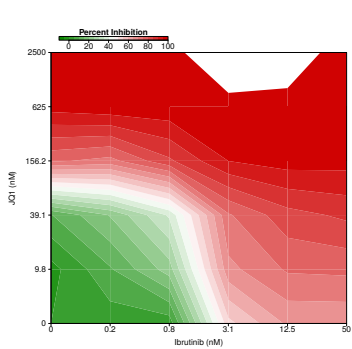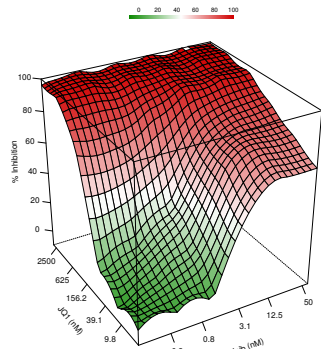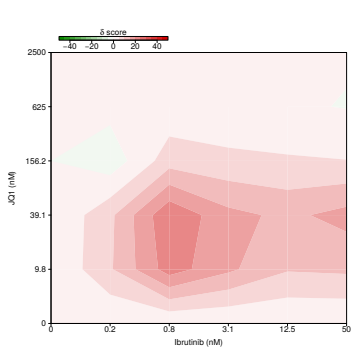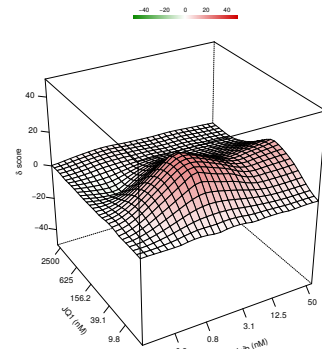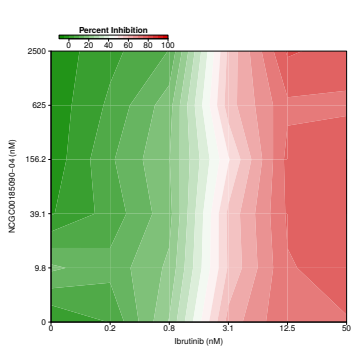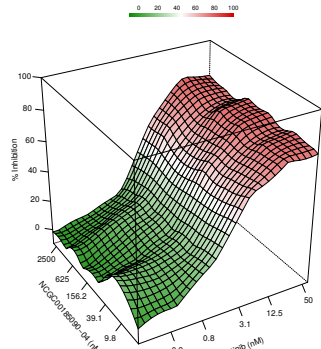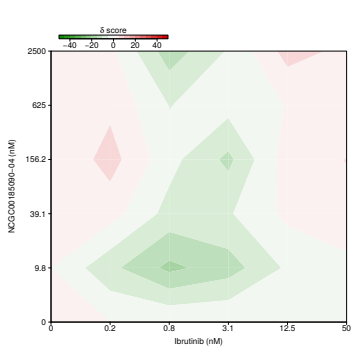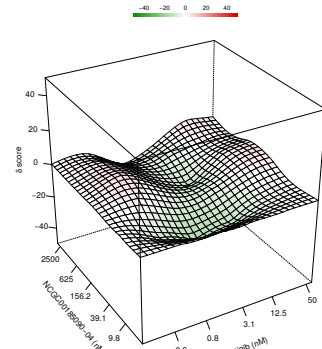

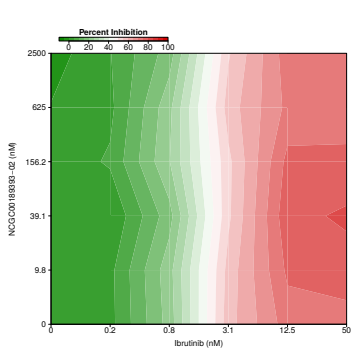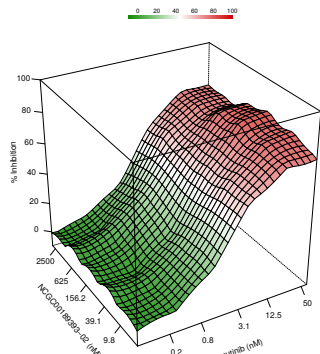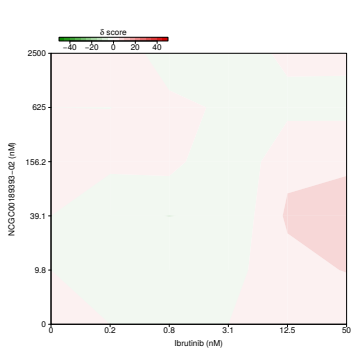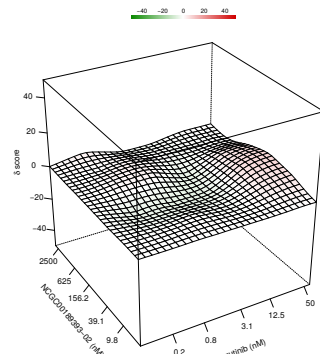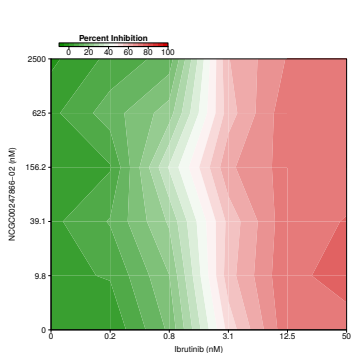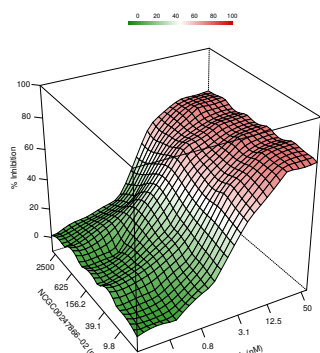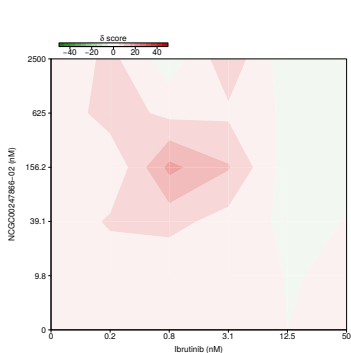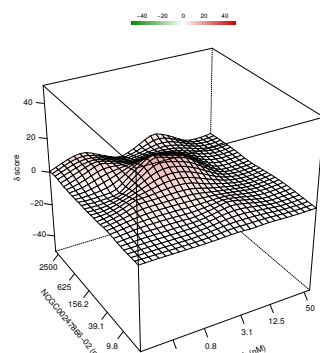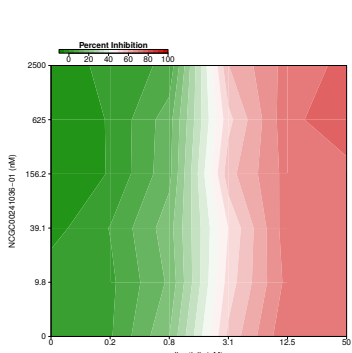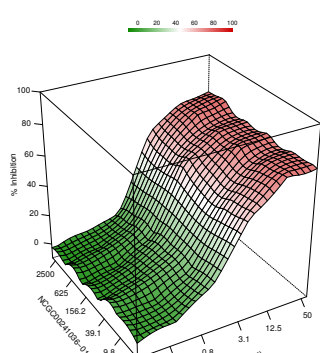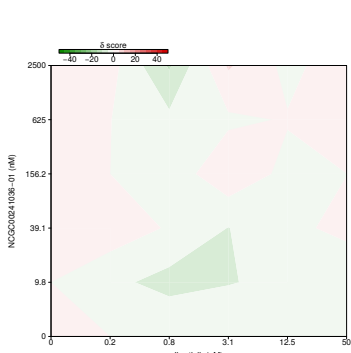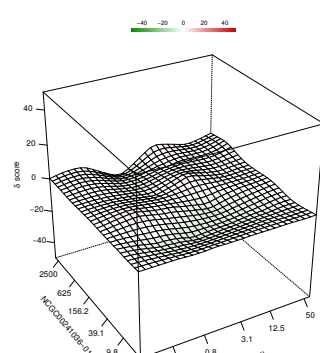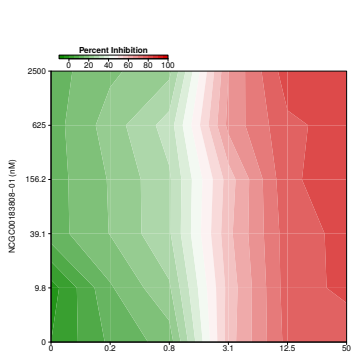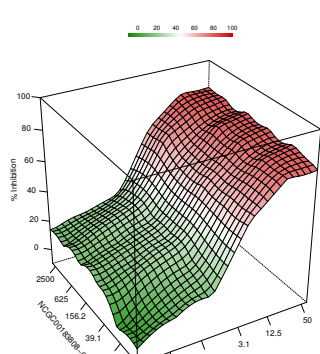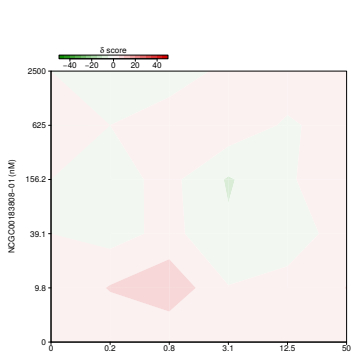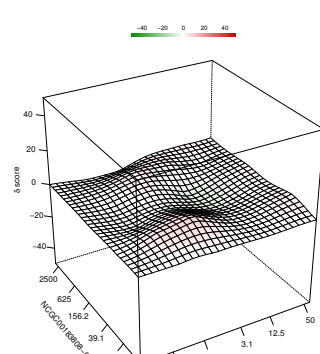

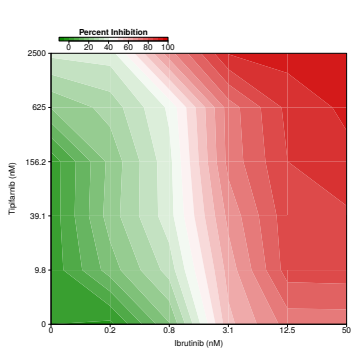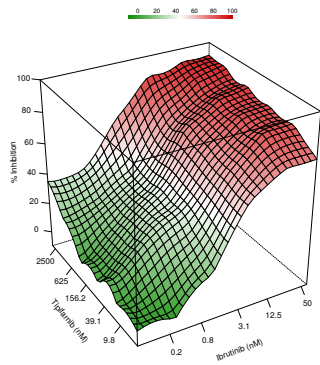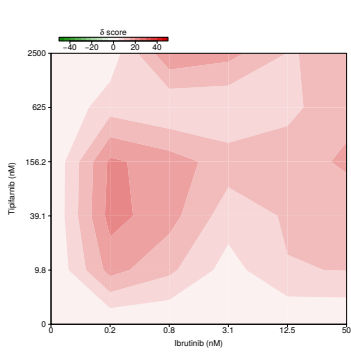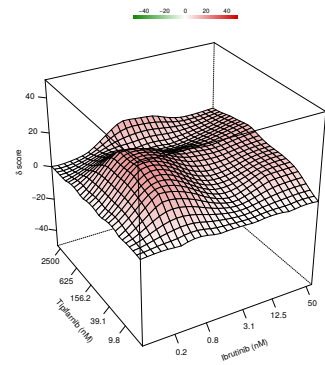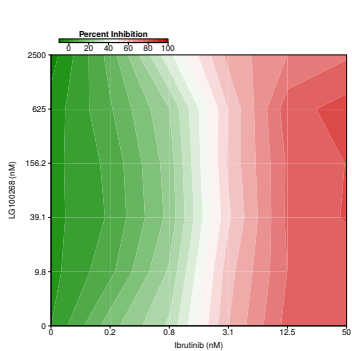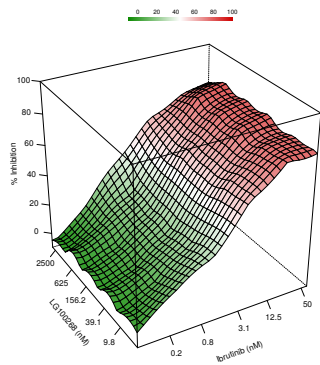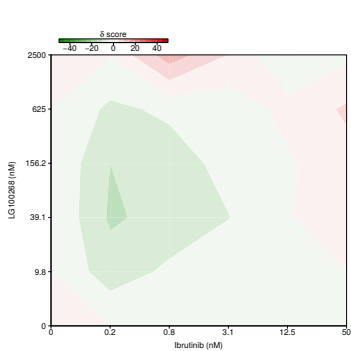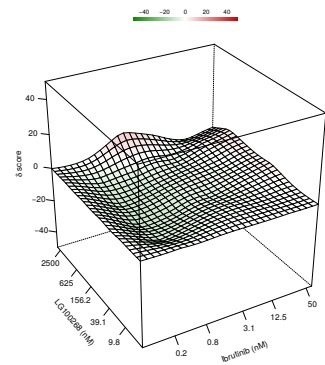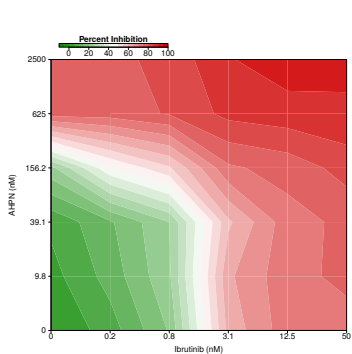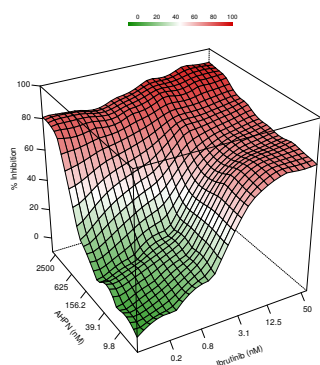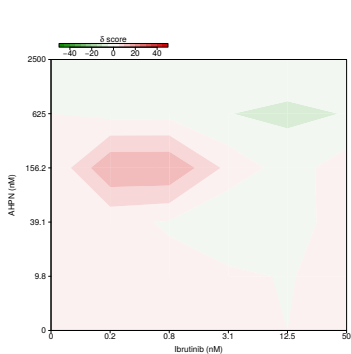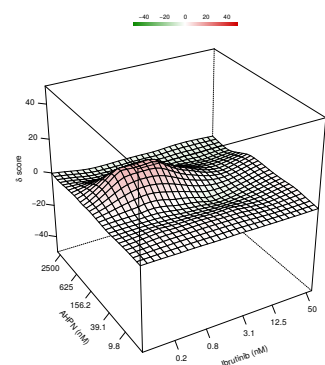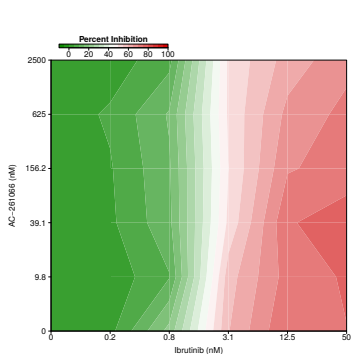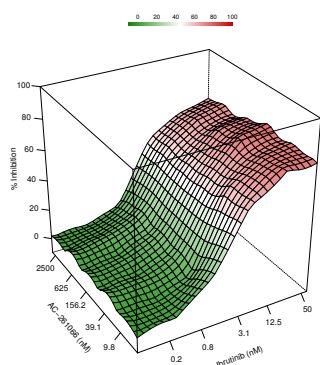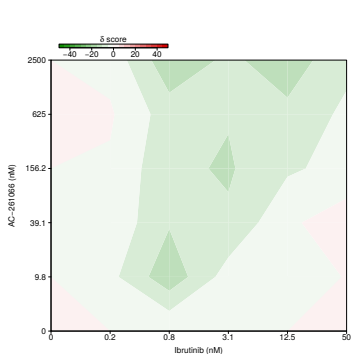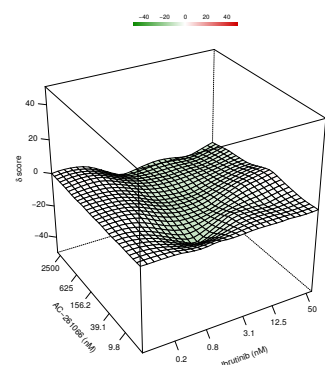

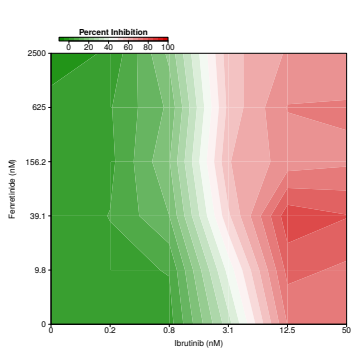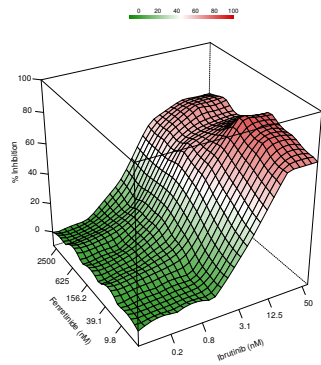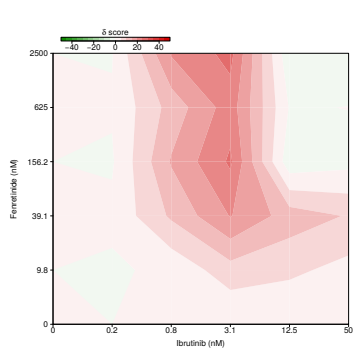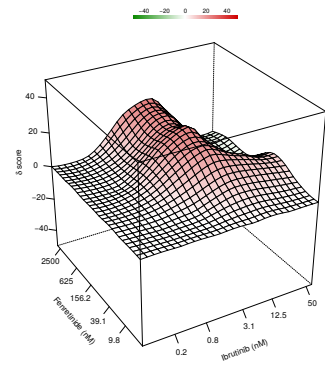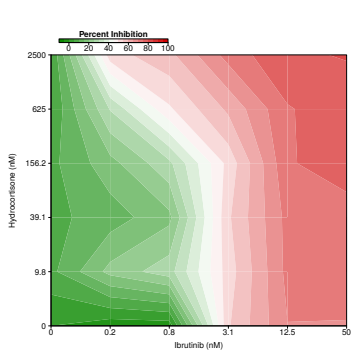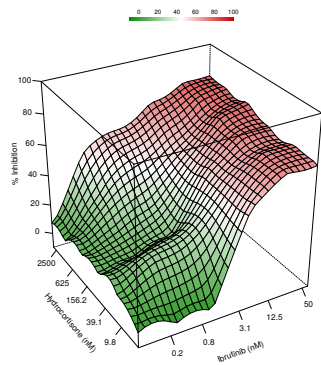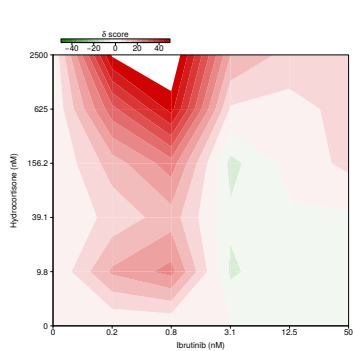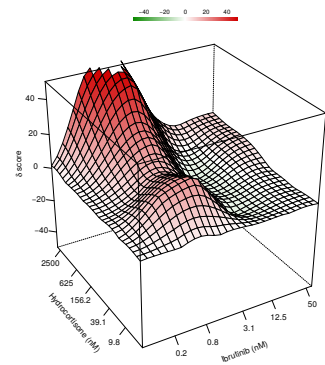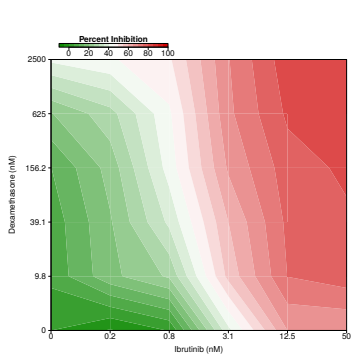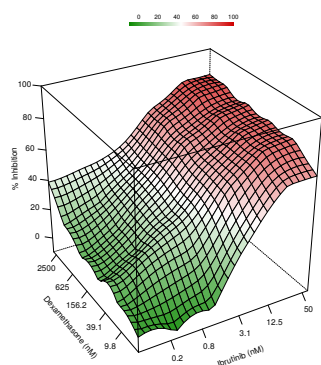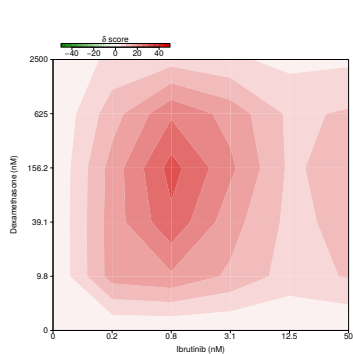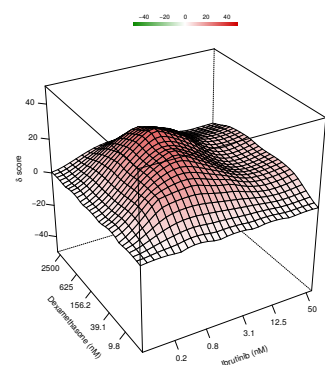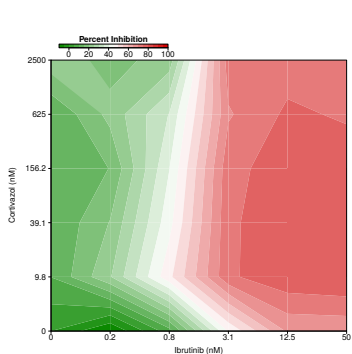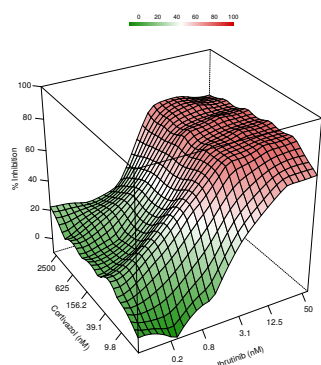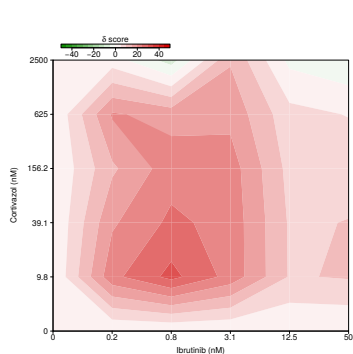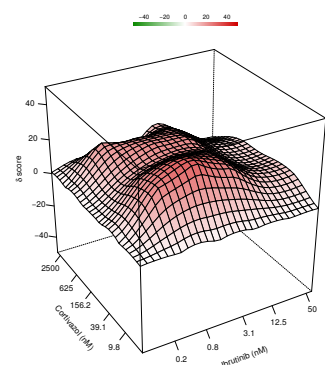

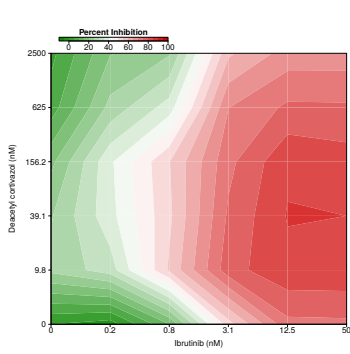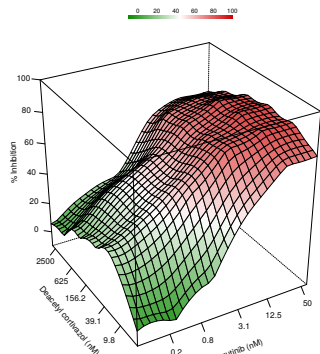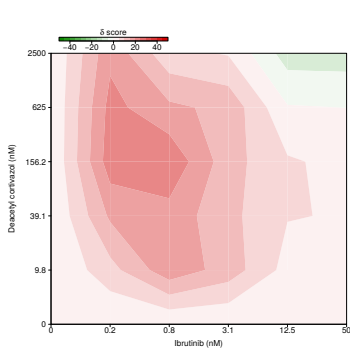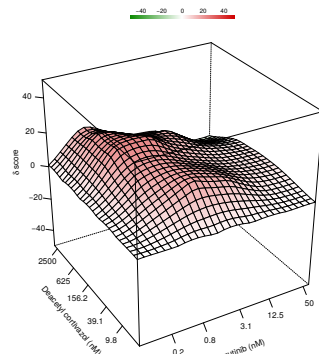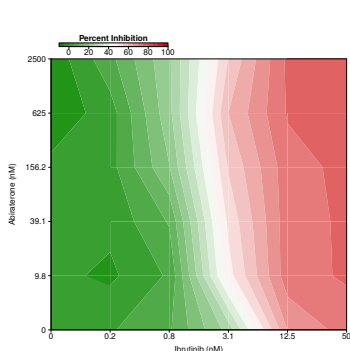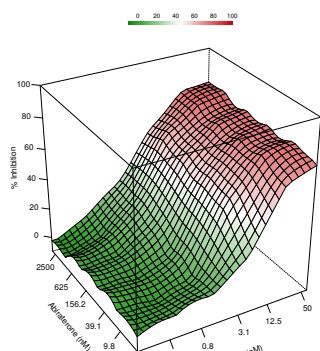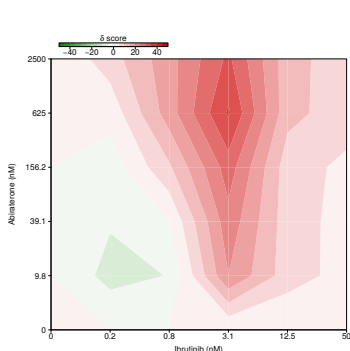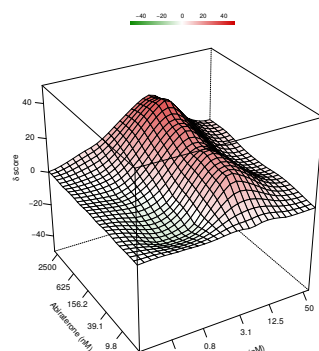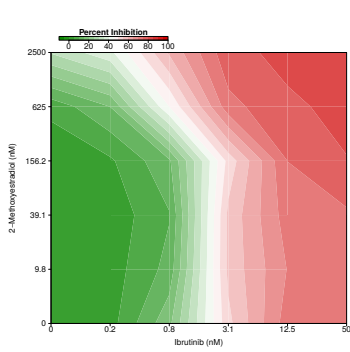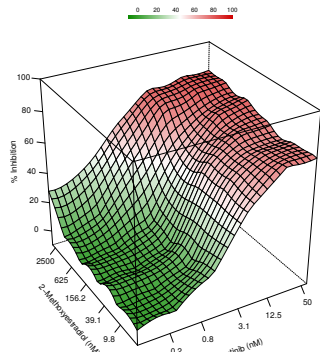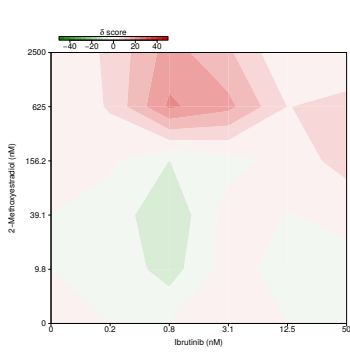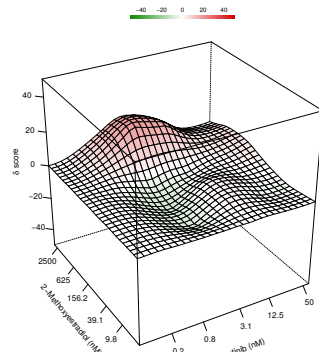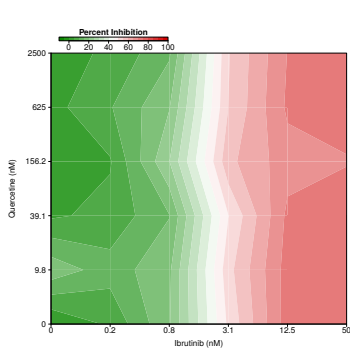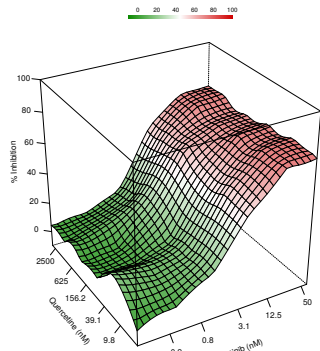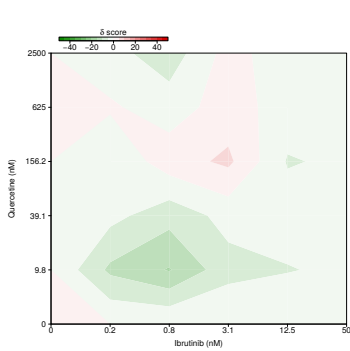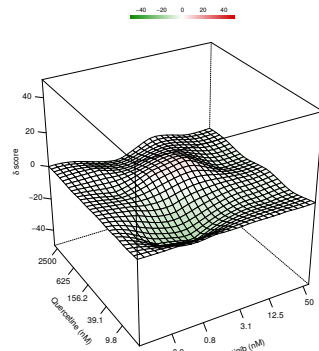

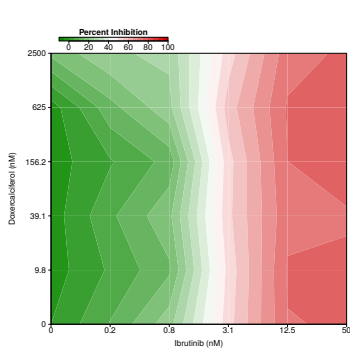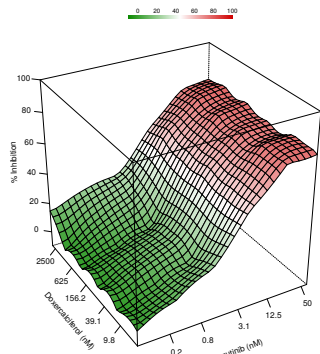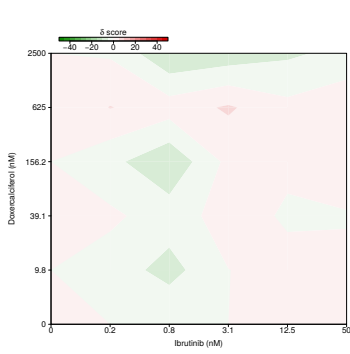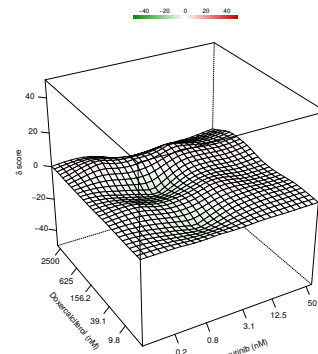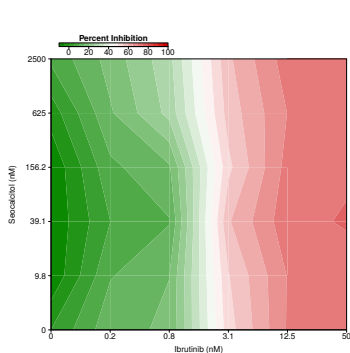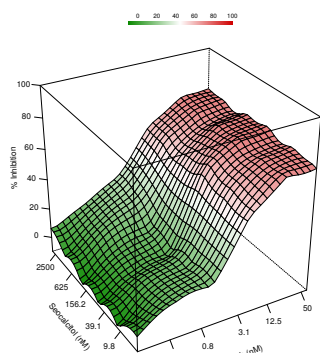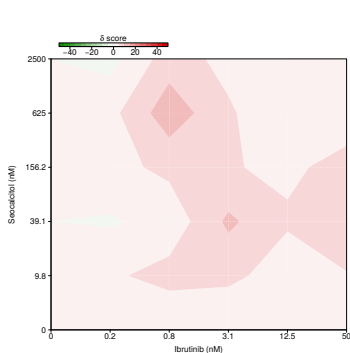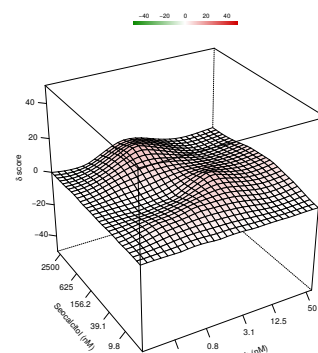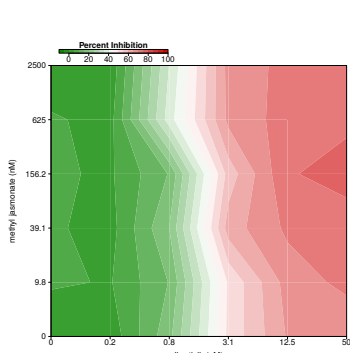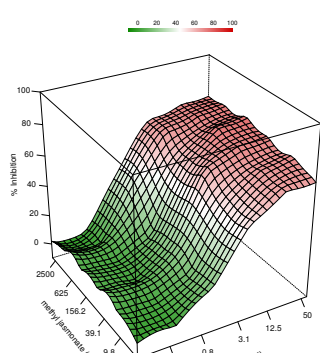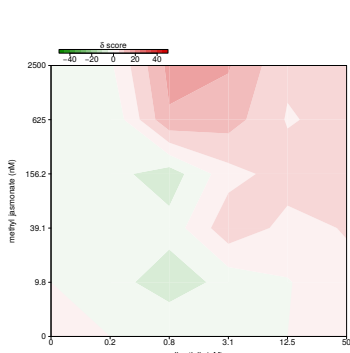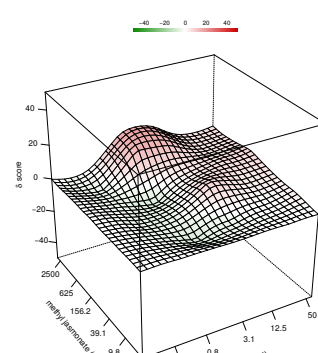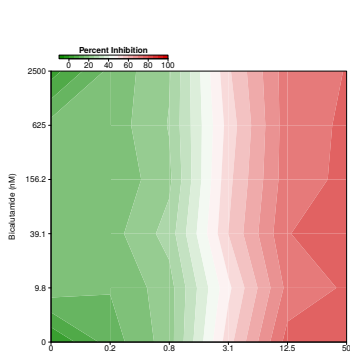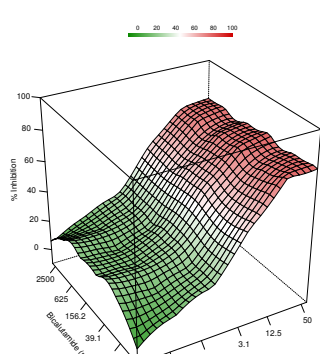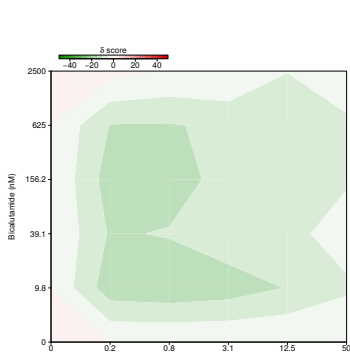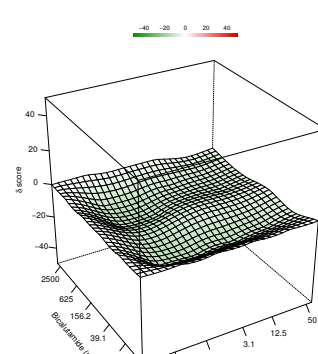

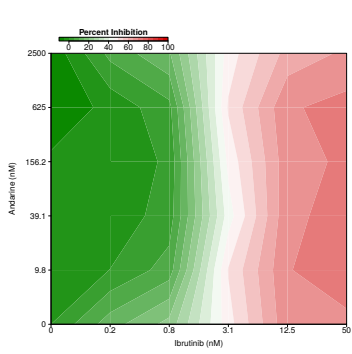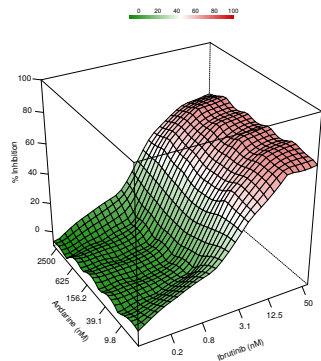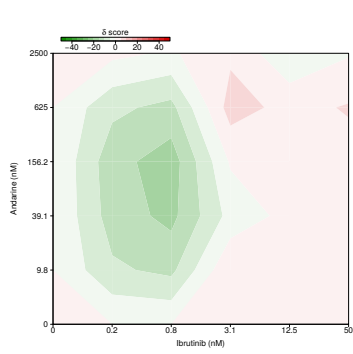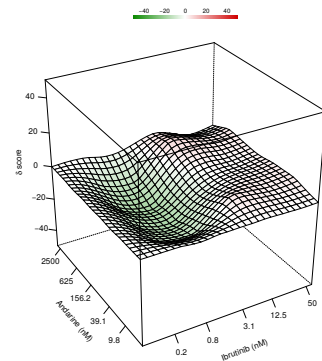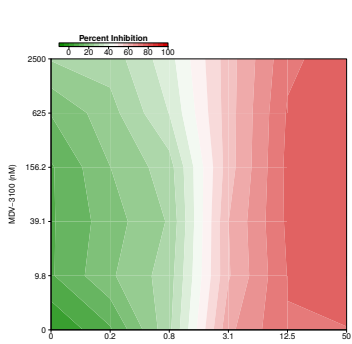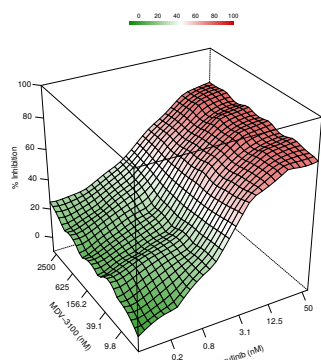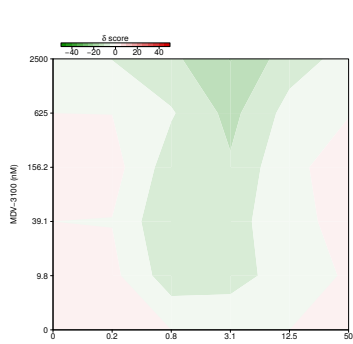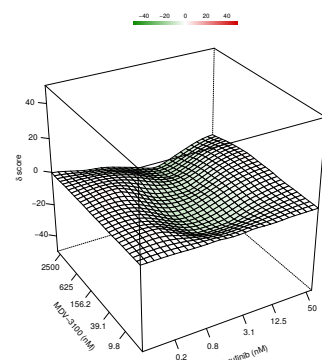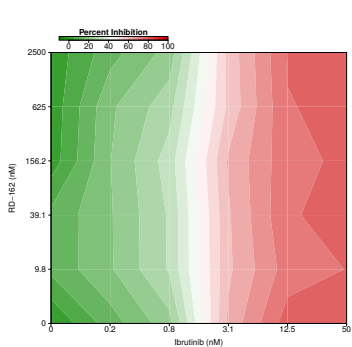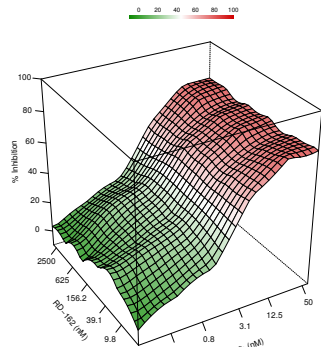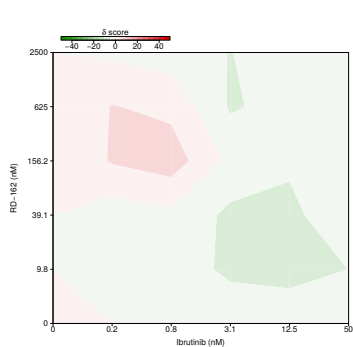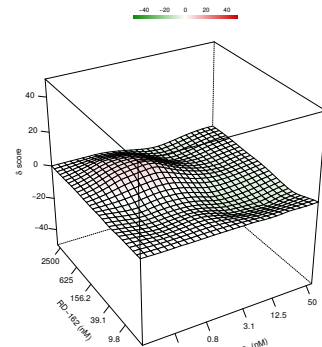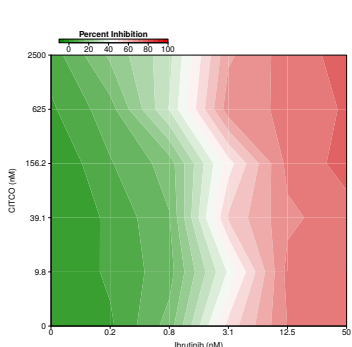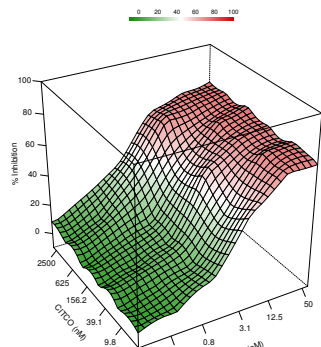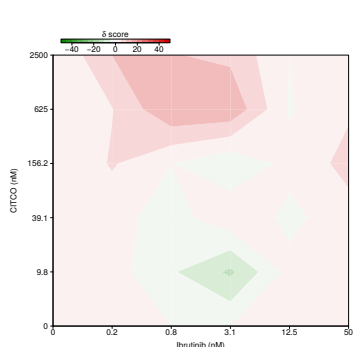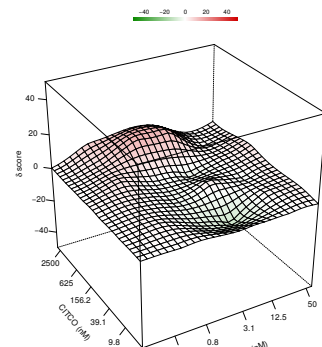

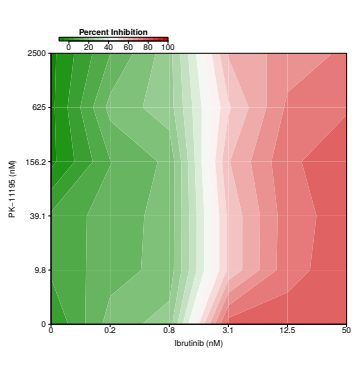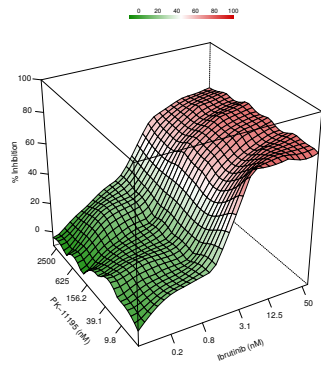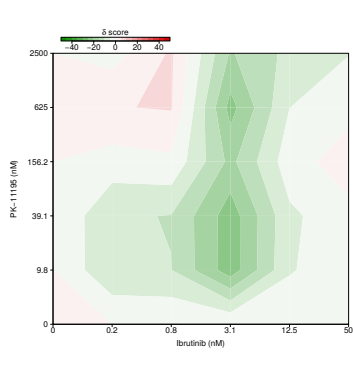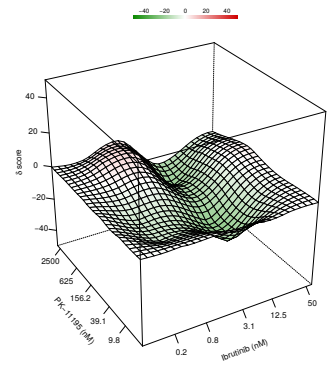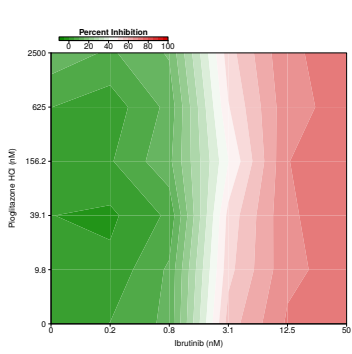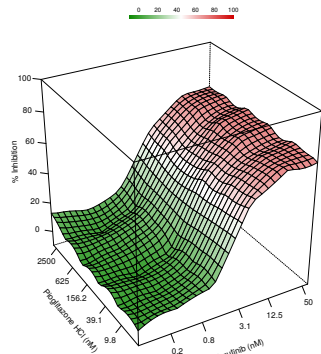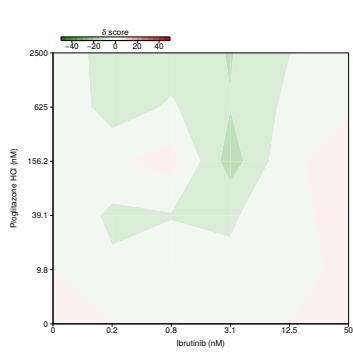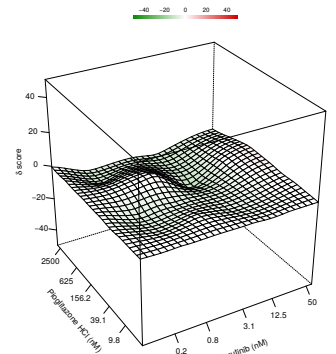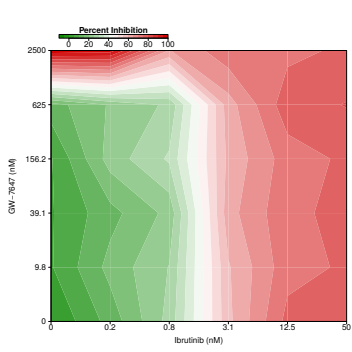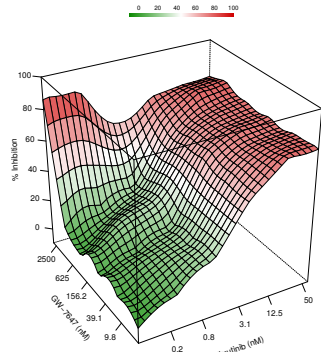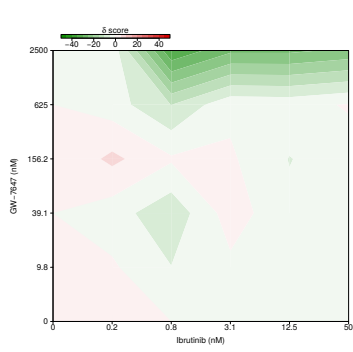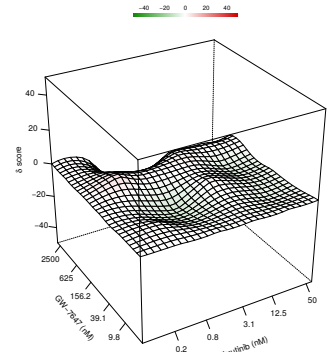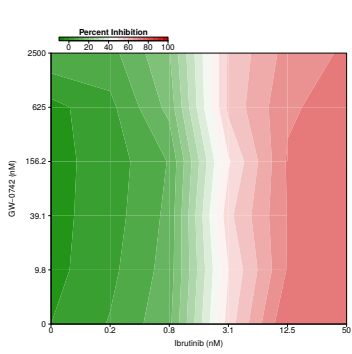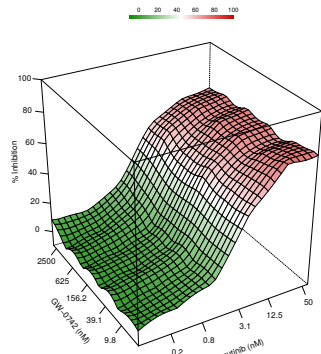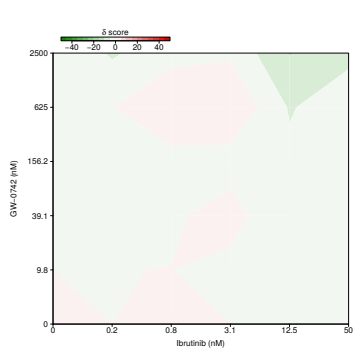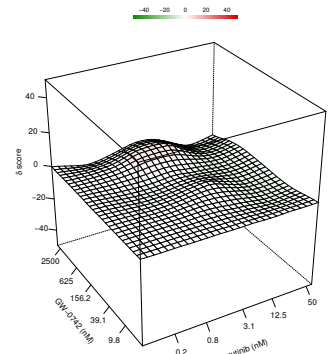

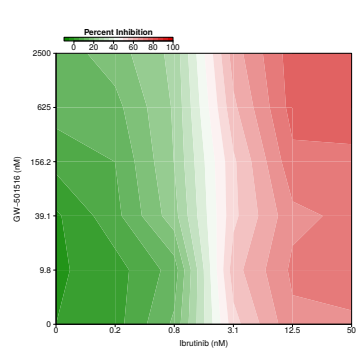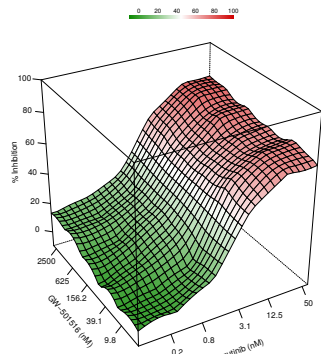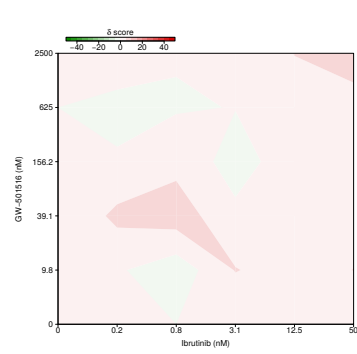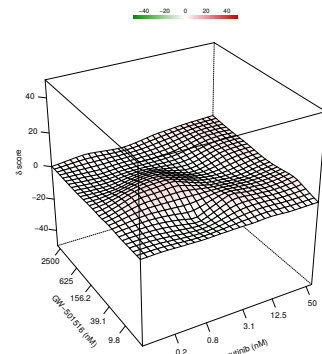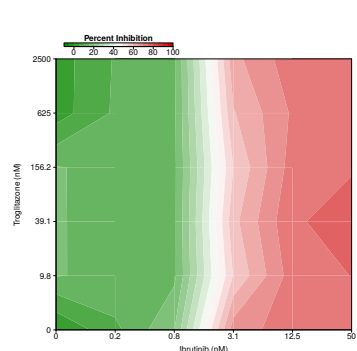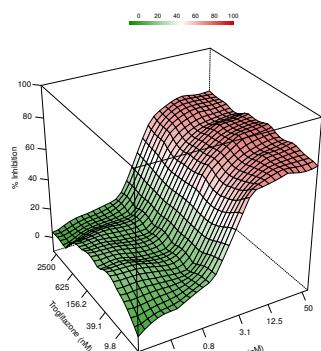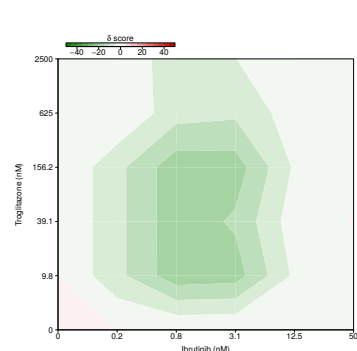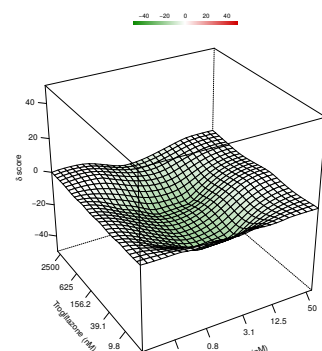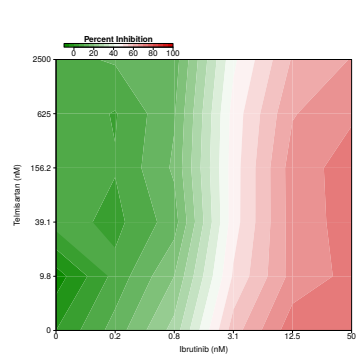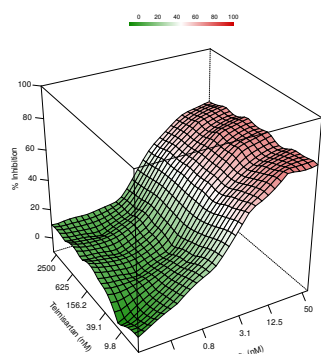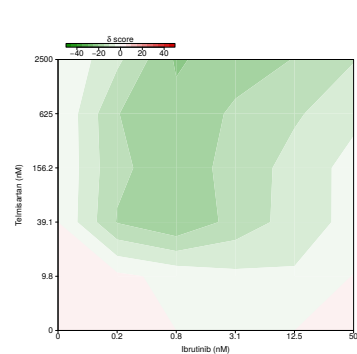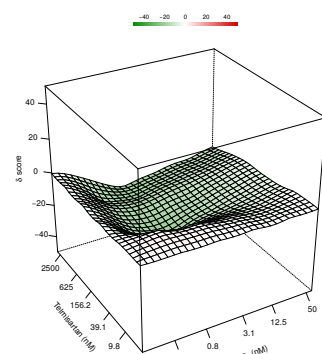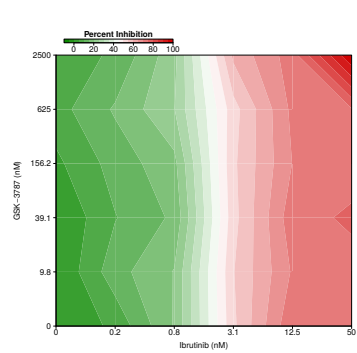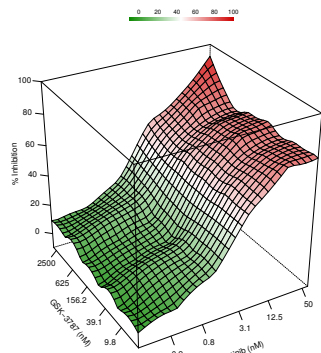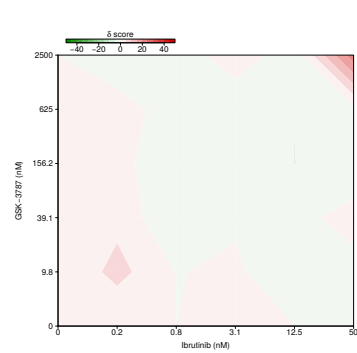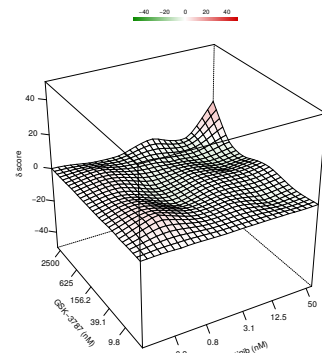

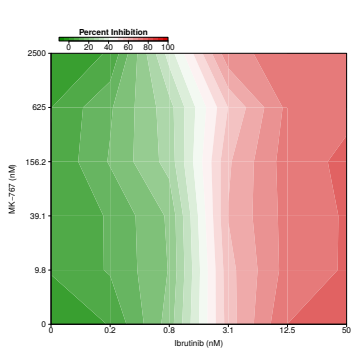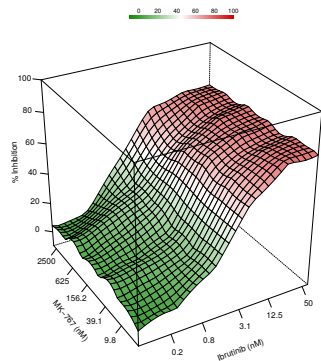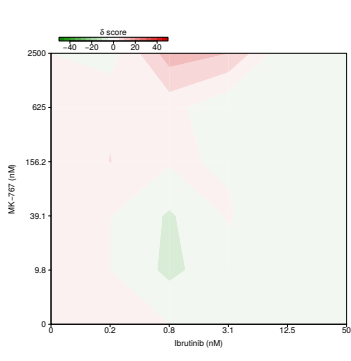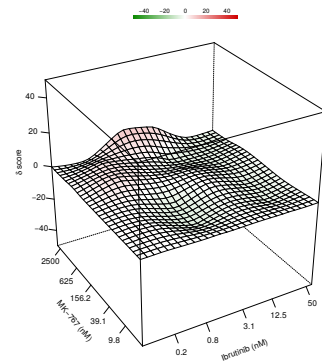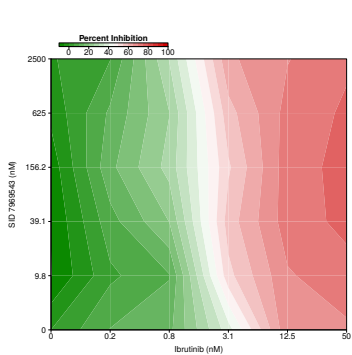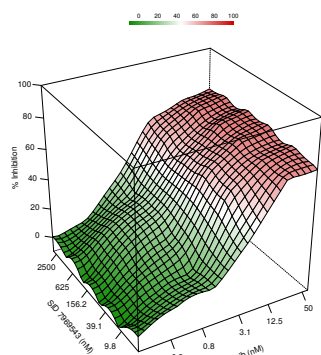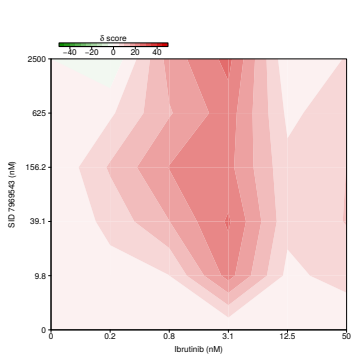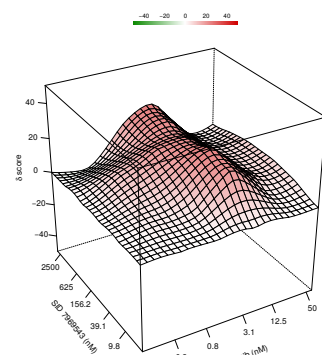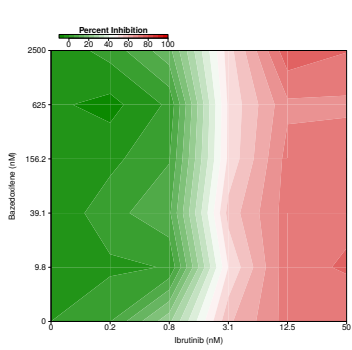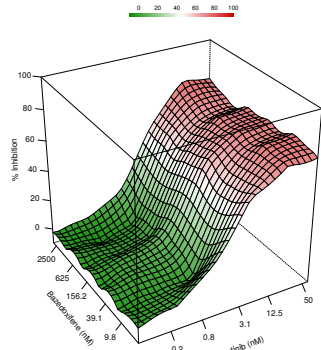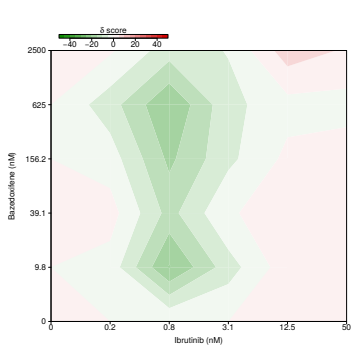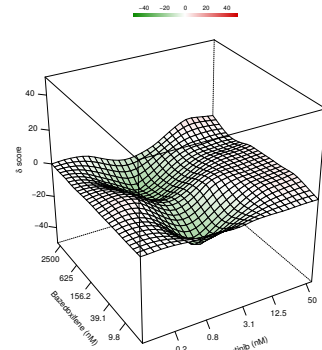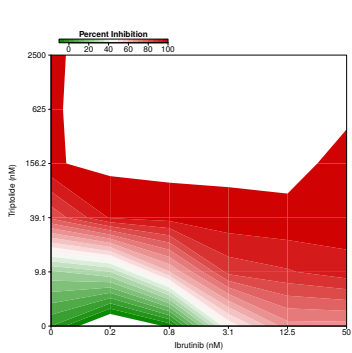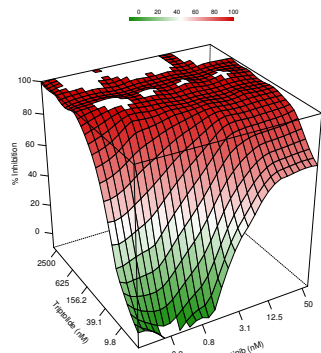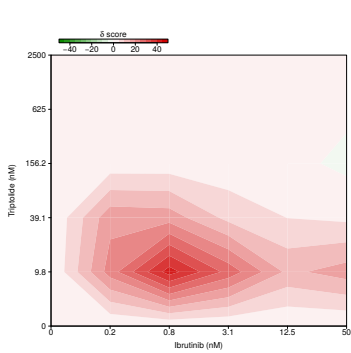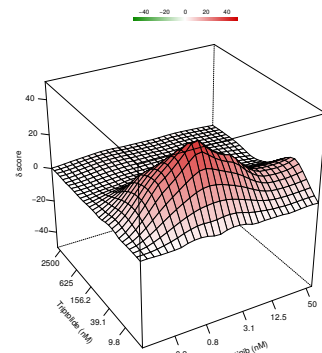

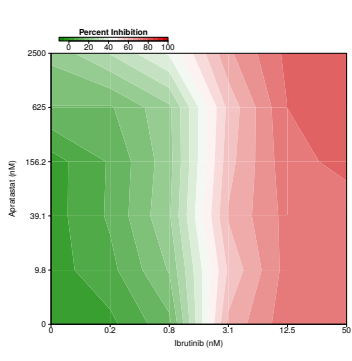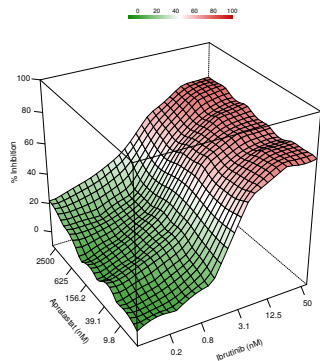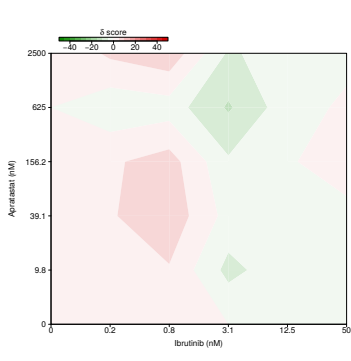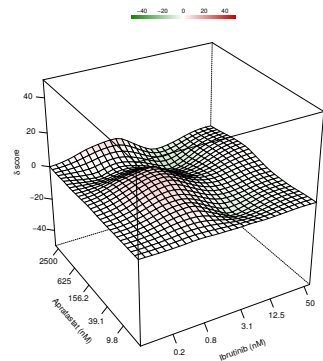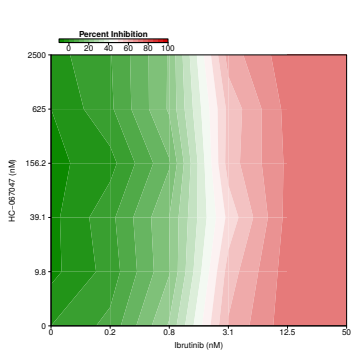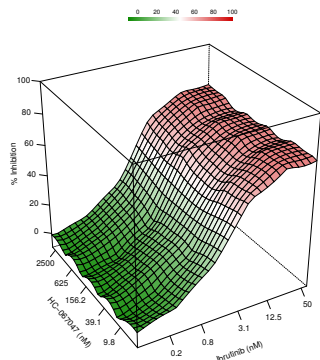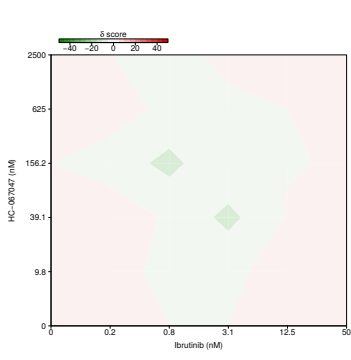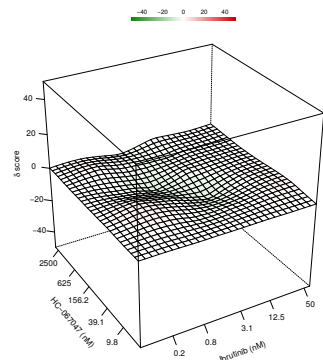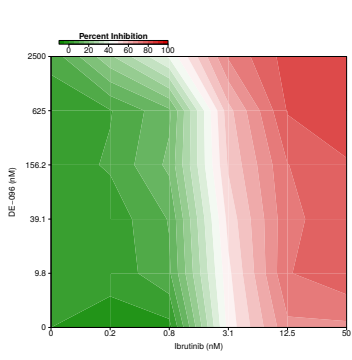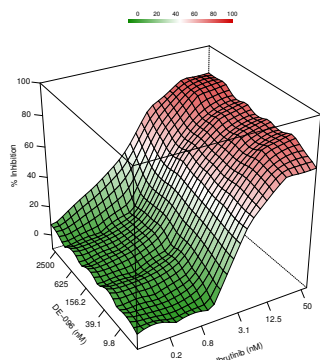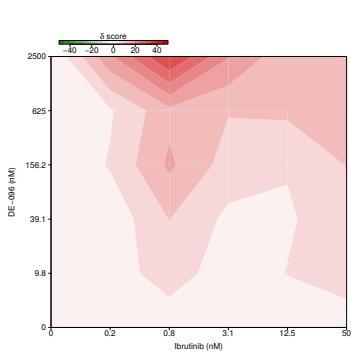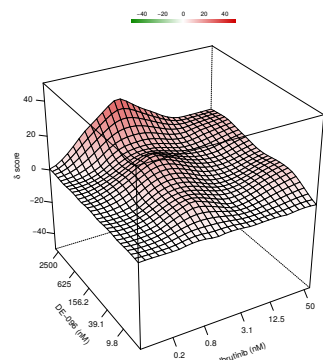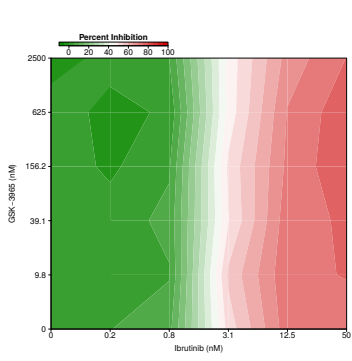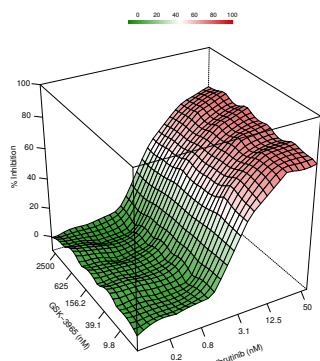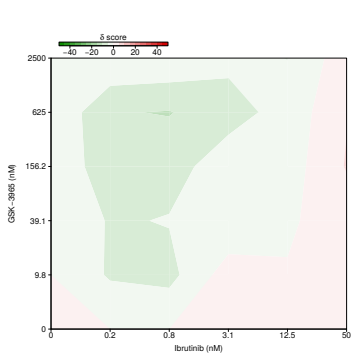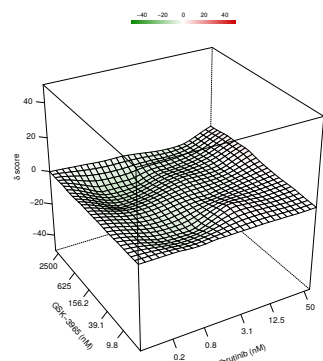

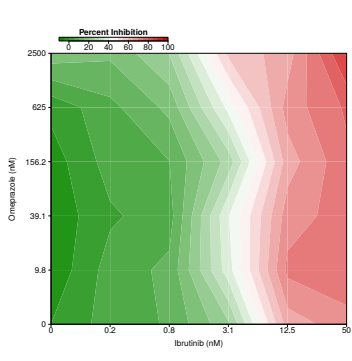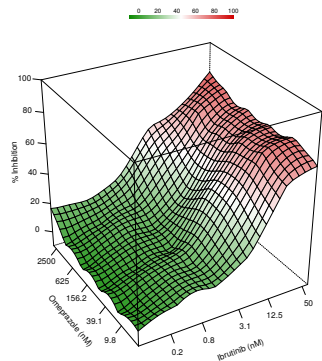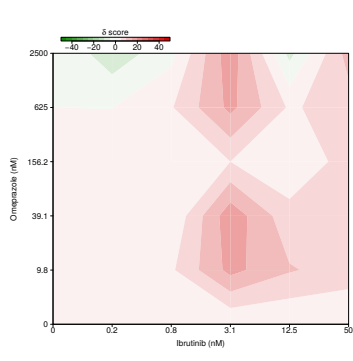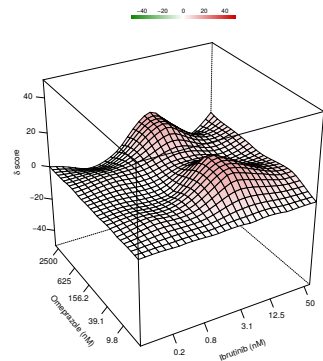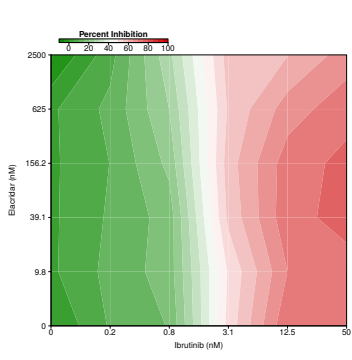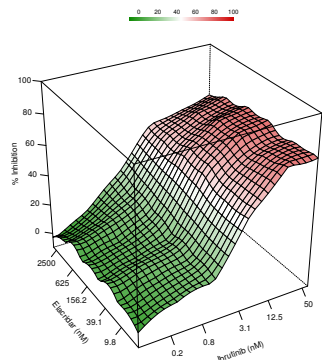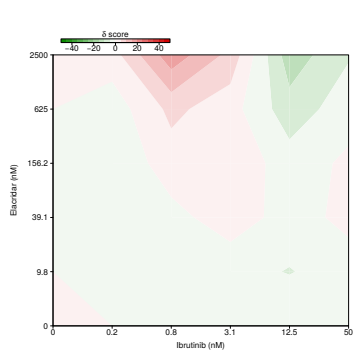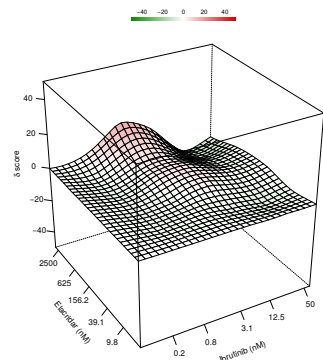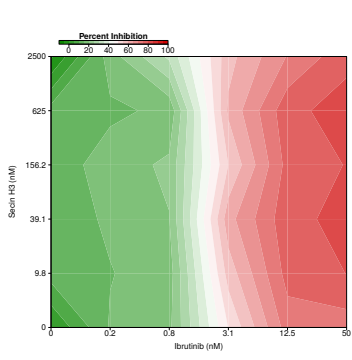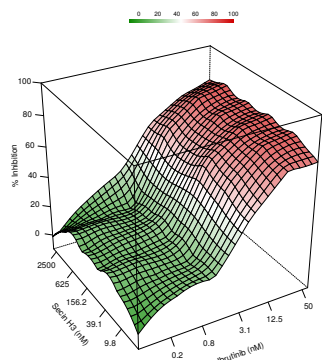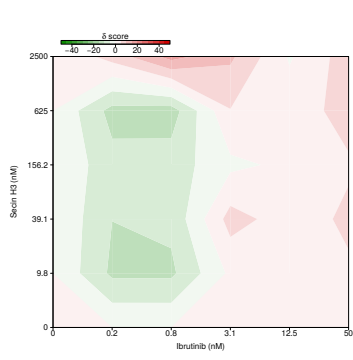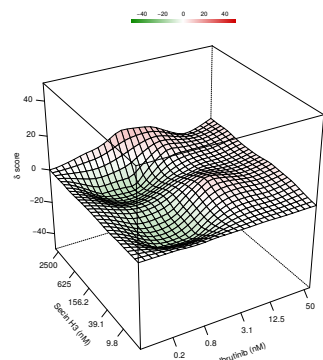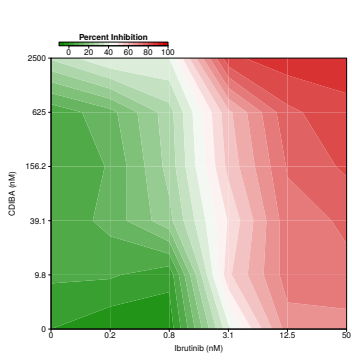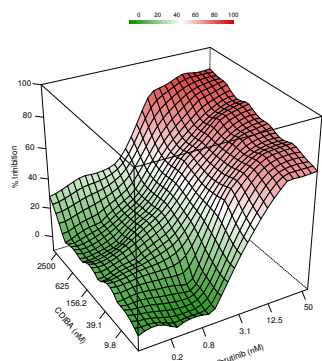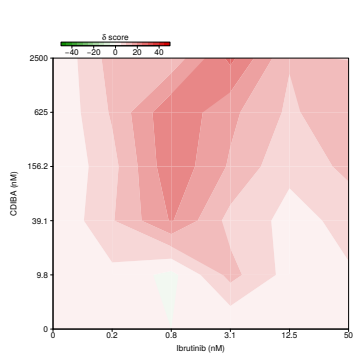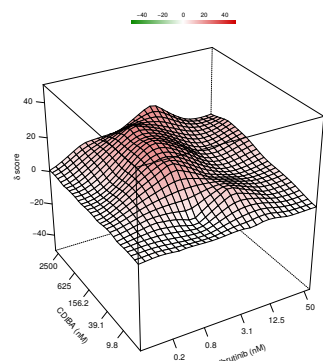

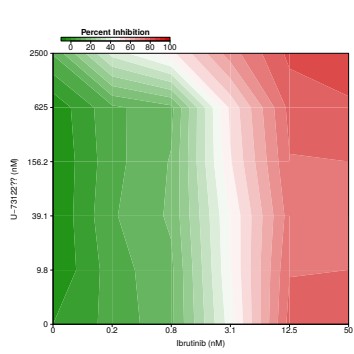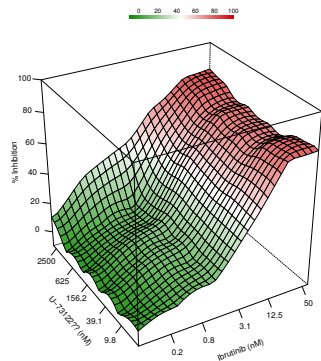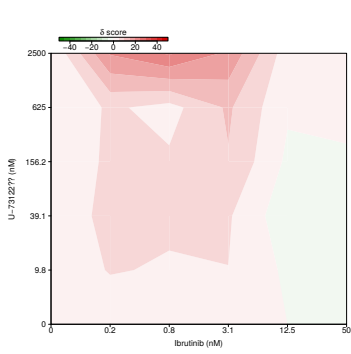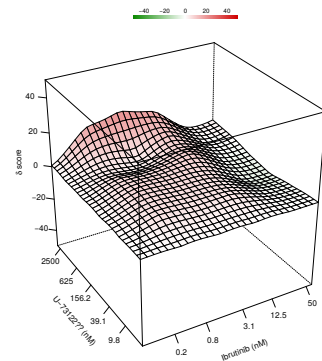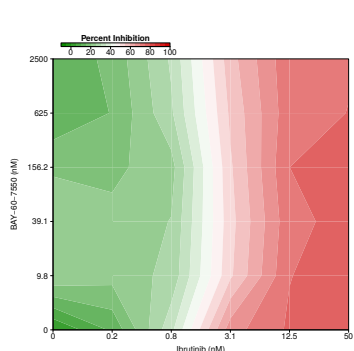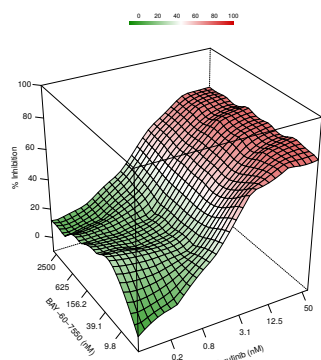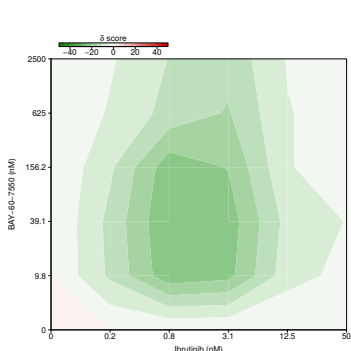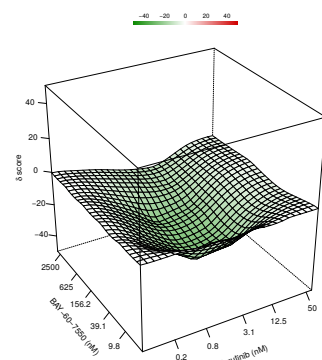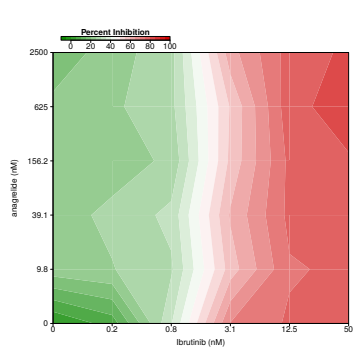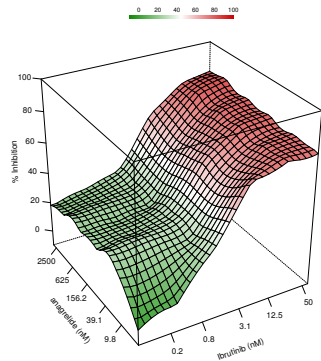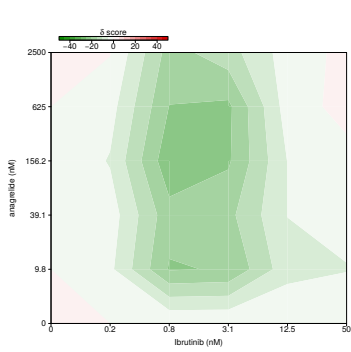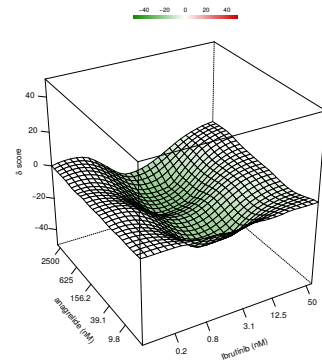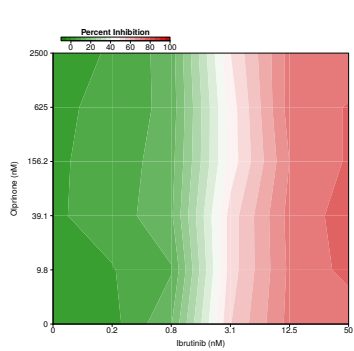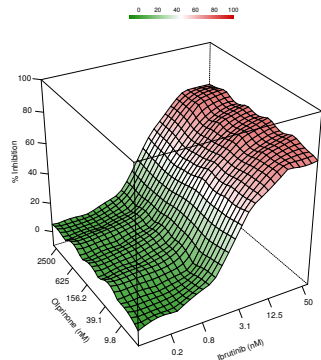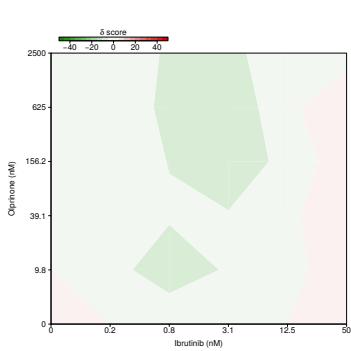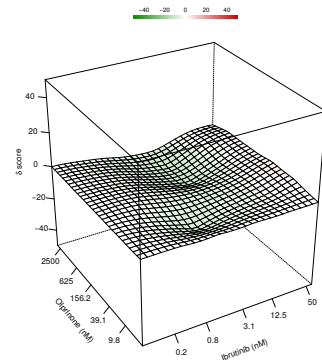

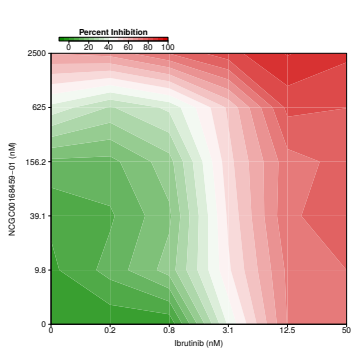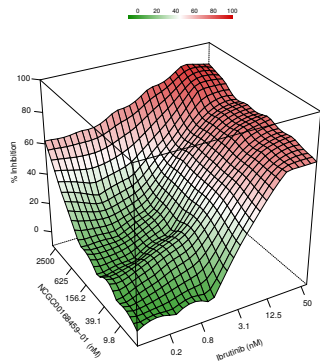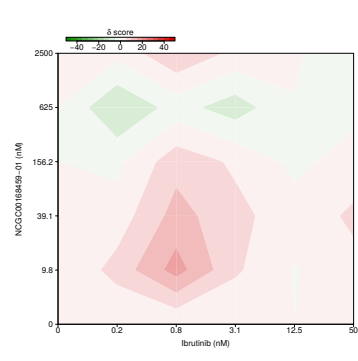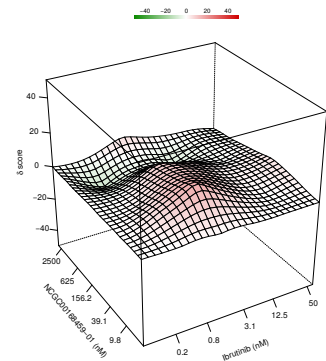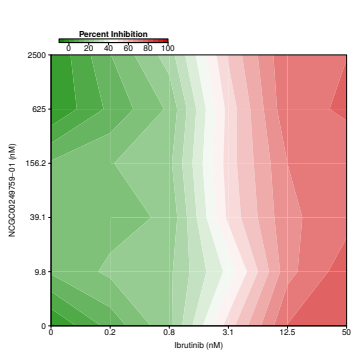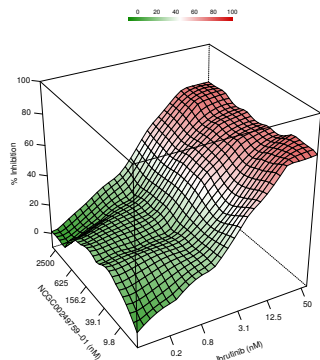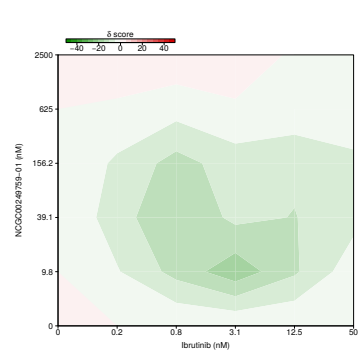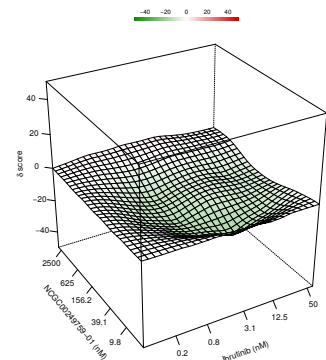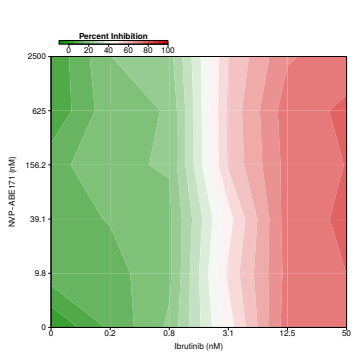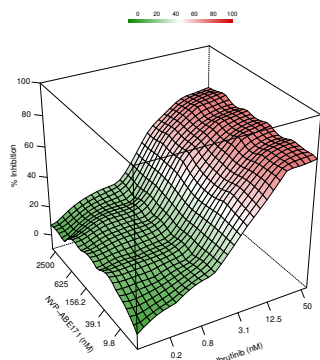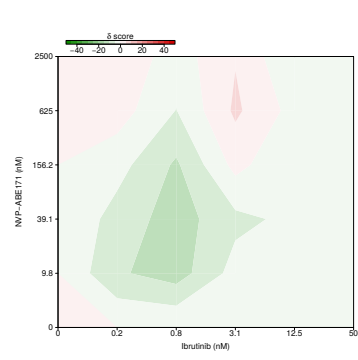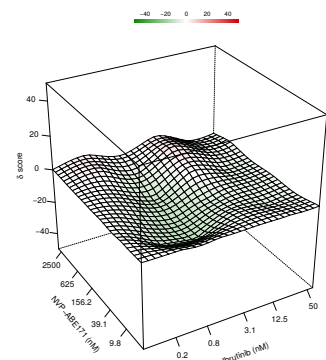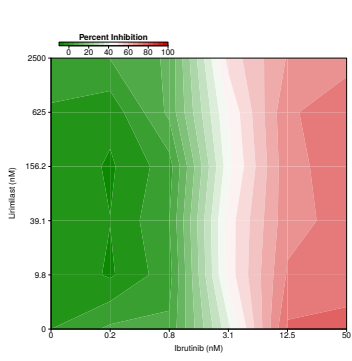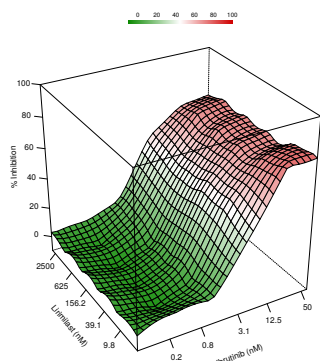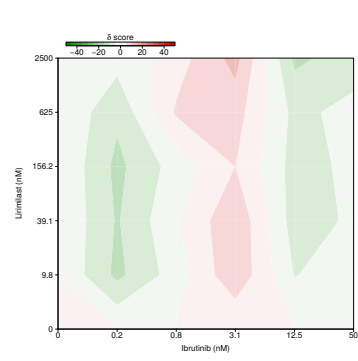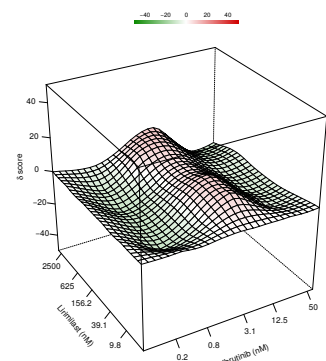

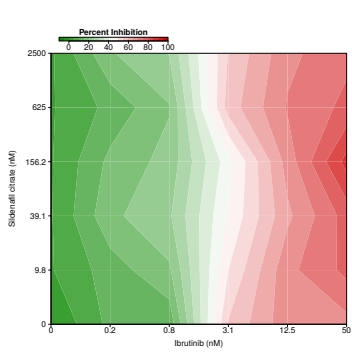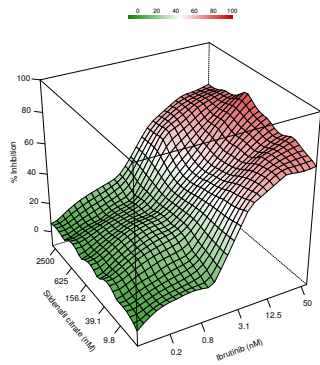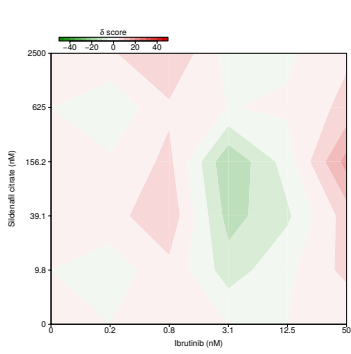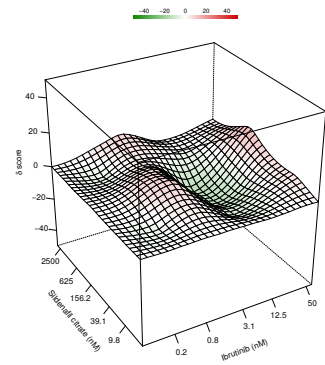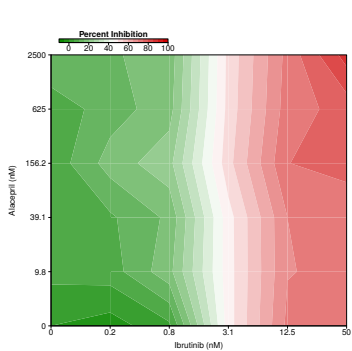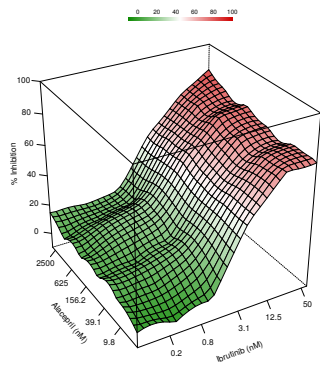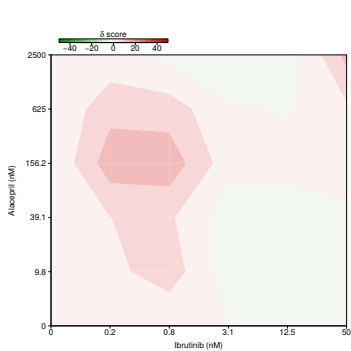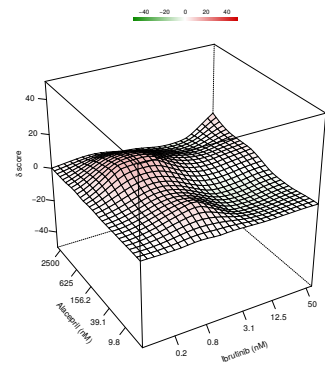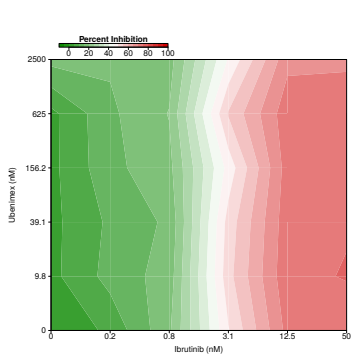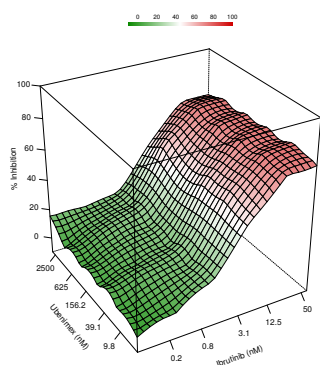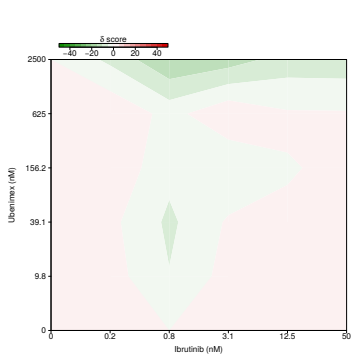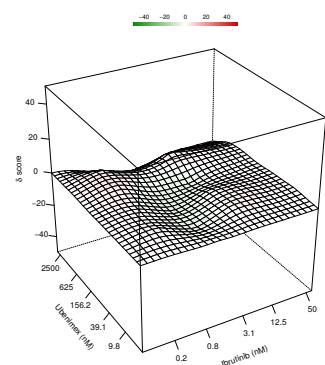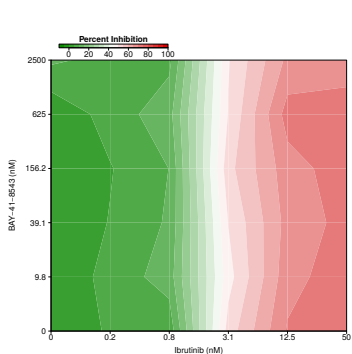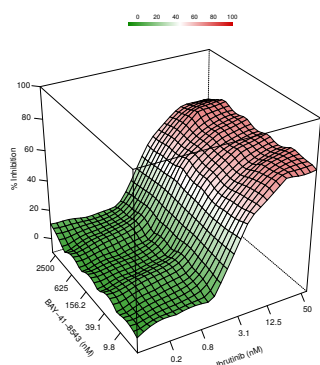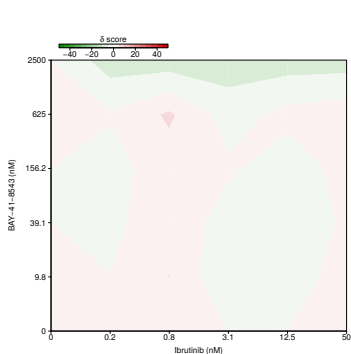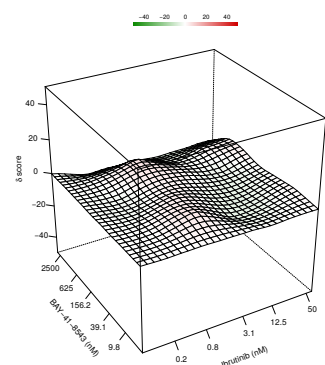

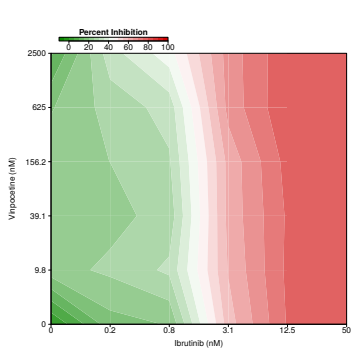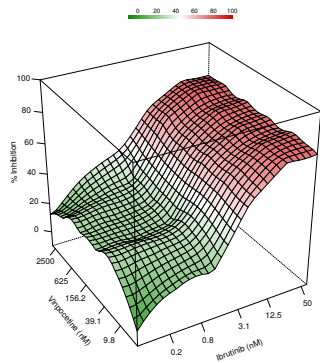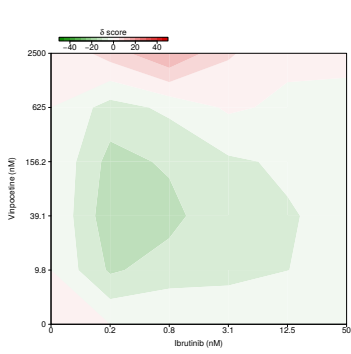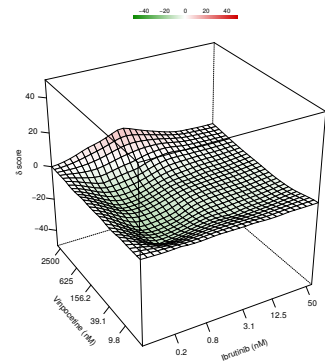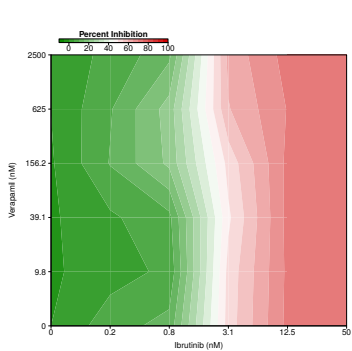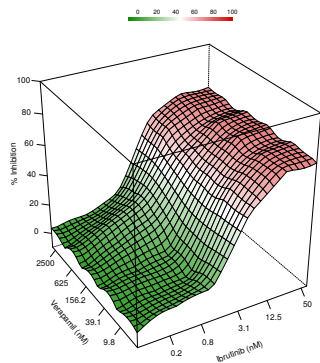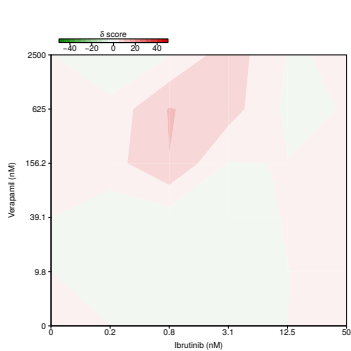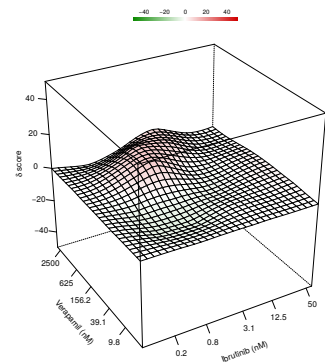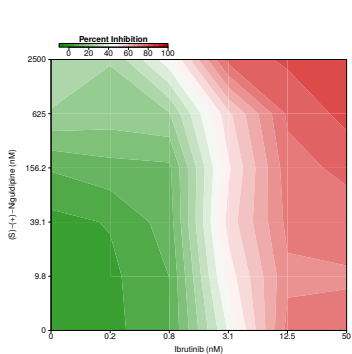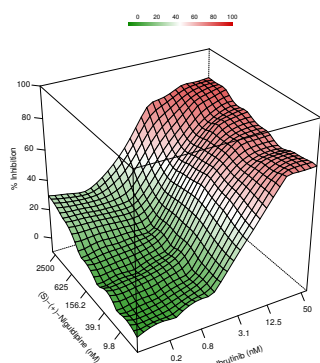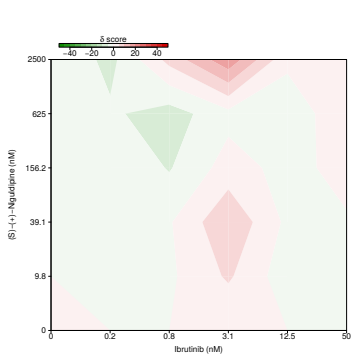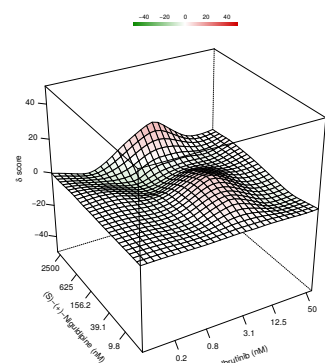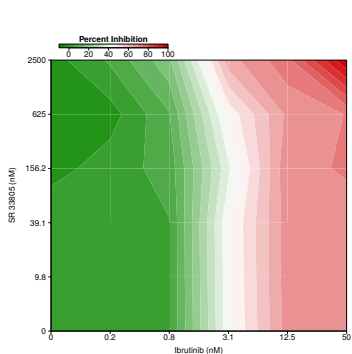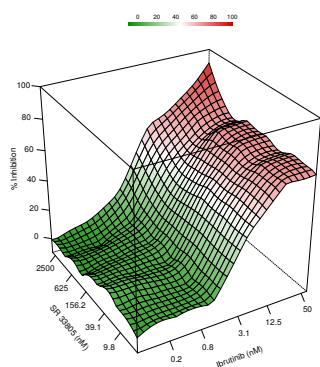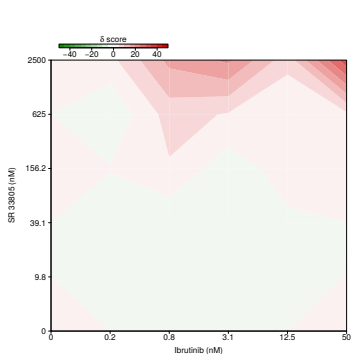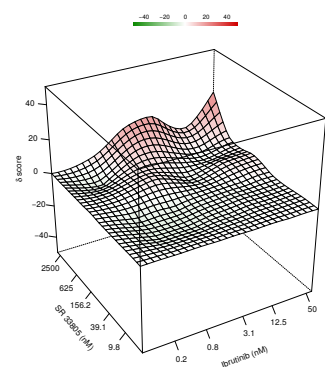

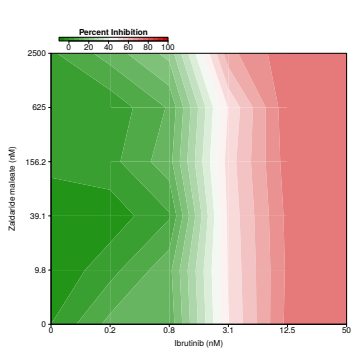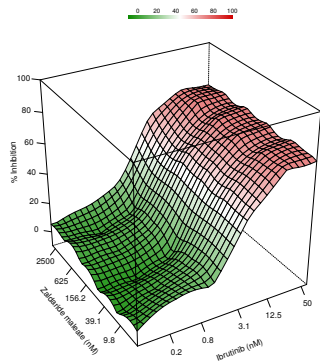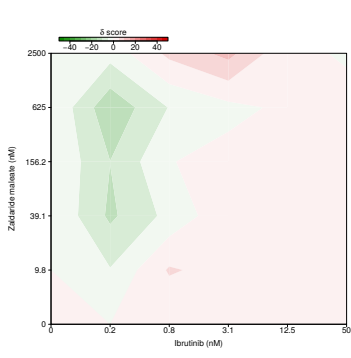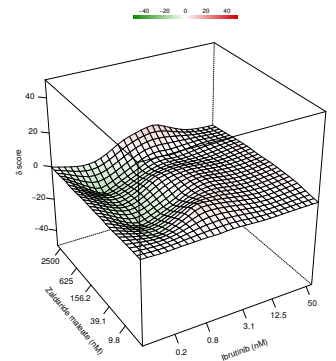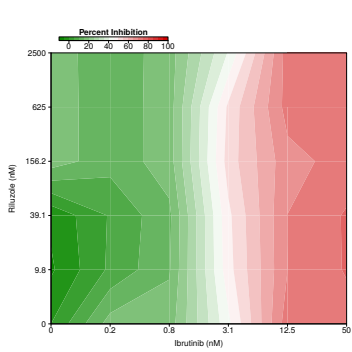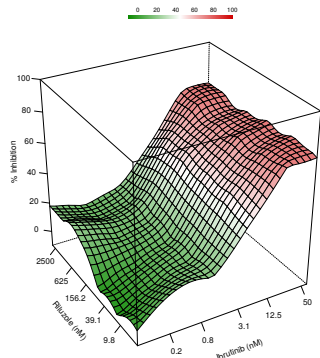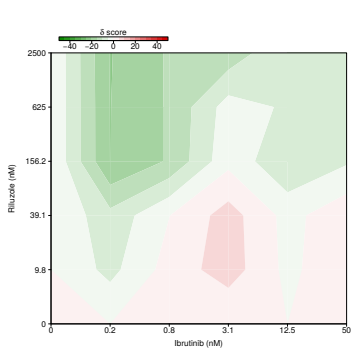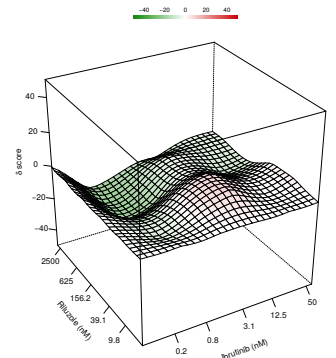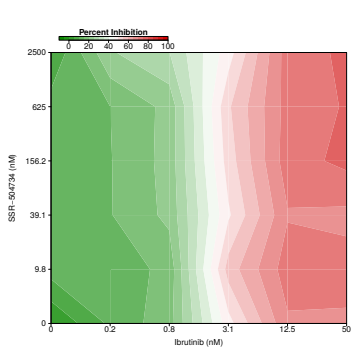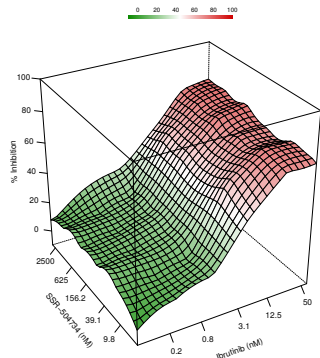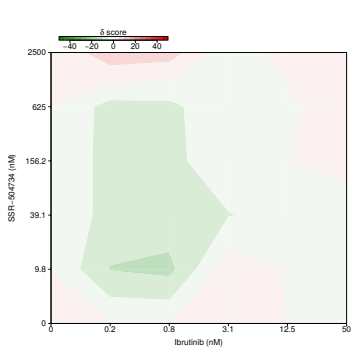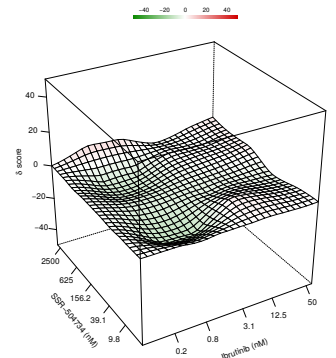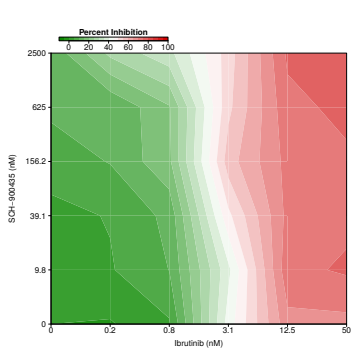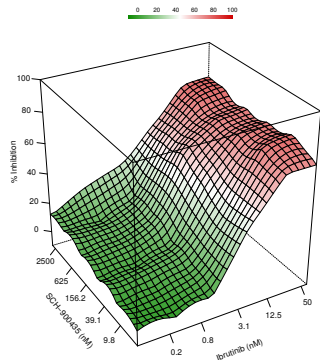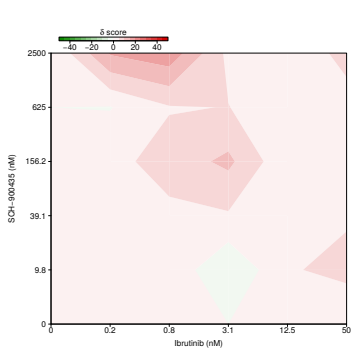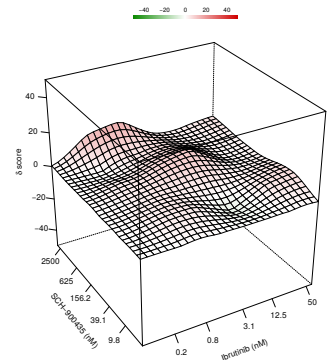

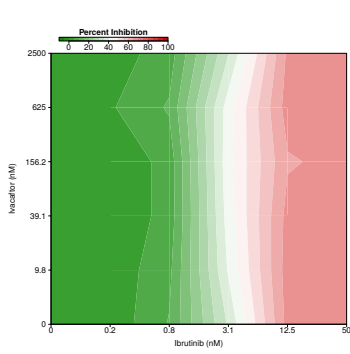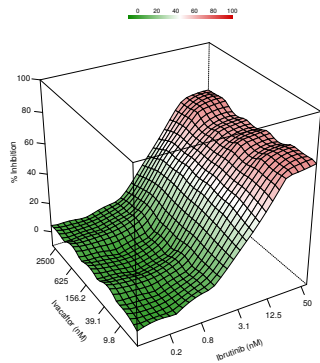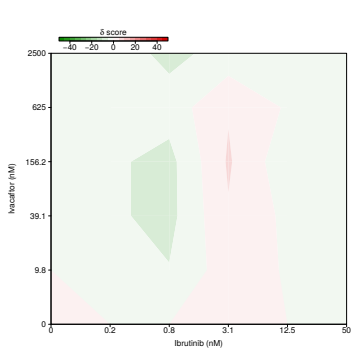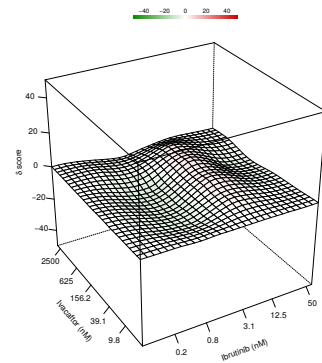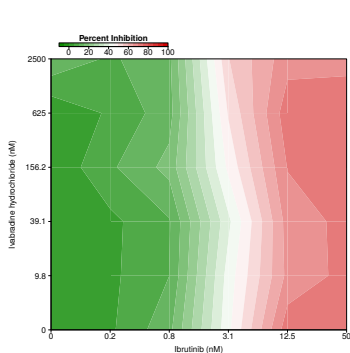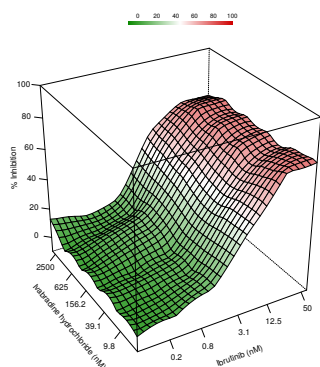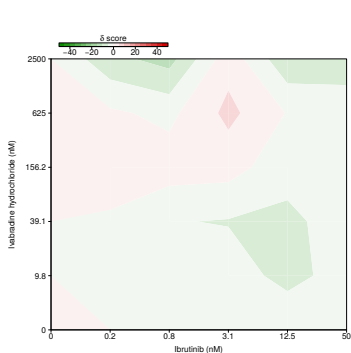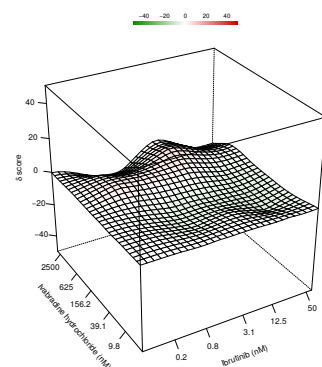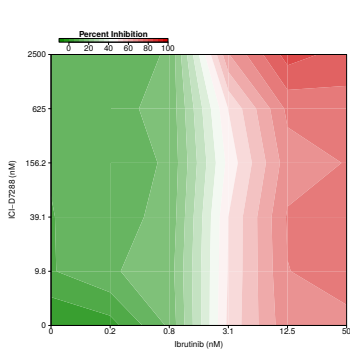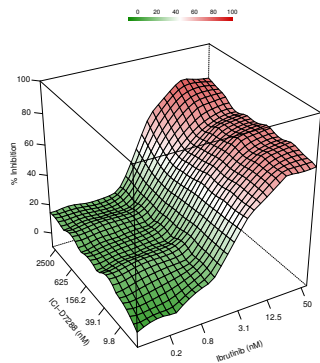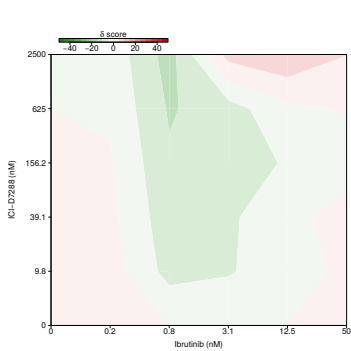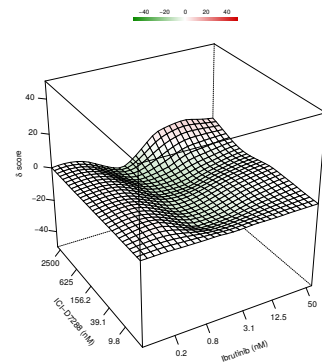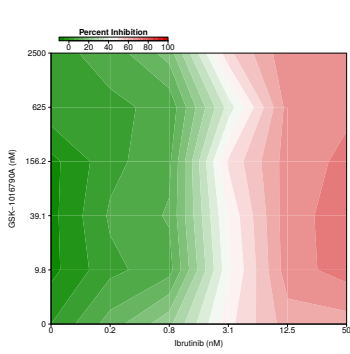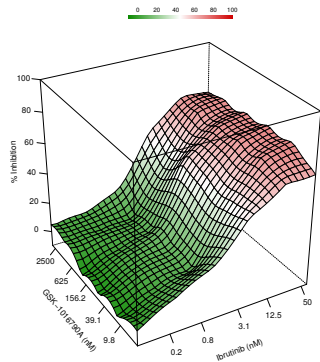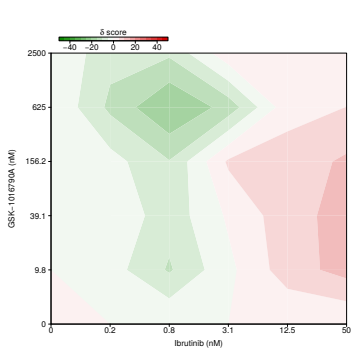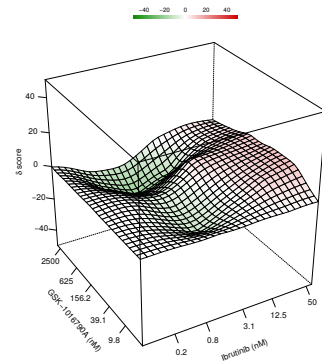

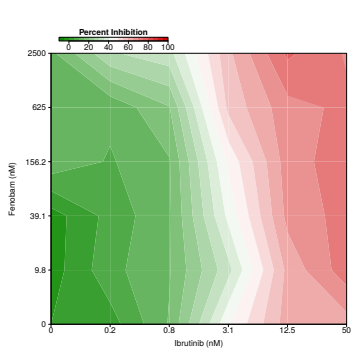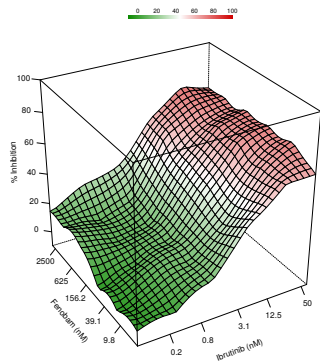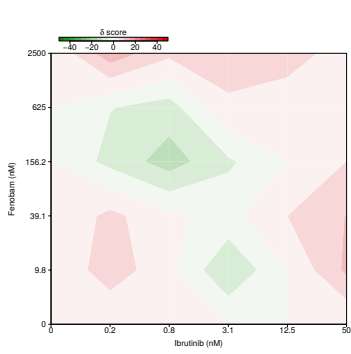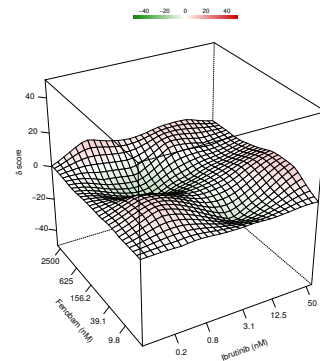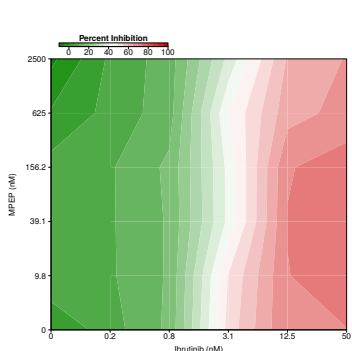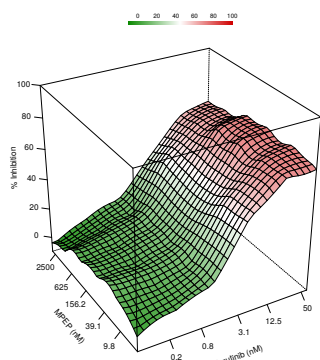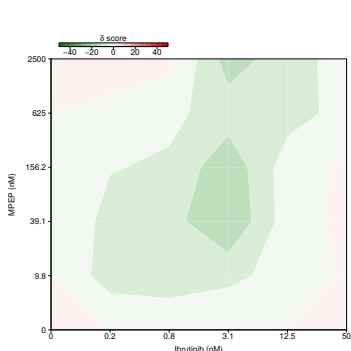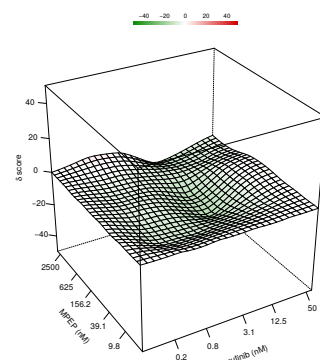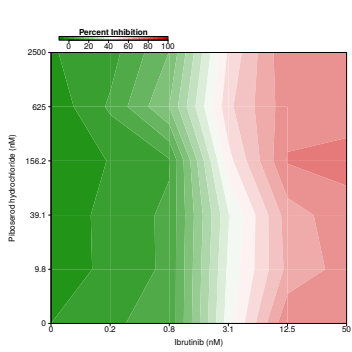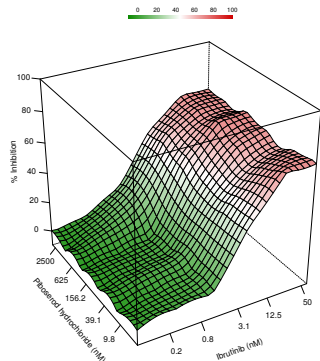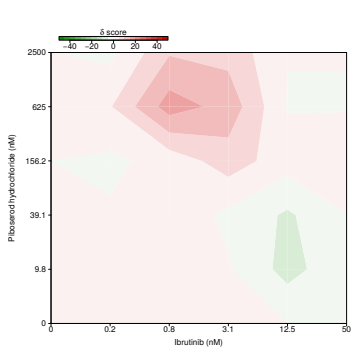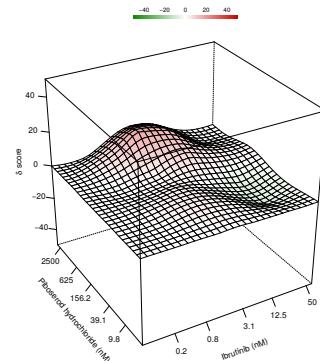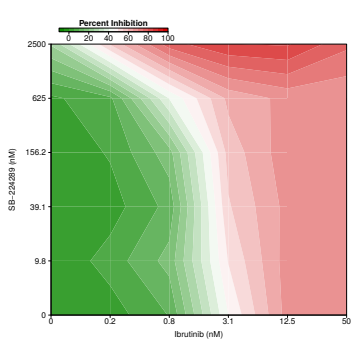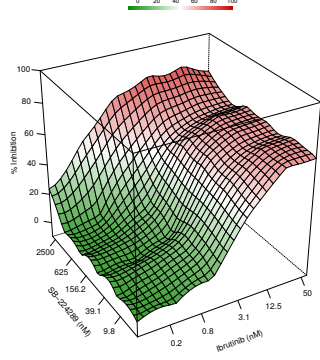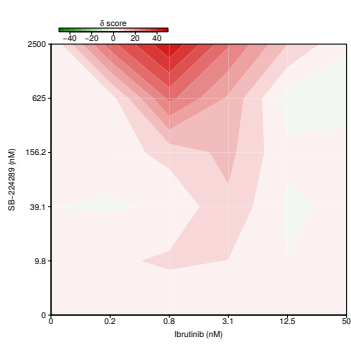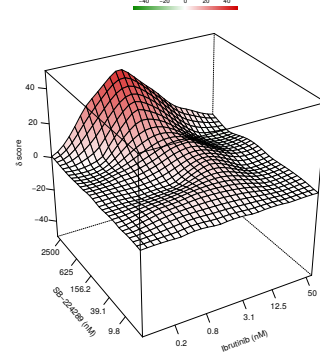

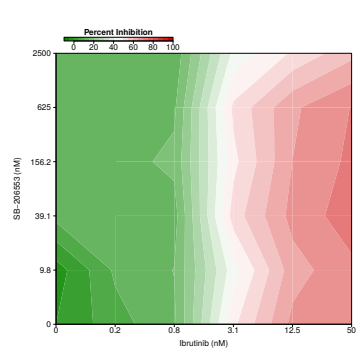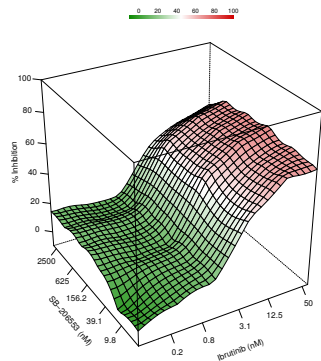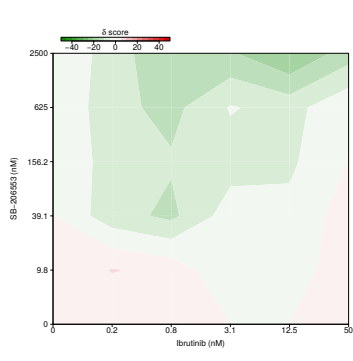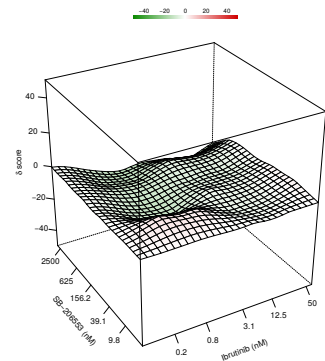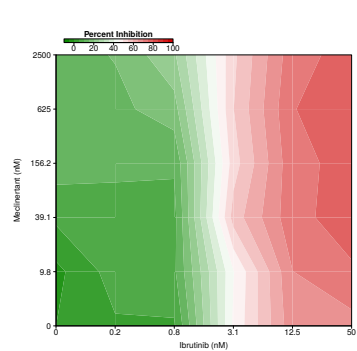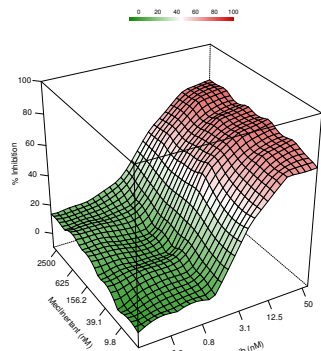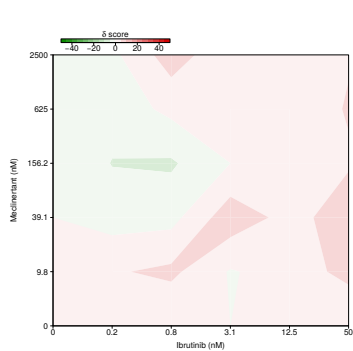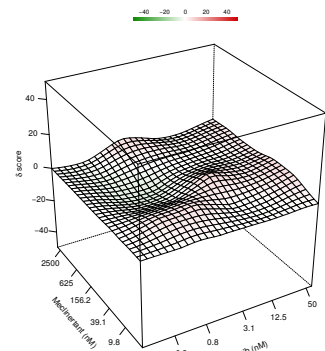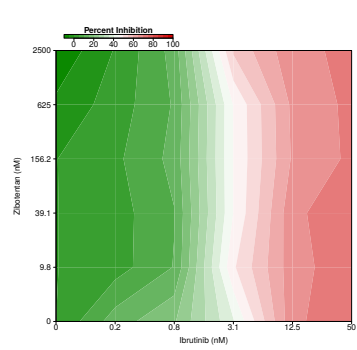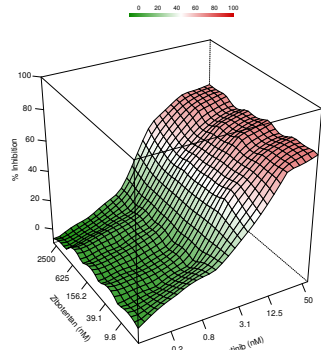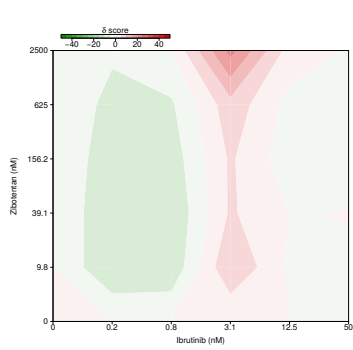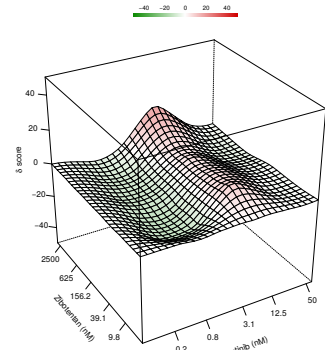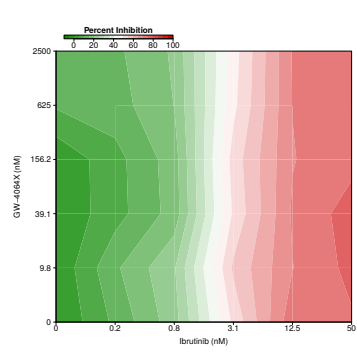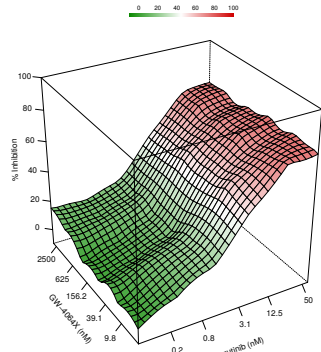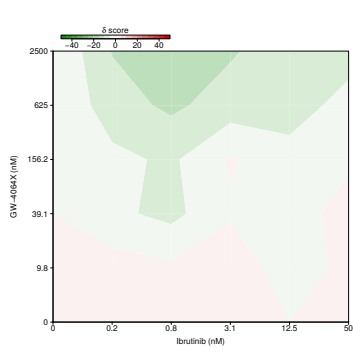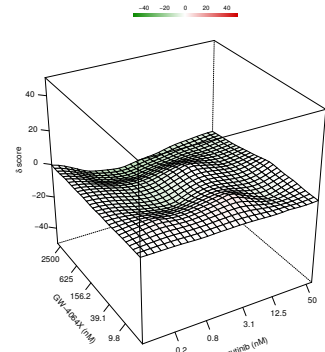

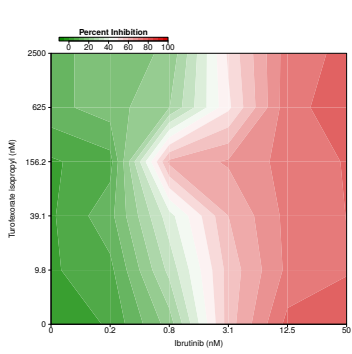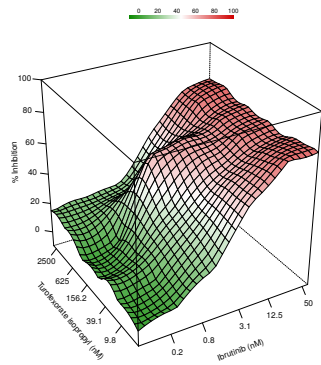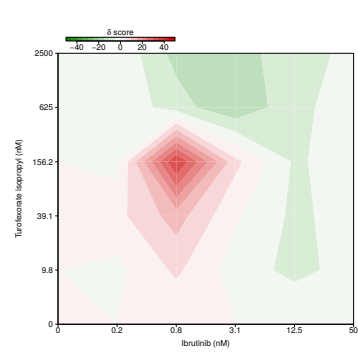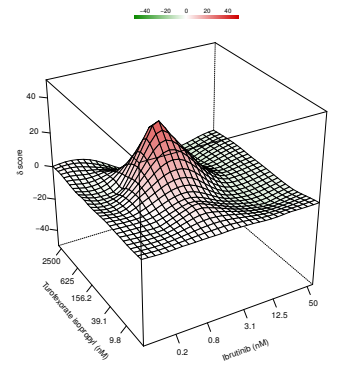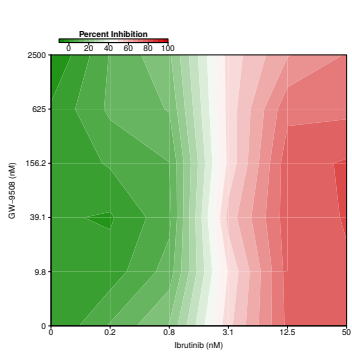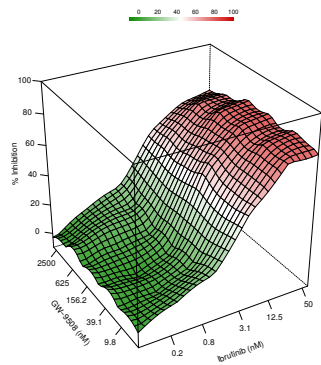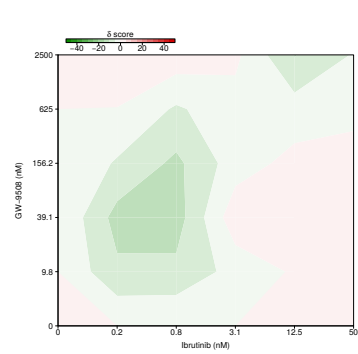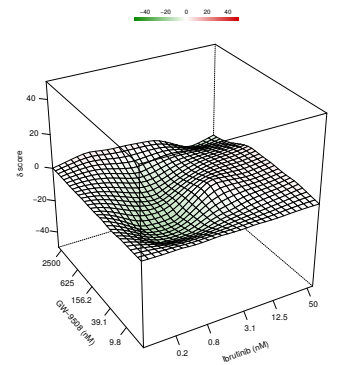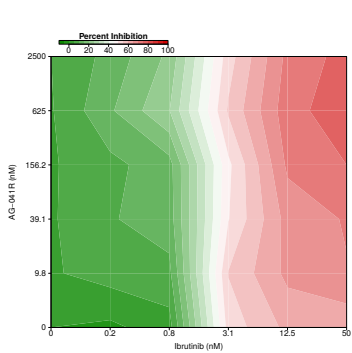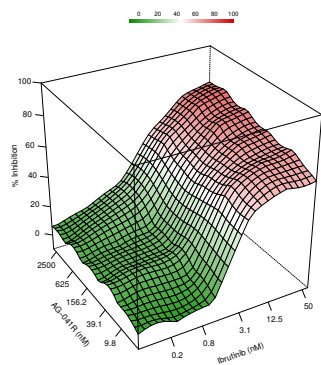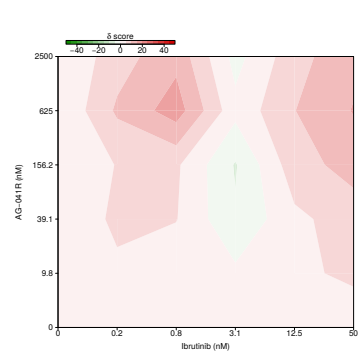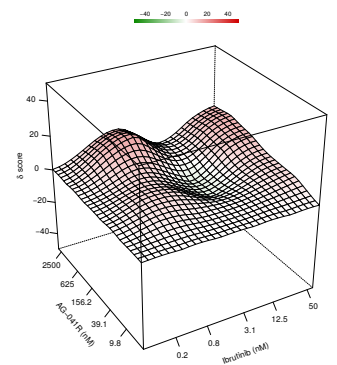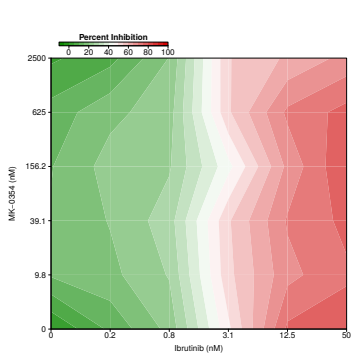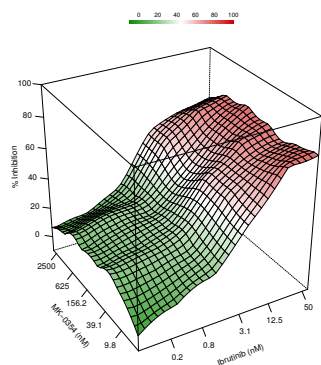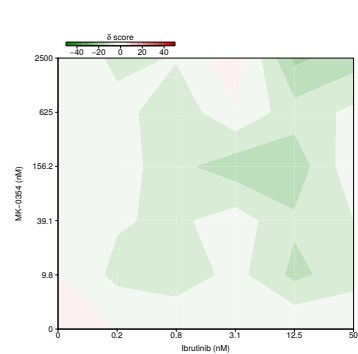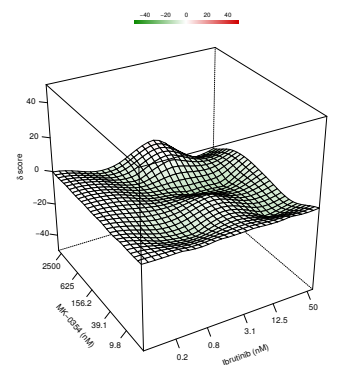

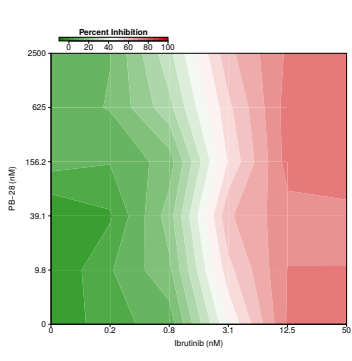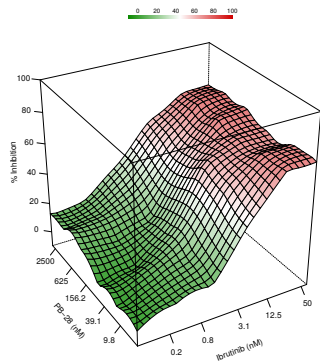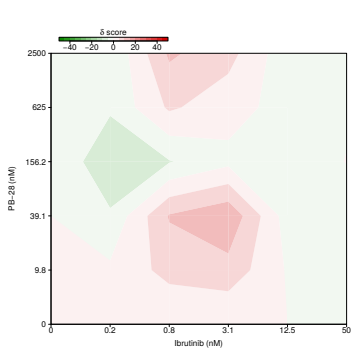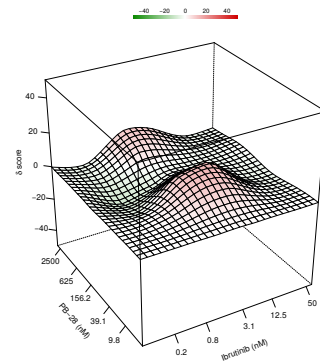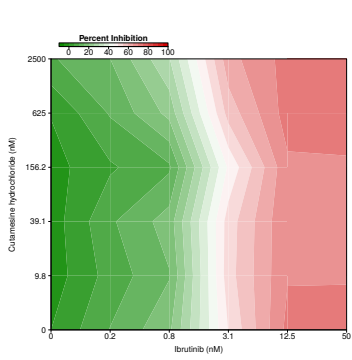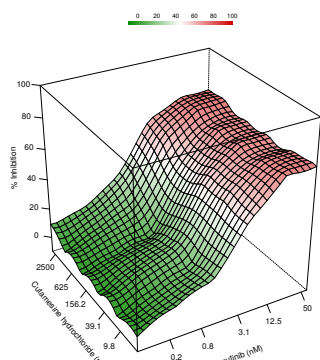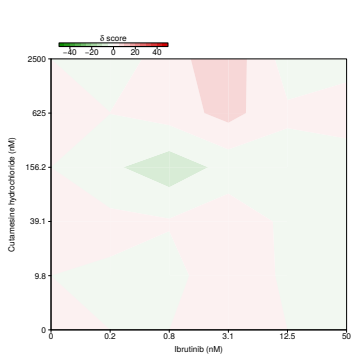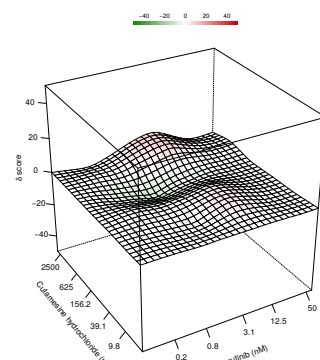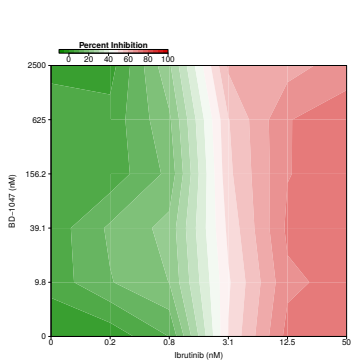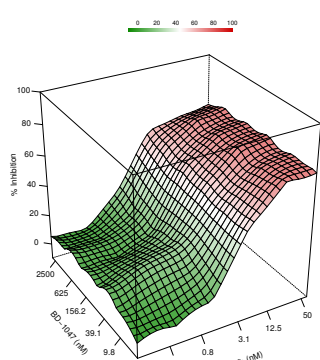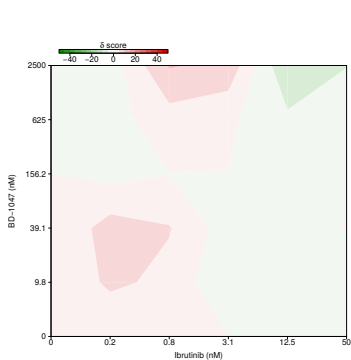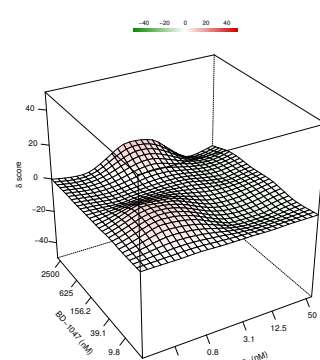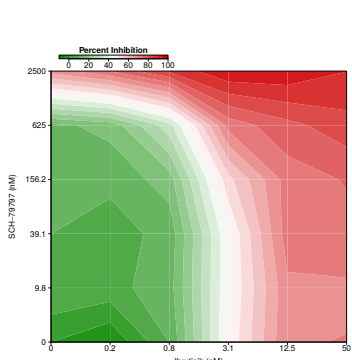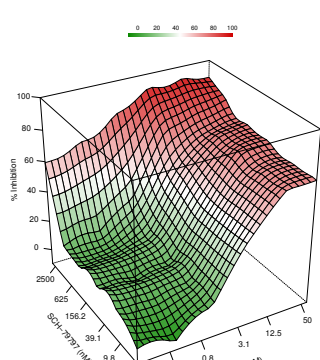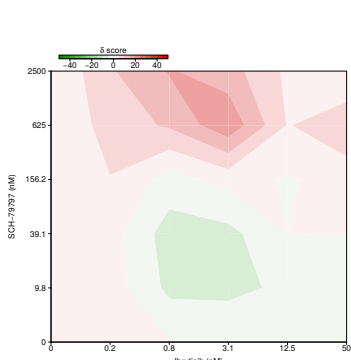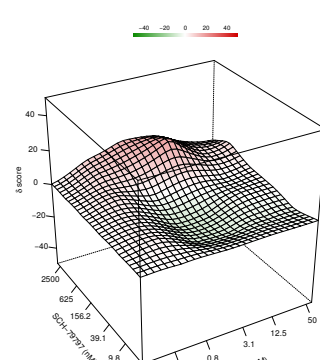

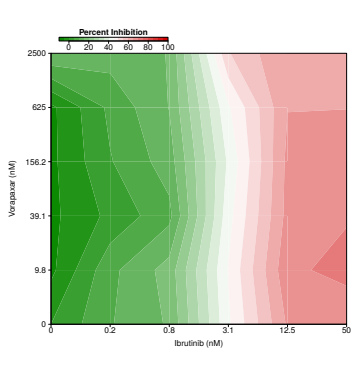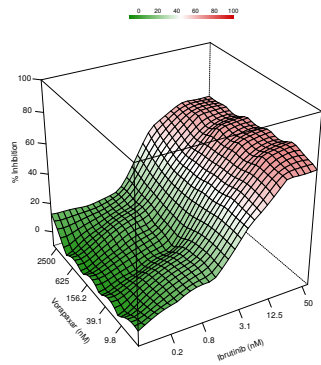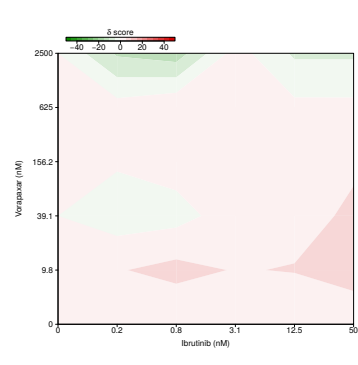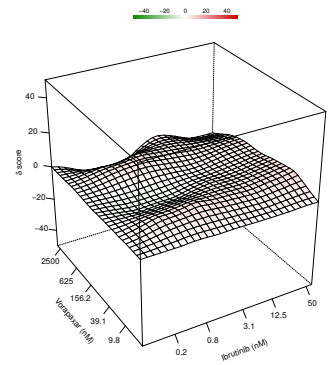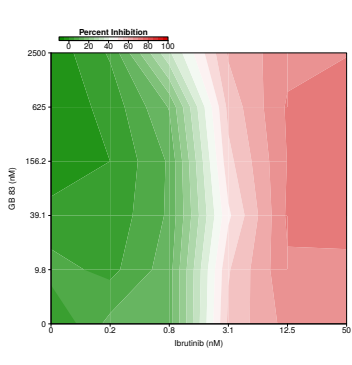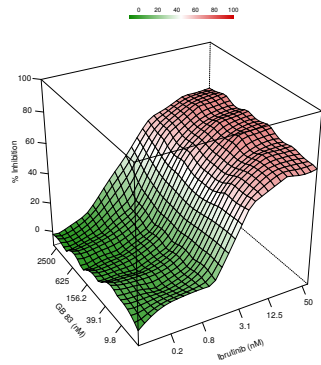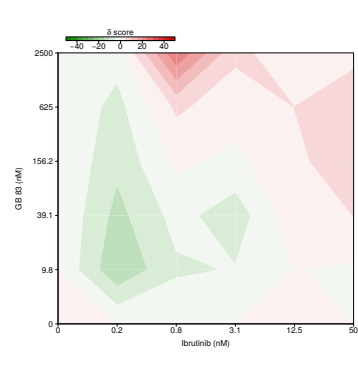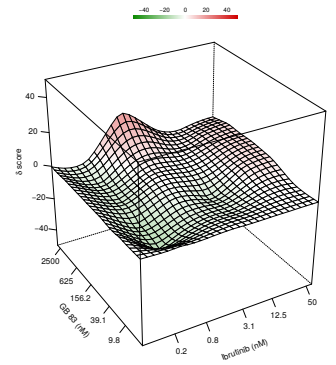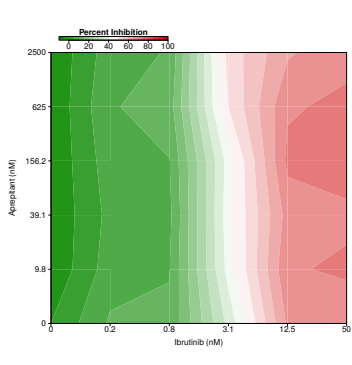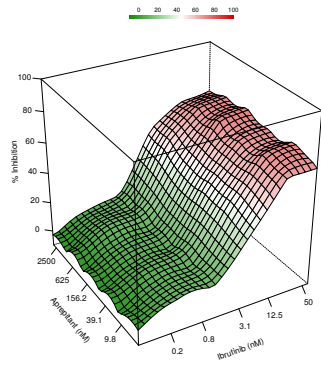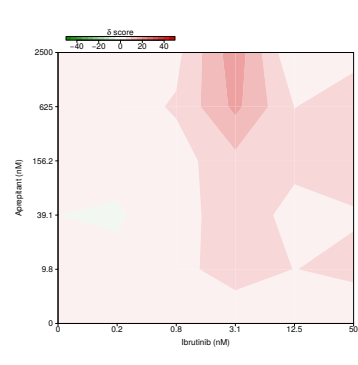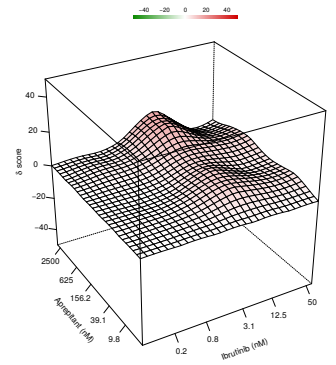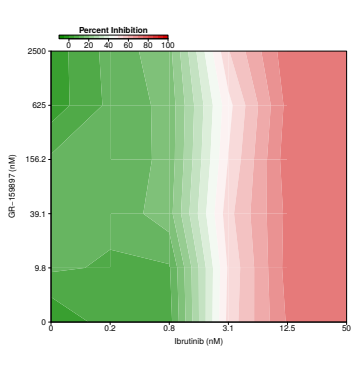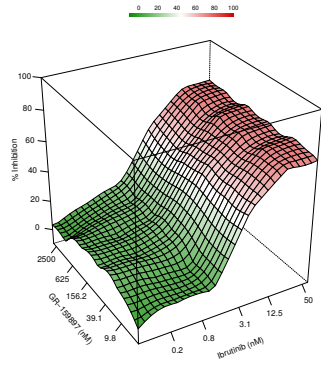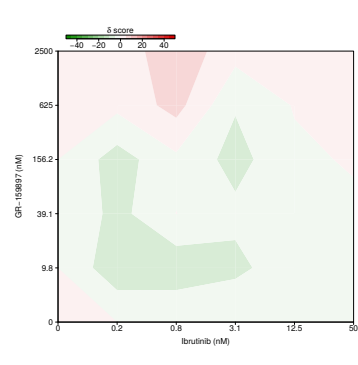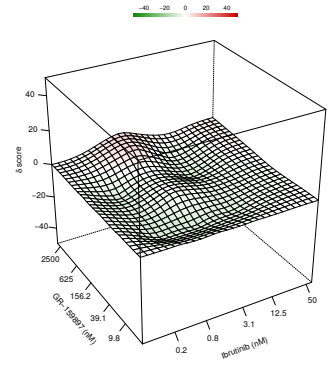

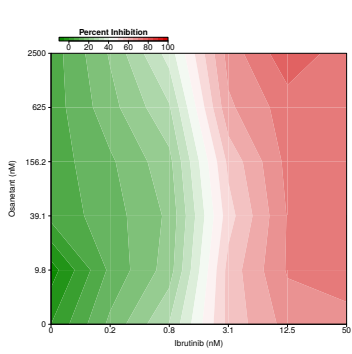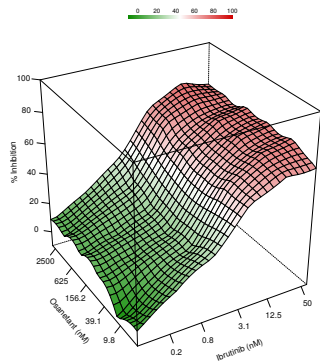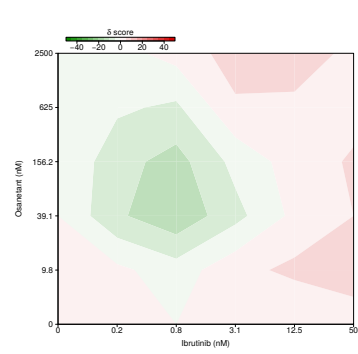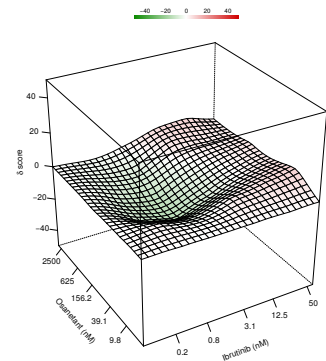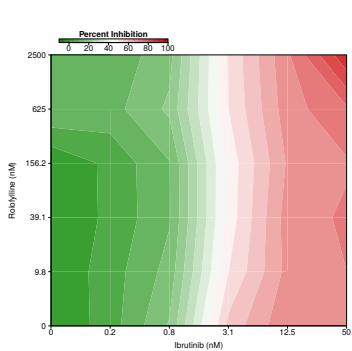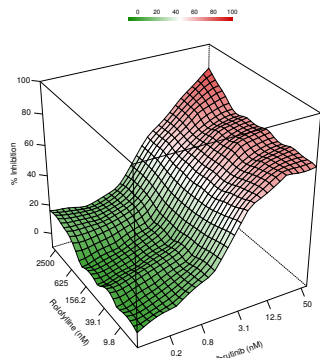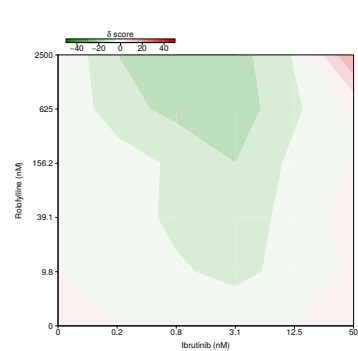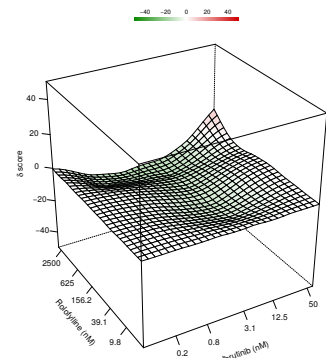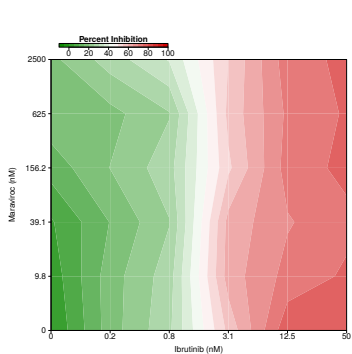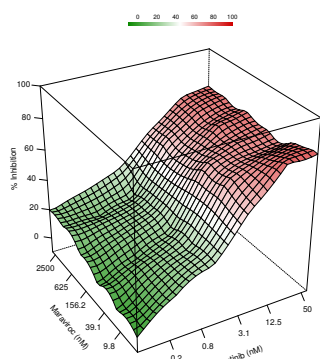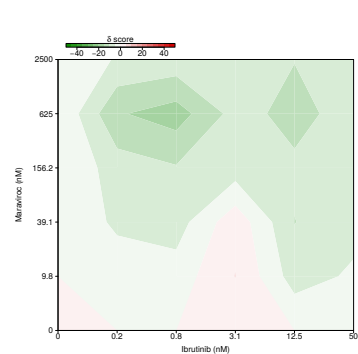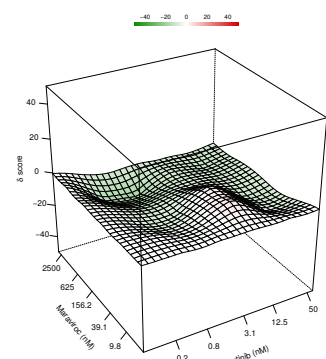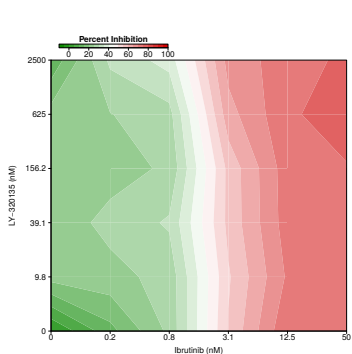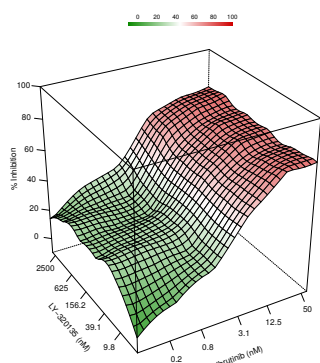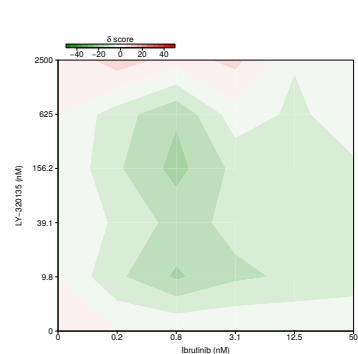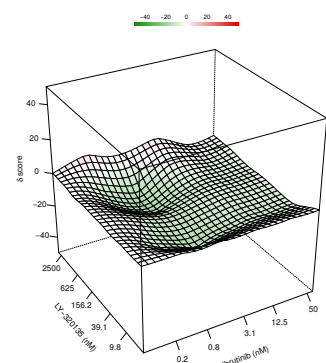

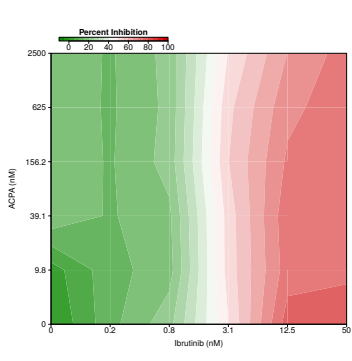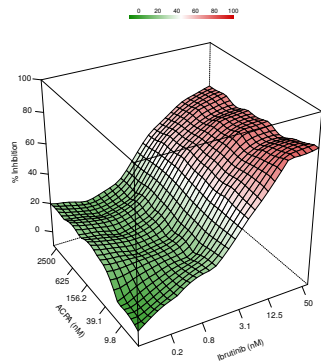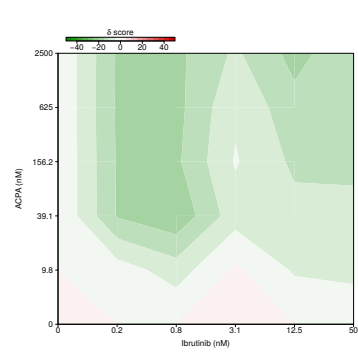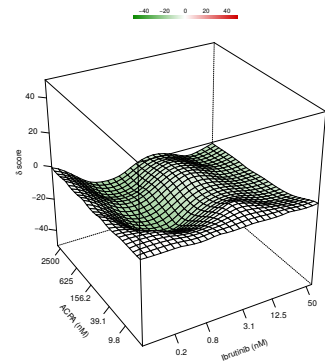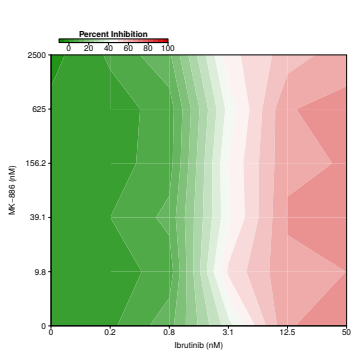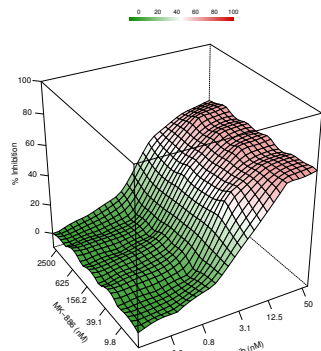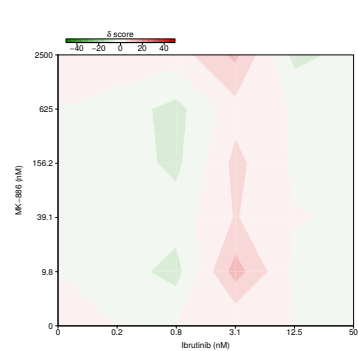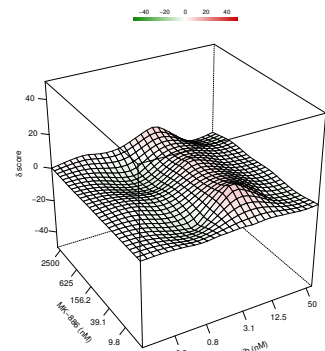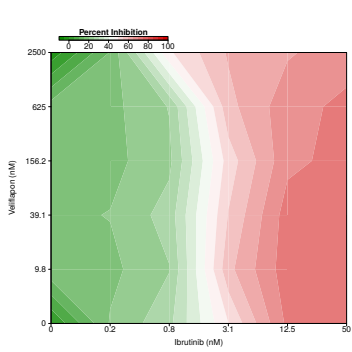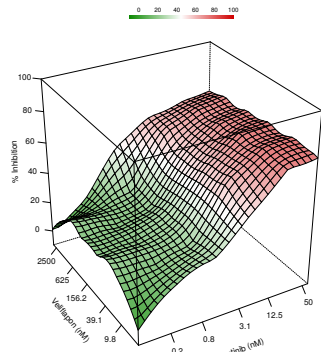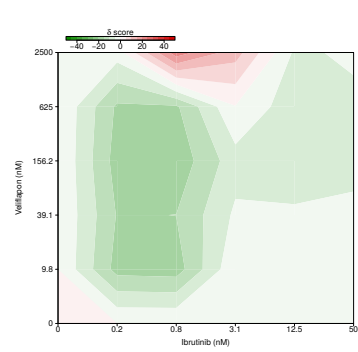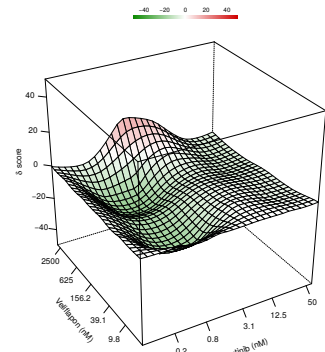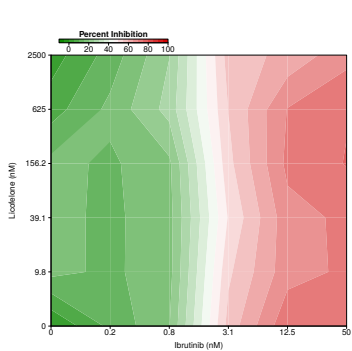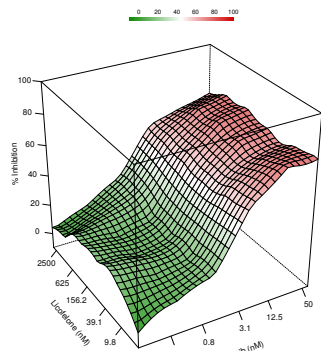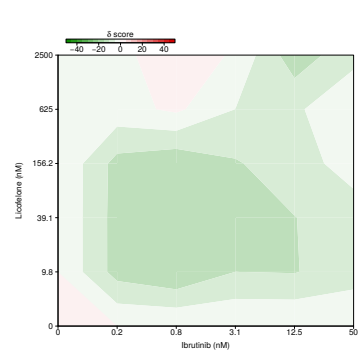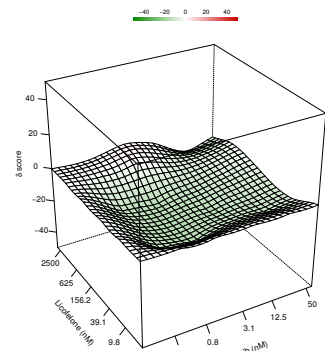

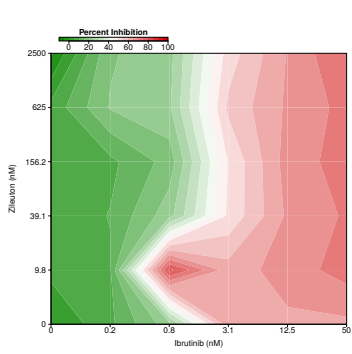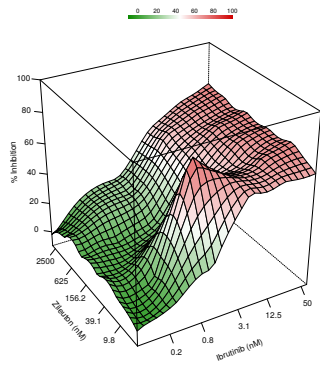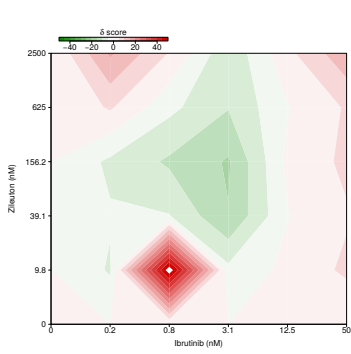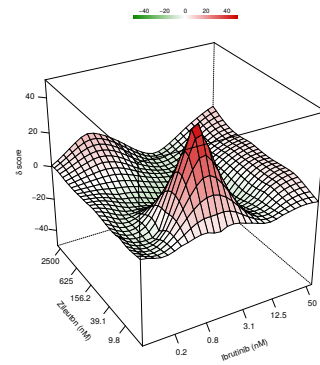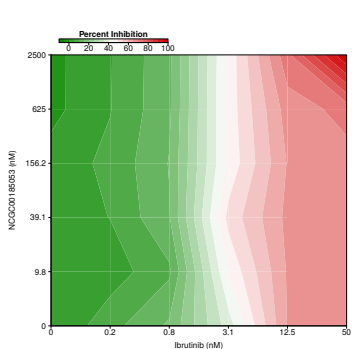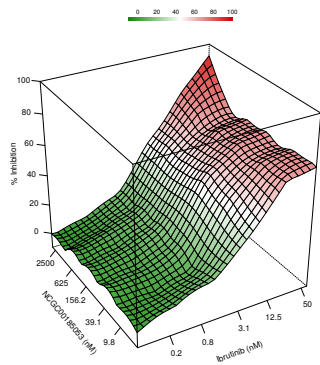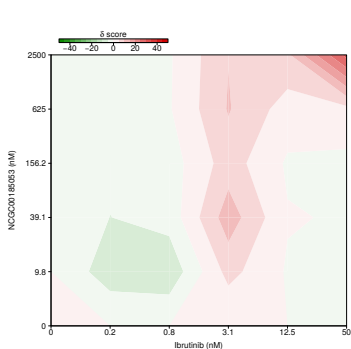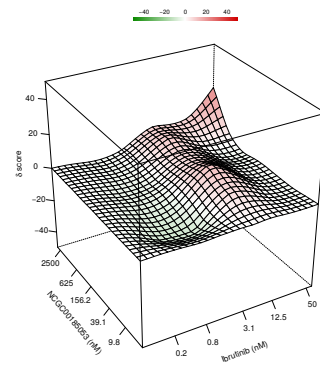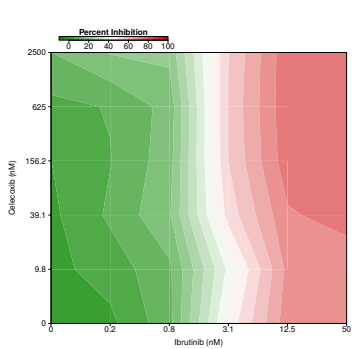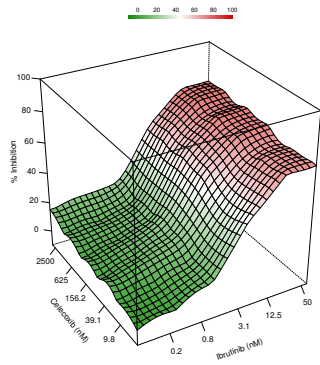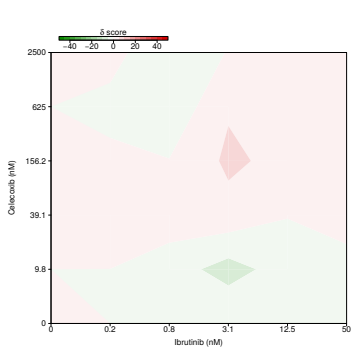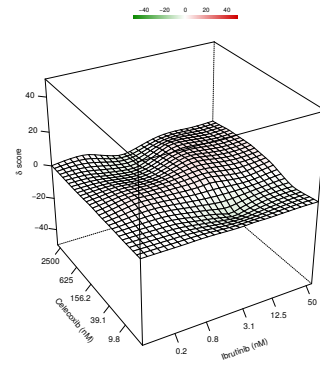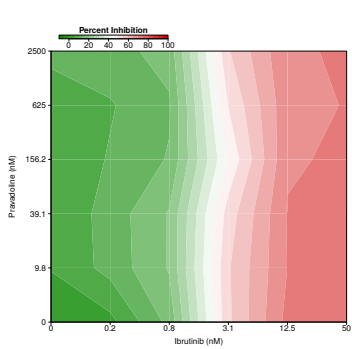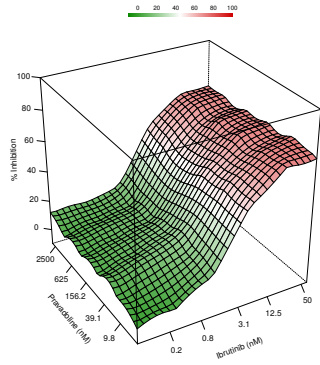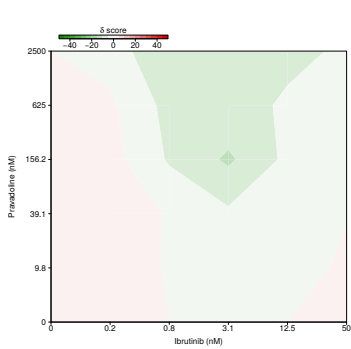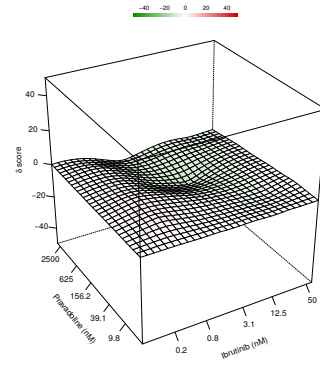



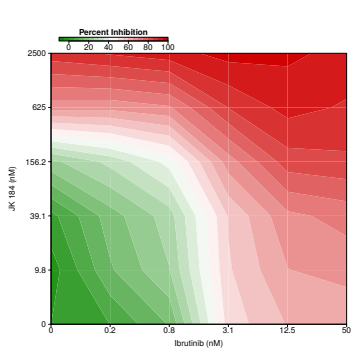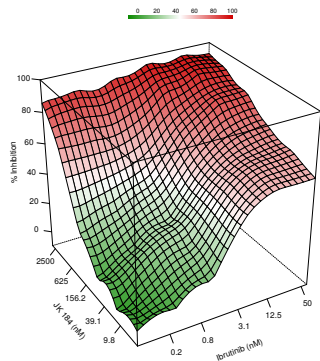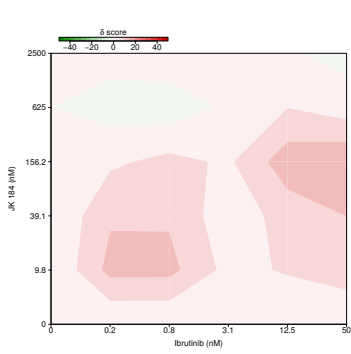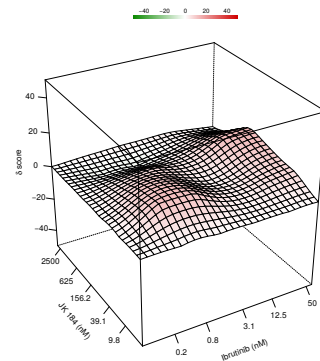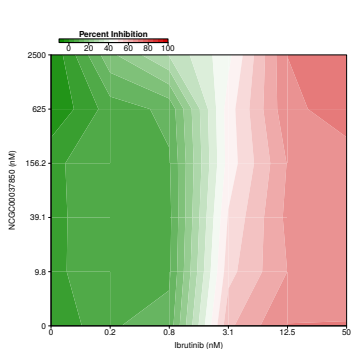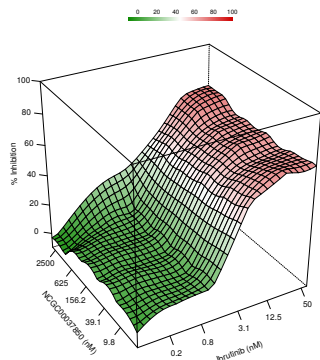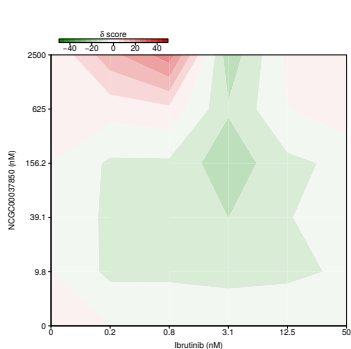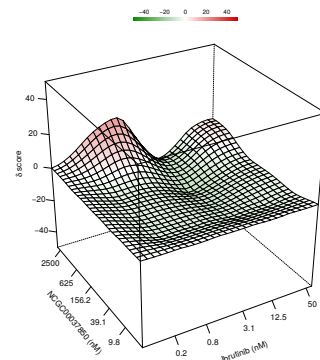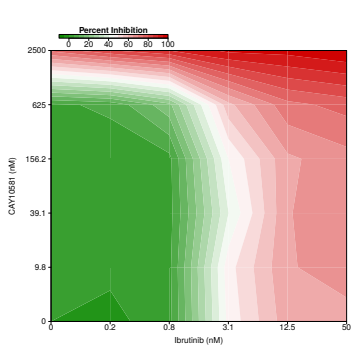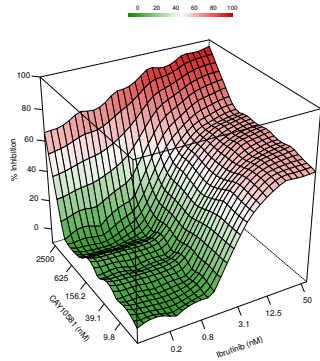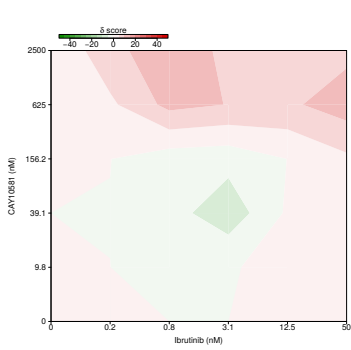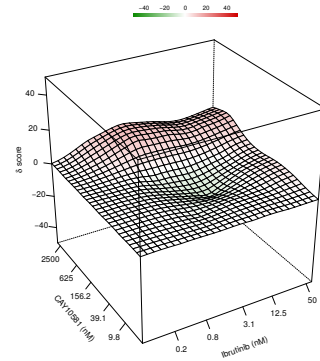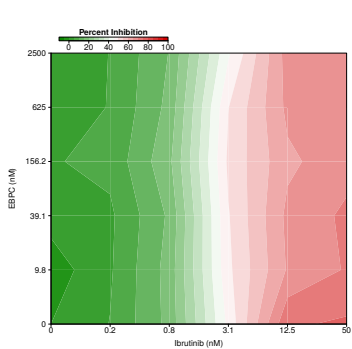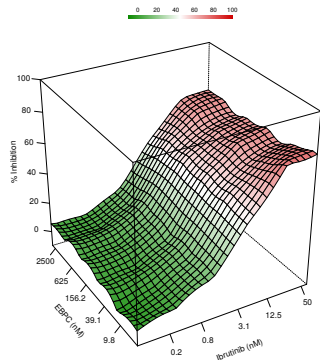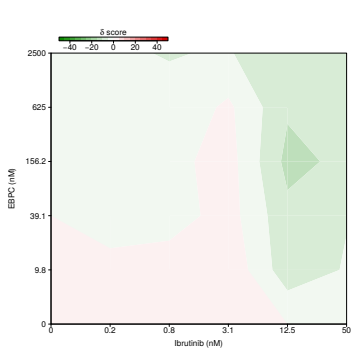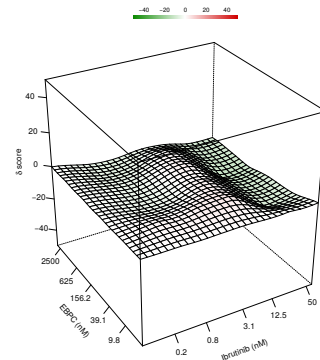

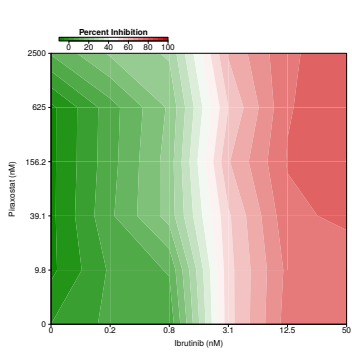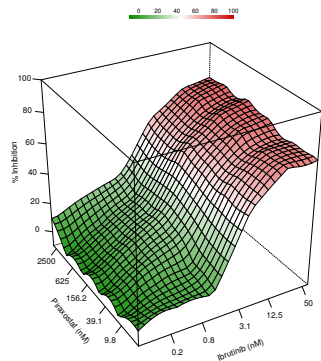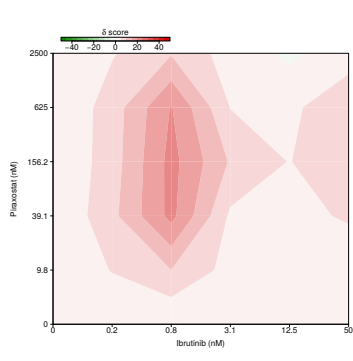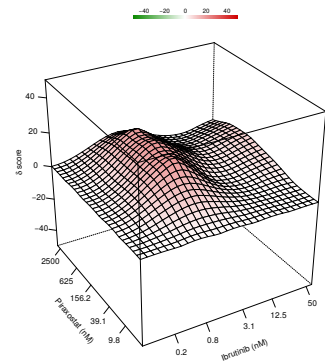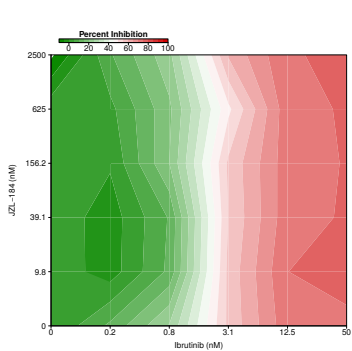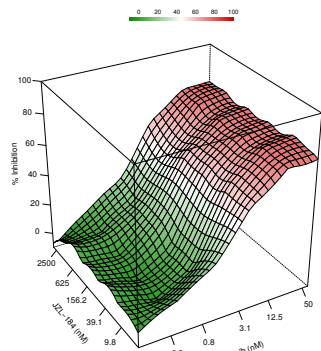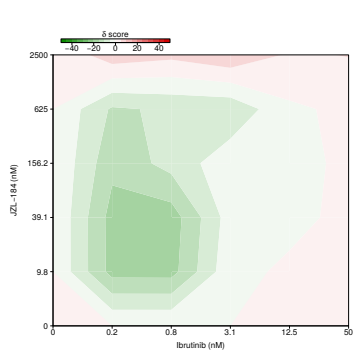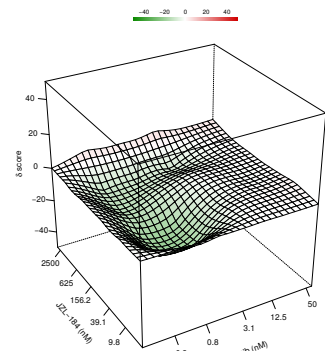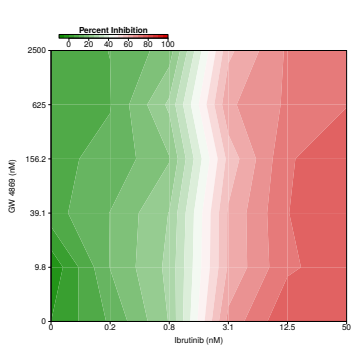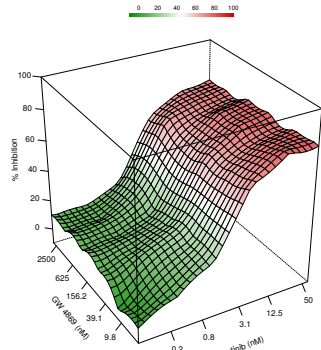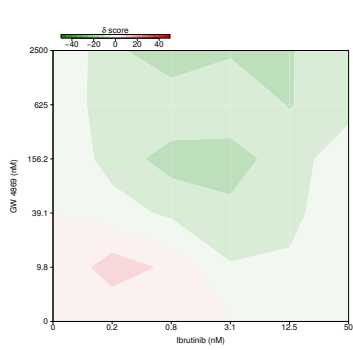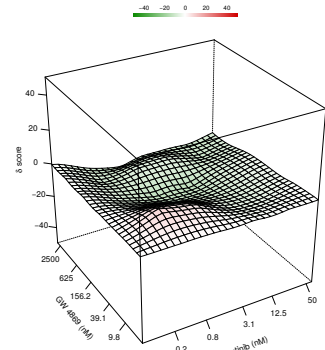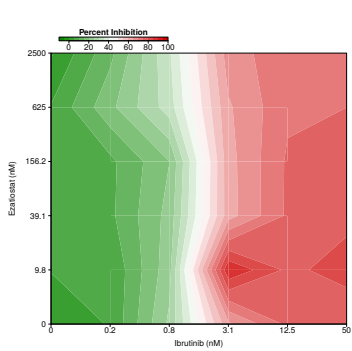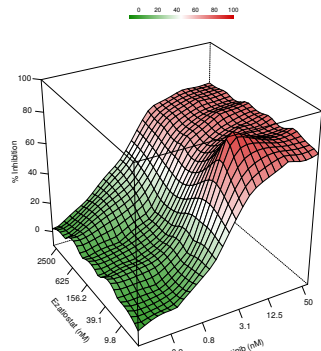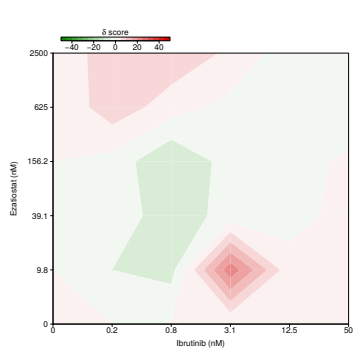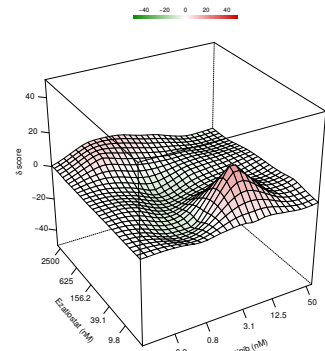

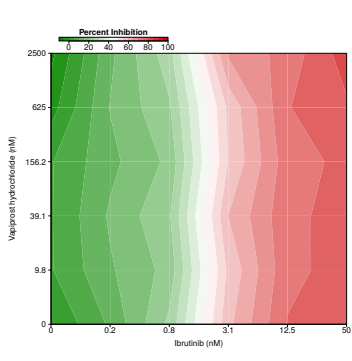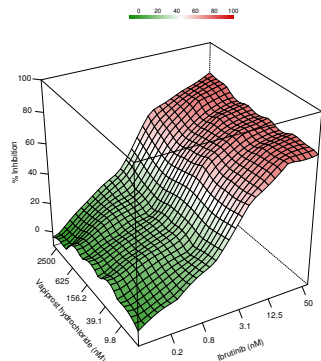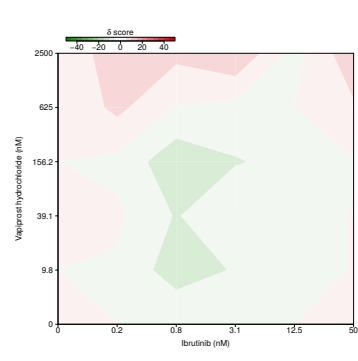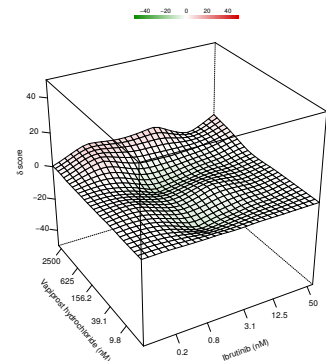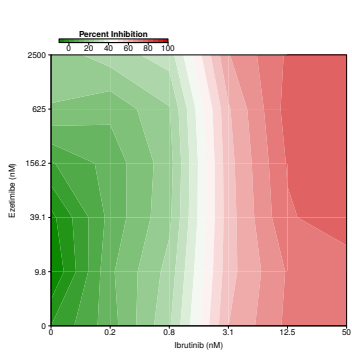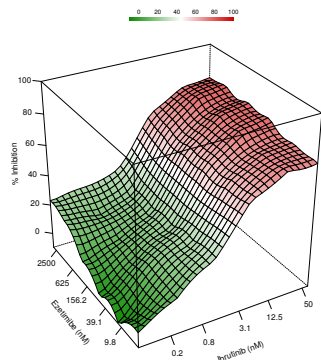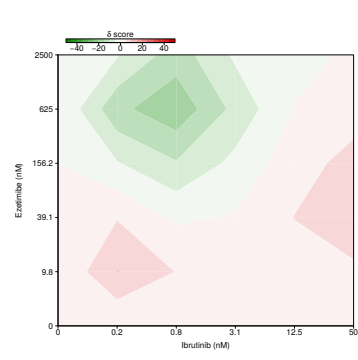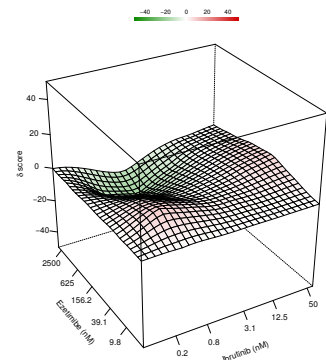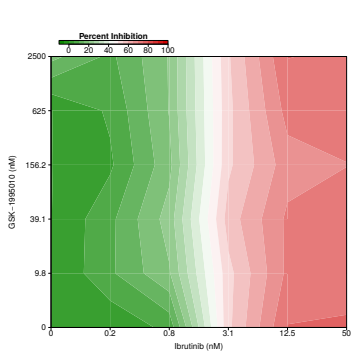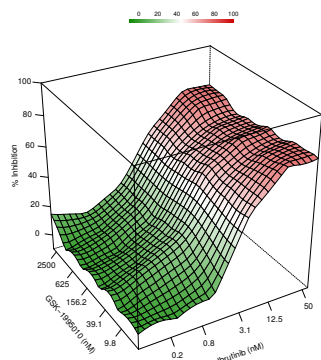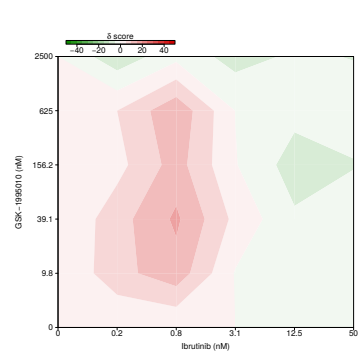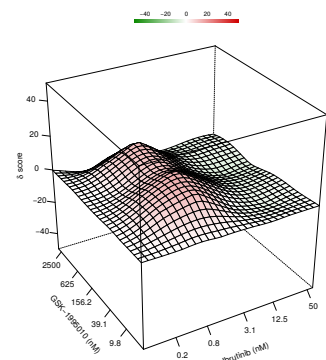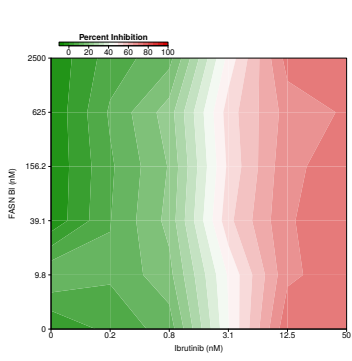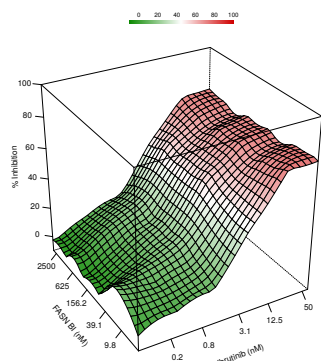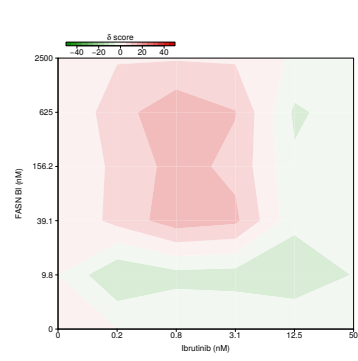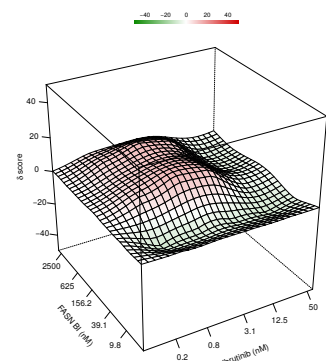

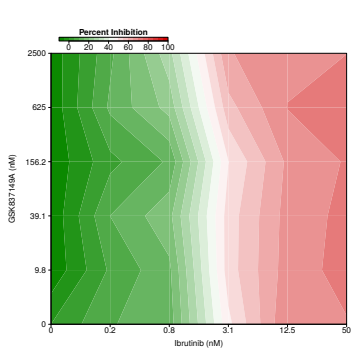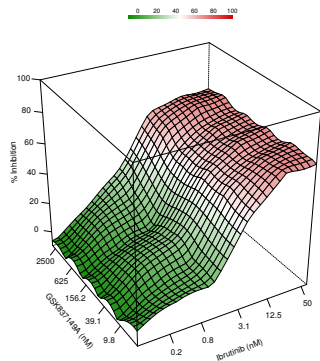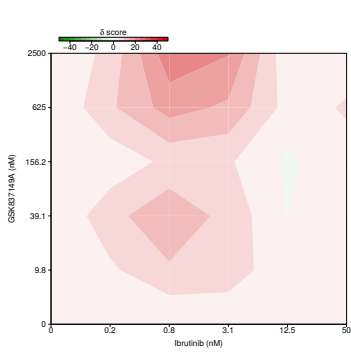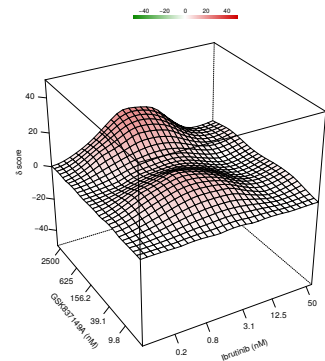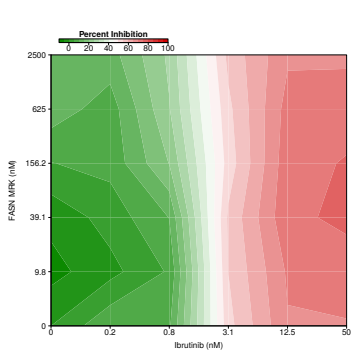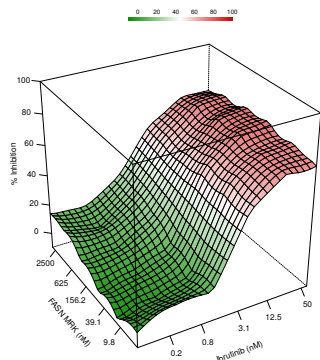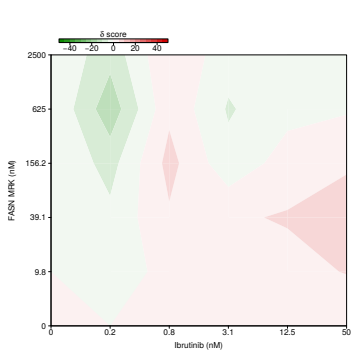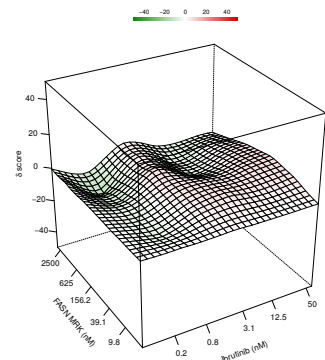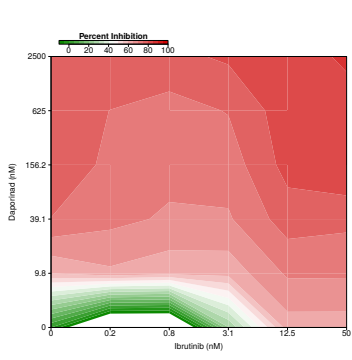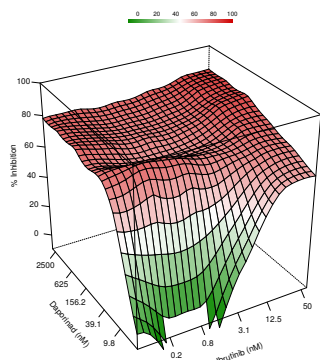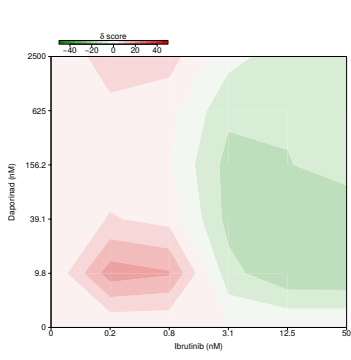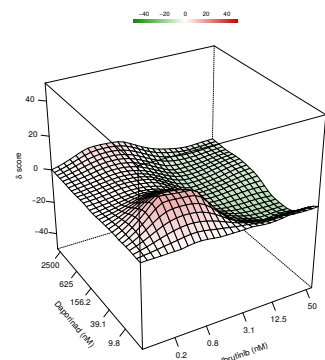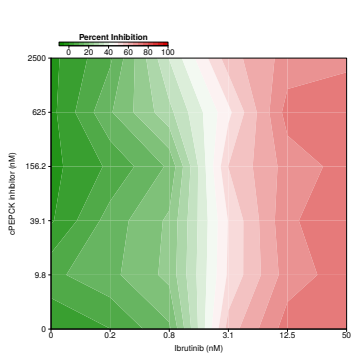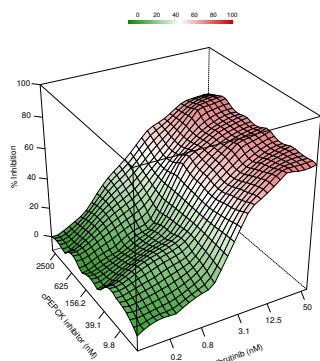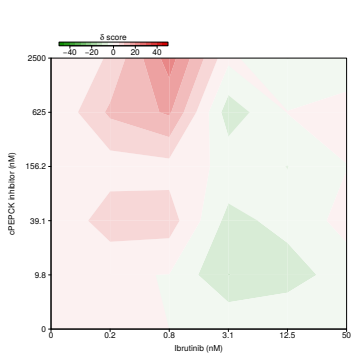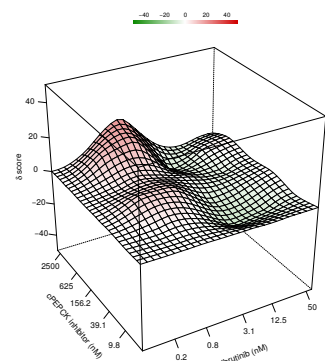

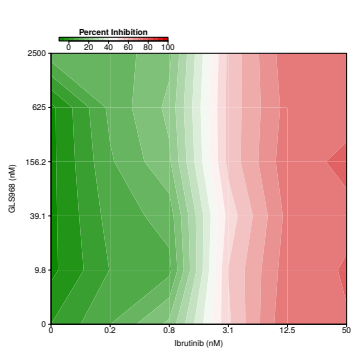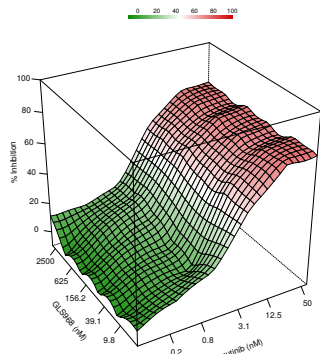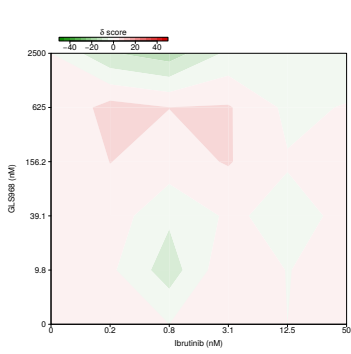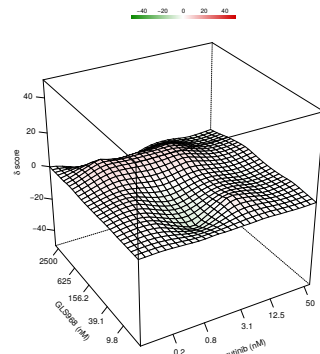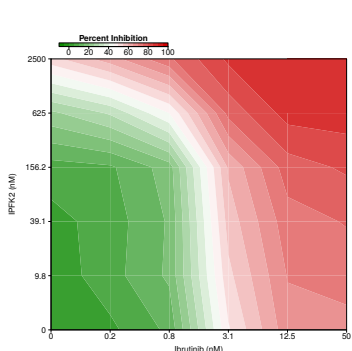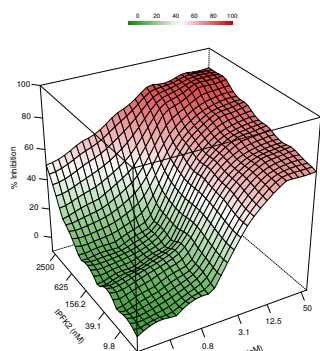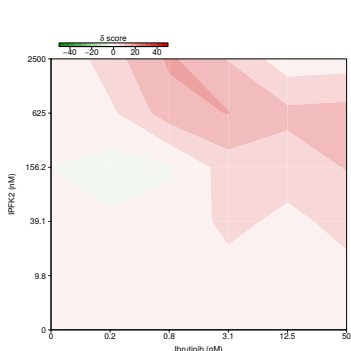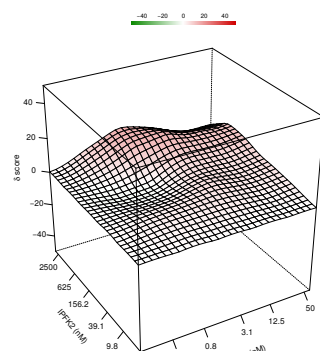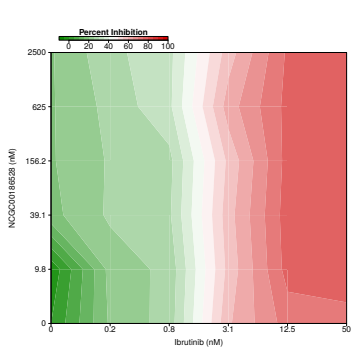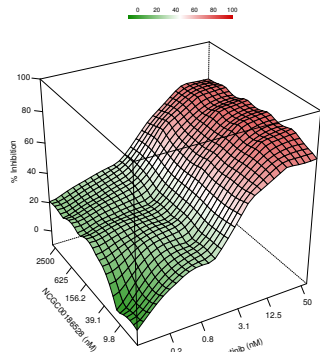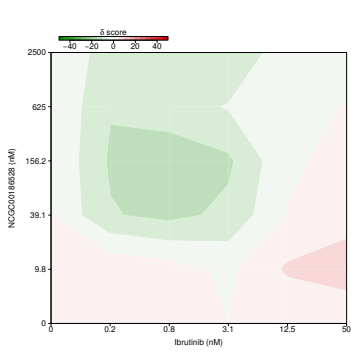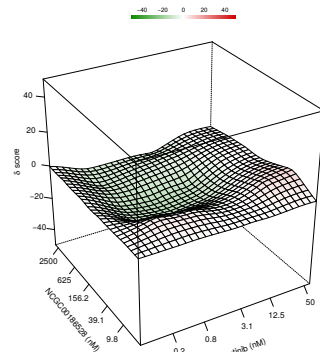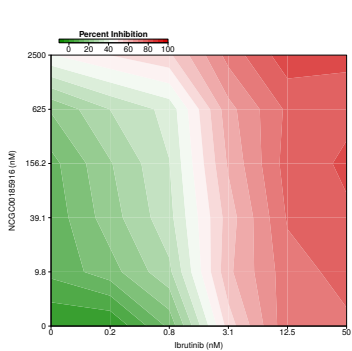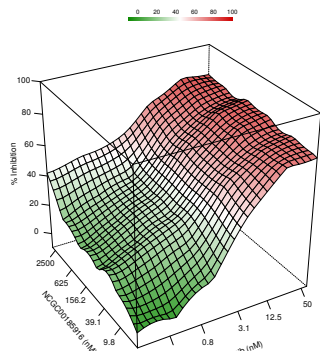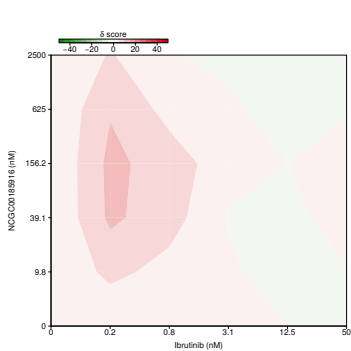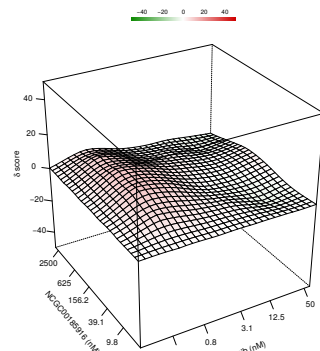

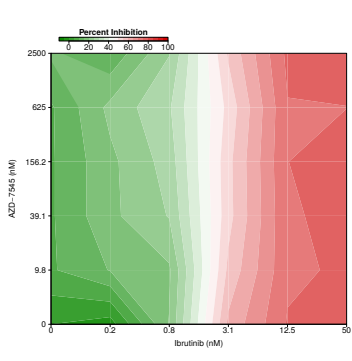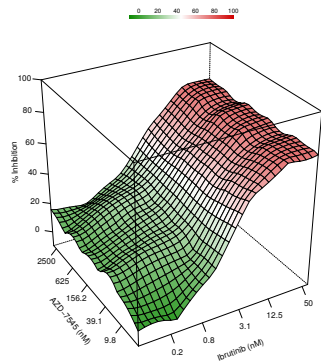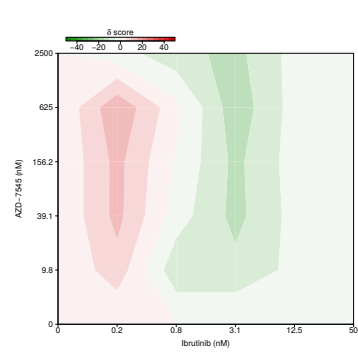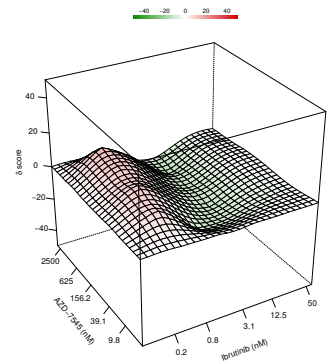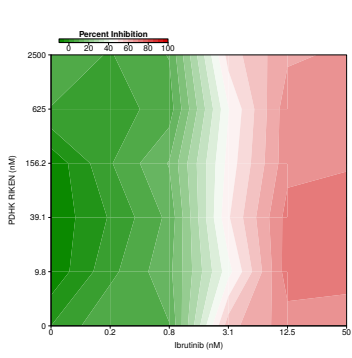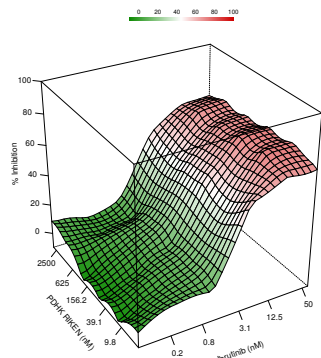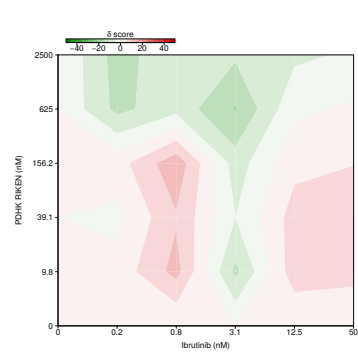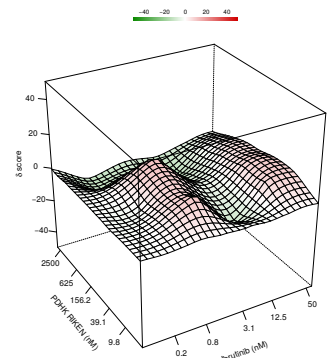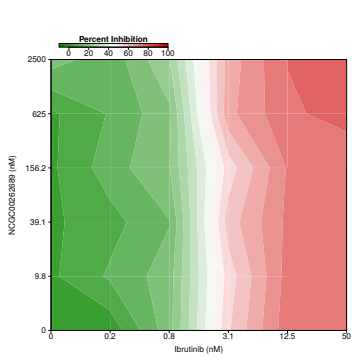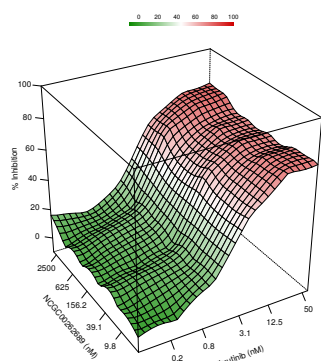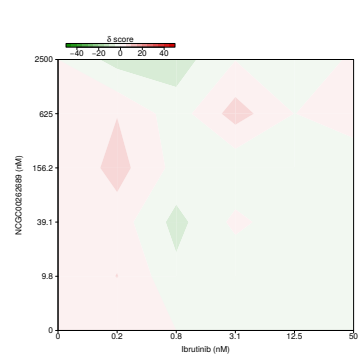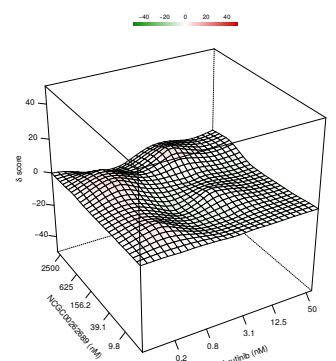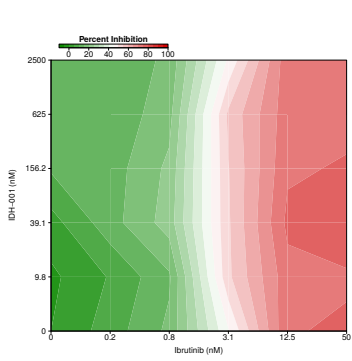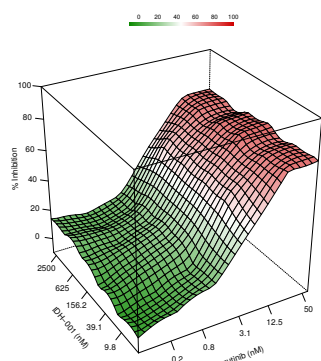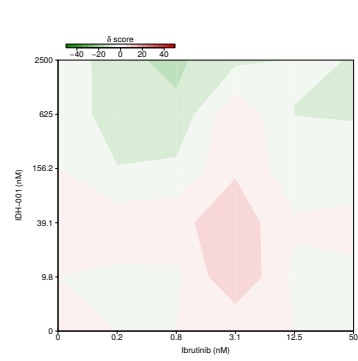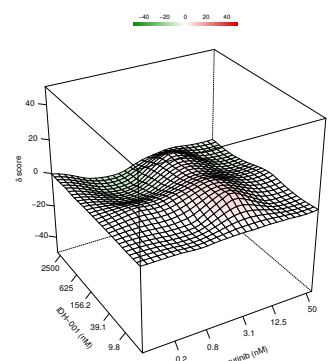

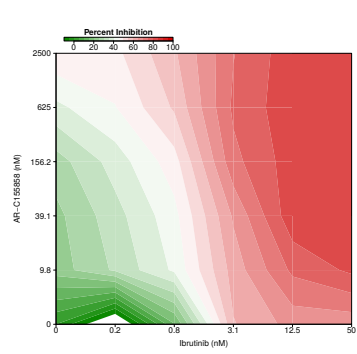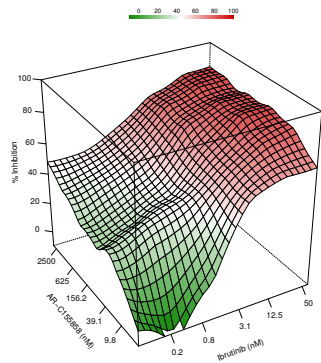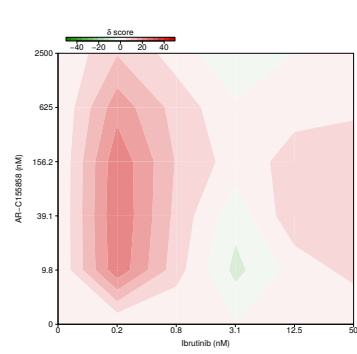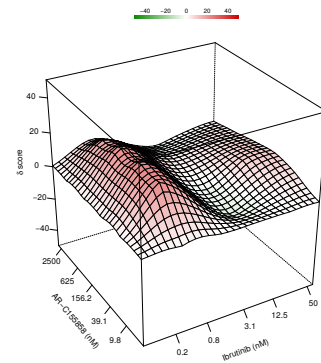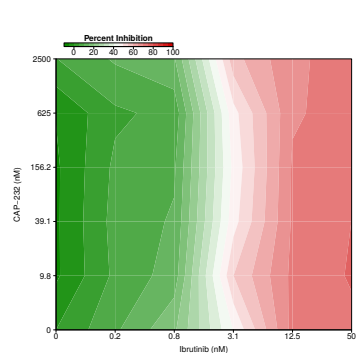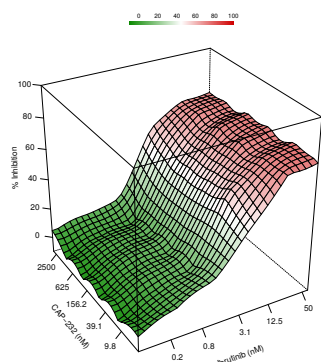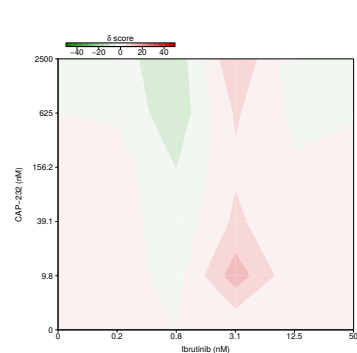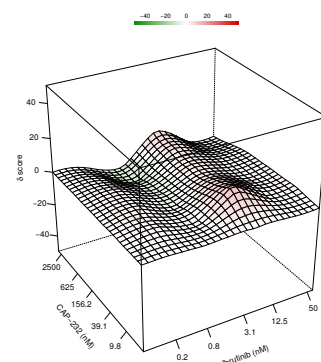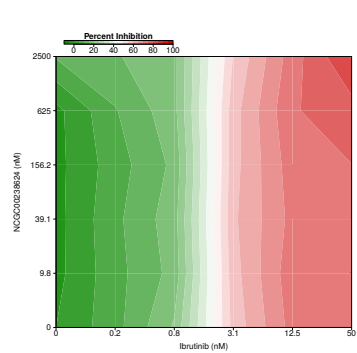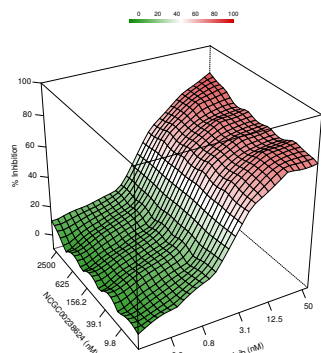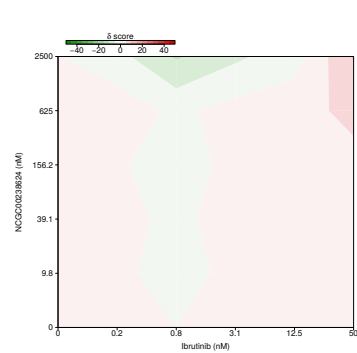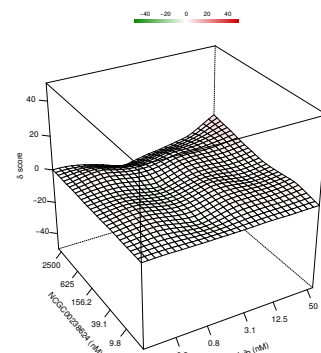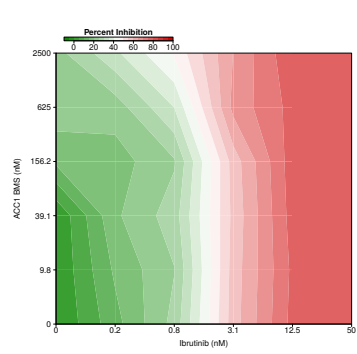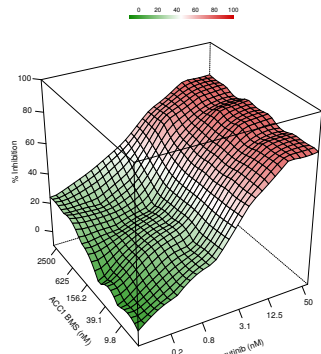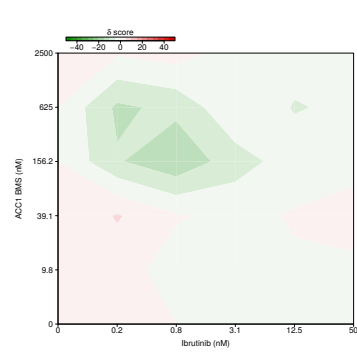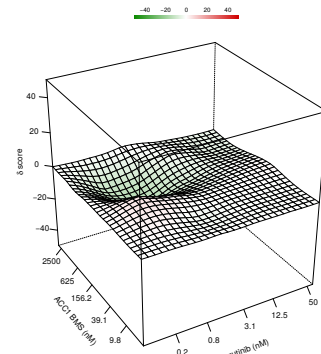

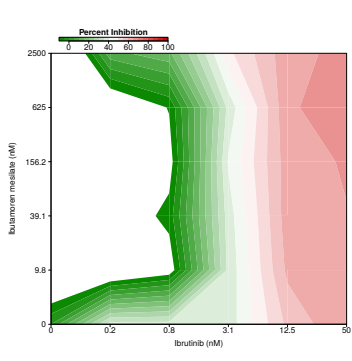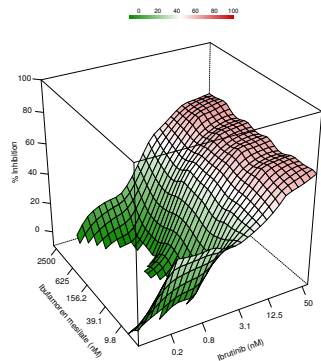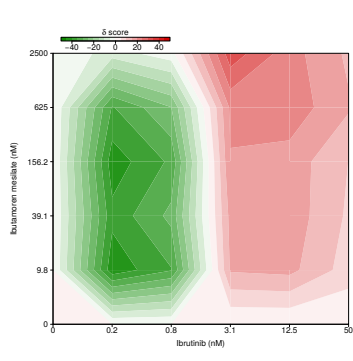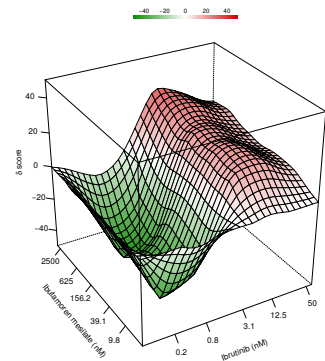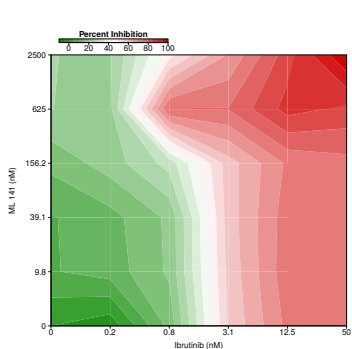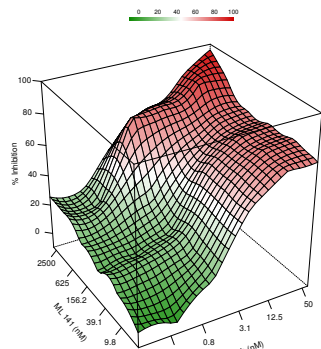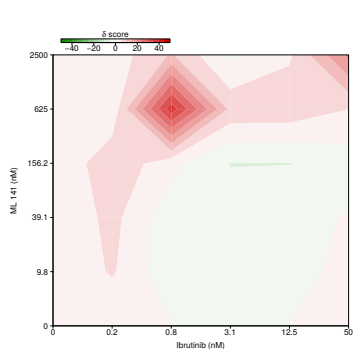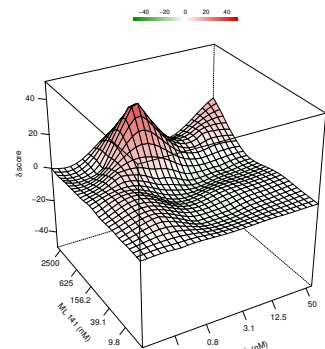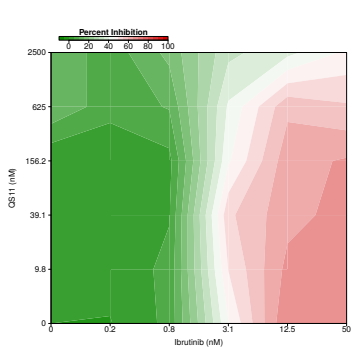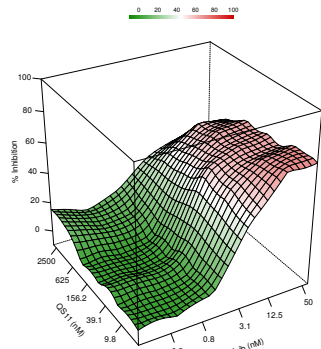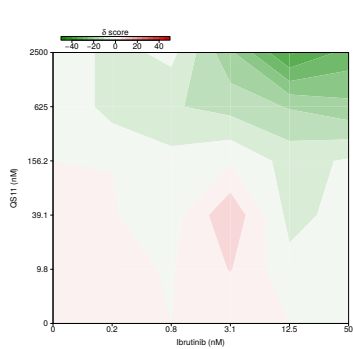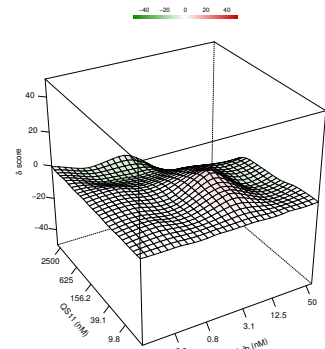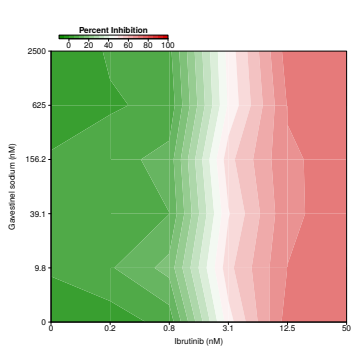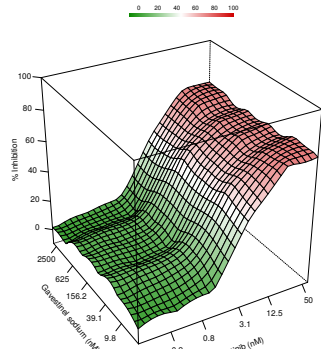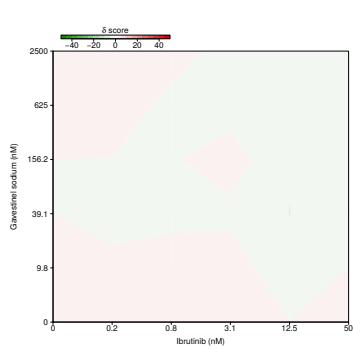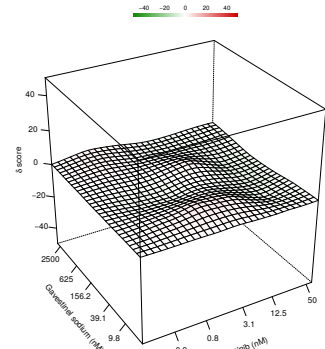



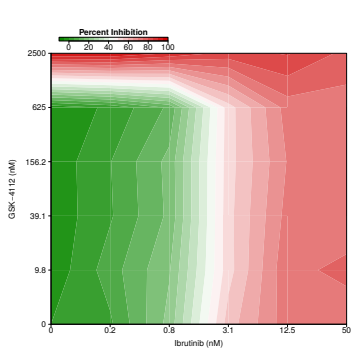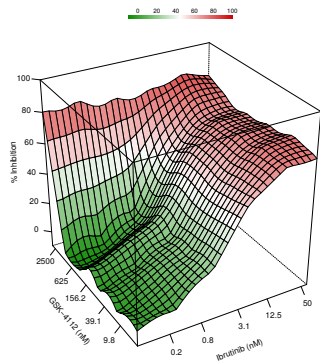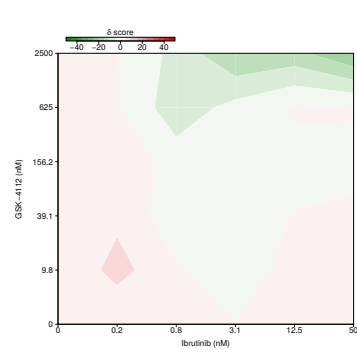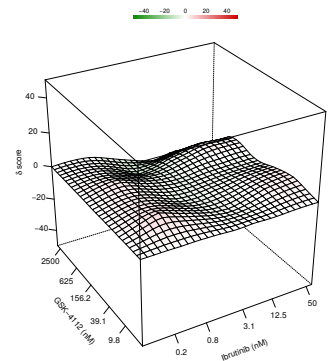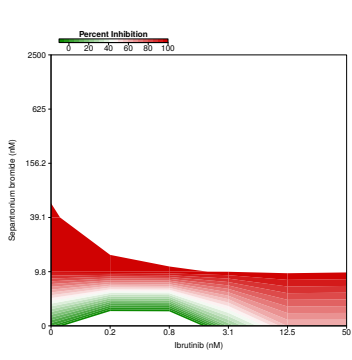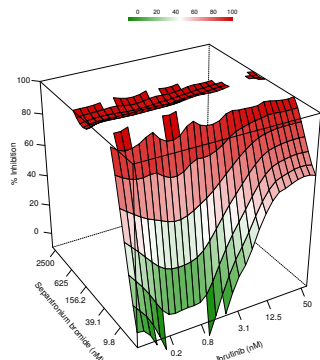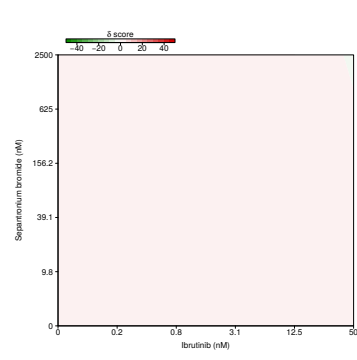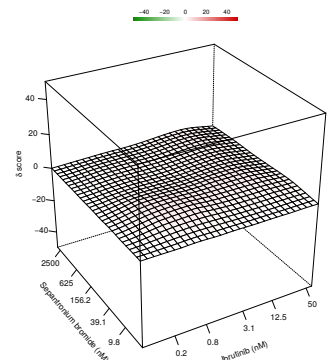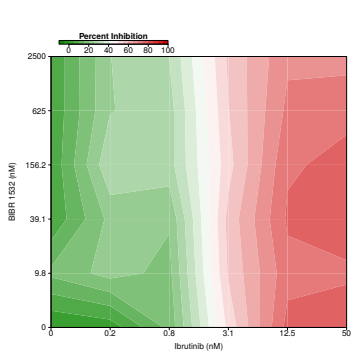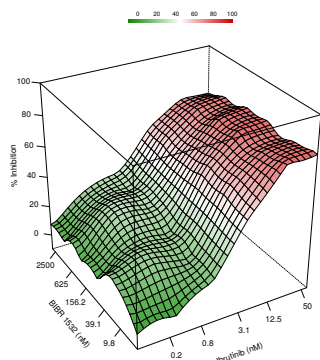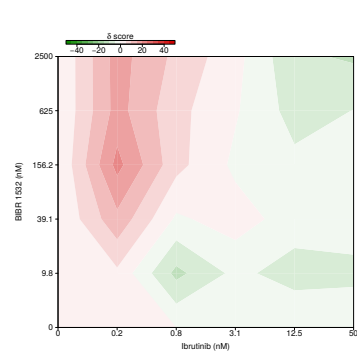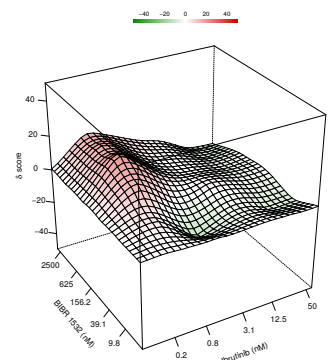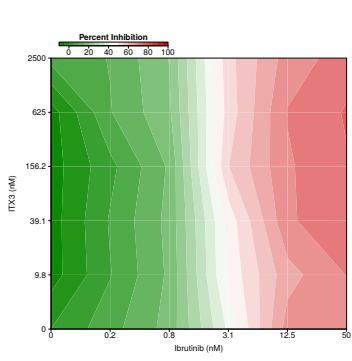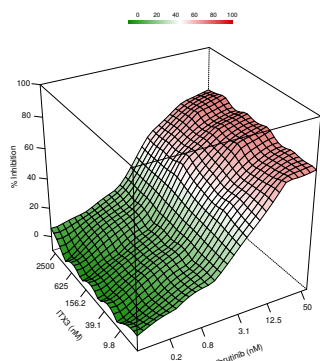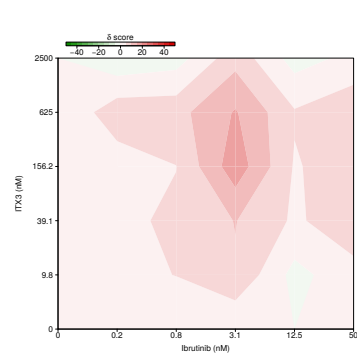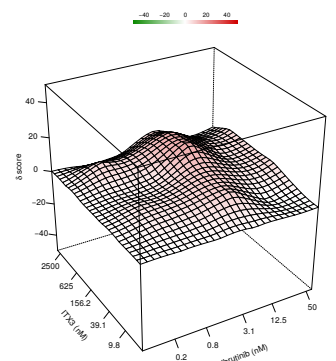



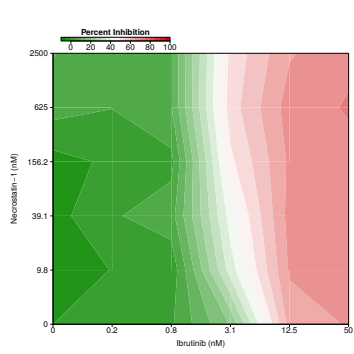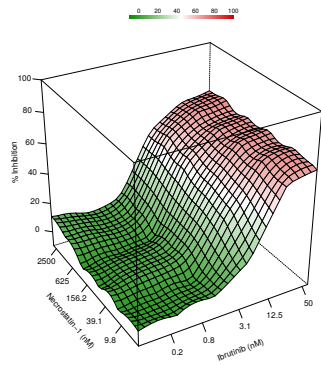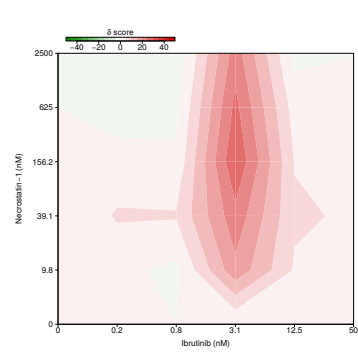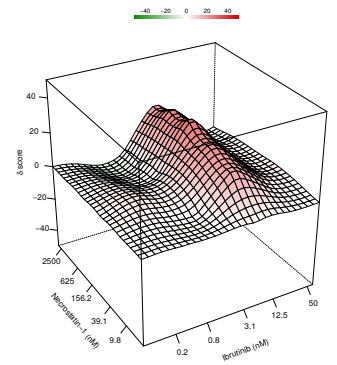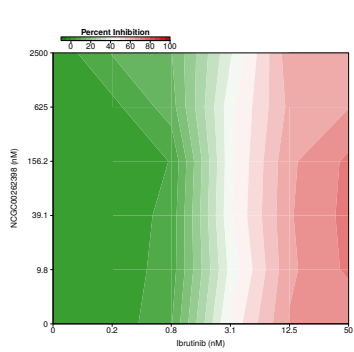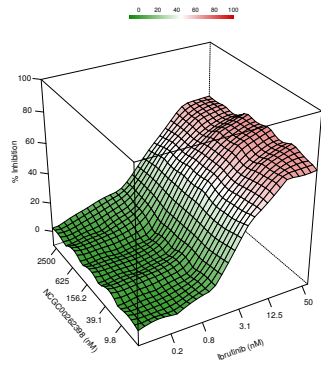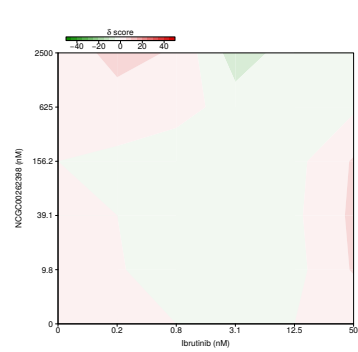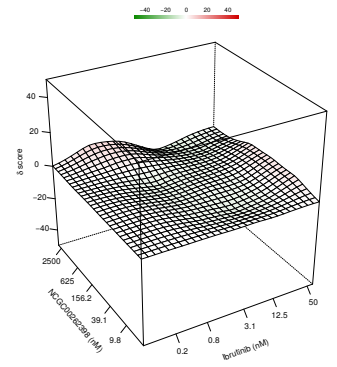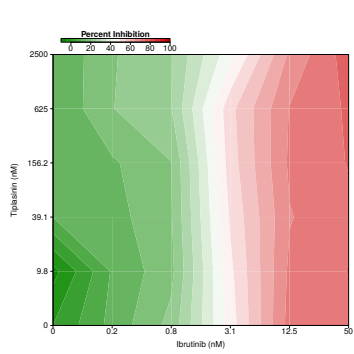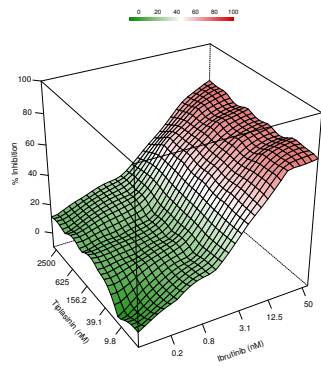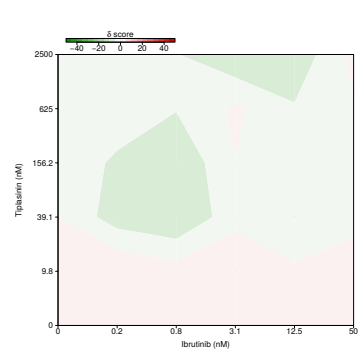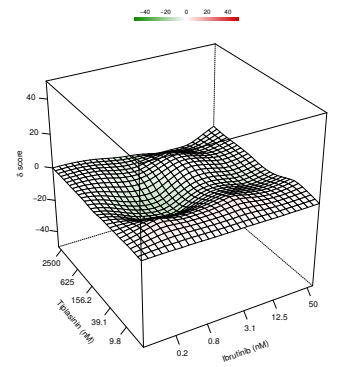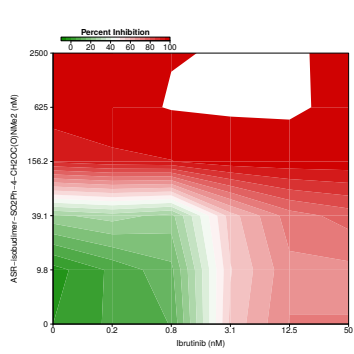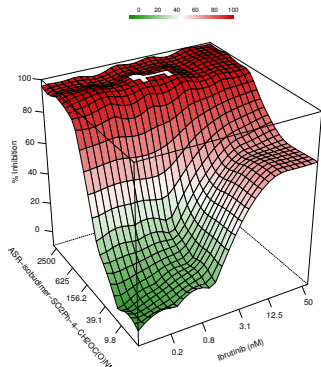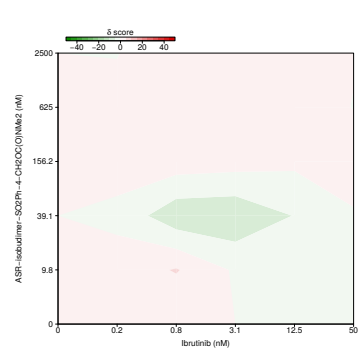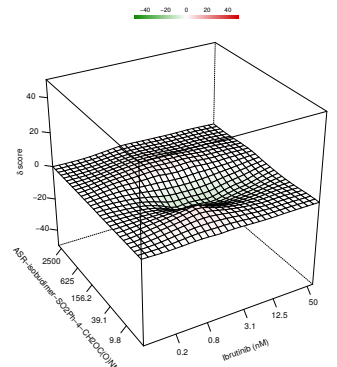

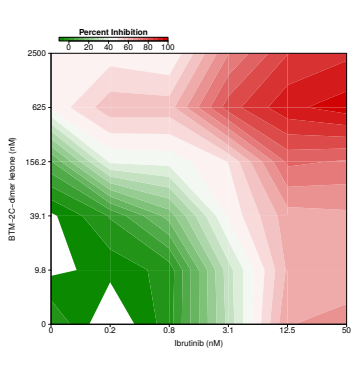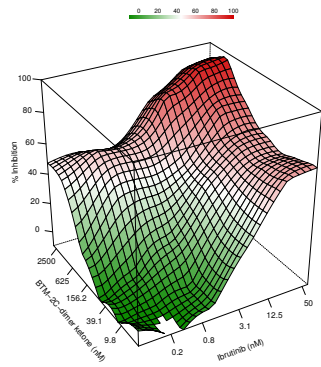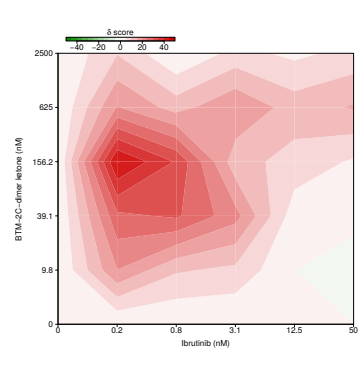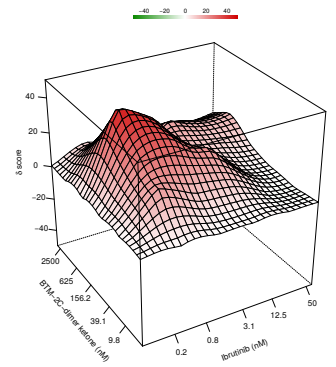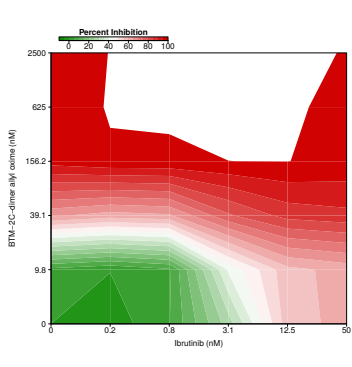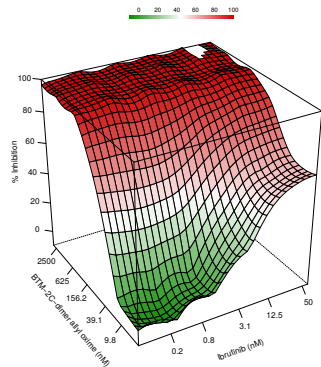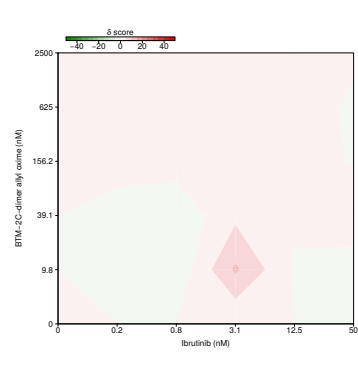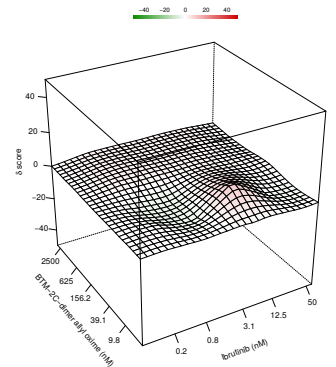

Supplement: Supplementary Fig. 3 — The interaction landscapes of the 466 drug combinations in the Mathews Griner data. [file mmc1.pdf]
