# Supplementary material for: Searching for Drug Synergy in Complex Dose–Response Landscapes Using an Interaction Potency Model
Source: Comput Struct Biotechnol J. 2015 Sep 25;13:504–13. doi: 10.1016/j.csbj.2015.09.001 (PMC4759128; doi:10.1016/j.csbj.2015.09.001)
Supplement: Supplementary Table 2 — The p-values for the AUROC differences between the interaction scores using the DeLong's test. [file mmc3.docx]

**Supplementary Table 2.** The p-values for the AUROC differences between the interaction scores using the DeLong's test.

|  | Delta | MedianExcess | NumExcess | ExcessHSA | ExcessCRX | LS3x3 | Beta | Gamma | CI |
| --- | --- | --- | --- | --- | --- | --- | --- | --- | --- |
| MedianExcess | 1.53E-25 |  |  |  |  |  |  |  |  |
| NumExcess | 0.0015 | 8.07E-22 |  |  |  |  |  |  |  |
| ExcessHSA | 0.0003 | 2.32E-21 | 0.4804 |  |  |  |  |  |  |
| ExcessCRX | 1.10E-08 | 3.07E-08 | 2.89E-06 | 7.06E-06 |  |  |  |  |  |
| LS3x3 | 0.0003 | 5.84E-21 | 0.1535 | 0.3454 | 5.51E-05 |  |  |  |  |
| Beta | 8.33E-05 | 8.54E-18 | 0.1068 | 0.1003 | 0.0002 | 0.4668 |  |  |  |
| Gamma | 0.0032 | 9.57E-21 | 0.8452 | 0.2501 | 3.42E-06 | 0.1846 | 0.0083 |  |  |
| CI | 1.73E-12 | 0.0004 | 5.80E-10 | 3.01E-10 | 0.0362 | 2.47E-09 | 2.15E-07 | 2.08E-10 |  |
| Alpha | 8.36E-10 | 0.0093 | 3.64E-07 | 1.17E-06 | 0.0717 | 5.15E-06 | 2.29E-05 | 3.19E-07 | 0.9265 |
