# Supplementary material for: Searching for Drug Synergy in Complex Dose–Response Landscapes Using an Interaction Potency Model
Source: Comput Struct Biotechnol J. 2015 Sep 25;13:504–13. doi: 10.1016/j.csbj.2015.09.001 (PMC4759128; doi:10.1016/j.csbj.2015.09.001)
Supplement: Supplementary File 1 — The R source code for calculating delta scores and the dynamic report including user instructions. [file mmc4.zip › Delta_calculation_dynamic_report.pdf]

Delta score calculation tutorial

Bhagwan Yadav

September 8, 2015

This is tutorial to compute delta score using R. First, we have to load required libraries in r programming

```
# Load libraries required
library(drc)
library(kriging)
library(reshape2)
library(compiler)
options(warn=-1)
```

Set the working directory to the folder where finction.R file is kept.

```
# Set working directory
setwd("/home/bhagwan/Dropbox/PhD_Projects/Project-5_VBS/Manuscript/r_codes")
```

Source the function file

```
# Source the functions file
source(paste(getwd(),"/functions.R",sep=""))
```

Read response data as well as metadata files

```
# Read response and metadata
response<-read.csv("/home/bhagwan/Dropbox/PhD_Projects/Project-5_VBS/data/Griner_et_al/241/responses.csv
metadata<-read.csv("/home/bhagwan/Dropbox/PhD_Projects/Project-5_VBS/data/Griner_et_al/241/metadata.csv"
```

Get the unique block IDs

```
# get unique bolck ids
blockId<-unique(response$BlockId)
```

Make matrix to store delta score

```
# Make matrix to store delta score
delta_score<-matrix(NA,length(blockId),4)
colnames(delta_score)<-c("blockId","Drug1","Drug2","Delta")
```

Compute delta score for each block store in above variable.

```
# Loop to compute delta for each combination
#set.seed(0)
suppressWarnings(
for(i in 1:length(blockId)){
```

```
        blockIndex<-which(response$BlockId %in% blockId[i])
        dataM<-response[blockIndex,c(2,3,4)]

################## Preparing Input Files start ######################
#  Making plate matrix file
        plate.file<-acast(dataM,Col~Row,value.var="Value")
        plate.file<-apply(t(apply(t(plate.file),2,rev)),2,rev)
        plate.mat <- 100-plate.file
        plate.mat<-apply(plate.mat,2,as.numeric)


# Mapping concentration range file
        meta.blockIndex<-which(metadata$BlockId %in% blockId[i])

        drug1name<-as.character(as.matrix(metadata$RowName[meta.blockIndex]))
        drug2name<-"Ibrutinib"
        colnames(plate.file)<-rep(drug1name,ncol(plate.file))
        rownames(plate.file)<-rep(drug2name,nrow(plate.file))

        drug1conc<-as.character(as.matrix(metadata$RowConcs[meta.blockIndex]))
        drug1conc<-rev(as.numeric(unlist(strsplit(drug1conc,","))))*2
        drug2conc<-as.character(as.matrix(metadata$ColConcs[meta.blockIndex]))
        drug2conc<-rev(as.numeric(unlist(strsplit(drug2conc,","))))*2

        conc.file<-rbind(drug1conc,drug2conc)
        conc.file<-cbind(rbind(drug1name,drug2name),conc.file)
        conc.range <-conc.file
        conc.range[1,2:ncol(conc.range)]<-as.character(round(
as.numeric(conc.range[1,2:ncol(conc.range)]),digits=1))
        conc.range[2,2:ncol(conc.range)]<-as.character(
round(as.numeric(conc.range[2,2:ncol(conc.range)]),digits=1))
# Add col names and row names to plate mat
        colnames(plate.mat)<-colnames(plate.file)
        rownames(plate.mat)<-rownames(plate.file)

# Making drug combination file
        pairs.file<-matrix(NA,1,5)
        pairs.file[1,]<-c(1,drug1name,drug2name,"cellline",1)
        pairs.file <- as.data.frame(pairs.file)
        pair.list <- pairs.file
        colnames(pair.list)<-c("index","drug1","drug2","cell.line","plate")

#################### Preparing Input Files End ######################

###################### Baseline correction ####################

        output_baseline= c.SDbaselineCor(plate.mat,conc.range,pair.list)

####################### Two Way Fitting ######################
# Single plate analysis

# raw_matrix=output_baseline[[1]] # raw matrix
        cor_matrix=output_baseline[[2]] # matrix after baseline correction
```

```r
        drug_pair=output_baseline[[3]] # drug names

        output = c.twowayfitting(cor_matrix,drug_pair)

        delta_score[i,1] = blockId[i]
        delta_score[i,2] = unique(colnames(plate.mat))
        delta_score[i,3] = unique(rownames(plate.mat))
        delta_score[i,4] = round(output,digits=4)

})
```

```r
# print delta score
print(delta_score)

##       blockId Drug1                                Drug2       Delta
##  [1,] "1"     "Bendamustine"                       "Ibrutinib" "-6.3468"
##  [2,] "2"     "Lonidamine"                         "Ibrutinib" "-1.9388"
##  [3,] "3"     "Lenalidomide"                       "Ibrutinib" "6.2456"
##  [4,] "4"     "Cladribine"                         "Ibrutinib" "4.0013"
##  [5,] "5"     "Pentostatin"                        "Ibrutinib" "-3.9636"
##  [6,] "6"     "6-mercaptopurine"                   "Ibrutinib" "-2.1205"
##  [7,] "7"     "Everolimus"                         "Ibrutinib" "11.1983"
##  [8,] "8"     "Aminoglutethimide"                  "Ibrutinib" "-1.9985"
##  [9,] "9"     "Formestane"                         "Ibrutinib" "-0.8742"
## [10,] "10"    "Letrozole"                          "Ibrutinib" "-0.2915"
## [11,] "11"    "Pemetrexed disodium"                "Ibrutinib" "-0.4784"
## [12,] "12"    "Gemcitabine"                        "Ibrutinib" "1.2103"
## [13,] "13"    "Tamoxifen"                          "Ibrutinib" "4.0054"
## [14,] "14"    "Vincristine sulfate"                "Ibrutinib" "4.8455"
## [15,] "15"    "Combretastatin A-4"                 "Ibrutinib" "0.4951"
## [16,] "16"    "Plinabulin"                         "Ibrutinib" "5.9503"
## [17,] "17"    "Docetaxel"                          "Ibrutinib" "14.4558"
## [18,] "18"    "Blebbistatin"                       "Ibrutinib" "2.8375"
## [19,] "19"    "Ispinesib"                          "Ibrutinib" "17.5966"
## [20,] "20"    "Doxorubicin"                        "Ibrutinib" "5.3546"
## [21,] "21"    "Topotecan hydrochloride"            "Ibrutinib" "6.7647"
## [22,] "22"    "Cyclophosphamide"                   "Ibrutinib" "-4.7014"
## [23,] "23"    "5-Azacitidine"                      "Ibrutinib" "-3.2309"
## [24,] "24"    "Actinomycin D"                      "Ibrutinib" "0.7481"
## [25,] "25"    "Mithramycin"                        "Ibrutinib" "4.1517"
## [26,] "26"    "Itraconazole"                       "Ibrutinib" "3.1795"
## [27,] "27"    "Hydroxychloroquine sulfate"         "Ibrutinib" "-0.9994"
## [28,] "28"    "Mifepristone"                       "Ibrutinib" "1.4239"
## [29,] "29"    "Metformin HCl"                      "Ibrutinib" "-2.2858"
## [30,] "30"    "Acadesine"                          "Ibrutinib" "2.9887"
## [31,] "31"    "A-769662"                           "Ibrutinib" "1.9776"
## [32,] "32"    "Imatinib"                           "Ibrutinib" "1.2214"
## [33,] "33"    "Nilotinib"                          "Ibrutinib" "6.2084"
## [34,] "34"    "Ponatinib"                          "Ibrutinib" "8.374"
## [35,] "35"    "DCC-2036"                           "Ibrutinib" "4.1032"
## [36,] "36"    "Dasatinib"                          "Ibrutinib" "3.1478"
## [37,] "37"    "Bosutinib"                          "Ibrutinib" "0.5349"
## [38,] "38"    "Saracatinib"                        "Ibrutinib" "5.4986"
```

```
## [39,] "39" "PD-166285"                        "Ibrutinib" "5.5027"
## [40,] "40" "Vandetanib"                       "Ibrutinib" "-2.7533"
## [41,] "41" "Erlotinib hydrochloride"          "Ibrutinib" "0.9148"
## [42,] "42" "Gefitinib"                        "Ibrutinib" "-0.3579"
## [43,] "43" "Lapatinib"                        "Ibrutinib" "0.2124"
## [44,] "44" "Neratinib"                        "Ibrutinib" "-2.0381"
## [45,] "45" "Canertinib"                       "Ibrutinib" "-14.0386"
## [46,] "46" "AV-412"                           "Ibrutinib" "-6.8827"
## [47,] "47" "Dacomitinib"                      "Ibrutinib" "-5.2818"
## [48,] "48" "CP-724714"                        "Ibrutinib" "-5.0341"
## [49,] "49" "Afatinib"                         "Ibrutinib" "-2.6713"
## [50,] "50" "AEE-788"                          "Ibrutinib" "-0.504"
## [51,] "51" "CUDC-101"                         "Ibrutinib" "3.268"
## [52,] "52" "Pelitinib"                        "Ibrutinib" "-6.0806"
## [53,] "53" "WZ-4002"                          "Ibrutinib" "-9.6184"
## [54,] "54" "Linifanib"                        "Ibrutinib" "-6.2503"
## [55,] "55" "Axitinib"                         "Ibrutinib" "-11.7996"
## [56,] "56" "Sorafenib"                        "Ibrutinib" "-0.2569"
## [57,] "57" "Vatalanib"                        "Ibrutinib" "-5.9609"
## [58,] "58" "Motesanib"                        "Ibrutinib" "-3.674"
## [59,] "59" "Tivozanib"                        "Ibrutinib" "-3.5885"
## [60,] "60" "Vargatef"                         "Ibrutinib" "6.94"
## [61,] "61" "Brivanib"                         "Ibrutinib" "1.0834"
## [62,] "62" "Telatinib"                        "Ibrutinib" "-5.7898"
## [63,] "63" "Cabozantinib"                     "Ibrutinib" "-3.16"
## [64,] "64" "Cediranib"                        "Ibrutinib" "1.9473"
## [65,] "65" "BMS-794833"                       "Ibrutinib" "-6.2111"
## [66,] "66" "Lenvatinib"                       "Ibrutinib" "7.0859"
## [67,] "67" "OSI-632"                          "Ibrutinib" "-5.42"
## [68,] "68" "Foretinib"                        "Ibrutinib" "3.4648"
## [69,] "69" "Regorafenib"                      "Ibrutinib" "-1.9613"
## [70,] "70" "Dovitinib"                        "Ibrutinib" "-0.4967"
## [71,] "71" "Masitinib"                        "Ibrutinib" "3.1735"
## [72,] "72" "PD-173074"                        "Ibrutinib" "-3.1279"
## [73,] "73" "SG-00529"                         "Ibrutinib" "5.5659"
## [74,] "74" "Amuvatinib"                       "Ibrutinib" "-0.9104"
## [75,] "75" "OSI-930"                          "Ibrutinib" "4.1966"
## [76,] "76" "Crizotinib"                       "Ibrutinib" "3.8533"
## [77,] "77" "PHA-665752"                       "Ibrutinib" "0.9738"
## [78,] "78" "BMS-777607"                       "Ibrutinib" "0.671"
## [79,] "79" "PF-04217903"                      "Ibrutinib" "-1.3699"
## [80,] "80" "JNJ-38877605"                     "Ibrutinib" "0.0382"
## [81,] "81" "SGX-523"                          "Ibrutinib" "4.1568"
## [82,] "82" "AMG-51"                           "Ibrutinib" "0.428"
## [83,] "83" "Podofilox"                        "Ibrutinib" "3.8533"
## [84,] "84" "Linsitinib"                       "Ibrutinib" "1.622"
## [85,] "85" "GSK-1904529A"                     "Ibrutinib" "-6.2493"
## [86,] "86" "Dorsomorphin"                     "Ibrutinib" "-1.1126"
## [87,] "87" "LY2157299"                        "Ibrutinib" "-1.0503"
## [88,] "88" "LY2109761"                        "Ibrutinib" "-2.5351"
## [89,] "89" "A 83-01"                          "Ibrutinib" "-0.2188"
## [90,] "90" "SJN 2511"                         "Ibrutinib" "-0.3455"
## [91,] "91" "SD-208"                           "Ibrutinib" "3.3713"
```

```
##  [92,] "92"    "TAE-684"                           "Ibrutinib" "1.1458"
##  [93,] "93"    "SB-525334"                         "Ibrutinib" "-1.9296"
##  [94,] "94"    "SB-431542"                         "Ibrutinib" "-3.8038"
##  [95,] "95"    "Tandutinib"                        "Ibrutinib" "-5.8832"
##  [96,] "96"    "Quizartinib"                       "Ibrutinib" "0.3518"
##  [97,] "97"    "AZ-23"                             "Ibrutinib" "3.4151"
##  [98,] "98"    "LDN-193189"                        "Ibrutinib" "-3.3286"
##  [99,] "99"    "PRT-060318"                        "Ibrutinib" "2.6408"
## [100,] "100"   "R406"                              "Ibrutinib" "2.9619"
## [101,] "101"   "Perifosine"                        "Ibrutinib" "4.0698"
## [102,] "102"   "GDC-0941"                          "Ibrutinib" "2.8008"
## [103,] "103"   "GSK-2126458"                       "Ibrutinib" "1.9257"
## [104,] "104"   "IC-87114"                          "Ibrutinib" "7.3593"
## [105,] "105"   "PIK-90"                            "Ibrutinib" "6.4427"
## [106,] "106"   "BKM-120"                           "Ibrutinib" "0.9764"
## [107,] "107"   "CAL-101"                           "Ibrutinib" "10.8295"
## [108,] "108"   "BAG-956"                           "Ibrutinib" "5.4527"
## [109,] "109"   "AZD-6482"                          "Ibrutinib" "4.7657"
## [110,] "110"   "CAY10626"                          "Ibrutinib" "5.8777"
## [111,] "111"   "PI-103"                            "Ibrutinib" "3.9729"
## [112,] "112"   "17_-hydroxy Wortmannin"            "Ibrutinib" "10.3078"
## [113,] "113"   "Deguelin"                          "Ibrutinib" "4.0447"
## [114,] "114"   "MK-2206"                           "Ibrutinib" "11.2472"
## [115,] "115"   "A-674563"                          "Ibrutinib" "-0.1616"
## [116,] "116"   "GSK-690693"                        "Ibrutinib" "3.9824"
## [117,] "117"   "BEZ-235"                           "Ibrutinib" "6.6094"
## [118,] "118"   "PF-05212384"                       "Ibrutinib" "6.6506"
## [119,] "119"   "GDC-0980"                          "Ibrutinib" "4.1276"
## [120,] "120"   "Torin-1"                           "Ibrutinib" "6.072"
## [121,] "121"   "Torin-2"                           "Ibrutinib" "8.3212"
## [122,] "122"   "AZD-8055"                          "Ibrutinib" "3.4301"
## [123,] "123"   "OSI-027"                           "Ibrutinib" "3.2355"
## [124,] "124"   "KU-0063794"                        "Ibrutinib" "-0.2222"
## [125,] "125"   "WYE-354"                           "Ibrutinib" "3.3161"
## [126,] "126"   "Salirasib"                         "Ibrutinib" "3.6518"
## [127,] "127"   "NU-7441"                           "Ibrutinib" "7.3682"
## [128,] "128"   "KU 0060648"                        "Ibrutinib" "5.9441"
## [129,] "129"   "NSC 23766"                         "Ibrutinib" "0.5056"
## [130,] "130"   "NCGC00188866-01"                   "Ibrutinib" "1.3509"
## [131,] "131"   "VX-702"                            "Ibrutinib" "1.526"
## [132,] "132"   "SB-203580"                         "Ibrutinib" "0.9049"
## [133,] "133"   "VX-745"                            "Ibrutinib" "2.2659"
## [134,] "134"   "Doramapimod"                       "Ibrutinib" "4.3496"
## [135,] "135"   "PH-797804"                         "Ibrutinib" "-0.1161"
## [136,] "136"   "RWJ-67657"                         "Ibrutinib" "0.9253"
## [137,] "137"   "Talmapimod"                        "Ibrutinib" "3.541"
## [138,] "138"   "FMK"                               "Ibrutinib" "5.3827"
## [139,] "139"   "BI-D1870"                          "Ibrutinib" "4.2654"
## [140,] "140"   "NCGC00253463-01"                   "Ibrutinib" "5.8137"
## [141,] "141"   "PHA-408"                           "Ibrutinib" "2.1371"
## [142,] "142"   "Withaferin A"                      "Ibrutinib" "4.6917"
## [143,] "143"   "BX-795"                            "Ibrutinib" "9.8416"
## [144,] "144"   "Bardoxolone methyl"                "Ibrutinib" "6.6851"
```

```
## [145,] "145"   "IKK16"                                  "Ibrutinib" "4.0977"
## [146,] "146"   "PF-184"                                 "Ibrutinib" "0.6804"
## [147,] "147"   "IMD-0354"                               "Ibrutinib" "-0.0515"
## [148,] "148"   "PS-1145"                                "Ibrutinib" "1.0353"
## [149,] "149"   "MLN-120B"                               "Ibrutinib" "2.3123"
## [150,] "150"   "cycloheximide"                          "Ibrutinib" "-0.0464"
## [151,] "151"   "CT-99021"                               "Ibrutinib" "-6.72"
## [152,] "152"   "SB-216763"                              "Ibrutinib" "-12.6769"
## [153,] "153"   "NCGC00161703"                           "Ibrutinib" "10.4519"
## [154,] "154"   "WAY-204688"                             "Ibrutinib" "3.3684"
## [155,] "155"   "Sulfasalazine"                          "Ibrutinib" "-0.883"
## [156,] "156"   "NCGC00263020-01"                        "Ibrutinib" "4.3433"
## [157,] "157"   "BX-795"                                 "Ibrutinib" "-2.2567"
## [158,] "158"   "KU-60019"                               "Ibrutinib" "1.2964"
## [159,] "159"   "KU-0064"                                "Ibrutinib" "3.5376"
## [160,] "160"   "CP-466722"                              "Ibrutinib" "0.4825"
## [161,] "161"   "SM-164"                                 "Ibrutinib" "-7.164"
## [162,] "162"   "TW-37"                                  "Ibrutinib" "-0.3849"
## [163,] "163"   "(-)-Gossypol"                           "Ibrutinib" "-0.7108"
## [164,] "164"   "Chelerythrine chloride"                 "Ibrutinib" "3.174"
## [165,] "165"   "Obatoclax"                              "Ibrutinib" "-3.3754"
## [166,] "166"   "Navitoclax"                             "Ibrutinib" "13.2502"
## [167,] "167"   "Nutlin-3"                               "Ibrutinib" "6.466"
## [168,] "168"   "Ac-SAH-p53-8"                           "Ibrutinib" "-0.2576"
## [169,] "169"   "Serdemetan"                             "Ibrutinib" "3.6526"
## [170,] "170"   "HLI-373989"                             "Ibrutinib" "-3.4475"
## [171,] "171"   "SJ-172550"                              "Ibrutinib" "2.3218"
## [172,] "172"   "AZD-7762"                               "Ibrutinib" "6.9659"
## [173,] "173"   "PF-477736"                              "Ibrutinib" "6.4006"
## [174,] "174"   "Flavopiridol???"                        "Ibrutinib" "3.8065"
## [175,] "175"   "Purvalanol B"                           "Ibrutinib" "-1.7598"
## [176,] "176"   "Seliciclib"                             "Ibrutinib" "2.809"
## [177,] "177"   "PHA-690509"                             "Ibrutinib" "6.6421"
## [178,] "178"   "AT7519"                                 "Ibrutinib" "2.1474"
## [179,] "179"   "AT7519"                                 "Ibrutinib" "1.4255"
## [180,] "180"   "SNS-032"                                "Ibrutinib" "2.362"
## [181,] "181"   "PHA-793887"                             "Ibrutinib" "2.2839"
## [182,] "182"   "PD-0332991"                             "Ibrutinib" "8.2939"
## [183,] "183"   "DA-3003-1"                              "Ibrutinib" "0.396"
## [184,] "184"   "JNJ-7706621"                            "Ibrutinib" "-0.7994"
## [185,] "185"   "SNS-314"                                "Ibrutinib" "2.6661"
## [186,] "186"   "Tozasertib"                             "Ibrutinib" "3.9173"
## [187,] "187"   "Alisertib"                              "Ibrutinib" "1.7995"
## [188,] "188"   "Danusertib"                             "Ibrutinib" "1.6745"
## [189,] "189"   "CYC-116"                                "Ibrutinib" "0.3857"
## [190,] "190"   "AZD-1152-HQPA"                          "Ibrutinib" "1.7661"
## [191,] "191"   "AMG-900"                                "Ibrutinib" "0.1407"
## [192,] "192"   "ENMD-981693"                            "Ibrutinib" "0.6939"
## [193,] "193"   "CHIR-265"                               "Ibrutinib" "2.9094"
## [194,] "194"   "AZ-628"                                 "Ibrutinib" "-0.3608"
## [195,] "195"   "Vemurafenib"                            "Ibrutinib" "-1.7404"
## [196,] "196"   "PLX-4720"                               "Ibrutinib" "0.8676"
## [197,] "197"   "AR-00341677"                            "Ibrutinib" "-8.6081"
```

```
## [198,] "198"   "RDEA-119"                                     "Ibrutinib" "-3.7577"
## [199,] "199"   "Selumetinib"                                  "Ibrutinib" "2.0339"
## [200,] "200"   "PD-0325901"                                   "Ibrutinib" "6.848"
## [201,] "201"   "Trametinib"                                   "Ibrutinib" "8.5621"
## [202,] "202"   "TAK-733"                                      "Ibrutinib" "1.3587"
## [203,] "203"   "XMD8-92"                                      "Ibrutinib" "-1.2673"
## [204,] "204"   "NCGC00242487"                                 "Ibrutinib" "-5.7912"
## [205,] "205"   "S6K-18"                                       "Ibrutinib" "-2.3854"
## [206,] "206"   "PF-4708671"                                   "Ibrutinib" "3.5897"
## [207,] "207"   "PF-431396"                                    "Ibrutinib" "-0.8793"
## [208,] "208"   "PF-573228"                                    "Ibrutinib" "6.0946"
## [209,] "209"   "PCI-32765"                                    "Ibrutinib" "-3.3197"
## [210,] "210"   "NCGC00188382-01"                              "Ibrutinib" "7.2905"
## [211,] "211"   "BMS-509744"                                   "Ibrutinib" "4.4802"
## [212,] "212"   "Midostaurin"                                  "Ibrutinib" "2.6752"
## [213,] "213"   "Enzastaurin"                                  "Ibrutinib" "2.0163"
## [214,] "214"   "Sotrastaurin"                                 "Ibrutinib" "-2.4207"
## [215,] "215"   "Ruboxistaurin mesilate"                       "Ibrutinib" "6.2243"
## [216,] "216"   "NCGC00263018-01"                              "Ibrutinib" "-1.7551"
## [217,] "217"   "GW 843682X"                                   "Ibrutinib" "-0.1172"
## [218,] "218"   "BI-2536"                                      "Ibrutinib" "8.3663"
## [219,] "219"   "Volasertib"                                   "Ibrutinib" "4.3044"
## [220,] "220"   "GSK-461364A"                                  "Ibrutinib" "12.8113"
## [221,] "221"   "KN-93"                                        "Ibrutinib" "2.4081"
## [222,] "222"   "KN-62"                                        "Ibrutinib" "-3.8864"
## [223,] "223"   "GSK-269962A"                                  "Ibrutinib" "2.2392"
## [224,] "224"   "Y-27632"                                      "Ibrutinib" "-5.0092"
## [225,] "225"   "GSK-650394"                                   "Ibrutinib" "2.768"
## [226,] "226"   "AZD-1480"                                     "Ibrutinib" "3.9775"
## [227,] "227"   "Tofacitinib"                                  "Ibrutinib" "-3.3457"
## [228,] "228"   "Ruxolitinib"                                  "Ibrutinib" "-1.2363"
## [229,] "229"   "CYT387"                                       "Ibrutinib" "0.5113"
## [230,] "230"   "Lestaurtinib???"                              "Ibrutinib" "8.3604"
## [231,] "231"   "SB1518"                                       "Ibrutinib" "7.0246"
## [232,] "232"   "Leflunomide"                                  "Ibrutinib" "-4.9537"
## [233,] "233"   "NVP-BSK805"                                   "Ibrutinib" "1.8655"
## [234,] "234"   "Degrasyn"                                     "Ibrutinib" "2.0821"
## [235,] "235"   "RO495"                                        "Ibrutinib" "2.1487"
## [236,] "236"   "NCGC00244250-01"                              "Ibrutinib" "5.9113"
## [237,] "237"   "NCGC00244250-01"                              "Ibrutinib" "1.5557"
## [238,] "238"   "ICG-001"                                      "Ibrutinib" "3.1328"
## [239,] "239"   "Wnt-C59"                                      "Ibrutinib" "-5.9843"
## [240,] "240"   "D-4476"                                       "Ibrutinib" "-4.369"
## [241,] "241"   "Silmitasertib"                                "Ibrutinib" "9.2125"
## [242,] "242"   "NVP-LDE-225"                                  "Ibrutinib" "-3.2522"
## [243,] "243"   "Cyclopamine"                                  "Ibrutinib" "0.3075"
## [244,] "244"   "LY-2940680??"                                 "Ibrutinib" "-1.5425"
## [245,] "245"   "SANT-2"                                       "Ibrutinib" "3.7299"
## [246,] "246"   "Hh-Ag1.5"                                     "Ibrutinib" "4.9584"
## [247,] "247"   "Vismodegib"                                   "Ibrutinib" "-2.5658"
## [248,] "248"   "RO-4929097"                                   "Ibrutinib" "-3.9081"
## [249,] "249"   "MK-0752"                                      "Ibrutinib" "0.6312"
## [250,] "250"   "YO-01027"                                     "Ibrutinib" "-1.466"
```

```
## [251,] "251"   "GSI-9"                           "Ibrutinib" "0.1532"
## [252,] "252"   "NVP-XAV-939"                     "Ibrutinib" "-3.6868"
## [253,] "253"   "NCGC00263015-01"                 "Ibrutinib" "1.407"
## [254,] "254"   "CPG-52364"                       "Ibrutinib" "0.3165"
## [255,] "255"   "IRAK-1-4 Inhibitor I"            "Ibrutinib" "-6.1298"
## [256,] "256"   "NCGC00241410-01"                 "Ibrutinib" "-4.037"
## [257,] "257"   "NCGC00241411-01"                 "Ibrutinib" "-4.7634"
## [258,] "258"   "BI-78D3"                         "Ibrutinib" "-1.1909"
## [259,] "259"   "SR-3306"                         "Ibrutinib" "3.8395"
## [260,] "260"   "STF-083010"                      "Ibrutinib" "-0.0302"
## [261,] "261"   "Irestatin 9389"                  "Ibrutinib" "0.854"
## [262,] "262"   "SB-265610"                       "Ibrutinib" "-0.1963"
## [263,] "263"   "Apilimod"                        "Ibrutinib" "-2.4141"
## [264,] "264"   "PD-0220245"                      "Ibrutinib" "3.589"
## [265,] "265"   "KRP 203"                         "Ibrutinib" "0.9584"
## [266,] "266"   "AMG-47a"                         "Ibrutinib" "2.5703"
## [267,] "267"   "Pim 1 inhibitor 2"               "Ibrutinib" "-6.9234"
## [268,] "268"   "SGI-1776"                        "Ibrutinib" "2.9402"
## [269,] "269"   "KI-20227"                        "Ibrutinib" "0.8618"
## [270,] "270"   "ABT-702"                         "Ibrutinib" "-6.545"
## [271,] "271"   "AZ-3146"                         "Ibrutinib" "4.5631"
## [272,] "272"   "MK-1775"                         "Ibrutinib" "2.8493"
## [273,] "273"   "BMS-5"                           "Ibrutinib" "-2.4721"
## [274,] "274"   "AMG-Tie2-1"                      "Ibrutinib" "4.9118"
## [275,] "275"   "NVP231"                          "Ibrutinib" "3.1704"
## [276,] "276"   "D-NMAPPD"                        "Ibrutinib" "4.2715"
## [277,] "277"   "Pazopanib"                       "Ibrutinib" "-0.8906"
## [278,] "278"   "TG-101348"                       "Ibrutinib" "1.7072"
## [279,] "279"   "Alvespimycin hydrochloride"      "Ibrutinib" "2.0508"
## [280,] "280"   "CCT-018159"                      "Ibrutinib" "-4.962"
## [281,] "281"   "CNF-2024"                        "Ibrutinib" "2.0949"
## [282,] "282"   "NVP-AUY922"                      "Ibrutinib" "2.8594"
## [283,] "283"   "Elesclomol"                      "Ibrutinib" "8.6765"
## [284,] "284"   "VER-155008"                      "Ibrutinib" "-0.66"
## [285,] "285"   "ISOX"                            "Ibrutinib" "6.0767"
## [286,] "286"   "Vorinostat"                      "Ibrutinib" "1.4428"
## [287,] "287"   "AR-42"                           "Ibrutinib" "3.779"
## [288,] "288"   "Belinostat"                      "Ibrutinib" "8.2978"
## [289,] "289"   "Mocetinostat"                    "Ibrutinib" "-3.5319"
## [290,] "290"   "Panobinostat"                    "Ibrutinib" "3.2171"
## [291,] "291"   "Pracinostat"                     "Ibrutinib" "2.5123"
## [292,] "292"   "Romidepsin"                      "Ibrutinib" "0.9377"
## [293,] "293"   "SRT1720"                         "Ibrutinib" "-0.2566"
## [294,] "294"   "EX-527"                          "Ibrutinib" "0.577"
## [295,] "295"   "Entinostat"                      "Ibrutinib" "-1.1256"
## [296,] "296"   "MG-149"                          "Ibrutinib" "-2.413"
## [297,] "297"   "Veliparib"                       "Ibrutinib" "-3.4708"
## [298,] "298"   "Olaparib"                        "Ibrutinib" "-2.0275"
## [299,] "299"   "AG-14361"                        "Ibrutinib" "-2.3848"
## [300,] "300"   "Rucaparib"                       "Ibrutinib" "-0.7533"
## [301,] "301"   "Iniparib"                        "Ibrutinib" "2.3941"
## [302,] "302"   "MLN-2238??"                      "Ibrutinib" "-1.792"
## [303,] "303"   "Carfilzomib"                     "Ibrutinib" "5.2159"
```

```
## [304,] "304"   "Bortezomib"                         "Ibrutinib" "0.972"
## [305,] "305"   "Tosedostat"                         "Ibrutinib" "-3.7345"
## [306,] "306"   "Teriflunomide"                      "Ibrutinib" "0.0655"
## [307,] "307"   "PD-166793"                          "Ibrutinib" "-5.3921"
## [308,] "308"   "SSR-69071"                          "Ibrutinib" "-0.2588"
## [309,] "309"   "RG-108"                             "Ibrutinib" "-1.2989"
## [310,] "310"   "BIX-01294"                          "Ibrutinib" "0.2473"
## [311,] "311"   "JQ1"                                "Ibrutinib" "6.1719"
## [312,] "312"   "NCGC00185090-04"                    "Ibrutinib" "-1.9086"
## [313,] "313"   "NCGC00189393-02"                    "Ibrutinib" "0.3327"
## [314,] "314"   "NCGC00247866-02"                    "Ibrutinib" "2.1733"
## [315,] "315"   "NCGC00241036-01"                    "Ibrutinib" "-0.875"
## [316,] "316"   "NCGC00183808-01"                    "Ibrutinib" "0.7783"
## [317,] "317"   "Tipifarnib"                         "Ibrutinib" "9.9156"
## [318,] "318"   "LG100268"                           "Ibrutinib" "-1.6015"
## [319,] "319"   "AHPN"                               "Ibrutinib" "0.1237"
## [320,] "320"   "AC-261066"                          "Ibrutinib" "-4.7539"
## [321,] "321"   "Fenretinide"                        "Ibrutinib" "6.0443"
## [322,] "322"   "Hydrocortisone"                     "Ibrutinib" "10.2627"
## [323,] "323"   "Dexamethasone"                      "Ibrutinib" "11.5539"
## [324,] "324"   "Cortivazol"                         "Ibrutinib" "11.1809"
## [325,] "325"   "Deacetyl cortivazol"                "Ibrutinib" "7.8513"
## [326,] "326"   "Abiraterone"                        "Ibrutinib" "8.0934"
## [327,] "327"   "2-Methoxyestradiol"                 "Ibrutinib" "2.4975"
## [328,] "328"   "Quercetine"                         "Ibrutinib" "-2.7979"
## [329,] "329"   "Doxercalciferol"                    "Ibrutinib" "-0.7968"
## [330,] "330"   "Seocalcitol"                        "Ibrutinib" "3.7612"
## [331,] "331"   "methyl jasmonate"                   "Ibrutinib" "2.8518"
## [332,] "332"   "Bicalutamide"                       "Ibrutinib" "-6.2474"
## [333,] "333"   "Andarine"                           "Ibrutinib" "-2.2026"
## [334,] "334"   "MDV-3100"                           "Ibrutinib" "-3.5389"
## [335,] "335"   "RD-162"                             "Ibrutinib" "-1.6821"
## [336,] "336"   "CITCO"                              "Ibrutinib" "2.5567"
## [337,] "337"   "PK-11195"                           "Ibrutinib" "-5.393"
## [338,] "338"   "Pioglitazone HCl"                   "Ibrutinib" "-3.2065"
## [339,] "339"   "GW-7647"                            "Ibrutinib" "-5.1003"
## [340,] "340"   "GW-0742"                            "Ibrutinib" "-1.578"
## [341,] "341"   "GW-501516"                          "Ibrutinib" "2.0511"
## [342,] "342"   "Troglitazone"                       "Ibrutinib" "-5.9511"
## [343,] "343"   "Telmisartan"                        "Ibrutinib" "-8.6714"
## [344,] "344"   "GSK-3787"                           "Ibrutinib" "-0.1895"
## [345,] "345"   "MK-767"                             "Ibrutinib" "-0.5208"
## [346,] "346"   "SID 7969543"                        "Ibrutinib" "9.056"
## [347,] "347"   "Bazedoxifene"                       "Ibrutinib" "-2.8463"
## [348,] "348"   "Triptolide"                         "Ibrutinib" "6.637"
## [349,] "349"   "Apratastat"                         "Ibrutinib" "0.0233"
## [350,] "350"   "HC-067047"                          "Ibrutinib" "-0.2501"
## [351,] "351"   "DE-096"                             "Ibrutinib" "8.3289"
## [352,] "352"   "GSK-3965"                           "Ibrutinib" "-2.2712"
## [353,] "353"   "Omeprazole"                         "Ibrutinib" "4.6107"
## [354,] "354"   "Elacridar"                          "Ibrutinib" "-0.3537"
## [355,] "355"   "Secin H3"                           "Ibrutinib" "0.0463"
## [356,] "356"   "CDIBA"                              "Ibrutinib" "9.2589"
```

```
## [357,] "357"   "U-73122??"                      "Ibrutinib" "4.6077"
## [358,] "358"   "BAY-60-7550"                    "Ibrutinib" "-9.1944"
## [359,] "359"   "anagrelide"                     "Ibrutinib" "-7.7538"
## [360,] "360"   "Olprinone"                      "Ibrutinib" "-2.2559"
## [361,] "361"   "NCGC00168459-01"                "Ibrutinib" "2.1073"
## [362,] "362"   "NCGC00249759-01"                "Ibrutinib" "-4.5155"
## [363,] "363"   "NVP-ABE171"                     "Ibrutinib" "-2.8188"
## [364,] "364"   "Lirimilast"                     "Ibrutinib" "-1.961"
## [365,] "365"   "Sildenafil citrate"             "Ibrutinib" "0.9777"
## [366,] "366"   "Alacepril"                      "Ibrutinib" "2.1223"
## [367,] "367"   "Ubenimex"                       "Ibrutinib" "-1.4994"
## [368,] "368"   "BAY-41-8543"                    "Ibrutinib" "-1.0795"
## [369,] "369"   "Vinpocetine"                    "Ibrutinib" "-2.9691"
## [370,] "370"   "Verapamil"                      "Ibrutinib" "0.7655"
## [371,] "371"   "(S)-(+)-Niguldipine"            "Ibrutinib" "0.3308"
## [372,] "372"   "SR 33805"                       "Ibrutinib" "2.6059"
## [373,] "373"   "Zaldaride maleate"              "Ibrutinib" "-0.2416"
## [374,] "374"   "Riluzole"                       "Ibrutinib" "-5.0337"
## [375,] "375"   "SSR-504734"                     "Ibrutinib" "-1.7259"
## [376,] "376"   "SCH-900435"                     "Ibrutinib" "3.9508"
## [377,] "377"   "Ivacaftor"                      "Ibrutinib" "-1.5649"
## [378,] "378"   "Ivabradine hydrochloride"       "Ibrutinib" "-2.1055"
## [379,] "379"   "ICI-D7288"                      "Ibrutinib" "-1.9585"
## [380,] "380"   "GSK-1016790A"                   "Ibrutinib" "-0.4923"
## [381,] "381"   "Fenobam"                        "Ibrutinib" "1.473"
## [382,] "382"   "MPEP"                           "Ibrutinib" "-4.0393"
## [383,] "383"   "Piboserod hydrochloride"        "Ibrutinib" "1.9556"
## [384,] "384"   "SB-224289"                      "Ibrutinib" "6.5923"
## [385,] "385"   "SB-206553"                      "Ibrutinib" "-4.9748"
## [386,] "386"   "Meclinertant"                   "Ibrutinib" "1.6796"
## [387,] "387"   "Zibotentan"                     "Ibrutinib" "-0.9321"
## [388,] "388"   "GW-4064X"                       "Ibrutinib" "-3.4697"
## [389,] "389"   "Turofexorate isopropyl"         "Ibrutinib" "-1.1582"
## [390,] "390"   "GW-9508"                        "Ibrutinib" "-2.1943"
## [391,] "391"   "AG-041R"                        "Ibrutinib" "5.2175"
## [392,] "392"   "MK-0354"                        "Ibrutinib" "-5.3955"
## [393,] "393"   "PB-28"                          "Ibrutinib" "0.7069"
## [394,] "394"   "Cutamesine hydrochloride"       "Ibrutinib" "-0.0496"
## [395,] "395"   "BD-1047"                        "Ibrutinib" "-0.0756"
## [396,] "396"   "SCH-79797"                      "Ibrutinib" "2.2604"
## [397,] "397"   "Vorapaxar"                      "Ibrutinib" "0.882"
## [398,] "398"   "GB 83"                          "Ibrutinib" "0.3567"
## [399,] "399"   "Aprepitant"                     "Ibrutinib" "4.3963"
## [400,] "400"   "GR-159897"                      "Ibrutinib" "-1.0661"
## [401,] "401"   "Osanetant"                      "Ibrutinib" "-0.0306"
## [402,] "402"   "Rolofylline"                    "Ibrutinib" "-3.9131"
## [403,] "403"   "Maraviroc"                      "Ibrutinib" "-5.8541"
## [404,] "404"   "LY-320135"                      "Ibrutinib" "-5.4082"
## [405,] "405"   "ACPA"                           "Ibrutinib" "-8.9368"
## [406,] "406"   "MK-886"                         "Ibrutinib" "-0.0876"
## [407,] "407"   "Veliflapon"                     "Ibrutinib" "-5.5199"
## [408,] "408"   "Licofelone"                     "Ibrutinib" "-6.338"
## [409,] "409"   "Zileuton"                       "Ibrutinib" "1.0959"
```

```
## [410,] "410"   "NCGC00185053"                     "Ibrutinib" "1.8018"
## [411,] "411"   "Celecoxib"                        "Ibrutinib" "0.628"
## [412,] "412"   "Pravadoline"                      "Ibrutinib" "-2.4313"
## [413,] "413"   "PF-3845"                          "Ibrutinib" "-5.4409"
## [414,] "414"   "Fluvastatin"                      "Ibrutinib" "-1.3686"
## [415,] "415"   "Simvastatin"                      "Ibrutinib" "-6.5355"
## [416,] "416"   "Dalcetrapib"                      "Ibrutinib" "1.0539"
## [417,] "417"   "JK 184"                           "Ibrutinib" "4.3315"
## [418,] "418"   "NCGC00037850"                     "Ibrutinib" "-2.3021"
## [419,] "419"   "CAY10581"                         "Ibrutinib" "2.8465"
## [420,] "420"   "EBPC"                             "Ibrutinib" "-3.3191"
## [421,] "421"   "Piraxostat"                       "Ibrutinib" "5.9882"
## [422,] "422"   "JZL-184"                          "Ibrutinib" "-2.9316"
## [423,] "423"   "GW 4869"                          "Ibrutinib" "-5.0537"
## [424,] "424"   "Ezatiostat"                       "Ibrutinib" "0.3211"
## [425,] "425"   "Vapiprost hydrochloride"          "Ibrutinib" "-0.0533"
## [426,] "426"   "Ezetimibe"                        "Ibrutinib" "-0.8794"
## [427,] "427"   "GSK-1995010"                      "Ibrutinib" "0.9241"
## [428,] "428"   "FASN BI"                          "Ibrutinib" "1.6052"
## [429,] "429"   "GSK837149A"                       "Ibrutinib" "6.0655"
## [430,] "430"   "FASN MRK"                         "Ibrutinib" "-0.0071"
## [431,] "431"   "Daporinad"                        "Ibrutinib" "-2.8081"
## [432,] "432"   "cPEPCK inhibitor"                 "Ibrutinib" "0.9414"
## [433,] "433"   "GLS968"                           "Ibrutinib" "-0.0824"
## [434,] "434"   "IPFK2"                            "Ibrutinib" "4.9532"
## [435,] "435"   "NCGC00186528"                     "Ibrutinib" "-3.1249"
## [436,] "436"   "NCGC00185916"                     "Ibrutinib" "1.8846"
## [437,] "437"   "AZD-7545"                         "Ibrutinib" "-1.9476"
## [438,] "438"   "PDHK RIKEN"                       "Ibrutinib" "-0.6746"
## [439,] "439"   "NCGC00262689"                     "Ibrutinib" "-0.8472"
## [440,] "440"   "IDH-001"                          "Ibrutinib" "-1.5858"
## [441,] "441"   "AR-C155858"                       "Ibrutinib" "5.8313"
## [442,] "442"   "CAP-232"                          "Ibrutinib" "0.5516"
## [443,] "443"   "NCGC00238624"                     "Ibrutinib" "0.5642"
## [444,] "444"   "ACC1 BMS"                         "Ibrutinib" "-2.1855"
## [445,] "445"   "Ibutamoren mesilate"              "Ibrutinib" "-1.0902"
## [446,] "446"   "ML 141"                           "Ibrutinib" "3.1015"
## [447,] "447"   "QS11"                             "Ibrutinib" "-5.7021"
## [448,] "448"   "Gavestinel sodium"                "Ibrutinib" "-0.5013"
## [449,] "449"   "Eliprodil"                        "Ibrutinib" "0.7497"
## [450,] "450"   "Caroverine hydrochloride"         "Ibrutinib" "2.8002"
## [451,] "451"   "GYKI-53655"                       "Ibrutinib" "5.3415"
## [452,] "452"   "Dapagliflozin"                    "Ibrutinib" "-0.2877"
## [453,] "453"   "GSK-4112"                         "Ibrutinib" "-1.966"
## [454,] "454"   "Sepantronium bromide"             "Ibrutinib" "1.723"
## [455,] "455"   "BIBR 1532"                        "Ibrutinib" "0.8965"
## [456,] "456"   "ITX3"                             "Ibrutinib" "4.5565"
## [457,] "457"   "Salinomycin"                      "Ibrutinib" "6.1848"
## [458,] "458"   "Efavirenz"                        "Ibrutinib" "-3.7459"
## [459,] "459"   "Ivachtin"                         "Ibrutinib" "-0.7658"
## [460,] "460"   "PAC-1"                            "Ibrutinib" "2.345"
## [461,] "461"   "Necrostatin-1"                    "Ibrutinib" "5.0675"
## [462,] "462"   "NCGC00262398"                     "Ibrutinib" "-0.31"
```

```
## [463,] "463"   "Tiplasinin"                         "Ibrutinib" "-2.0905"
## [464,] "464"   "ASR-isobudimer-SO2Ph-4-CH2OC(O)NMe2" "Ibrutinib" "0.0207"
## [465,] "465"   "BTM-2C-dimer ketone"                "Ibrutinib" "11.1432"
## [466,] "466"   "BTM-2C-dimer allyl oxime"           "Ibrutinib" "1.0771"
```

Compiling dynamic reporting file

```
#library(knitr)
#library("tools")
#texi2pdf(knit("Delta_calculation_dynamic_report.Rnw"))
```
